# Supplementary figures and images for: Tumorigenicity decrease in Bcl-xL deficient MDCK cells ensuring the safety for influenza vaccine production
Source: PLoS One. 2024 Dec 16;19(12):e0311069. doi: 10.1371/journal.pone.0311069 (PMC11649150; doi:10.1371/journal.pone.0311069)

Unedited gels for Fig 1B

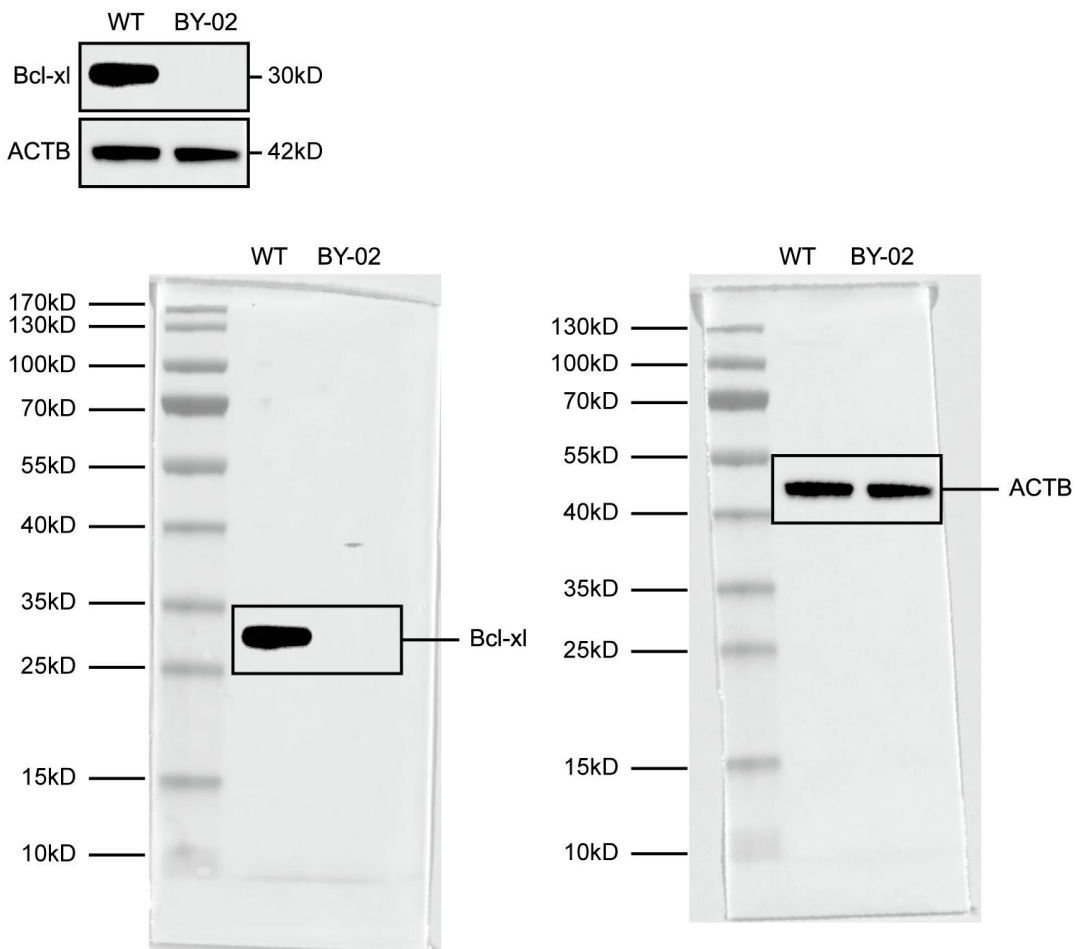

Unedited gels for Fig 4B

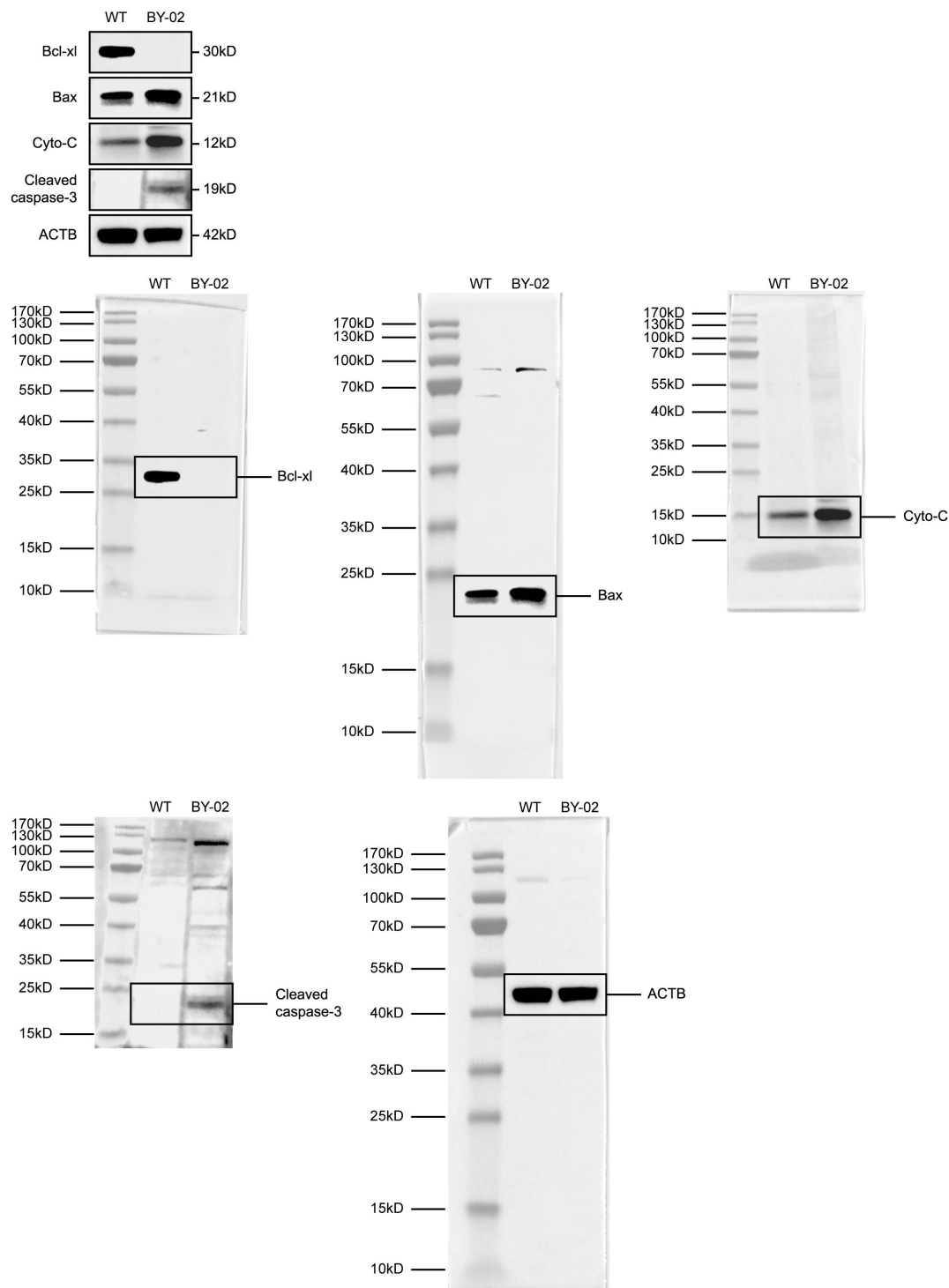

Unedited gels for Fig 4D

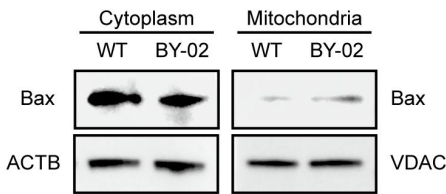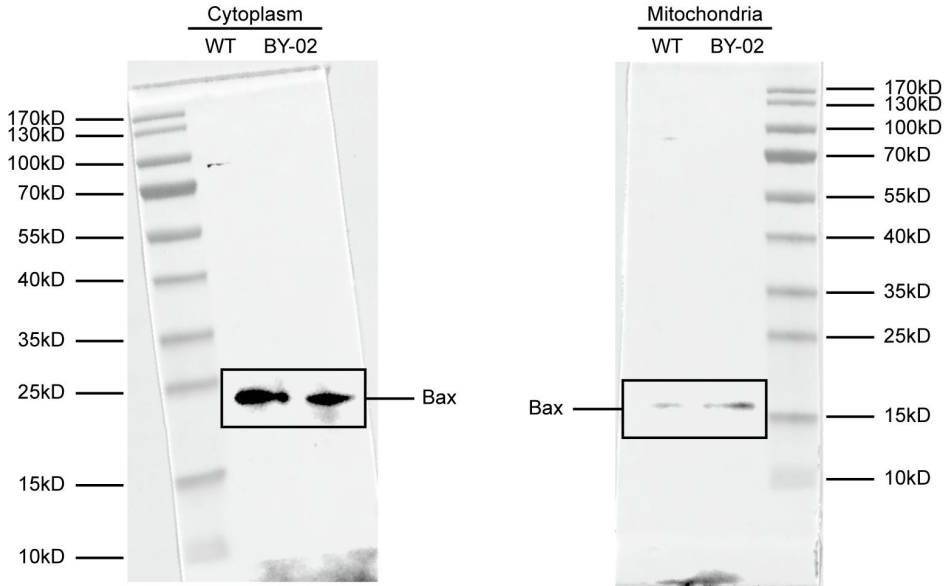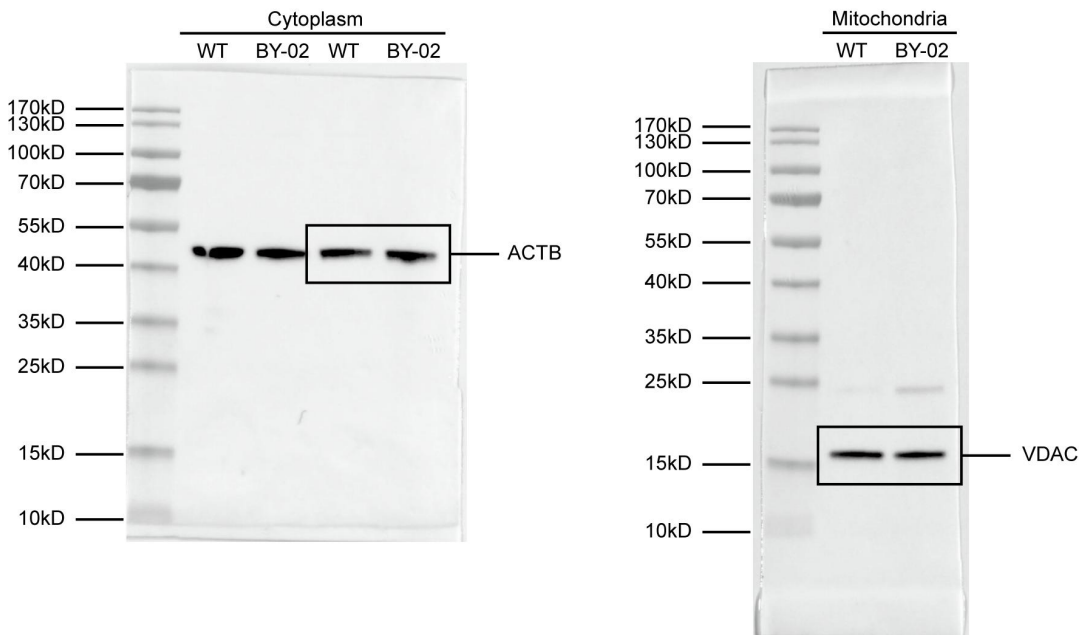

Supplement: S1 Raw image — (PDF) [file pone.0311069.s001.pdf]

Gene Number

800

600

400

200

0

B\_vs\_A

■ up-regulated

■ down-regulated

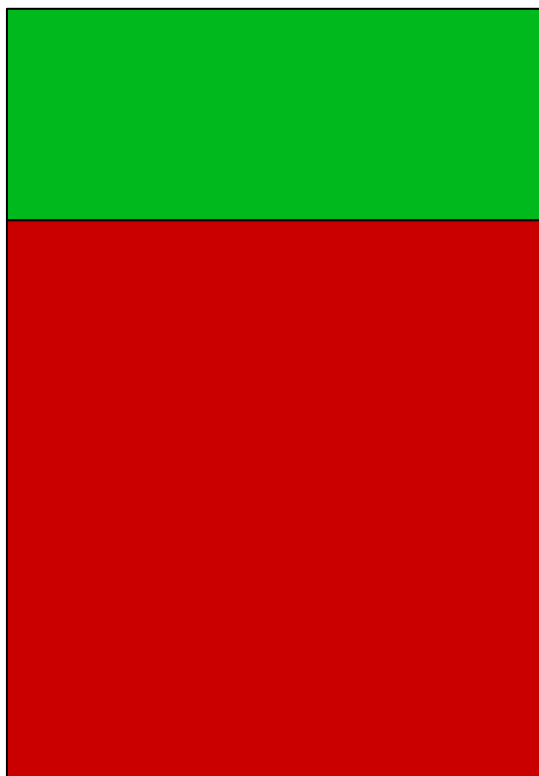

Supplement: S1 File — (ZIP) [file pone.0311069.s002.zip › S2_File/1_DEA/barplot/DE_gene_barplot.pdf]

Transcript Number

1500

1000

500

0

B\_vs\_A

■ up-regulated

■ down-regulated

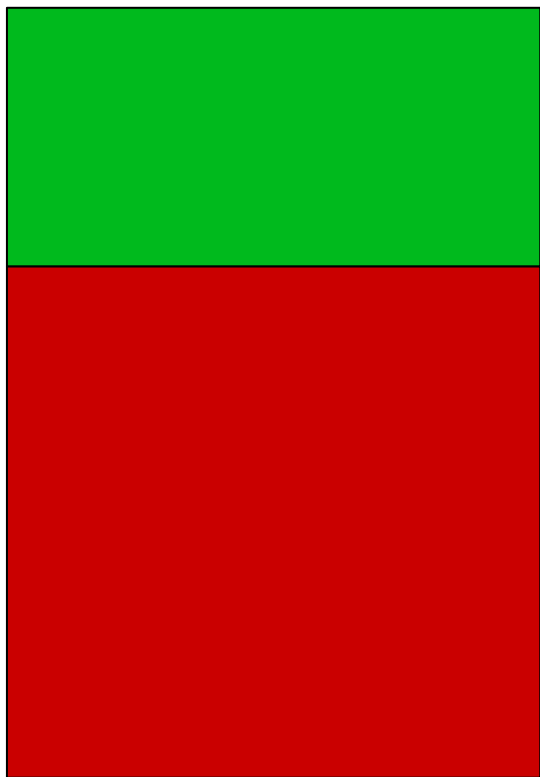

Supplement: S1 File — (ZIP) [file pone.0311069.s002.zip › S2_File/1_DEA/barplot/DE_transcript_barplot.pdf]

**TPM distribution boxplot**

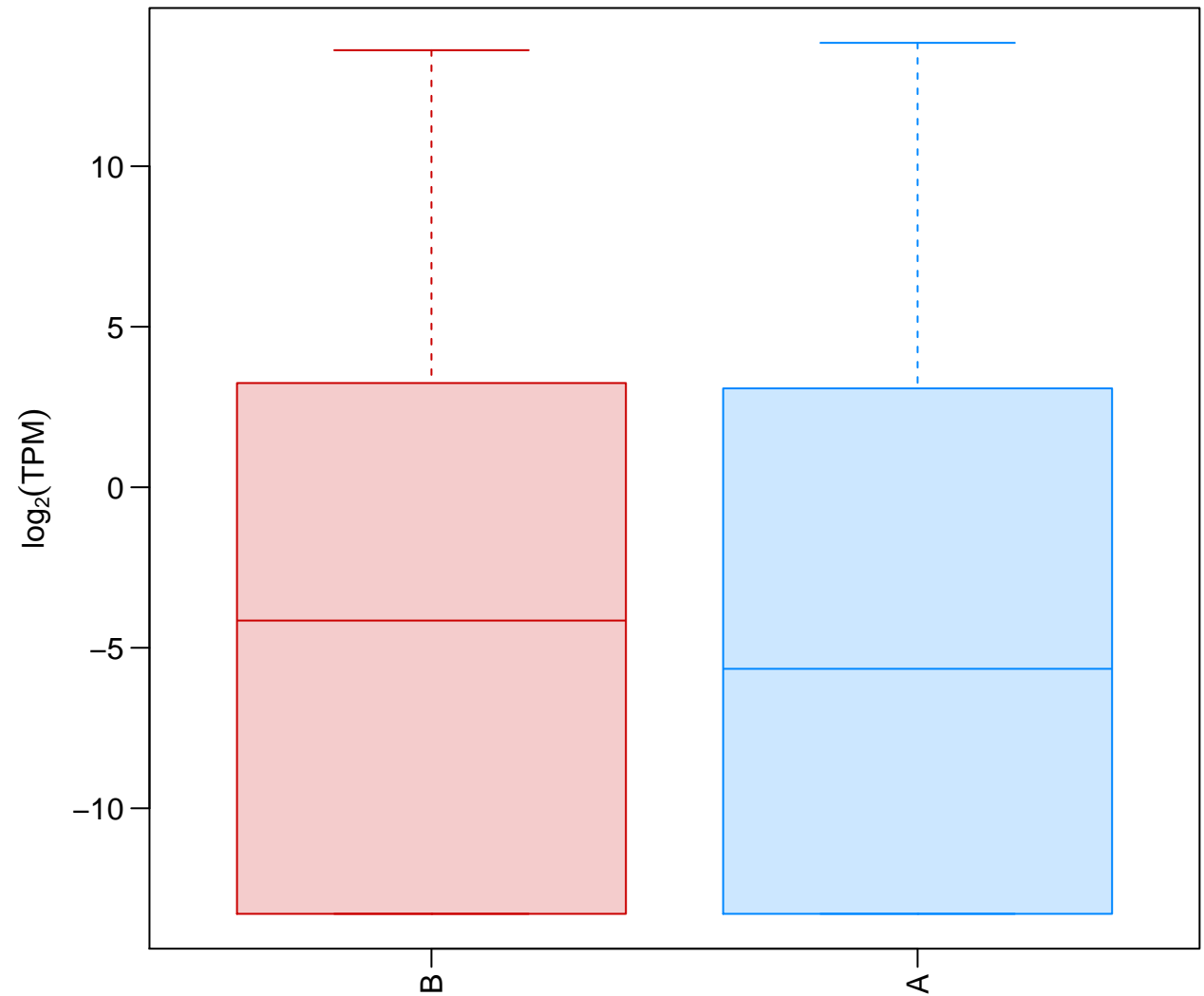

Supplement: S1 File — (ZIP) [file pone.0311069.s002.zip › S2_File/1_DEA/boxplot/B_vs_A.gene_tpm_boxplot.pdf]

**TPM distribution boxplot**

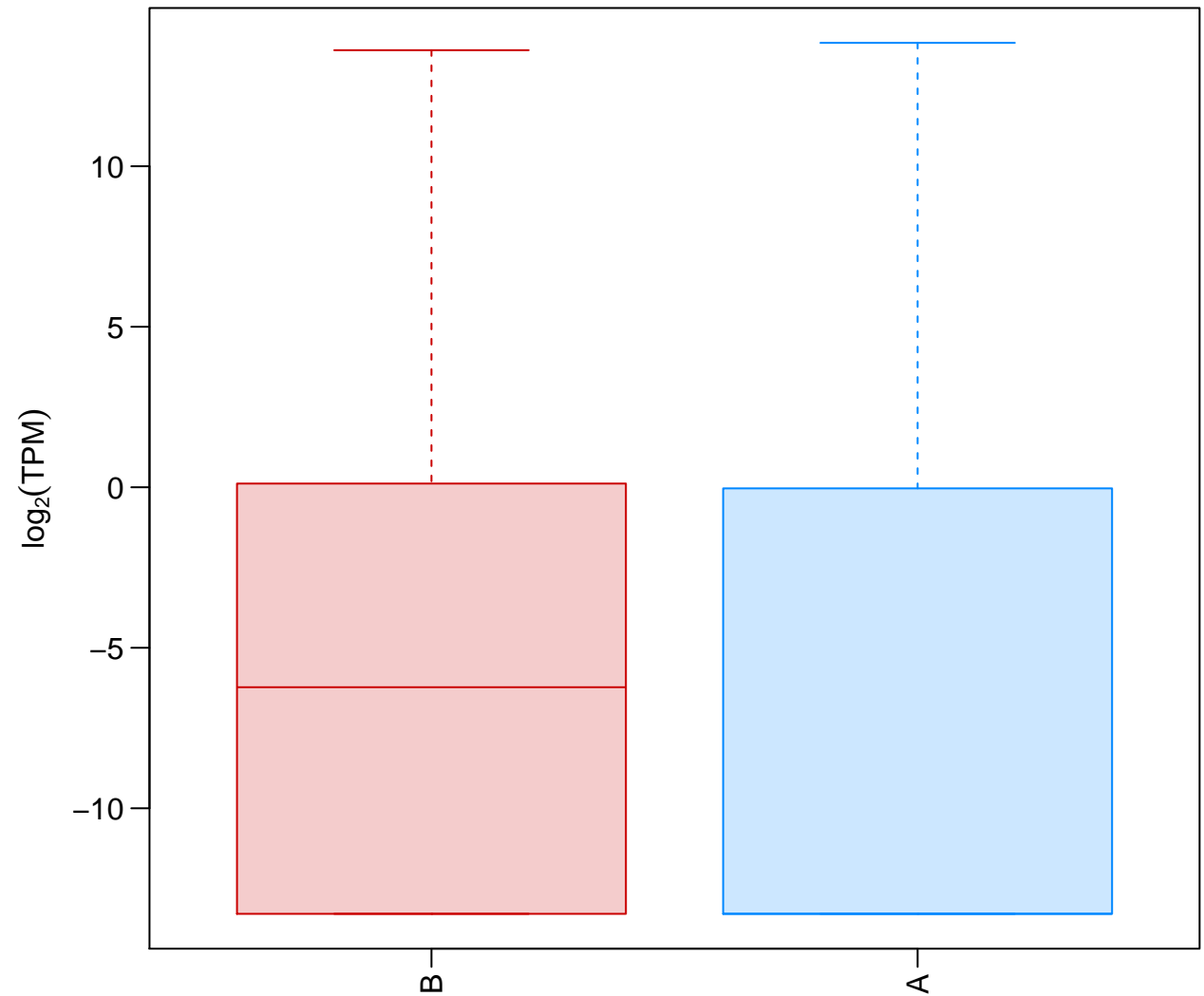

Supplement: S1 File — (ZIP) [file pone.0311069.s002.zip › S2_File/1_DEA/boxplot/B_vs_A.transcript_tpm_boxplot.pdf]

# TPM density distribution

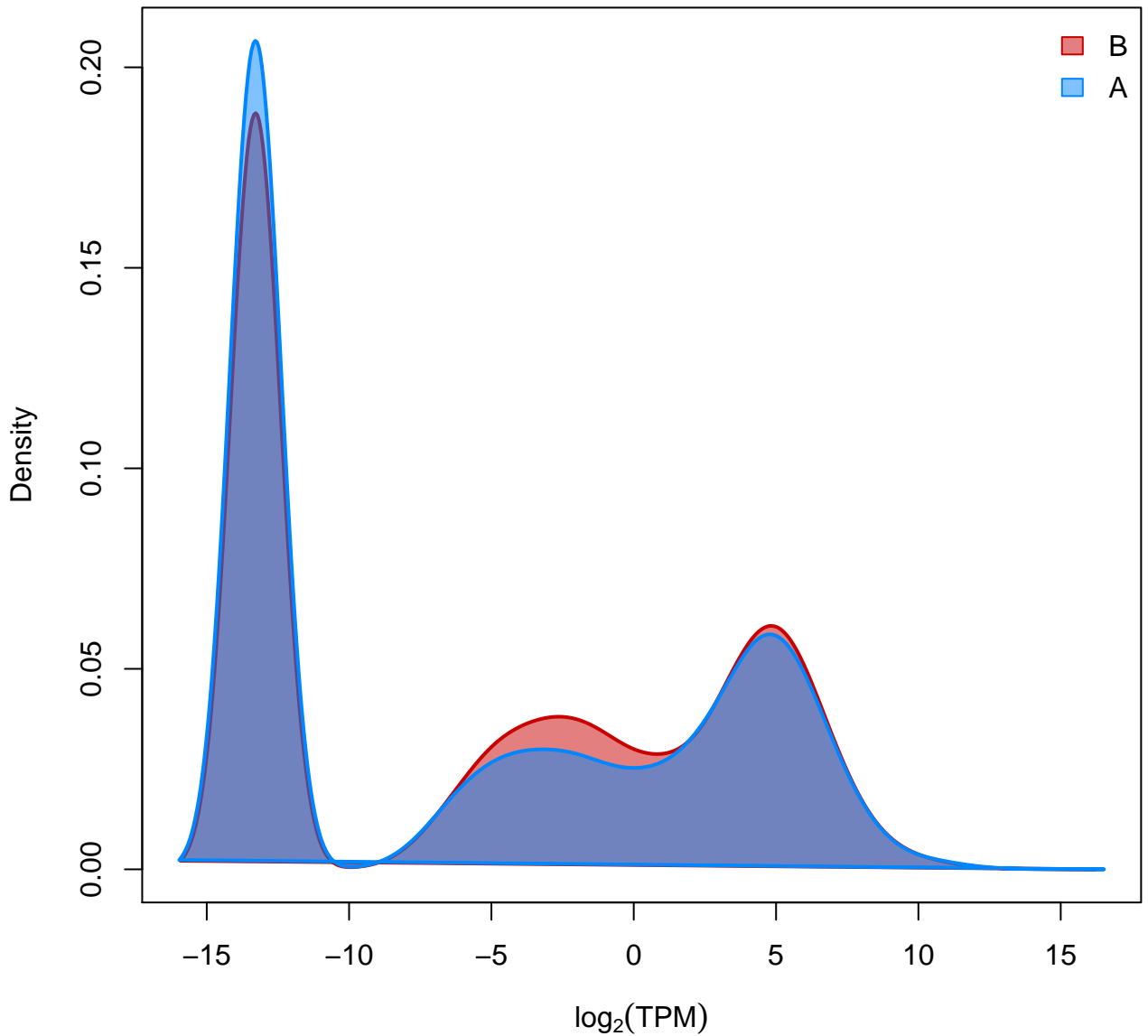

Supplement: S1 File — (ZIP) [file pone.0311069.s002.zip › S2_File/1_DEA/density/B_vs_A.gene_tpm_density.pdf]

# TPM density distribution

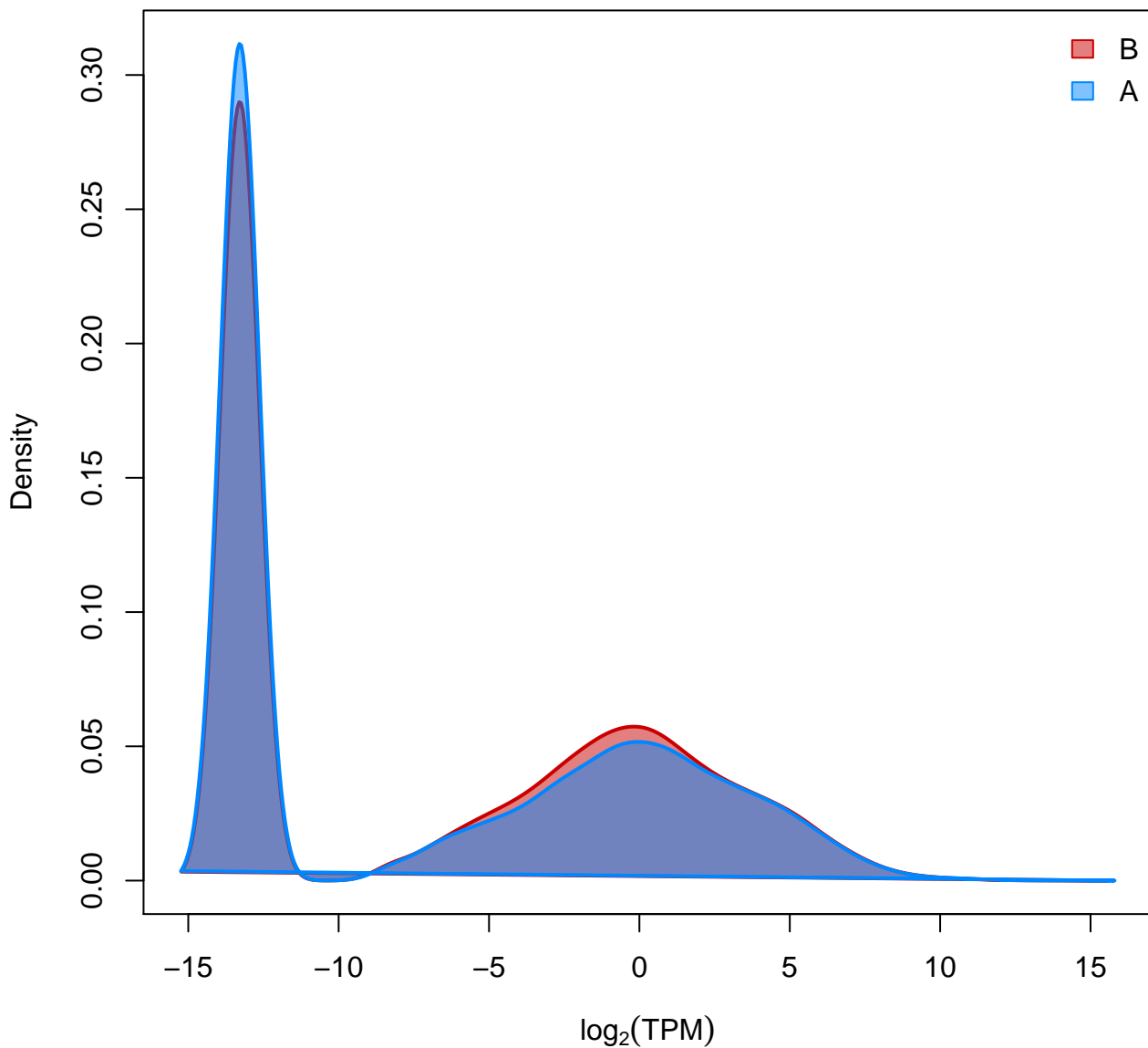

Supplement: S1 File — (ZIP) [file pone.0311069.s002.zip › S2_File/1_DEA/density/B_vs_A.transcript_tpm_density.pdf]

# B VS A

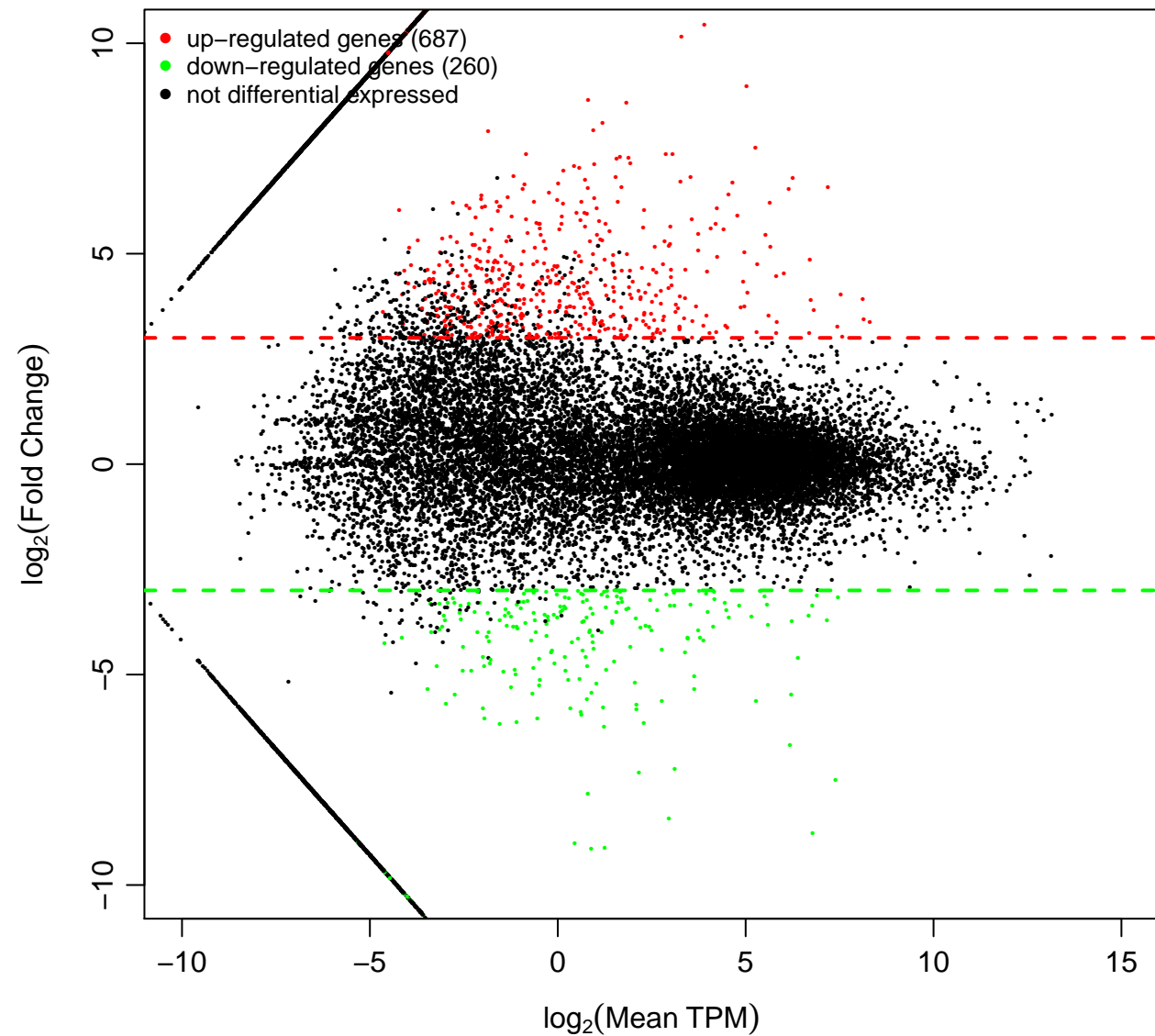

Supplement: S1 File — (ZIP) [file pone.0311069.s002.zip › S2_File/1_DEA/MA/B_vs_A.gene_MA.pdf]

# B VS A

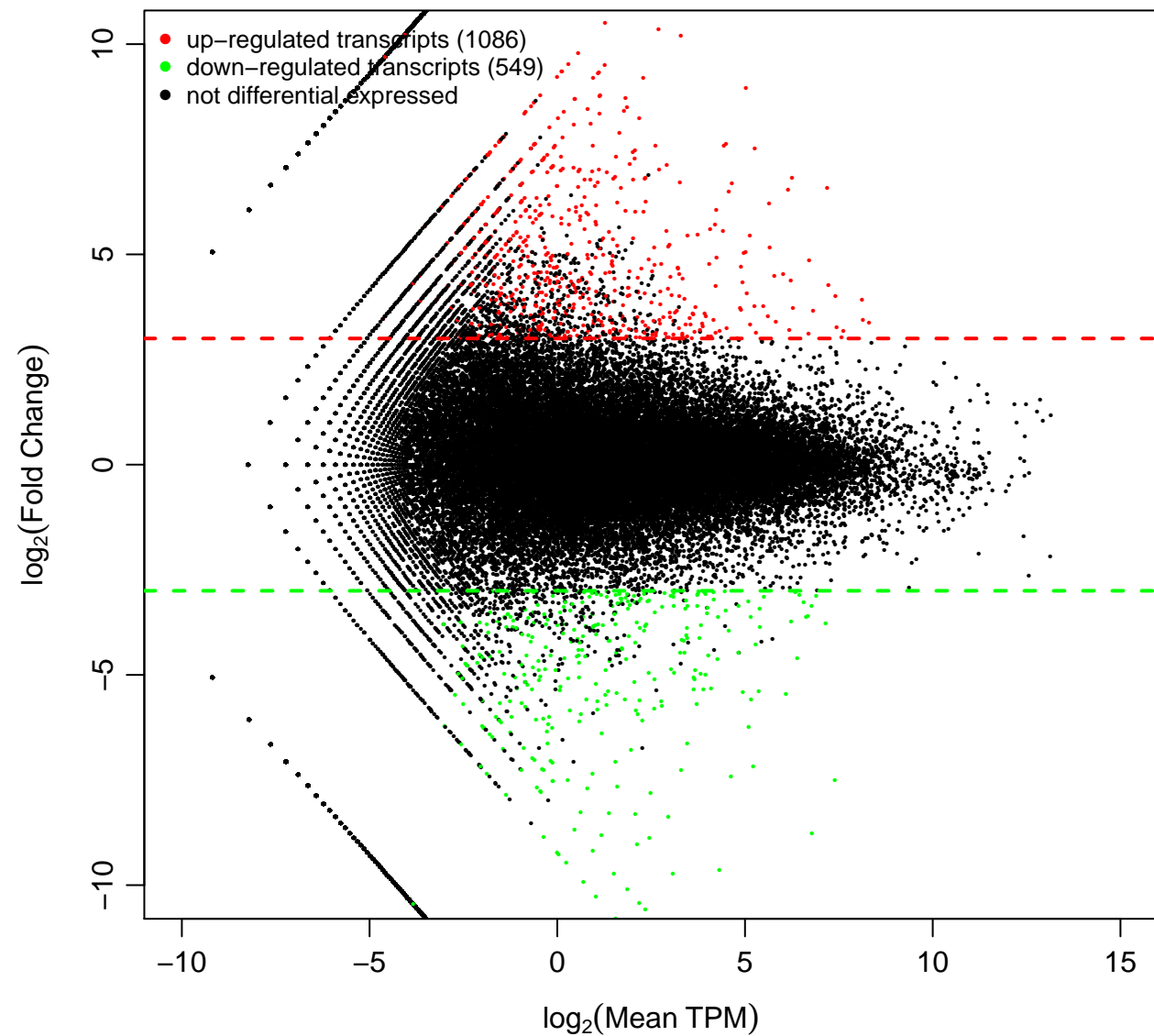

Supplement: S1 File — (ZIP) [file pone.0311069.s002.zip › S2_File/1_DEA/MA/B_vs_A.transcript_MA.pdf]

# B VS A

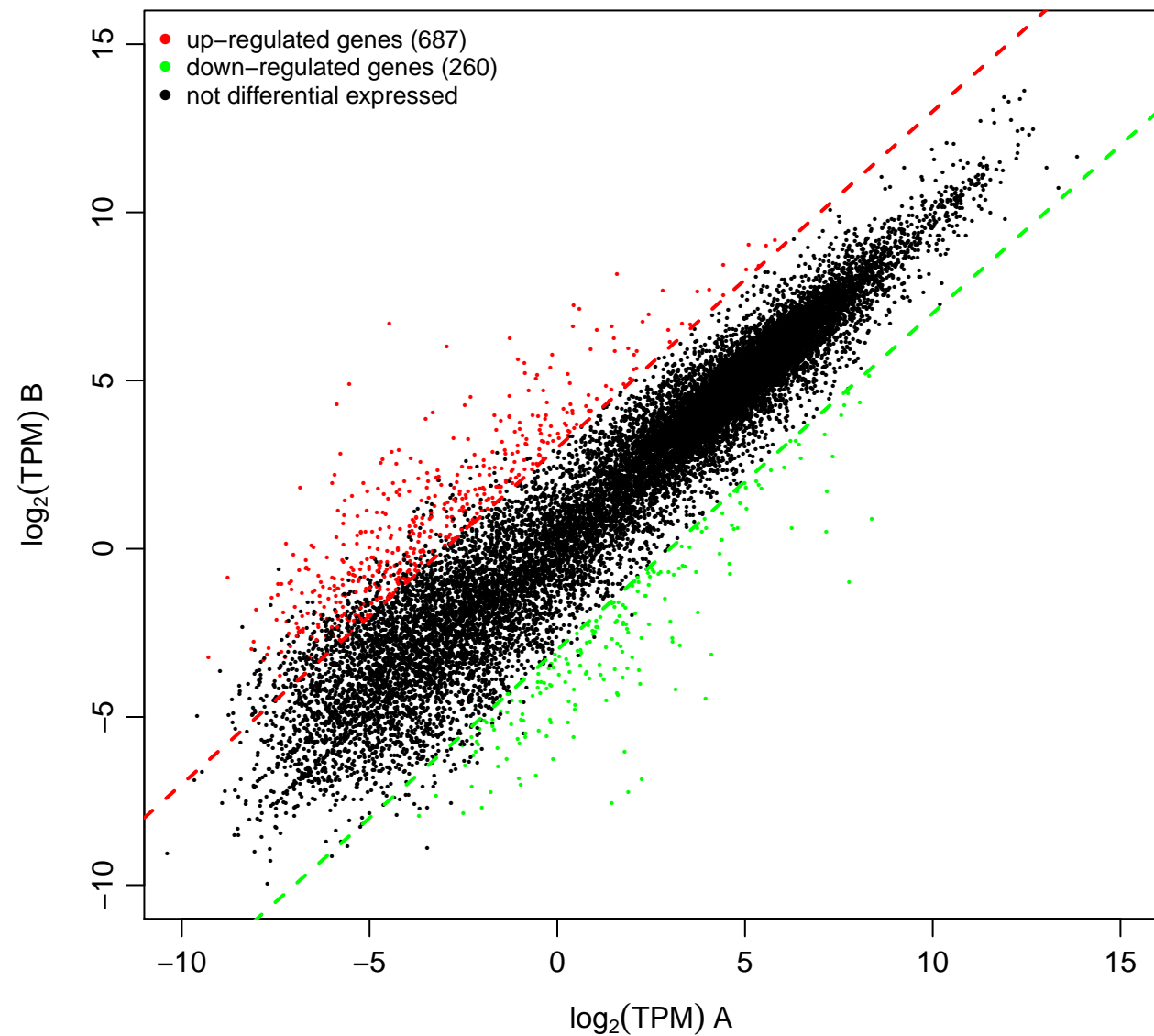

Supplement: S1 File — (ZIP) [file pone.0311069.s002.zip › S2_File/1_DEA/scatter/B_vs_A.gene_scatter.pdf]

# B VS A

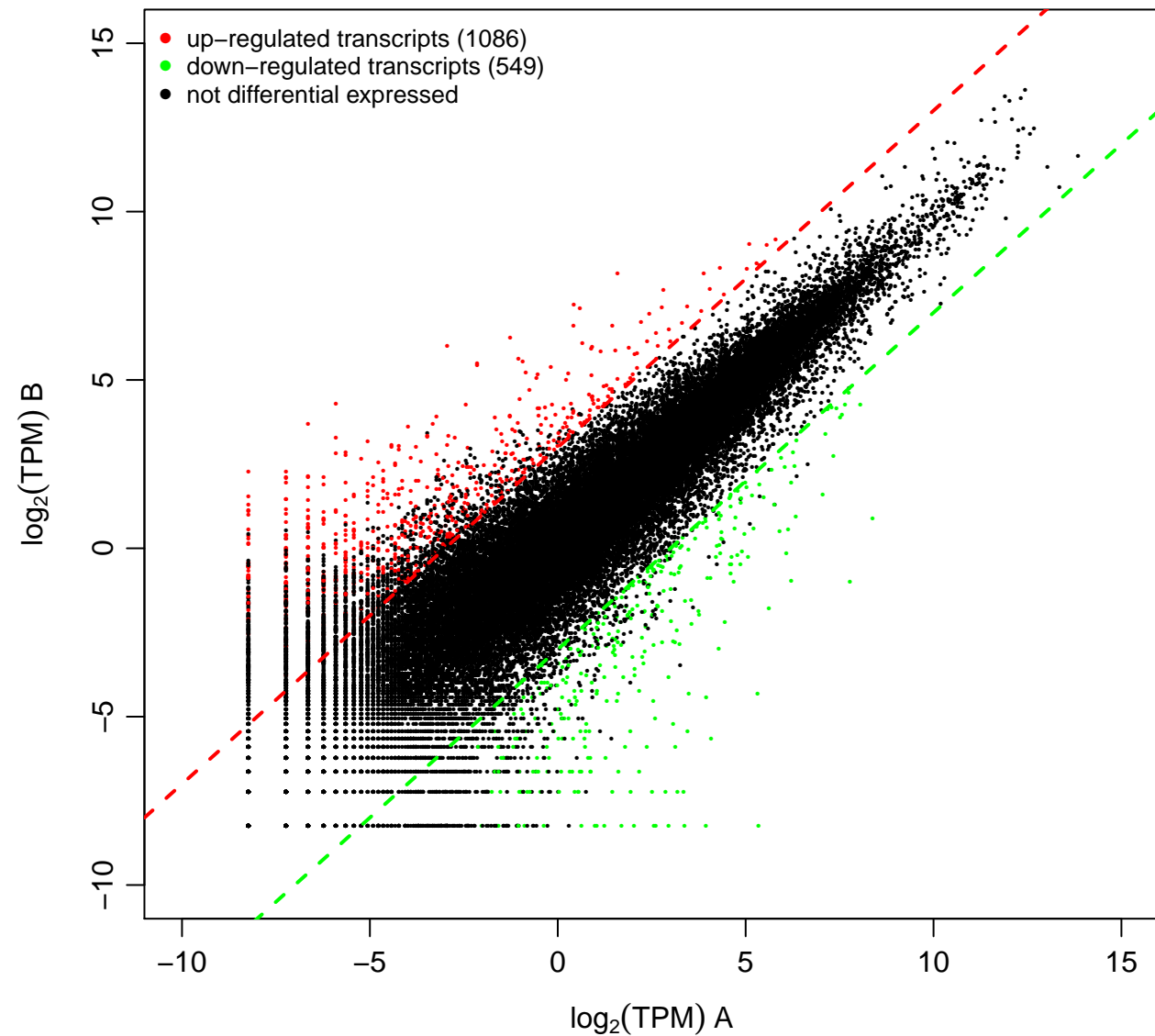

Supplement: S1 File — (ZIP) [file pone.0311069.s002.zip › S2_File/1_DEA/scatter/B_vs_A.transcript_scatter.pdf]

■ A  
■ B

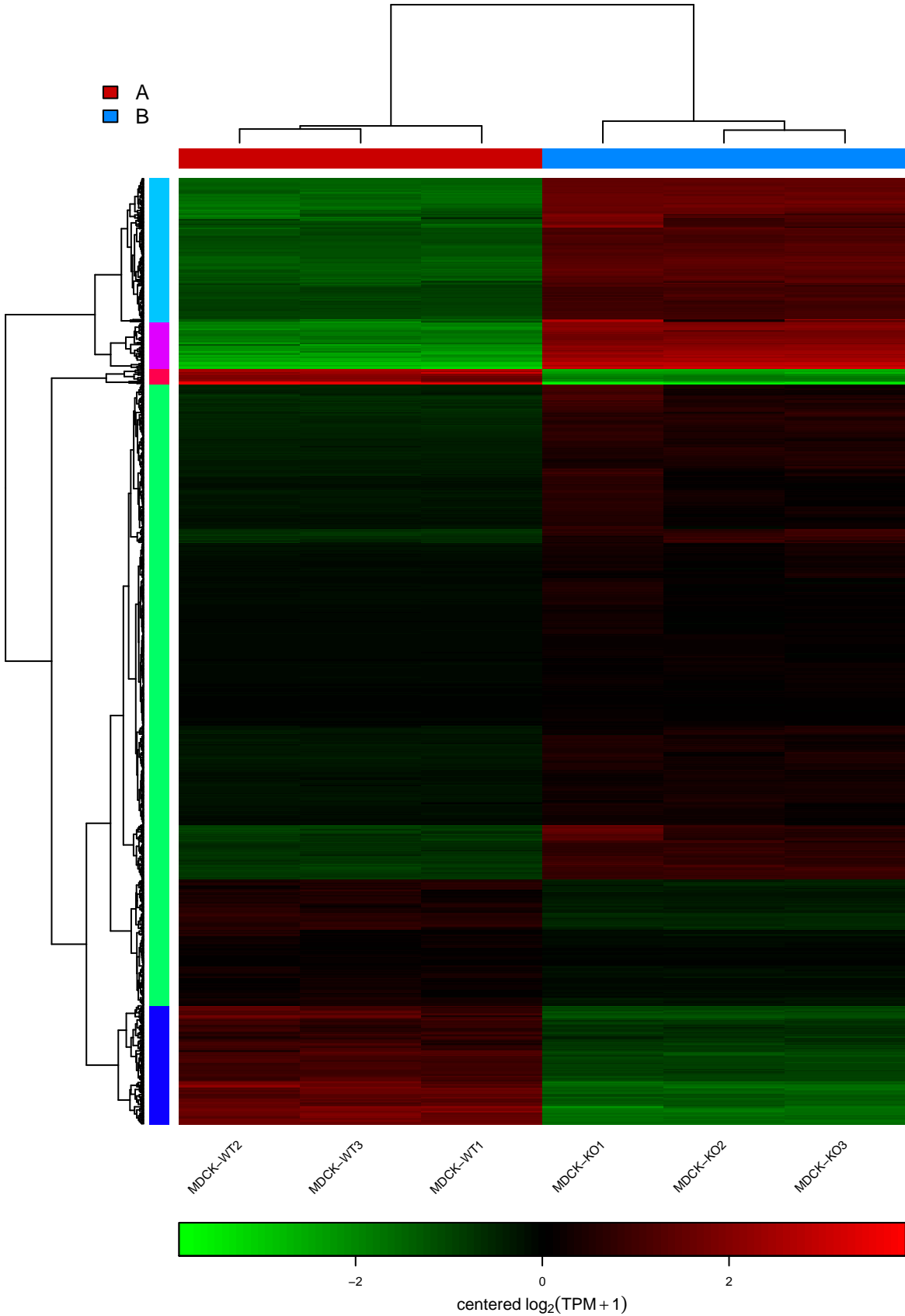

Supplement: S1 File — (ZIP) [file pone.0311069.s002.zip › S2_File/1_DEA/subcluster/union.DE_gene_heatmap.pdf]

**subcluster1, 622genes**

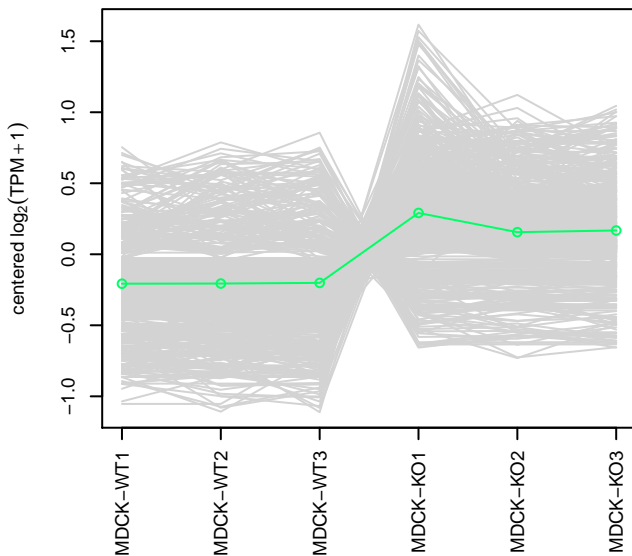

**subcluster2, 144genes**

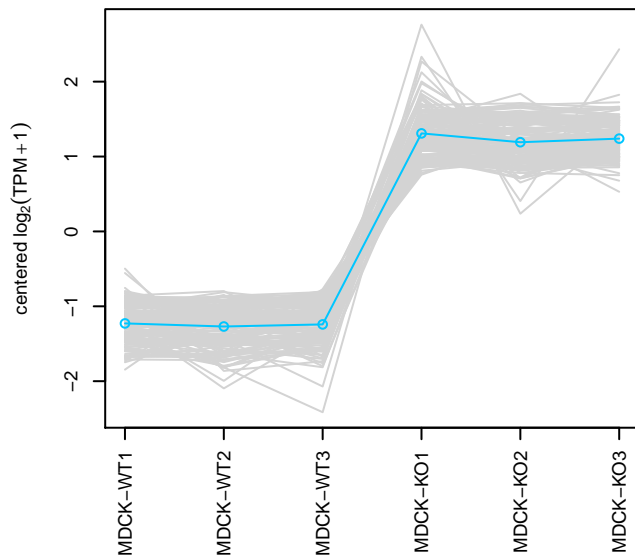

**subcluster3, 119genes**

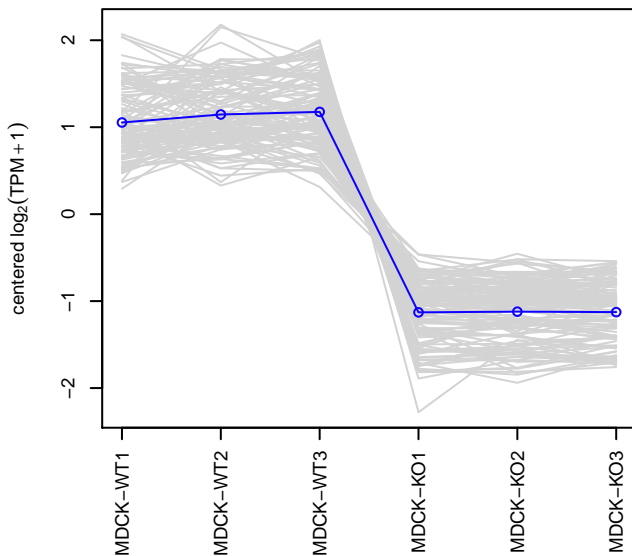

**subcluster4, 47genes**

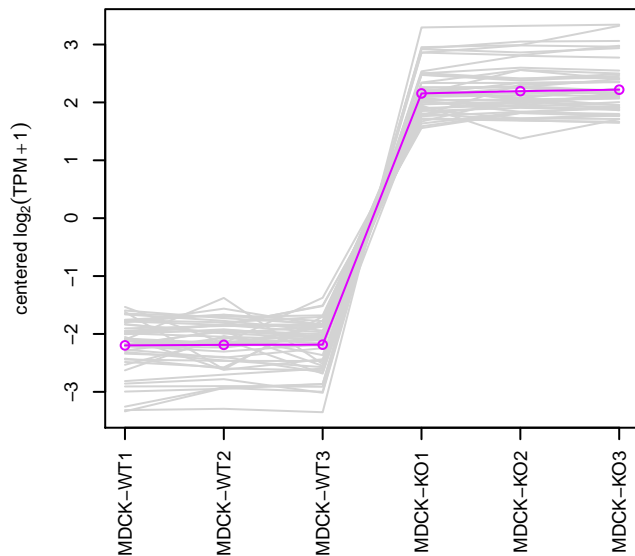

**subcluster5, 15genes**

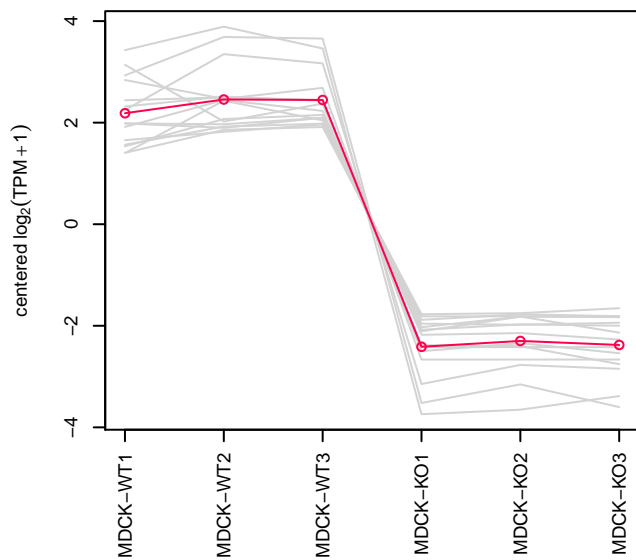

Supplement: S1 File — (ZIP) [file pone.0311069.s002.zip › S2_File/1_DEA/subcluster/union.DE_gene_subcluster.pdf]

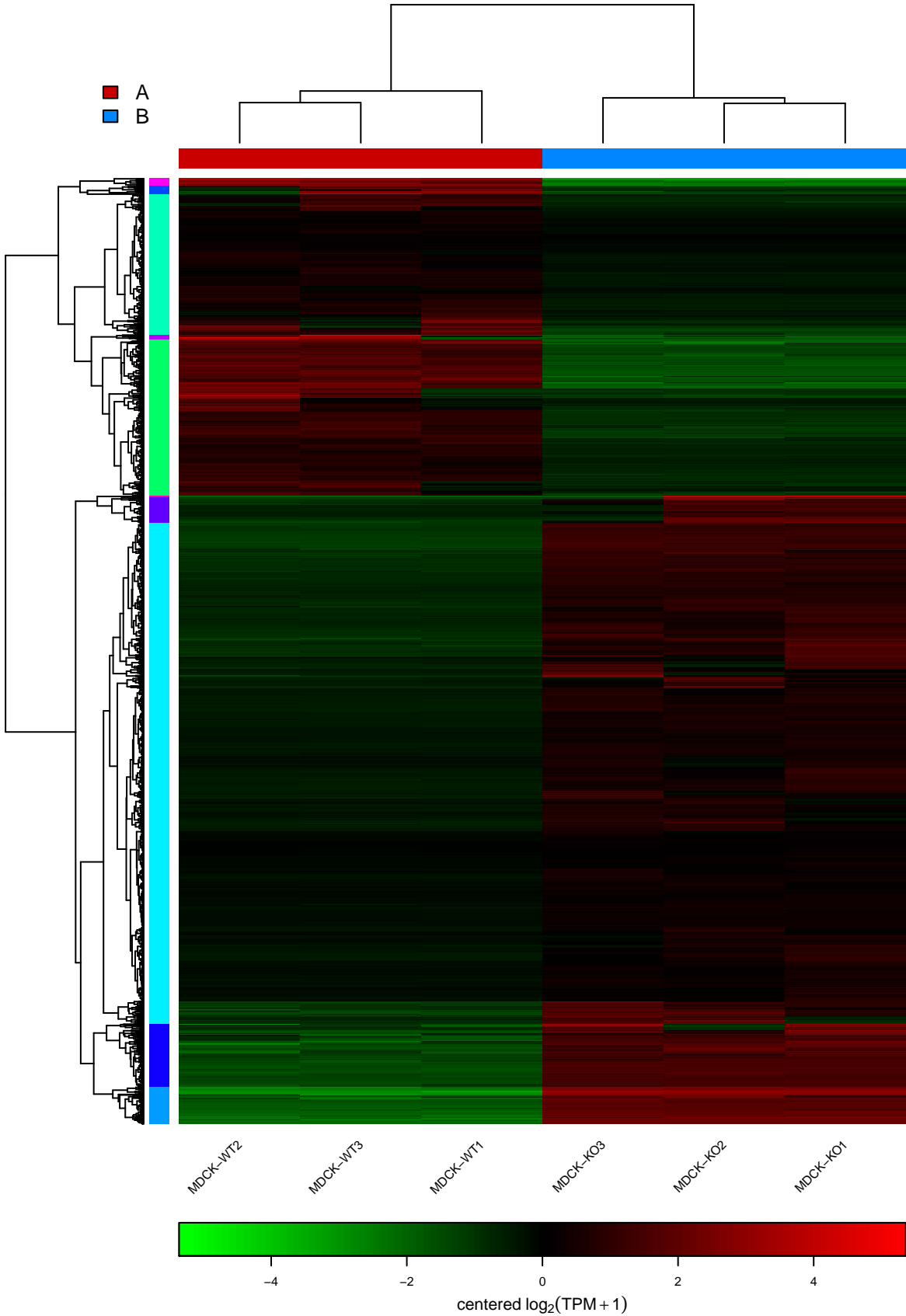

Supplement: S1 File — (ZIP) [file pone.0311069.s002.zip › S2_File/1_DEA/subcluster/union.DE_transcript_heatmap.pdf]

# B VS A

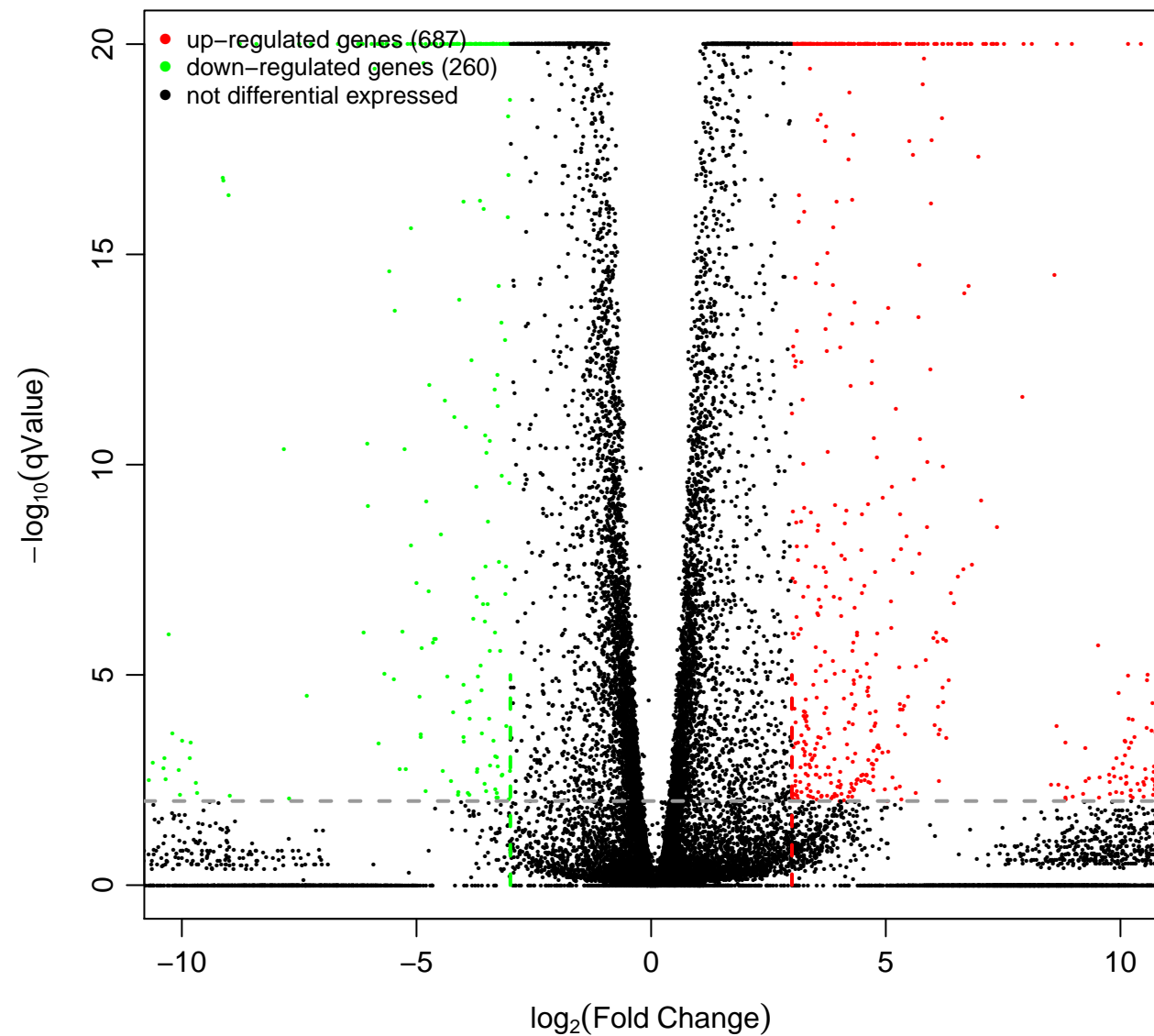

Supplement: S1 File — (ZIP) [file pone.0311069.s002.zip › S2_File/1_DEA/volcano/B_vs_A.gene_volcano.pdf]

# B VS A

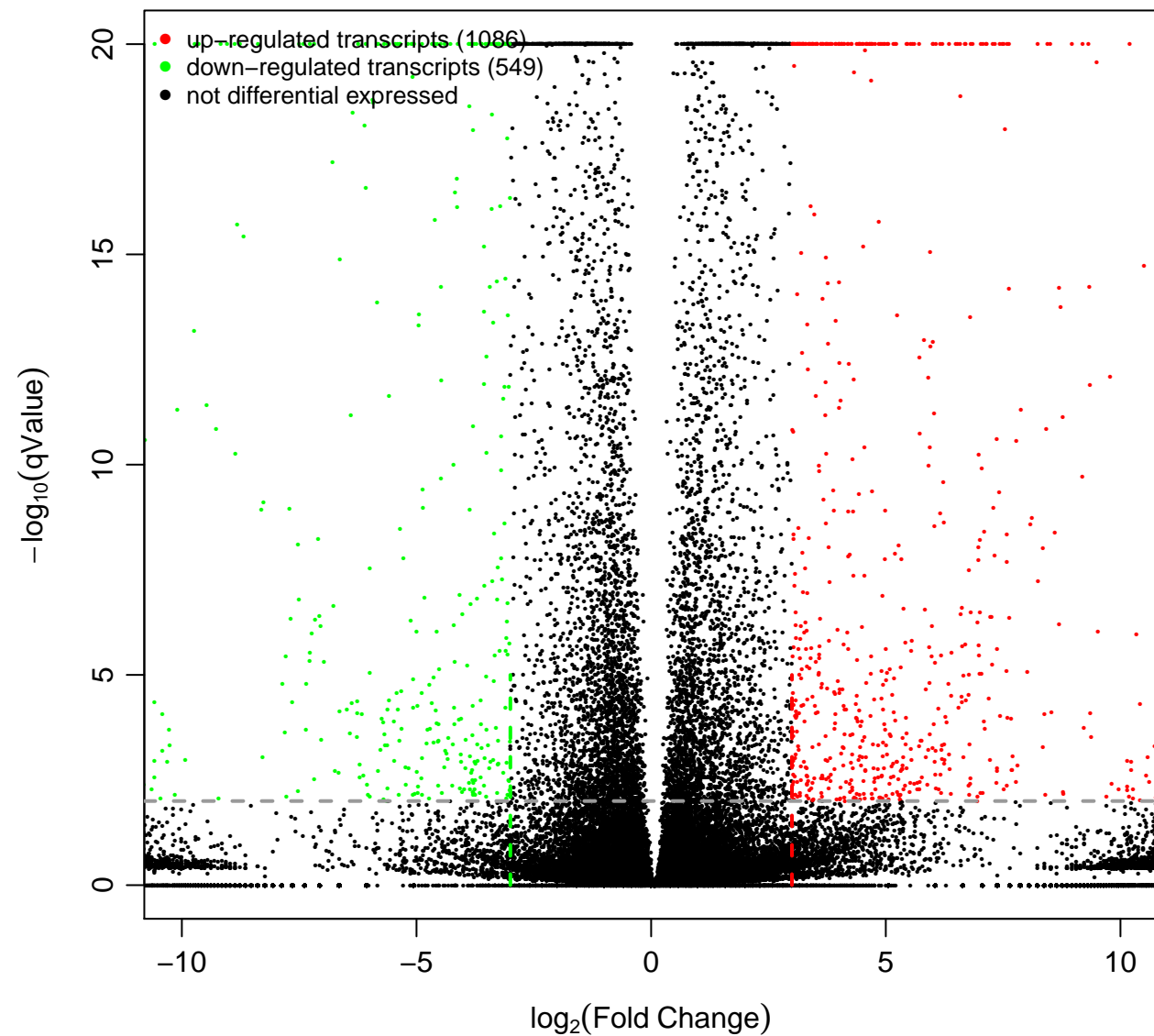

Supplement: S1 File — (ZIP) [file pone.0311069.s002.zip › S2_File/1_DEA/volcano/B_vs_A.transcript_volcano.pdf]

# Gene Function Classification (GO)

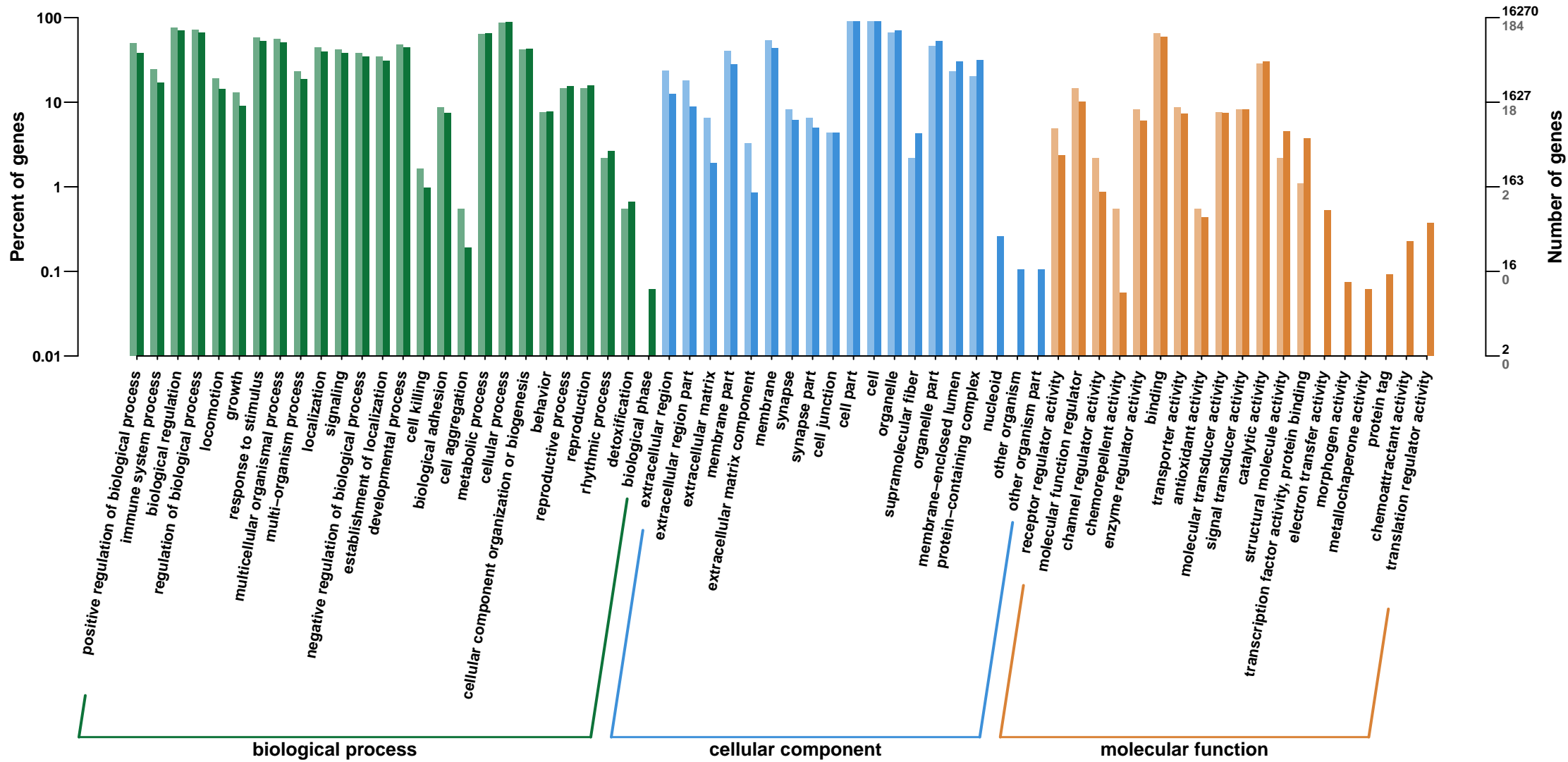

Supplement: S1 File — (ZIP) [file pone.0311069.s002.zip › S2_File/2_GO_enrichment/barplot/B_vs_A.down_GO_categorie.pdf]

# Gene Function Classification (GO)

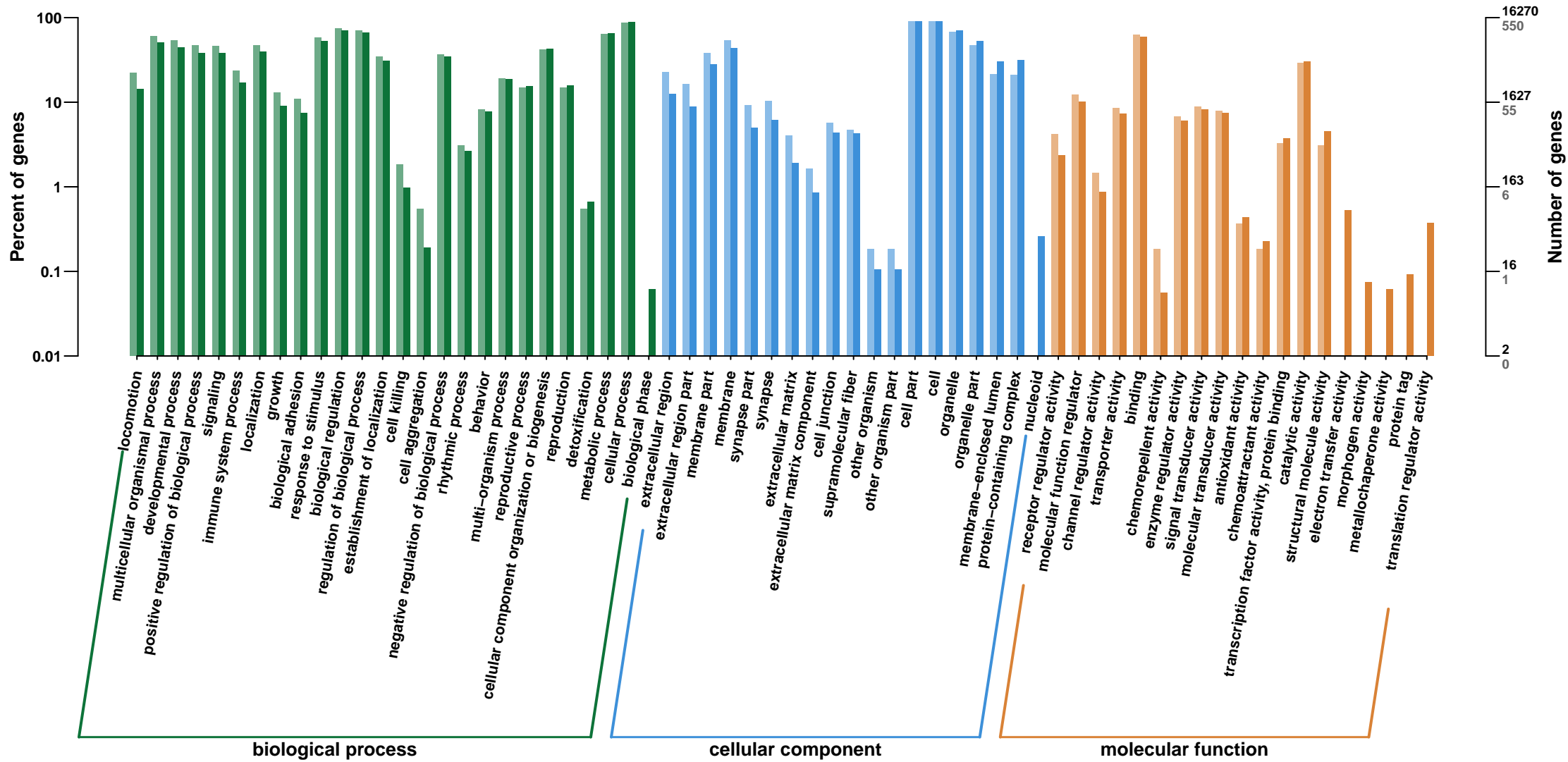

Supplement: S1 File — (ZIP) [file pone.0311069.s002.zip › S2_File/2_GO_enrichment/barplot/B_vs_A.sign_GO_categorie.pdf]

# Gene Function Classification (GO)

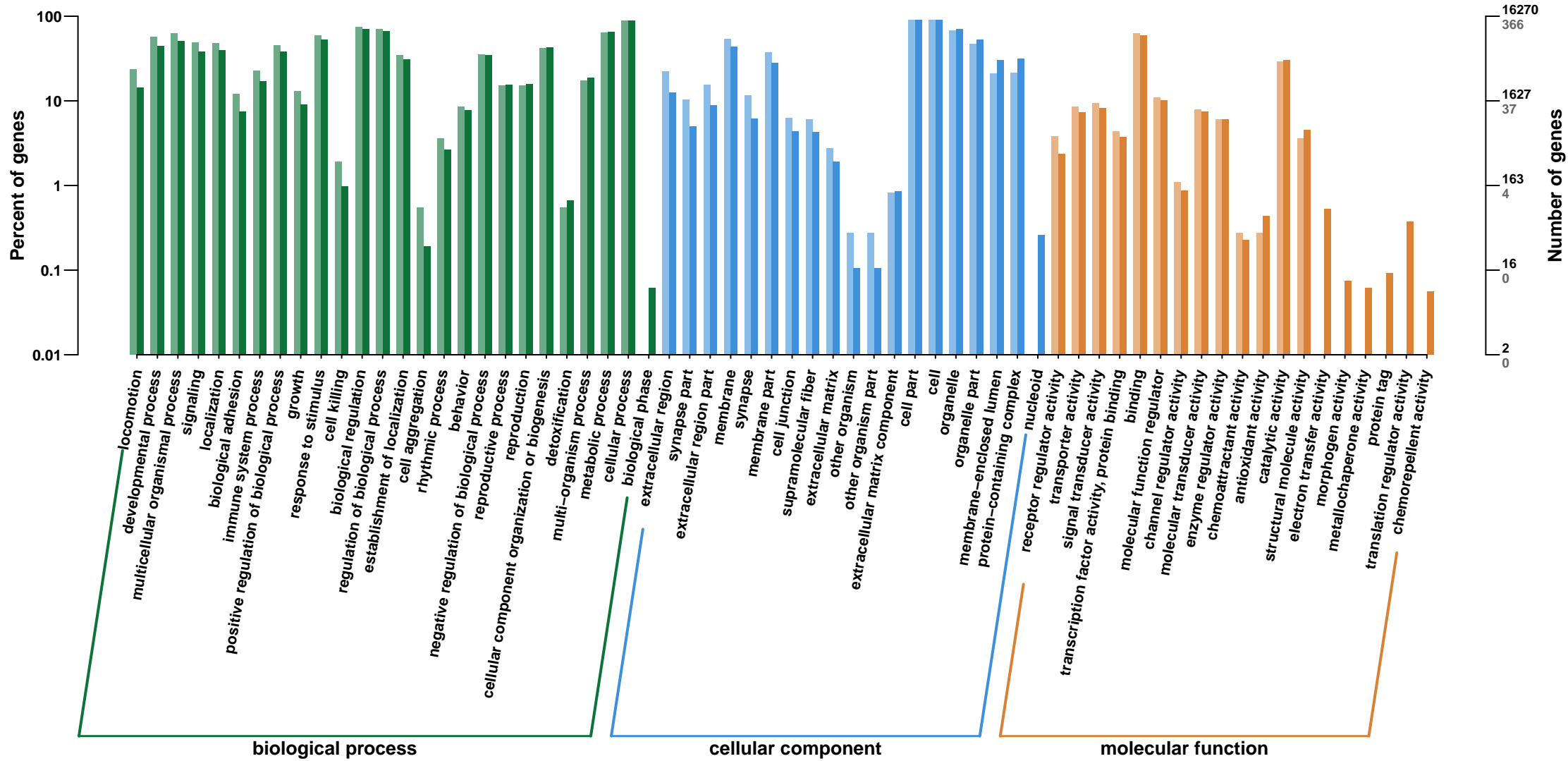

Supplement: S1 File — (ZIP) [file pone.0311069.s002.zip › S2_File/2_GO_enrichment/barplot/B_vs_A.up_GO_categorie.pdf]

# Gene Function Classification (GO)

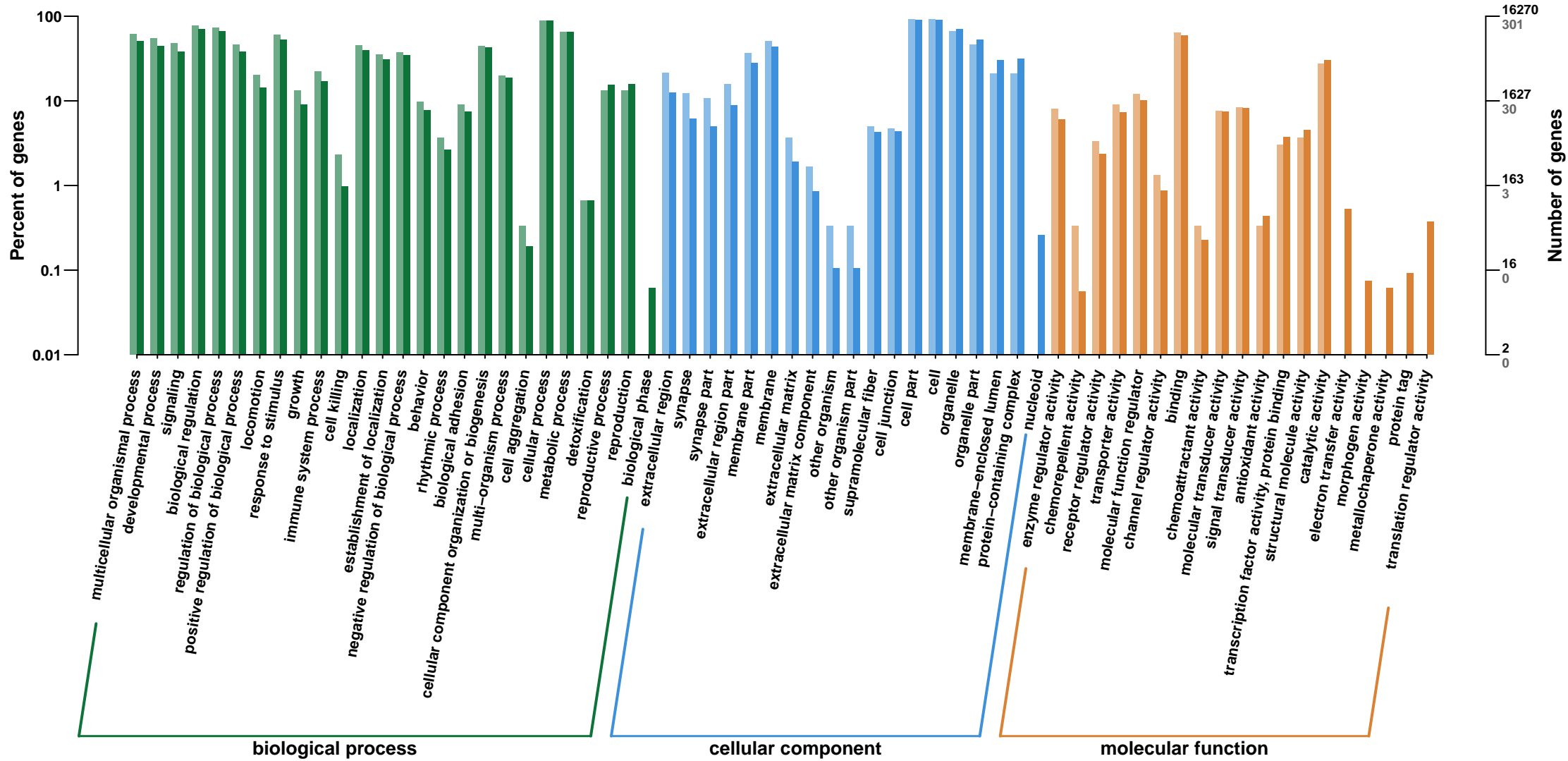

Supplement: S1 File — (ZIP) [file pone.0311069.s002.zip › S2_File/2_GO_enrichment/barplot/union.DE_gene.subcluster1_GO_categorie.pdf]

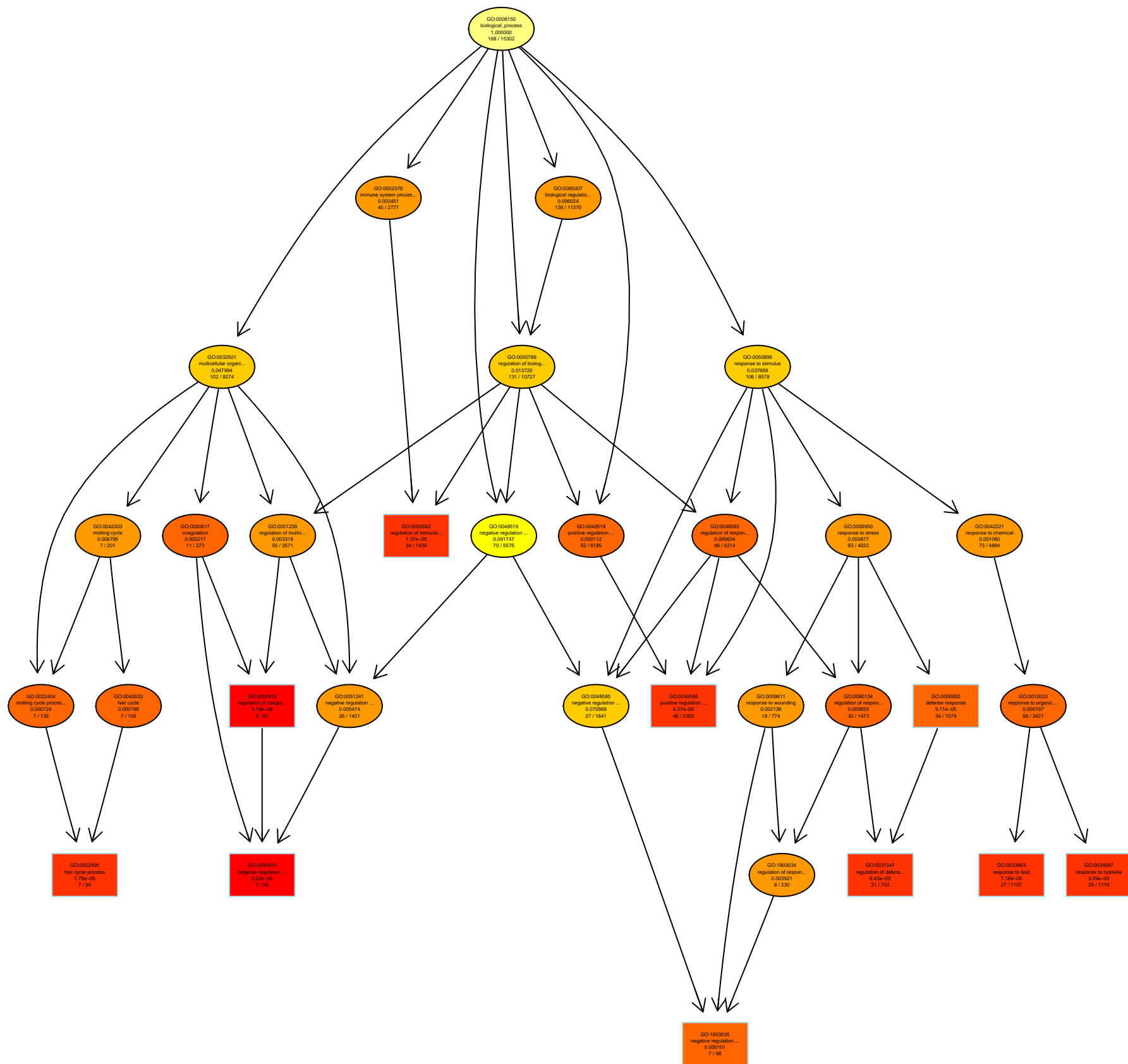

Supplement: S1 File — (ZIP) [file pone.0311069.s002.zip › S2_File/2_GO_enrichment/DAG/B_vs_A.down_BP_DAG.pdf]

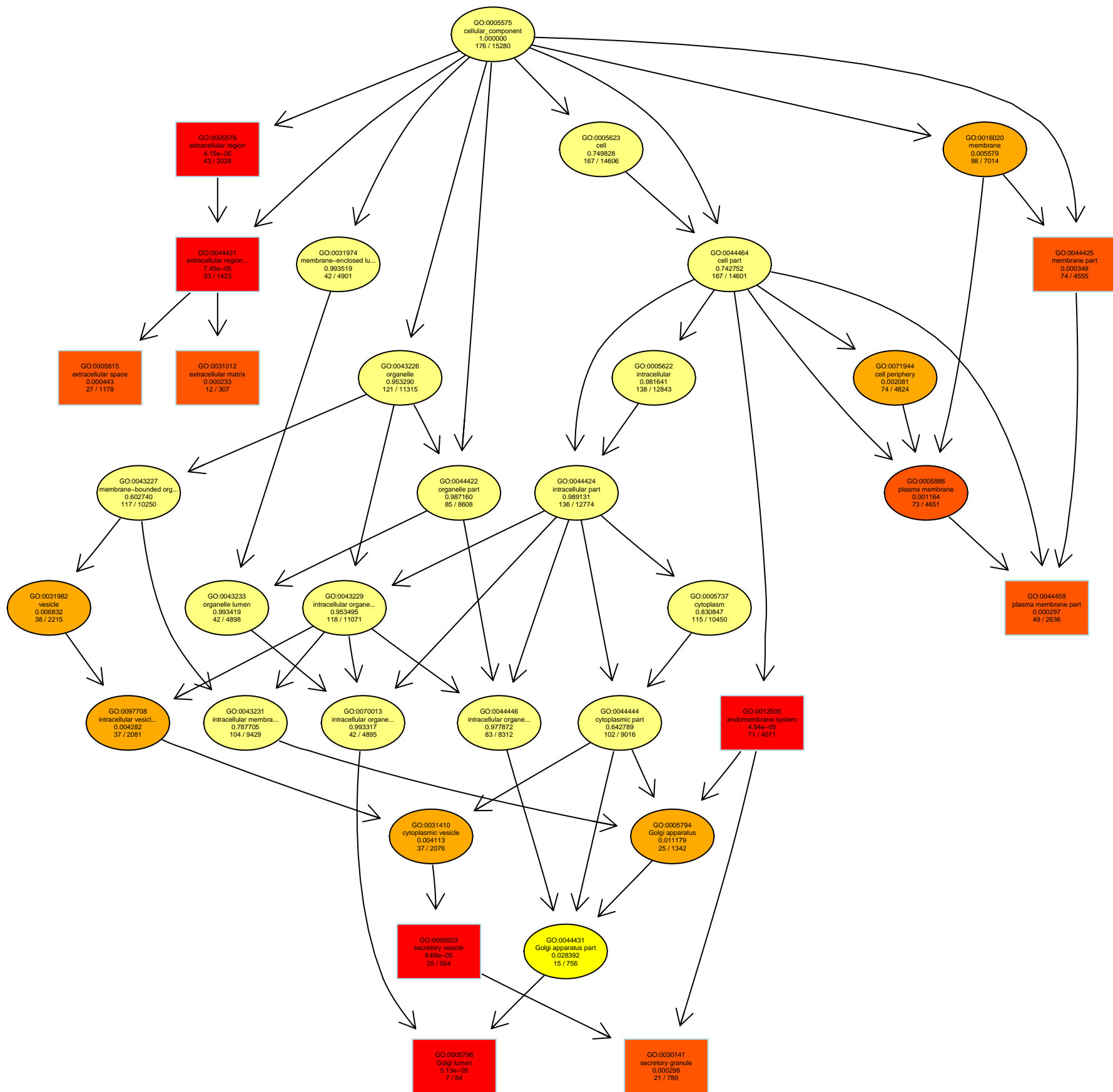

Supplement: S1 File — (ZIP) [file pone.0311069.s002.zip › S2_File/2_GO_enrichment/DAG/B_vs_A.down_CC_DAG.pdf]

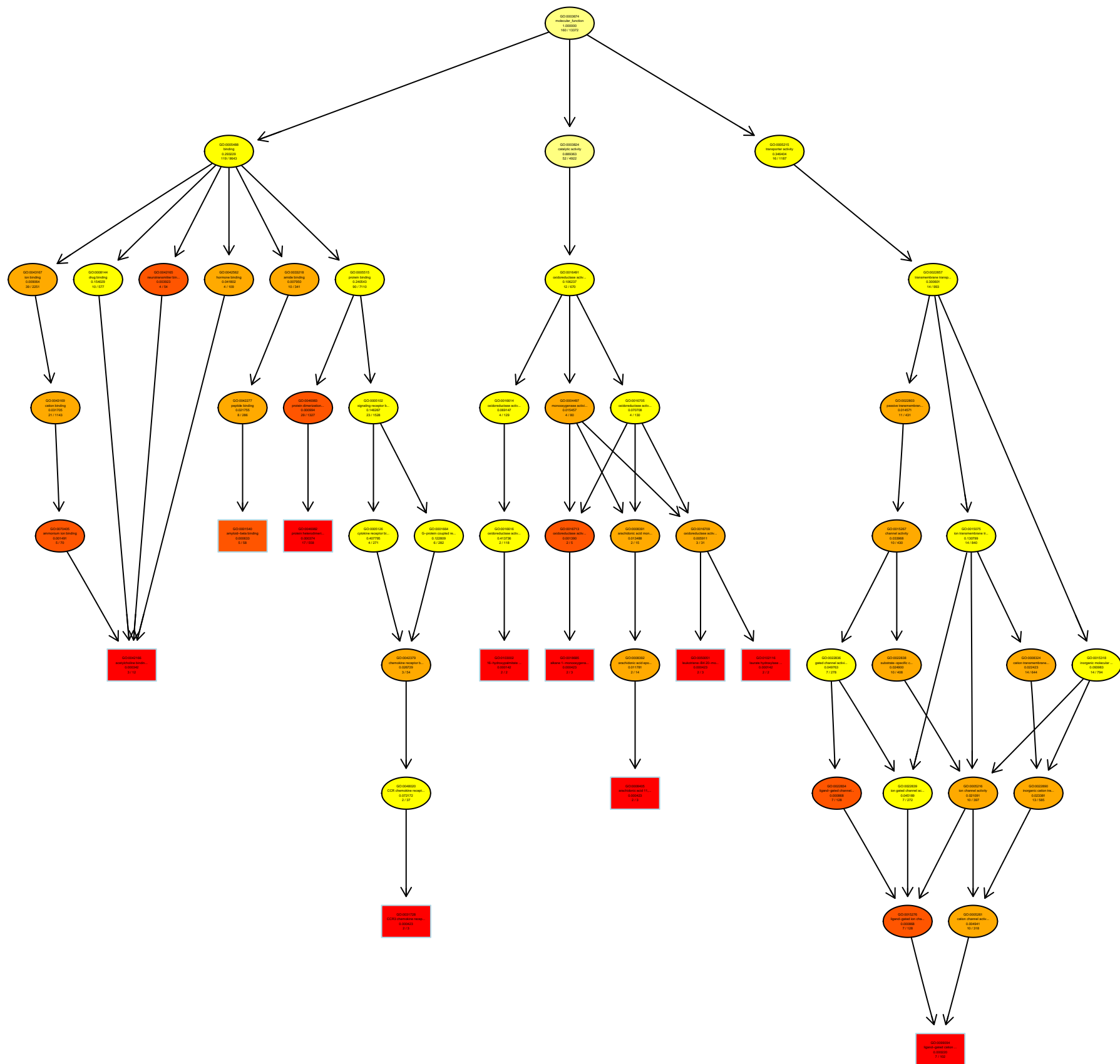

Supplement: S1 File — (ZIP) [file pone.0311069.s002.zip › S2_File/2_GO_enrichment/DAG/B_vs_A.down_MF_DAG.pdf]

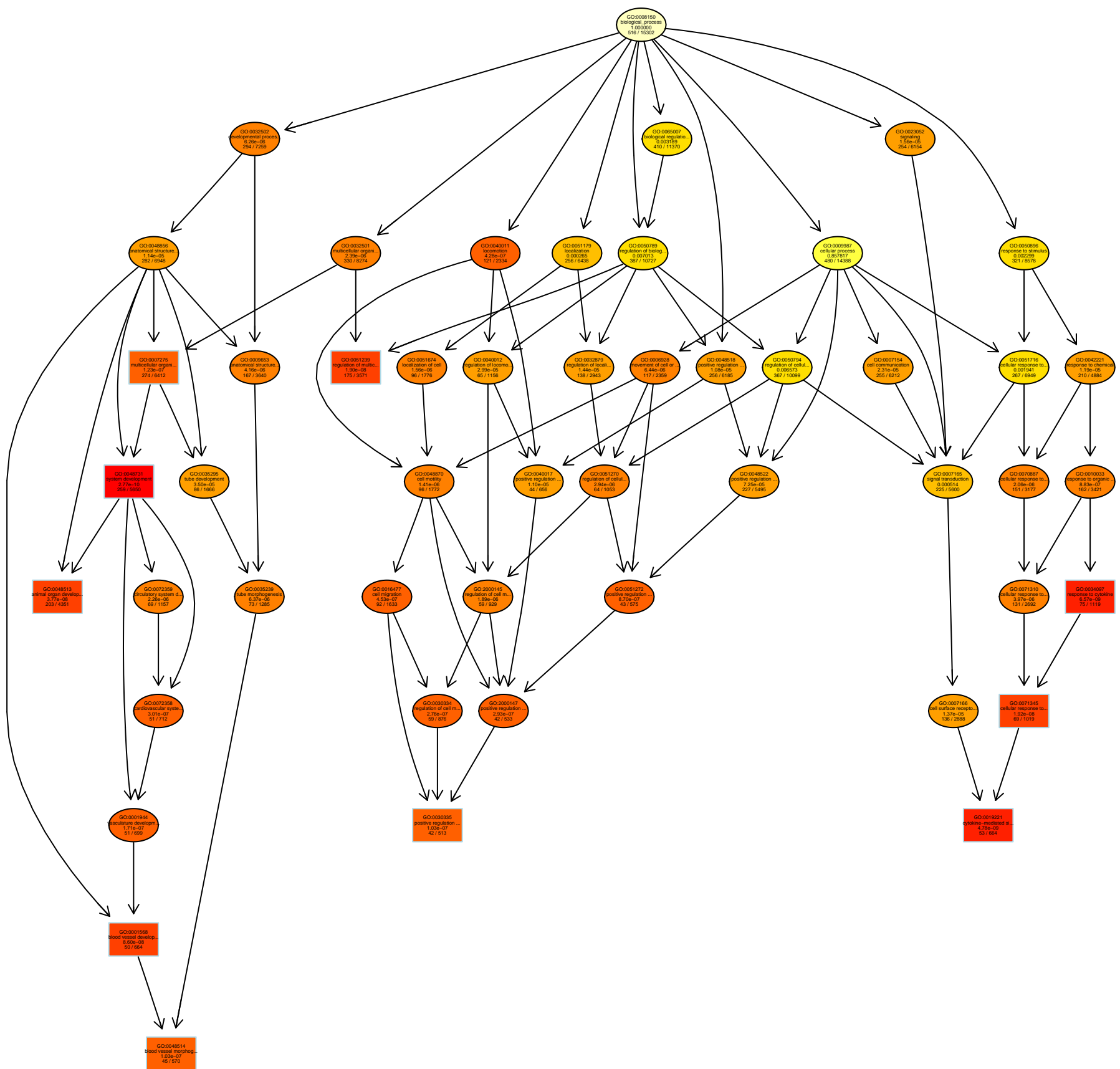

Supplement: S1 File — (ZIP) [file pone.0311069.s002.zip › S2_File/2_GO_enrichment/DAG/B_vs_A.sign_BP_DAG.pdf]

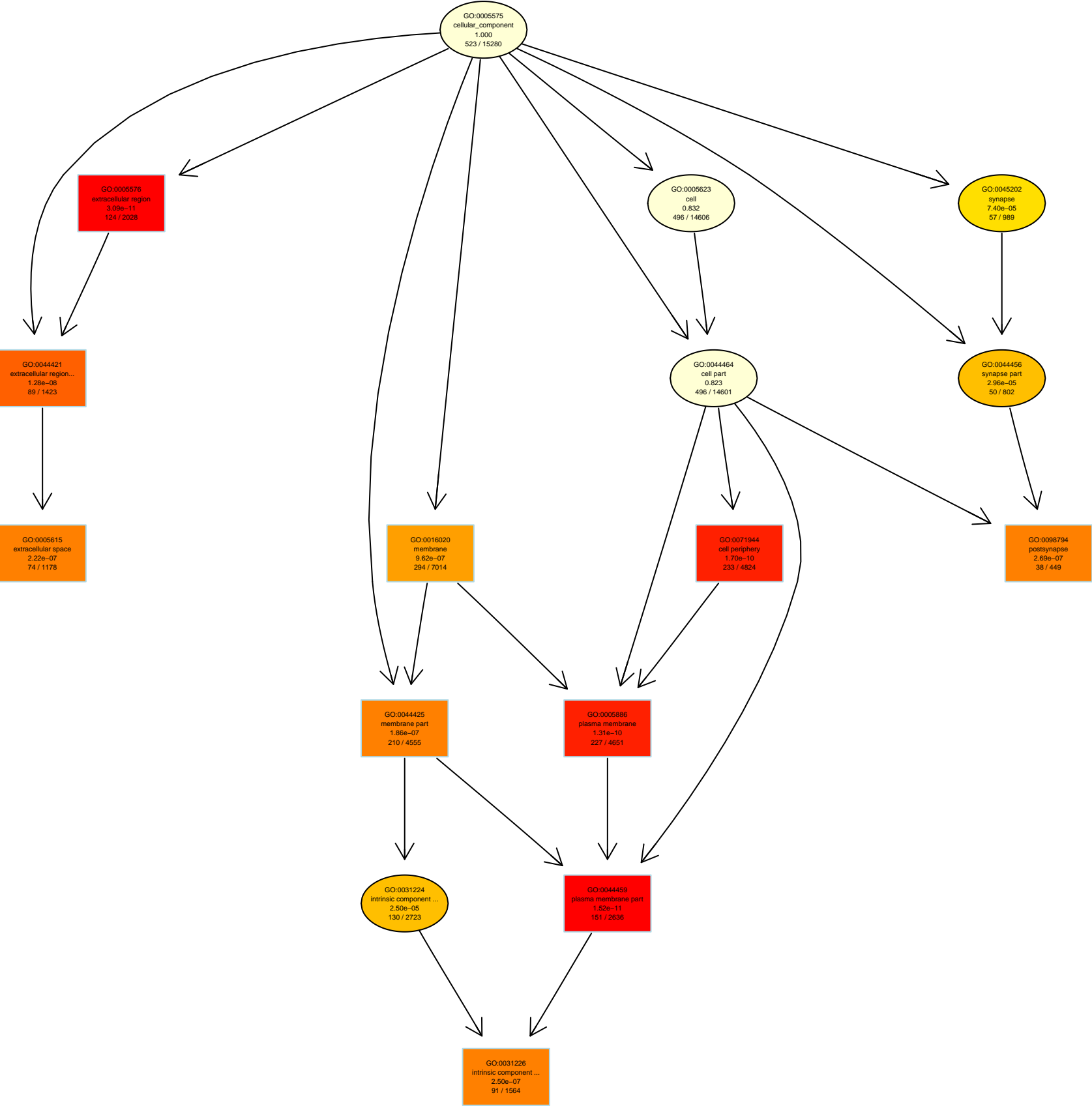

Supplement: S1 File — (ZIP) [file pone.0311069.s002.zip › S2_File/2_GO_enrichment/DAG/B_vs_A.sign_CC_DAG.pdf]

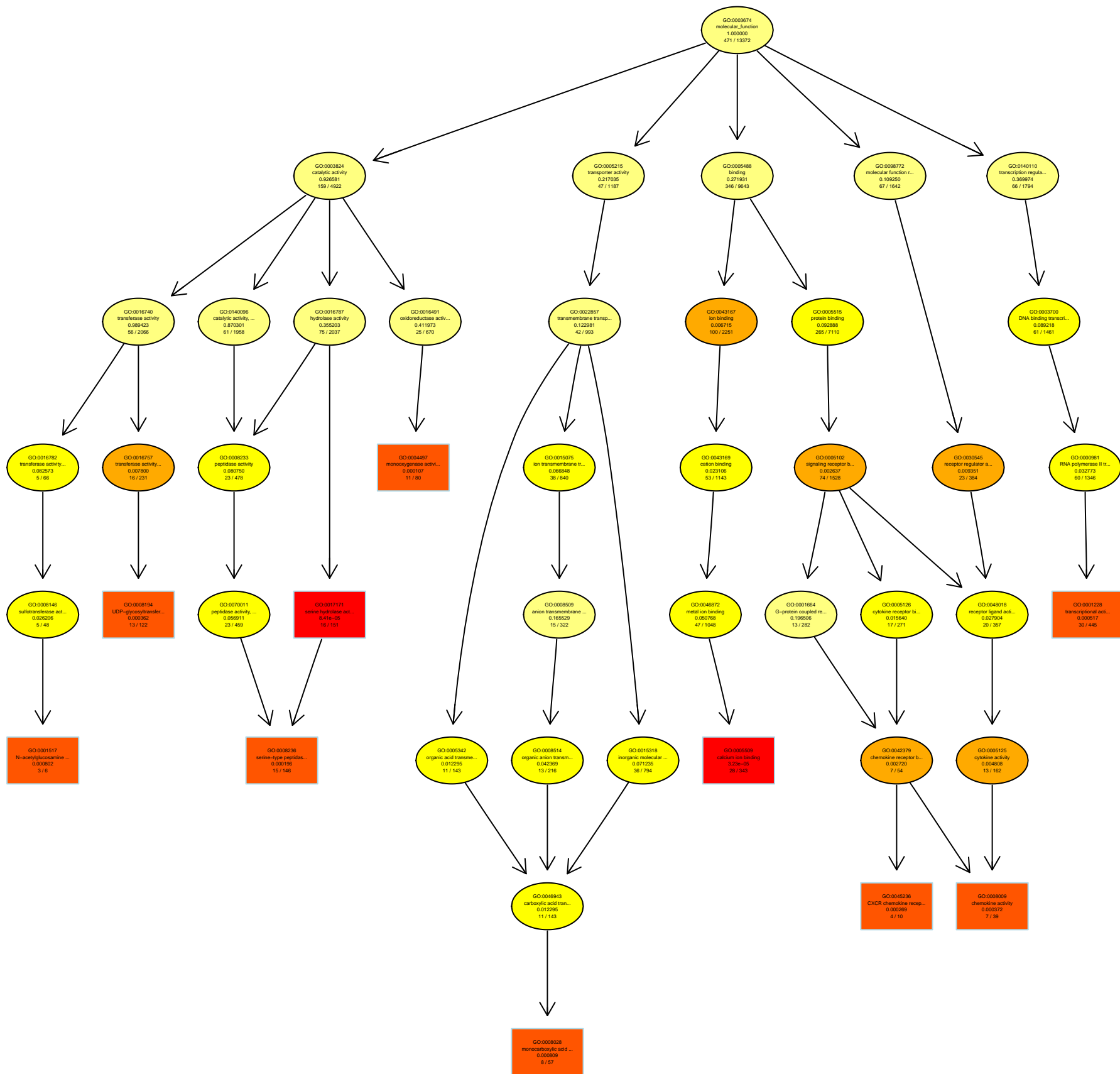

Supplement: S1 File — (ZIP) [file pone.0311069.s002.zip › S2_File/2_GO_enrichment/DAG/B_vs_A.sign_MF_DAG.pdf]

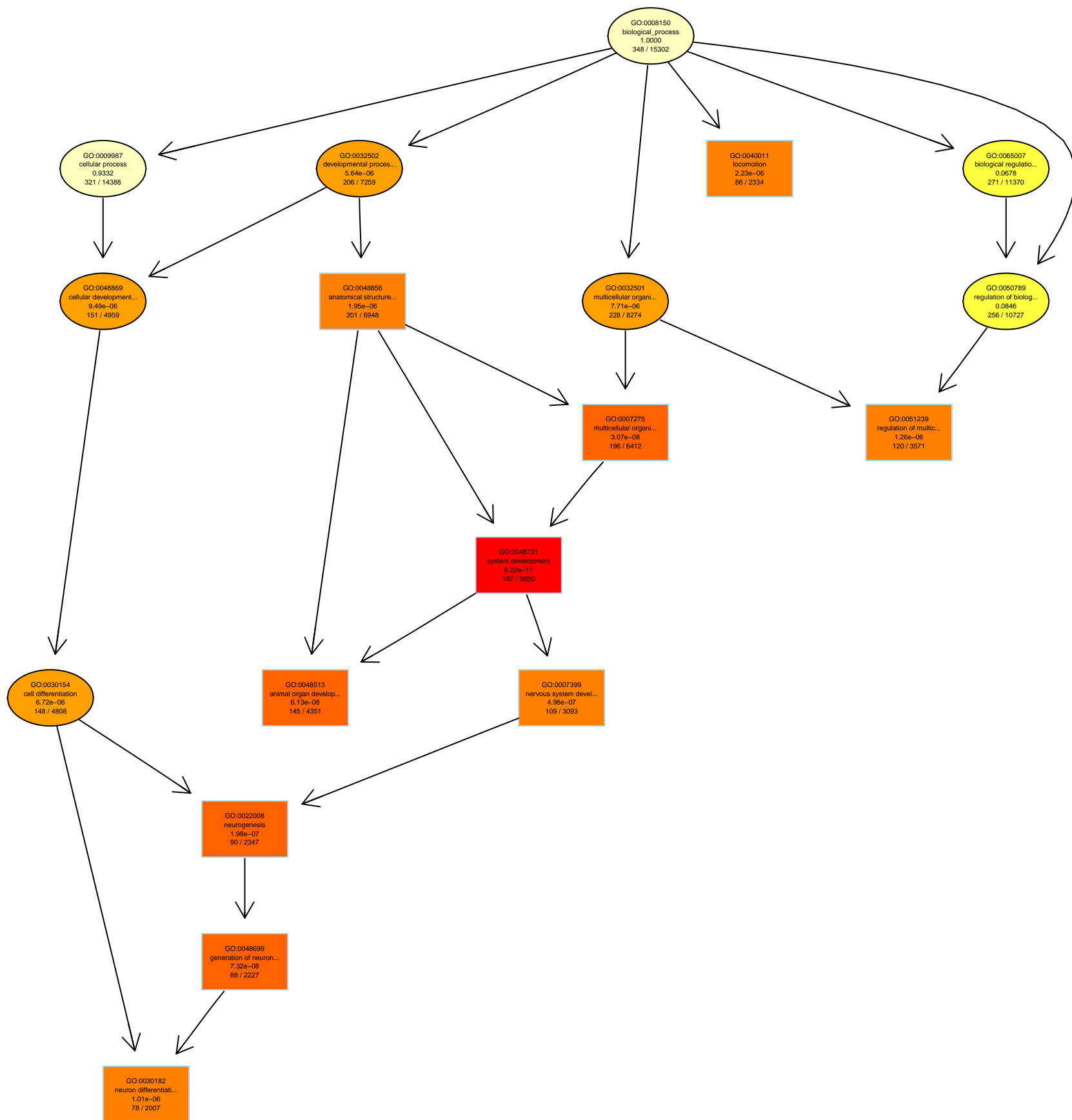

Supplement: S1 File — (ZIP) [file pone.0311069.s002.zip › S2_File/2_GO_enrichment/DAG/B_vs_A.up_BP_DAG.pdf]

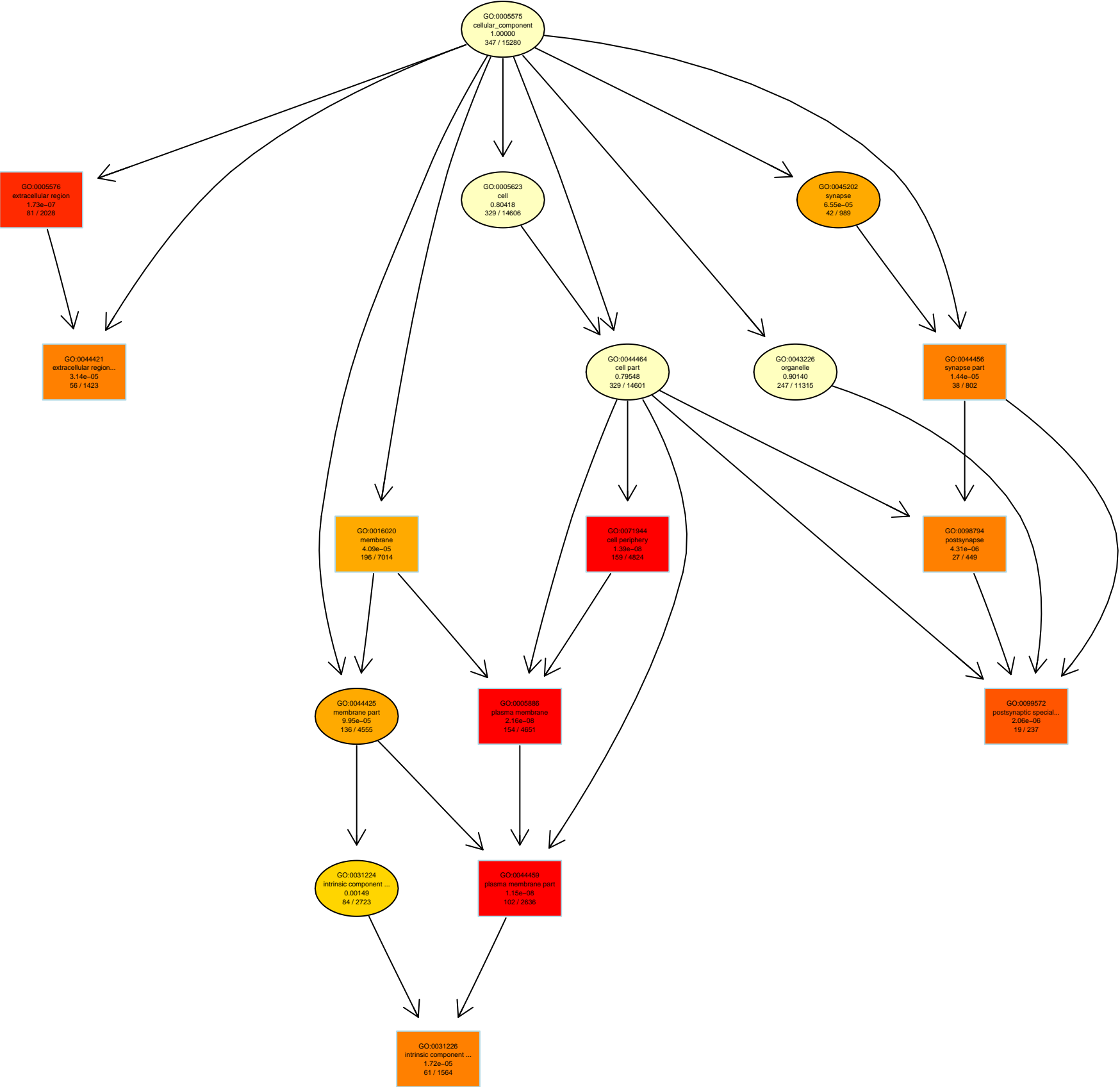

Supplement: S1 File — (ZIP) [file pone.0311069.s002.zip › S2_File/2_GO_enrichment/DAG/B_vs_A.up_CC_DAG.pdf]

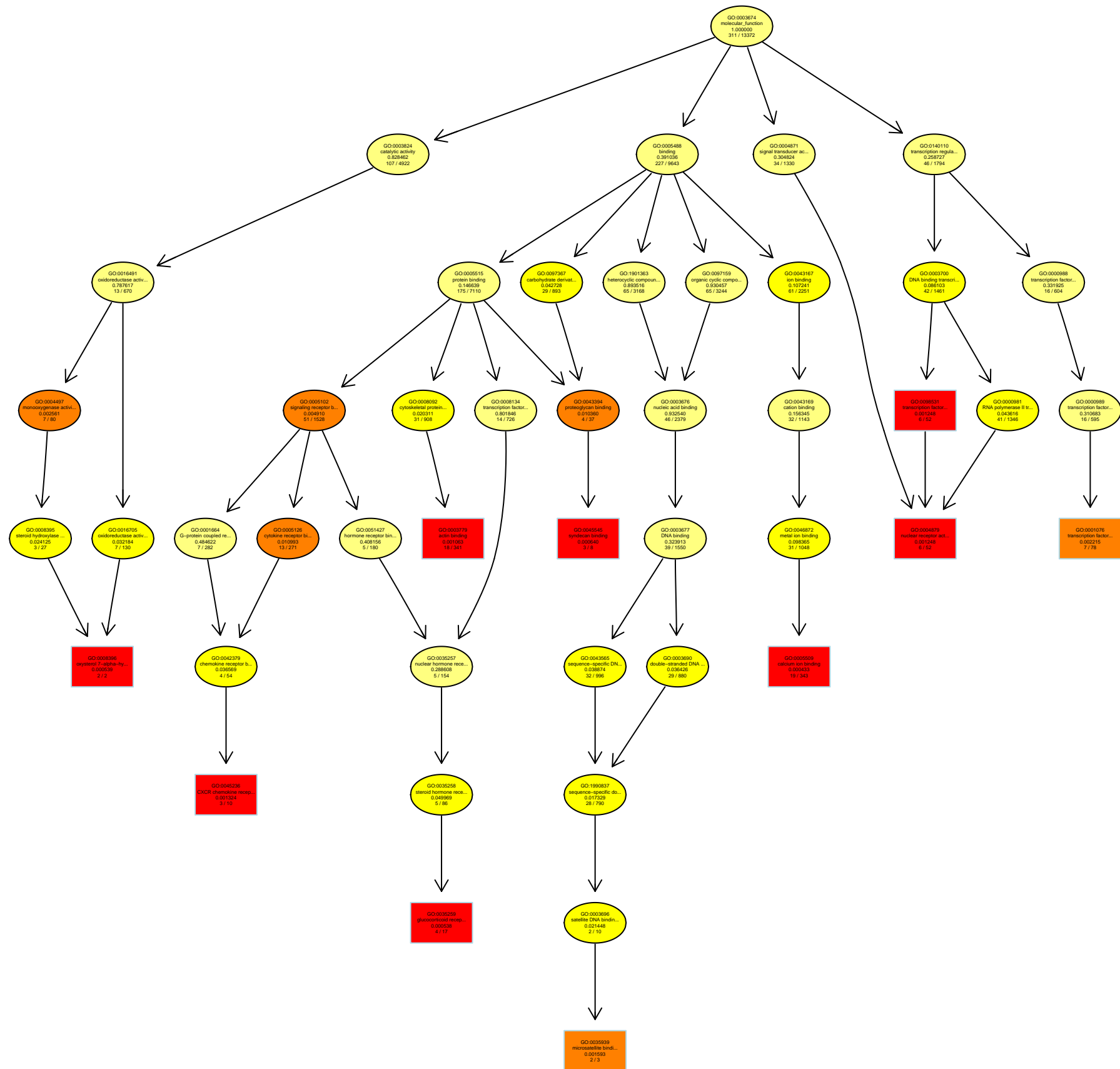

Supplement: S1 File — (ZIP) [file pone.0311069.s002.zip › S2_File/2_GO_enrichment/DAG/B_vs_A.up_MF_DAG.pdf]

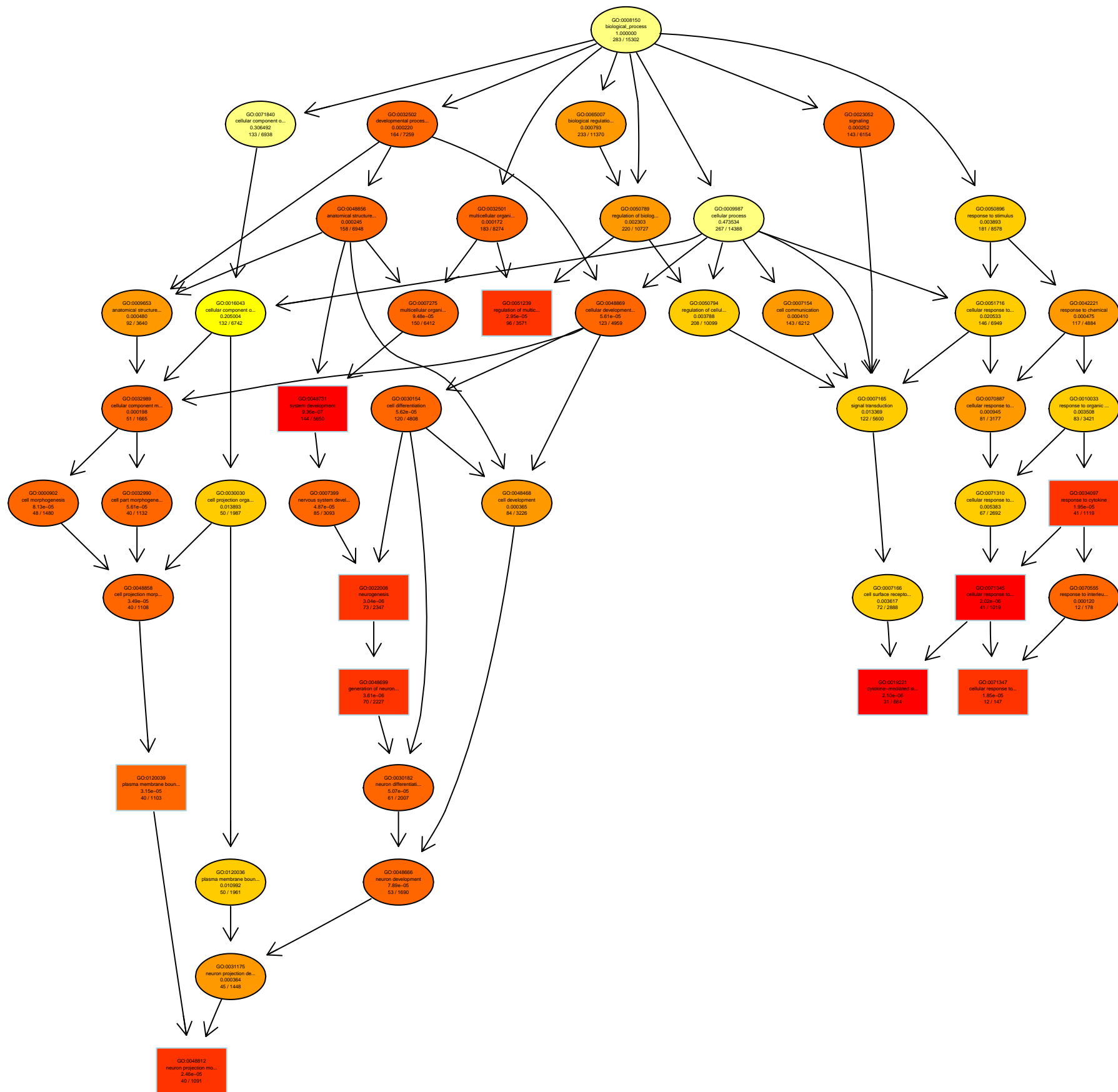

Supplement: S1 File — (ZIP) [file pone.0311069.s002.zip › S2_File/2_GO_enrichment/DAG/union.DE_gene.subcluster1_BP_DAG.pdf]

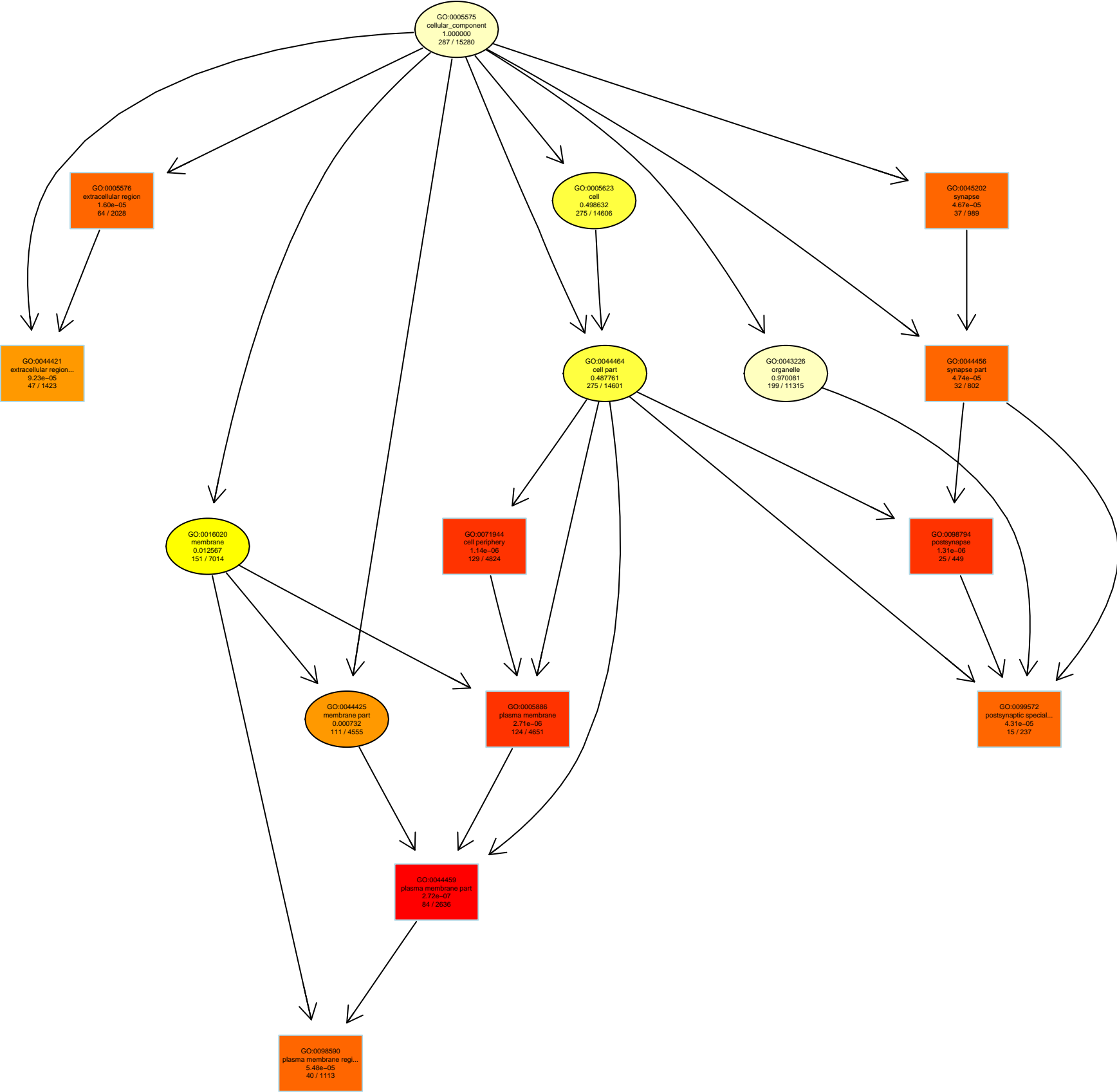

Supplement: S1 File — (ZIP) [file pone.0311069.s002.zip › S2_File/2_GO_enrichment/DAG/union.DE_gene.subcluster1_CC_DAG.pdf]

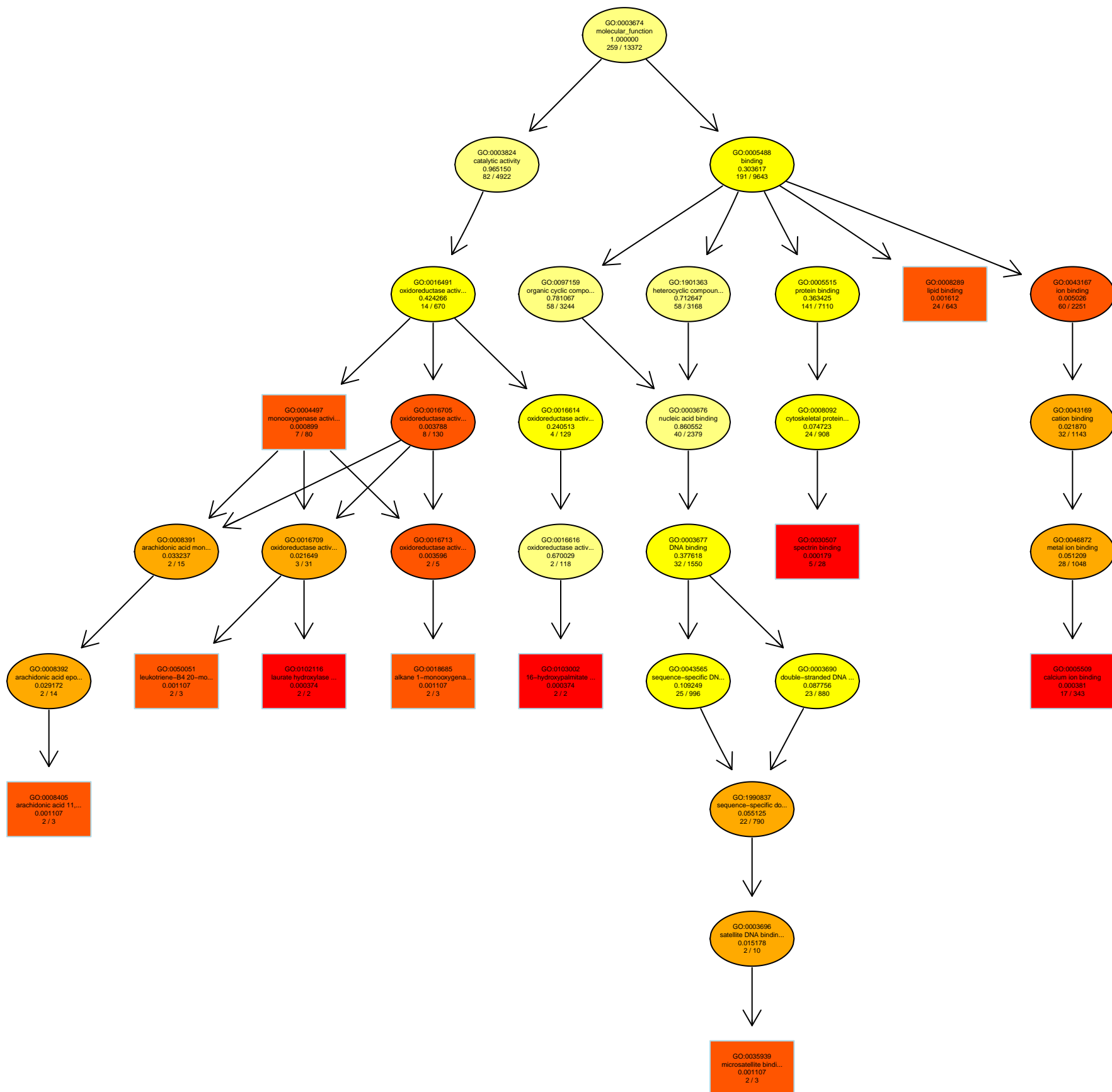

Supplement: S1 File — (ZIP) [file pone.0311069.s002.zip › S2_File/2_GO_enrichment/DAG/union.DE_gene.subcluster1_MF_DAG.pdf]

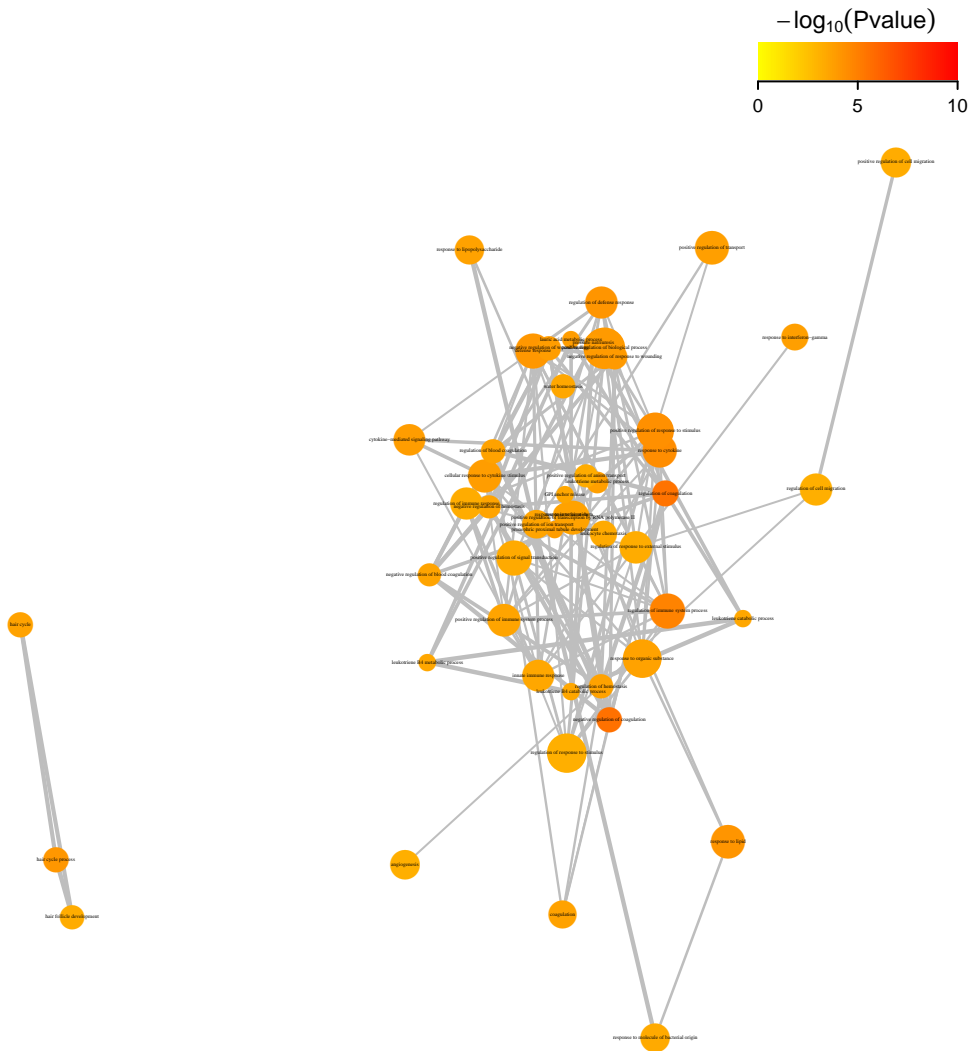

Supplement: S1 File — (ZIP) [file pone.0311069.s002.zip › S2_File/2_GO_enrichment/enrichmap/B_vs_A.down_BP_enrichment_map.pdf]

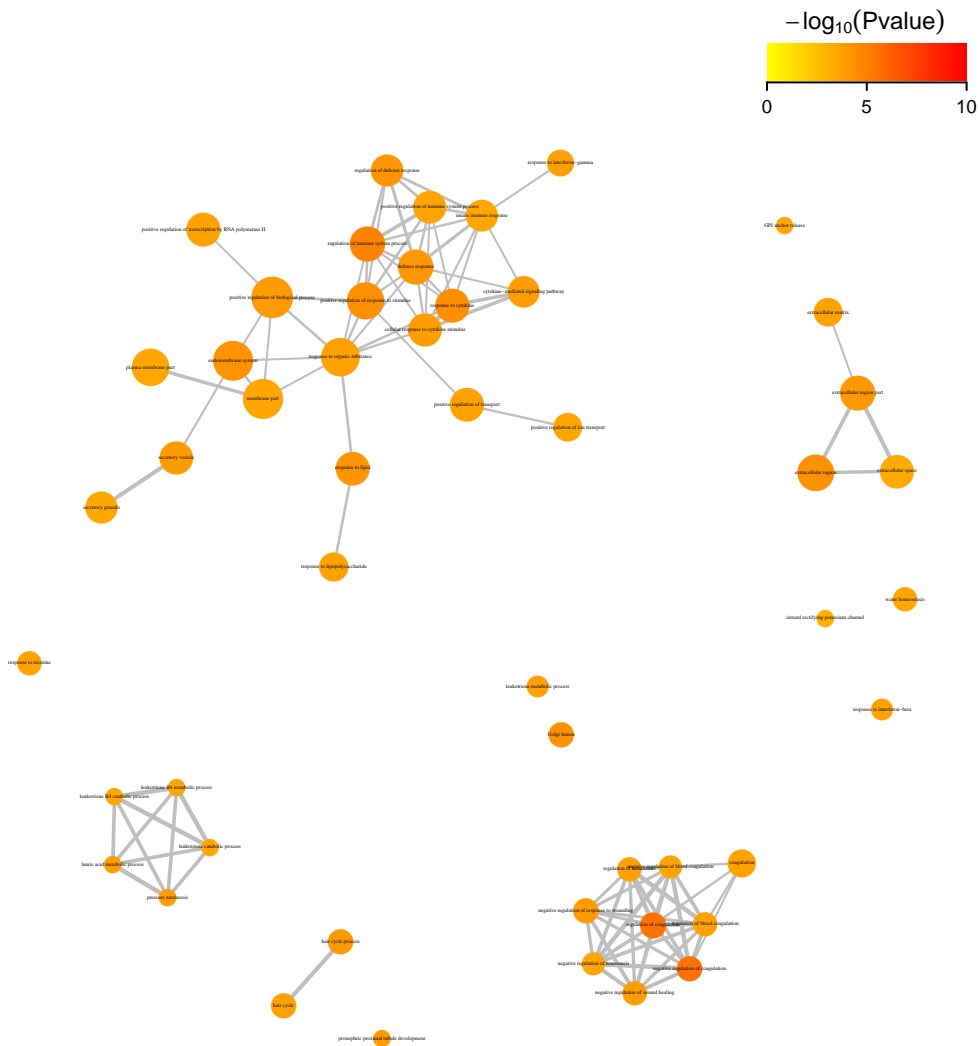

Supplement: S1 File — (ZIP) [file pone.0311069.s002.zip › S2_File/2_GO_enrichment/enrichmap/B_vs_A.down_GO_enrichment_map.pdf]

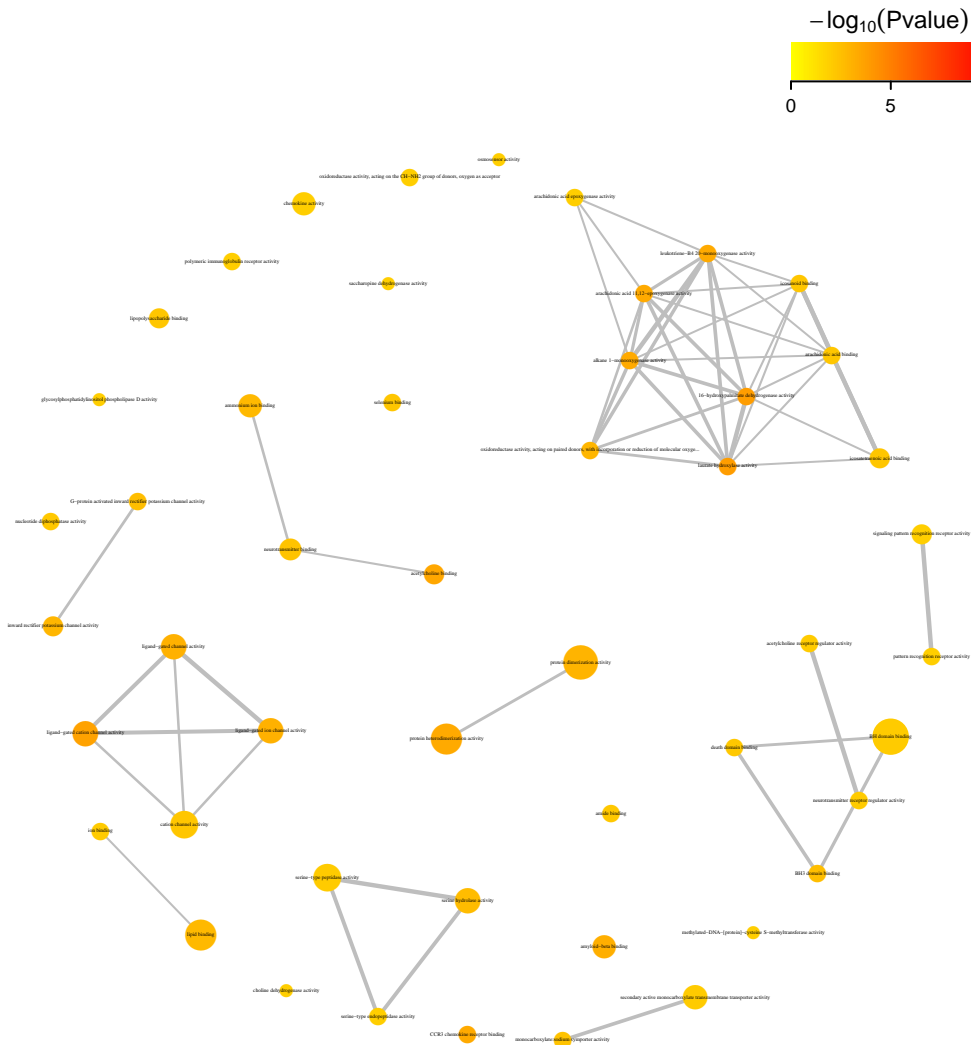

Supplement: S1 File — (ZIP) [file pone.0311069.s002.zip › S2_File/2_GO_enrichment/enrichmap/B_vs_A.down_MF_enrichment_map.pdf]

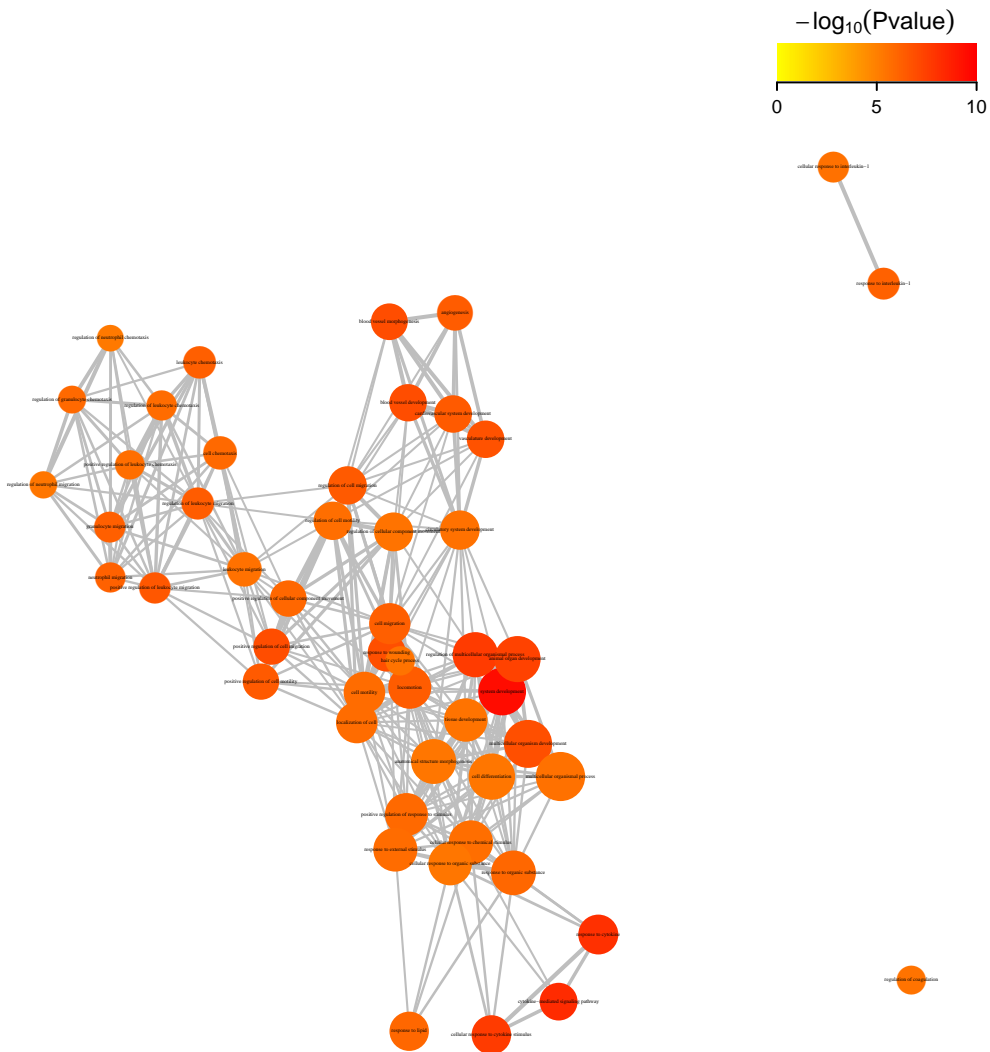

Supplement: S1 File — (ZIP) [file pone.0311069.s002.zip › S2_File/2_GO_enrichment/enrichmap/B_vs_A.sign_BP_enrichment_map.pdf]

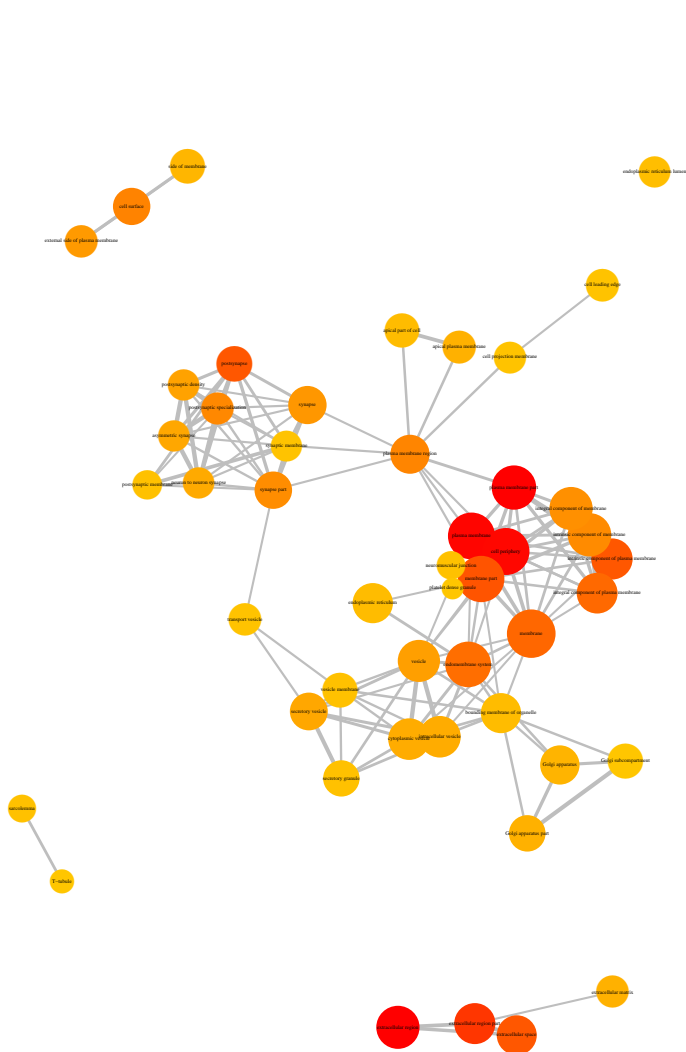

Supplement: S1 File — (ZIP) [file pone.0311069.s002.zip › S2_File/2_GO_enrichment/enrichmap/B_vs_A.sign_CC_enrichment_map.pdf]

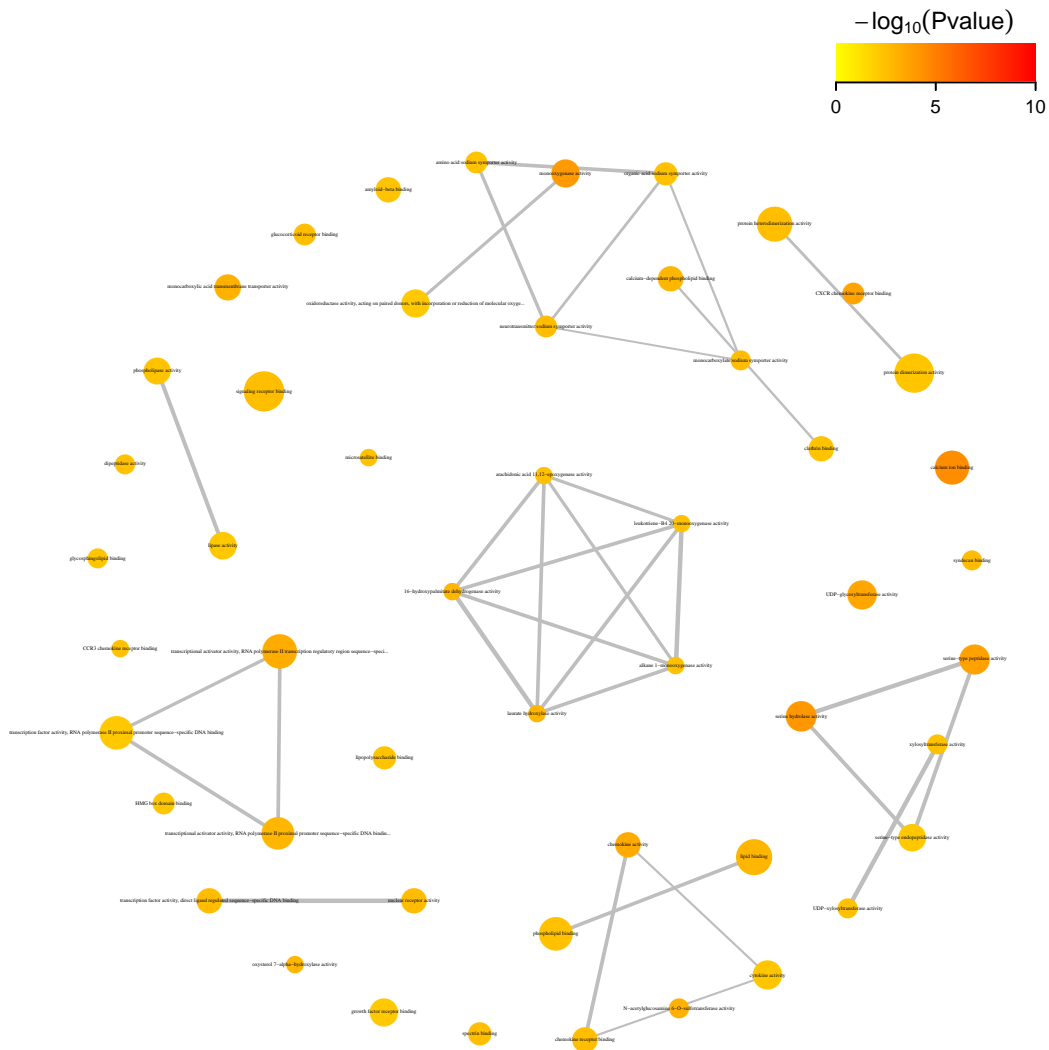

Supplement: S1 File — (ZIP) [file pone.0311069.s002.zip › S2_File/2_GO_enrichment/enrichmap/B_vs_A.sign_MF_enrichment_map.pdf]

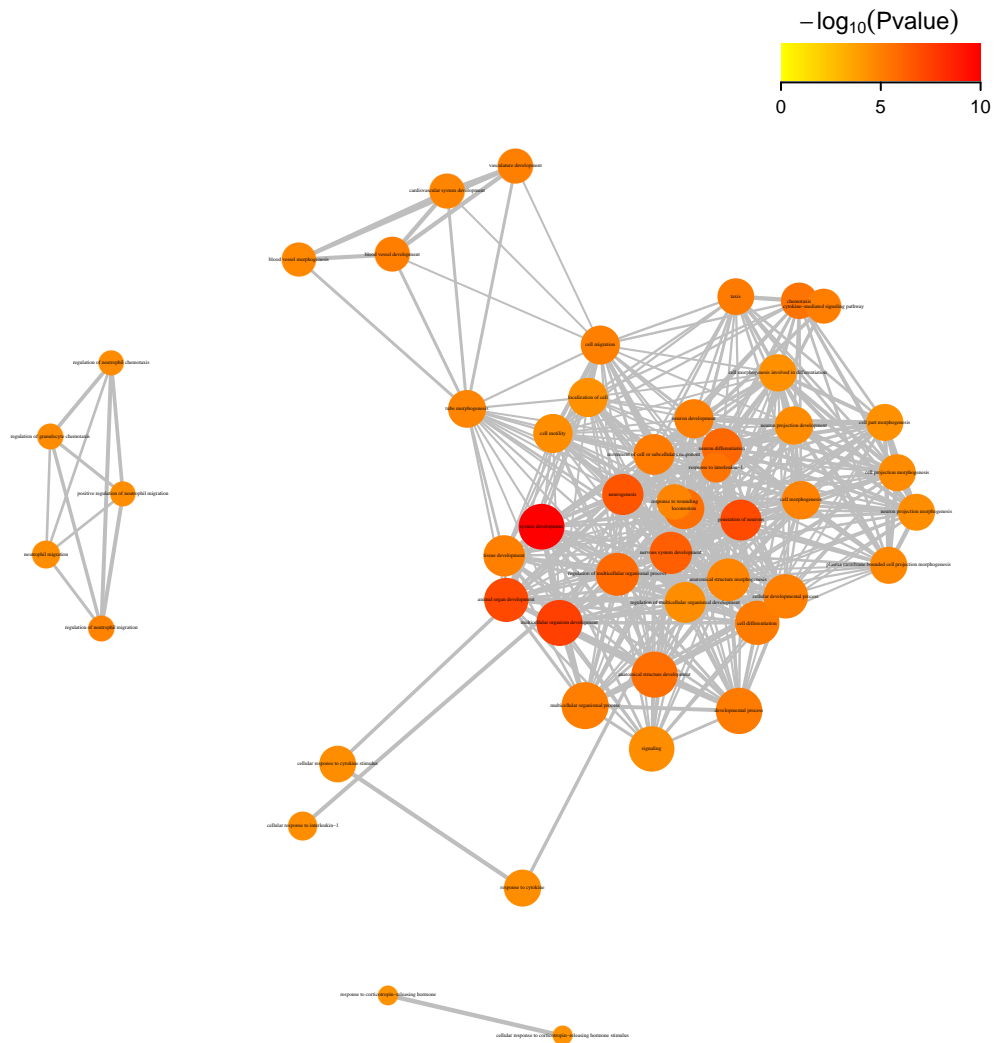

Supplement: S1 File — (ZIP) [file pone.0311069.s002.zip › S2_File/2_GO_enrichment/enrichmap/B_vs_A.up_BP_enrichment_map.pdf]

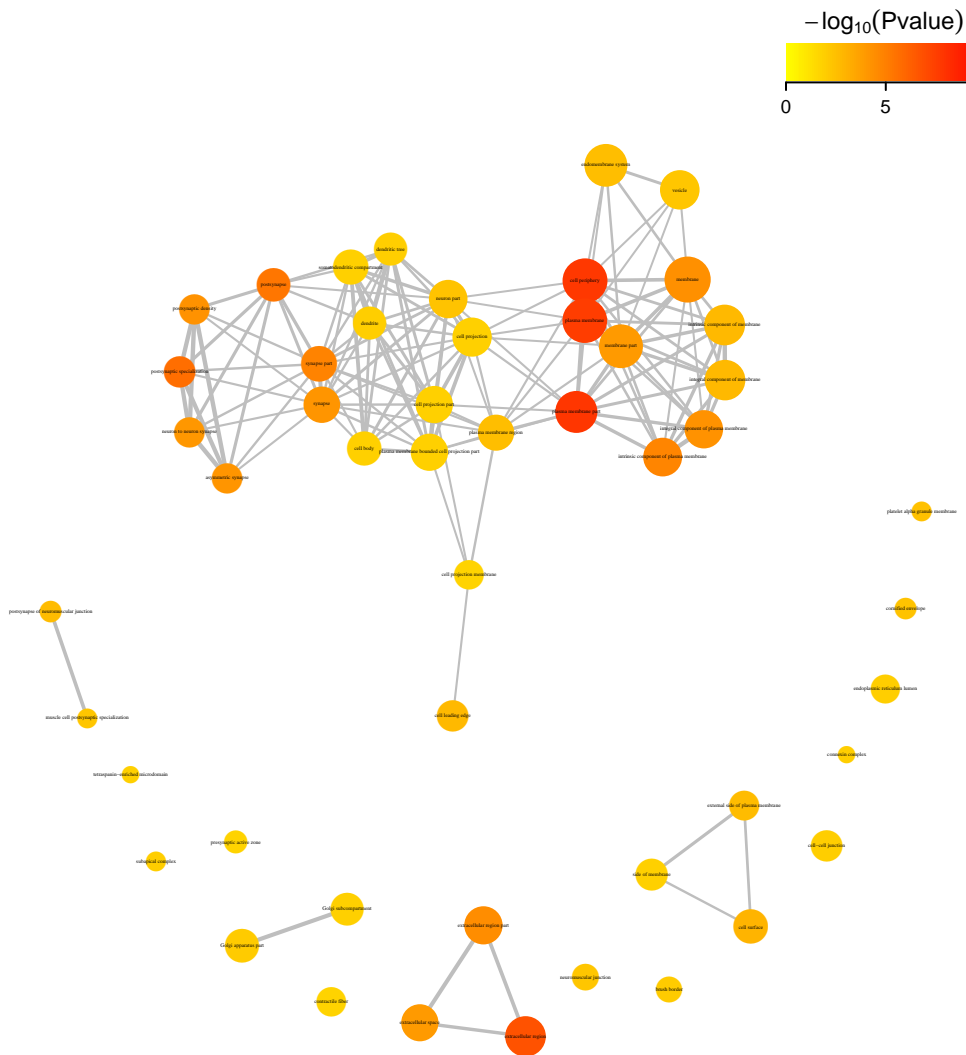

Supplement: S1 File — (ZIP) [file pone.0311069.s002.zip › S2_File/2_GO_enrichment/enrichmap/B_vs_A.up_CC_enrichment_map.pdf]

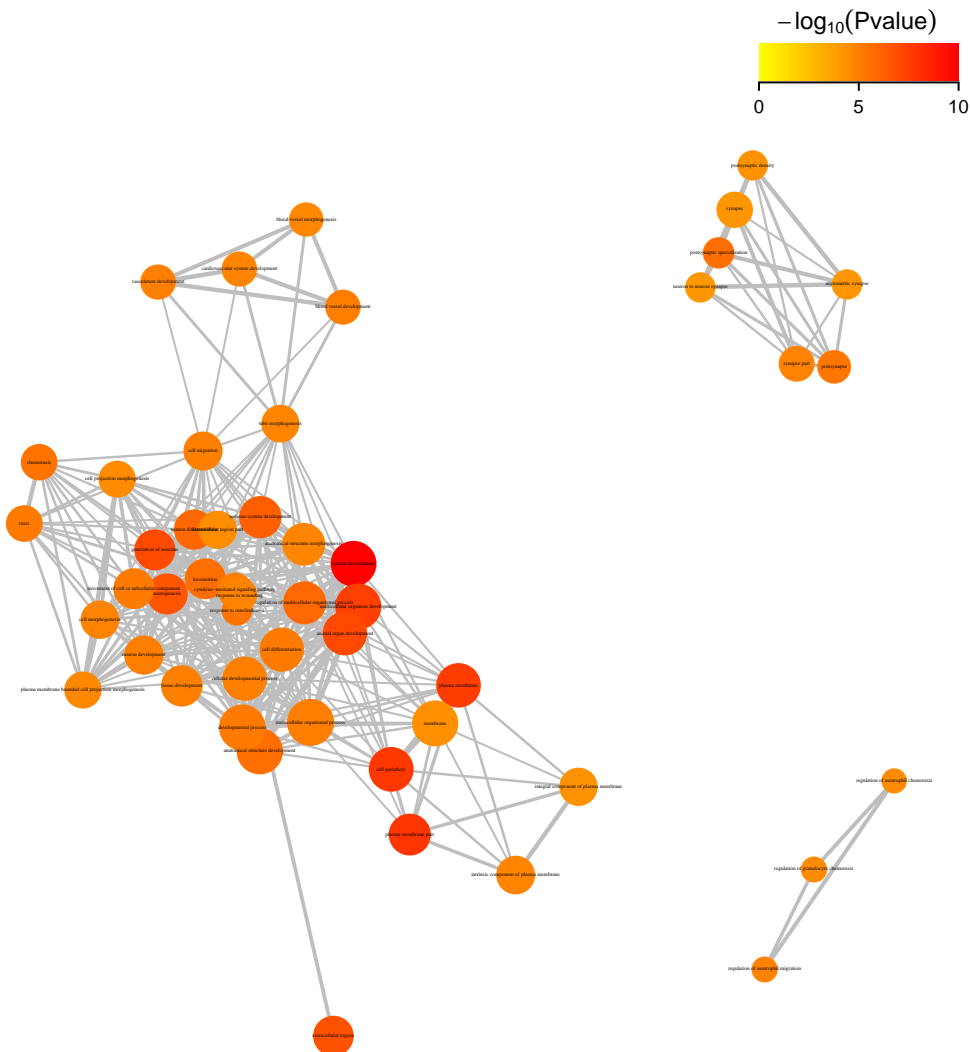

Supplement: S1 File — (ZIP) [file pone.0311069.s002.zip › S2_File/2_GO_enrichment/enrichmap/B_vs_A.up_GO_enrichment_map.pdf]

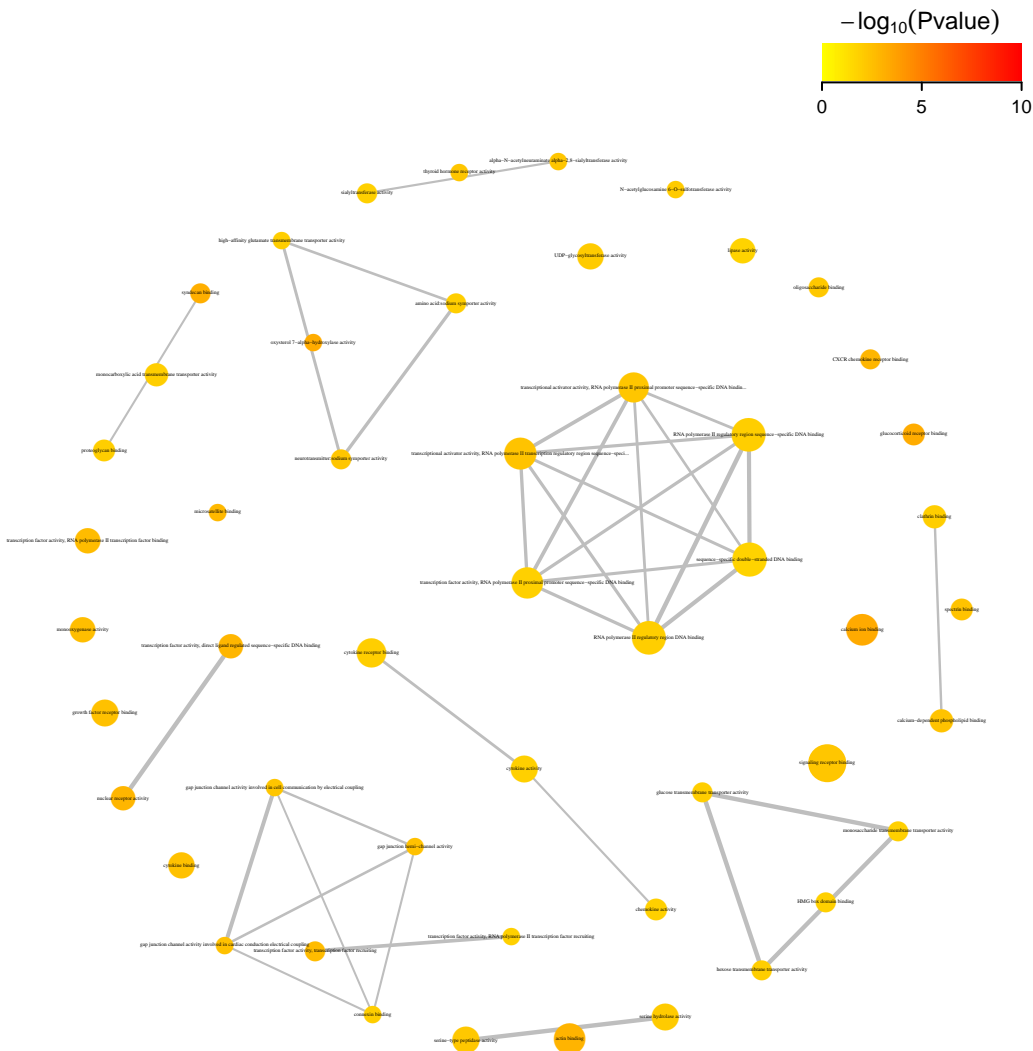

Supplement: S1 File — (ZIP) [file pone.0311069.s002.zip › S2_File/2_GO_enrichment/enrichmap/B_vs_A.up_MF_enrichment_map.pdf]

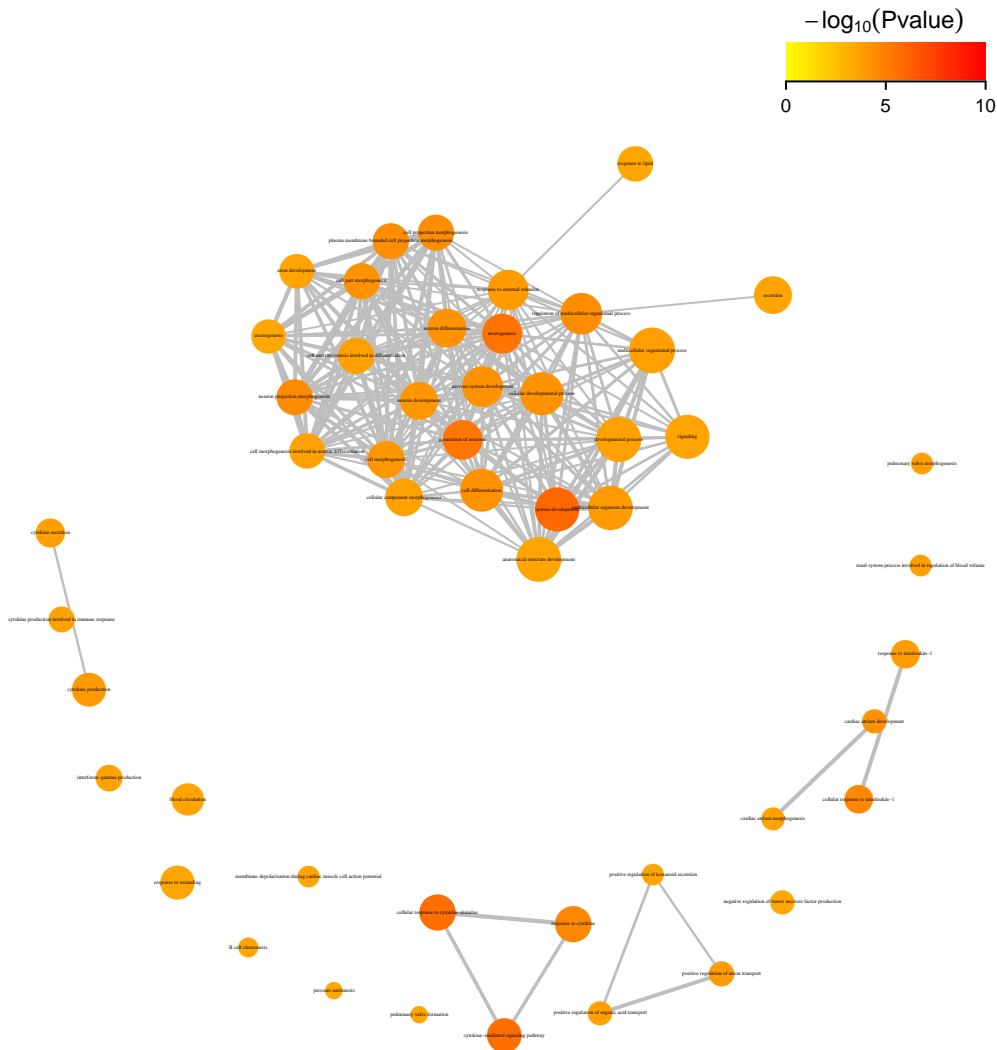

Supplement: S1 File — (ZIP) [file pone.0311069.s002.zip › S2_File/2_GO_enrichment/enrichmap/union.DE_gene.subcluster1_BP_enrichment_map.pdf]

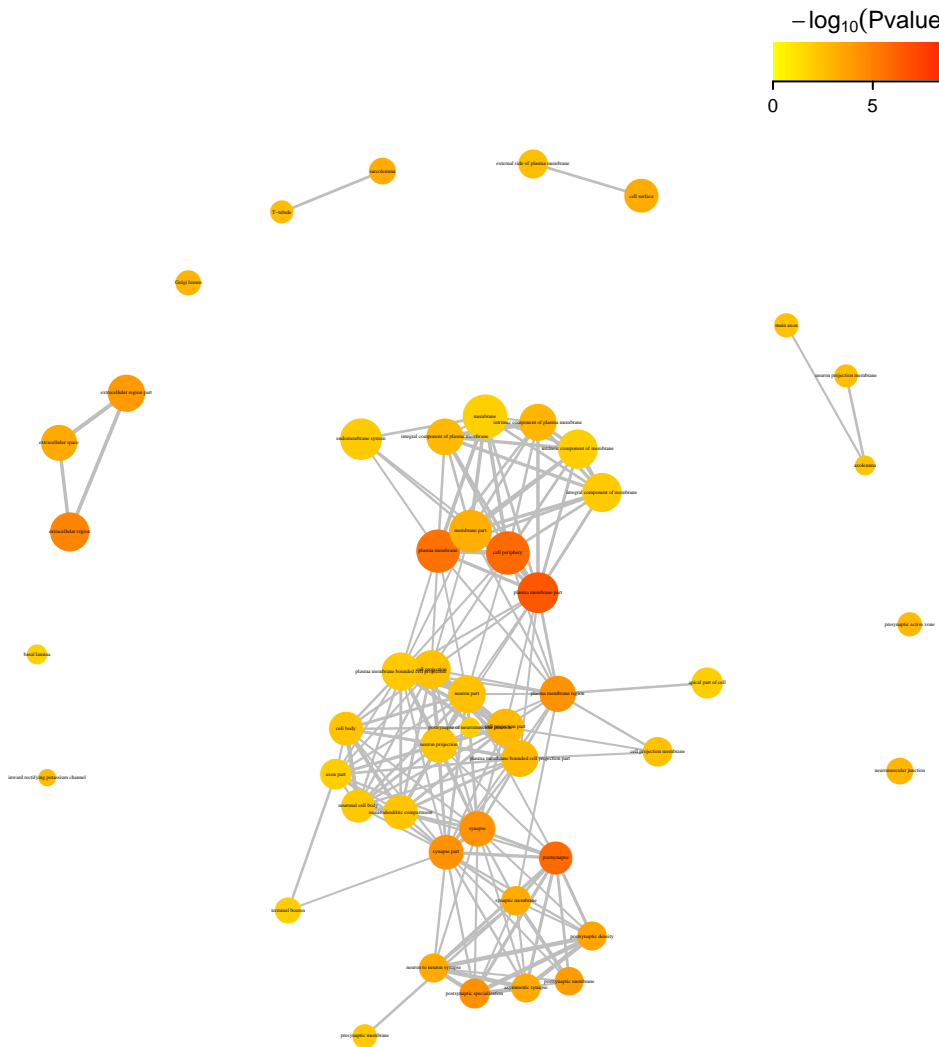

Supplement: S1 File — (ZIP) [file pone.0311069.s002.zip › S2_File/2_GO_enrichment/enrichmap/union.DE_gene.subcluster1_CC_enrichment_map.pdf]

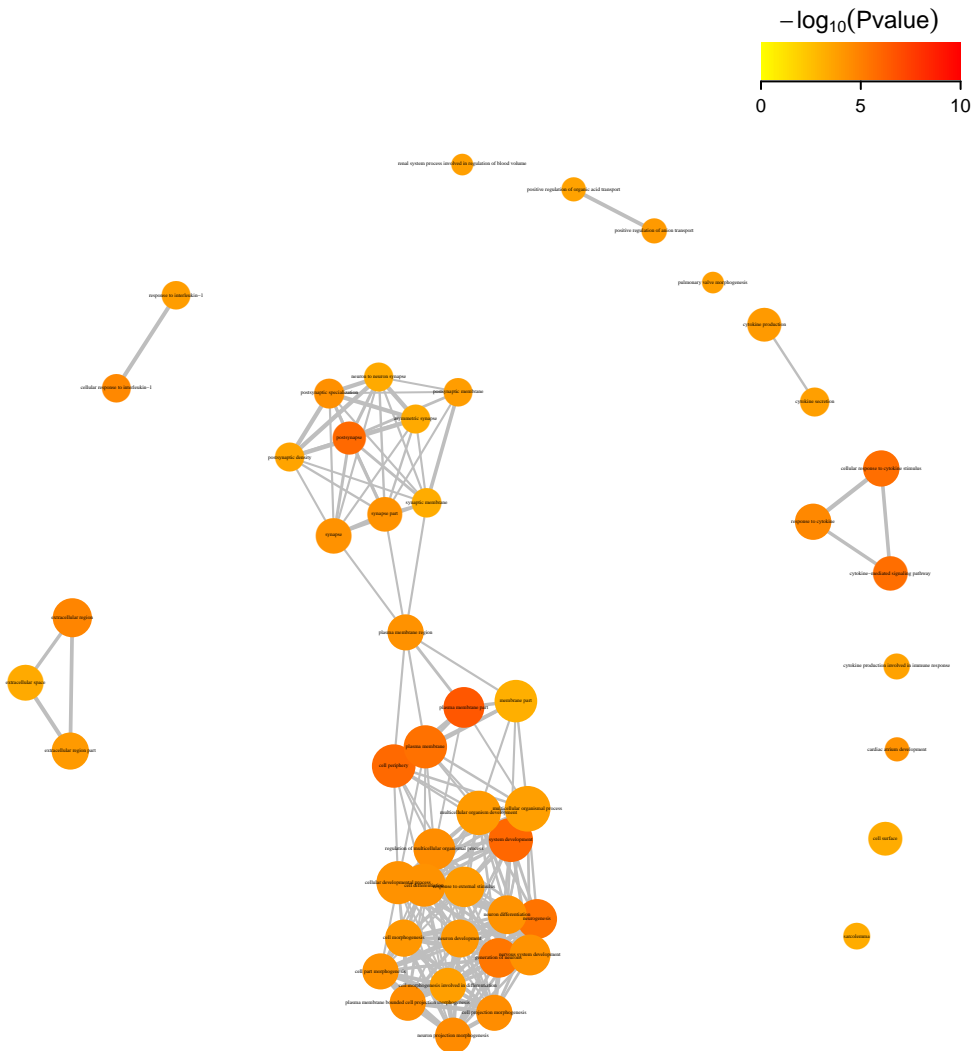

Supplement: S1 File — (ZIP) [file pone.0311069.s002.zip › S2_File/2_GO_enrichment/enrichmap/union.DE_gene.subcluster1_GO_enrichment_map.pdf]

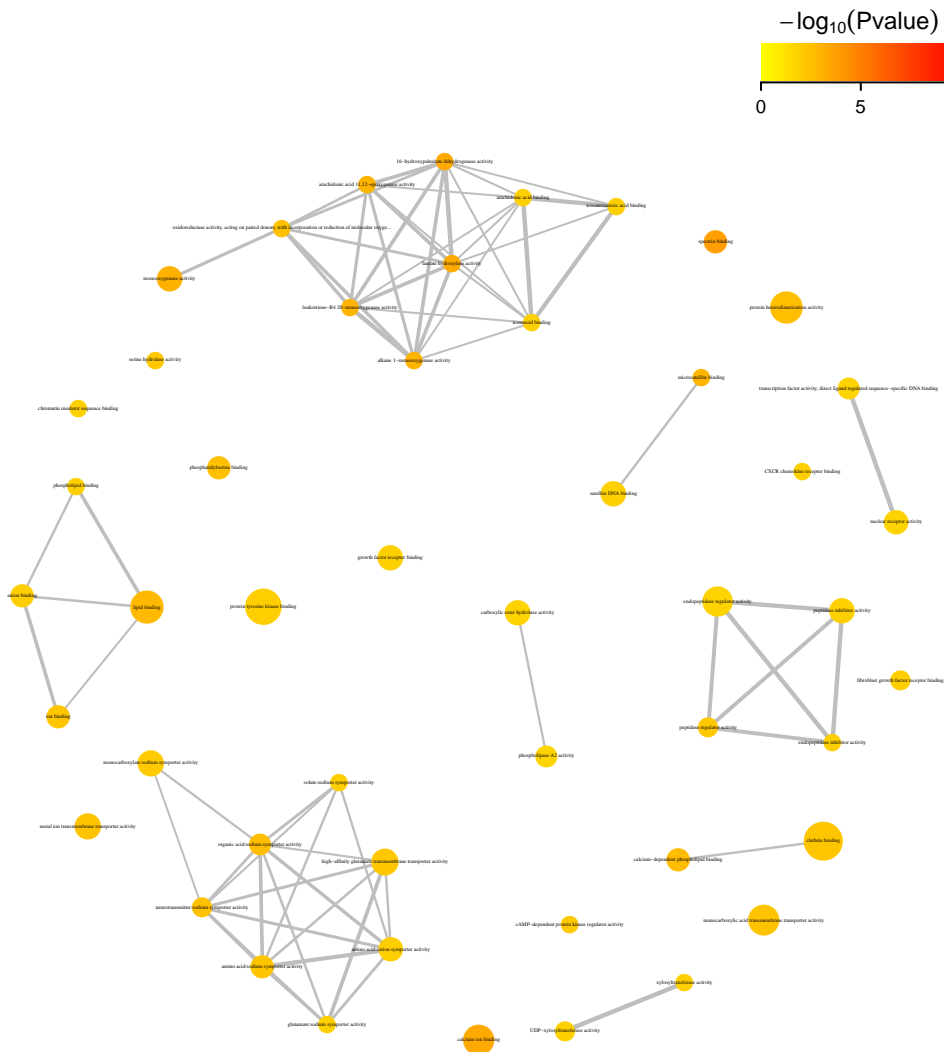

Supplement: S1 File — (ZIP) [file pone.0311069.s002.zip › S2_File/2_GO_enrichment/enrichmap/union.DE_gene.subcluster1_MF_enrichment_map.pdf]

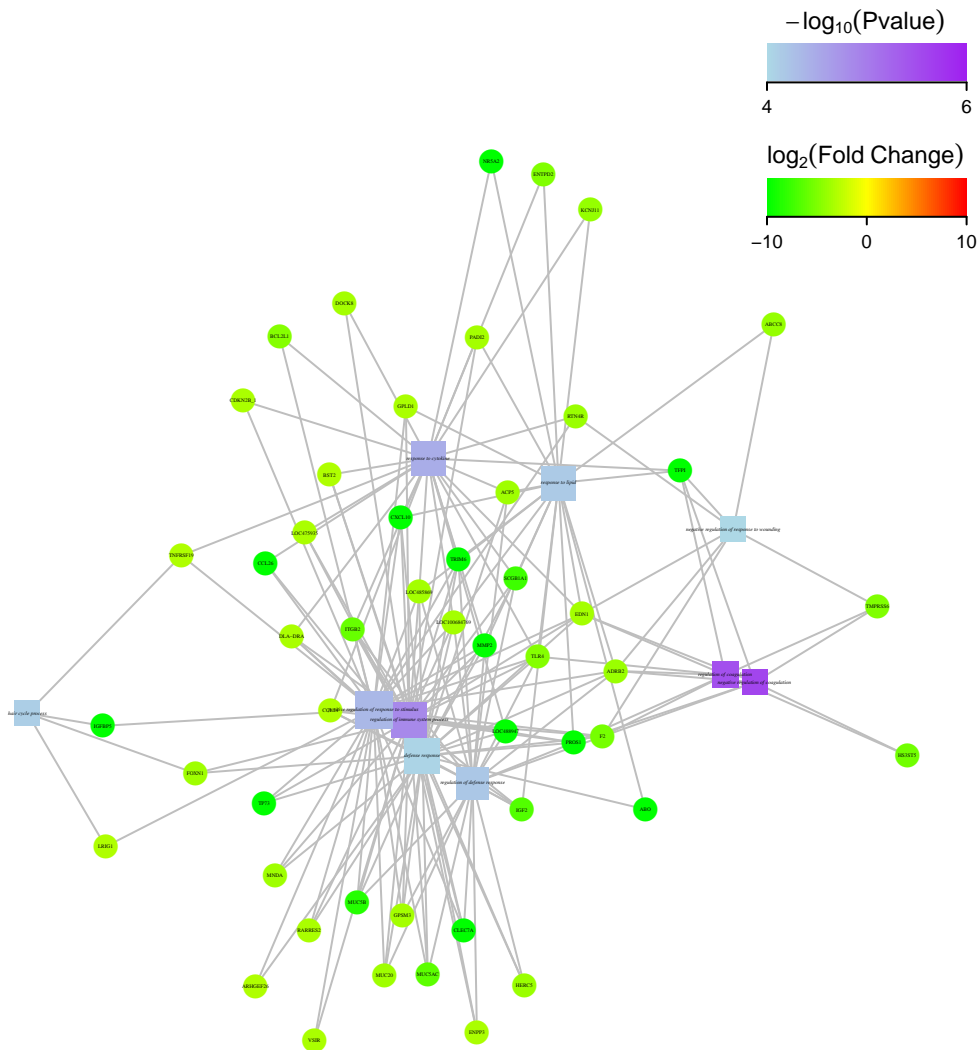

Supplement: S1 File — (ZIP) [file pone.0311069.s002.zip › S2_File/2_GO_enrichment/network/B_vs_A.down_BP_enrichment_network.pdf]

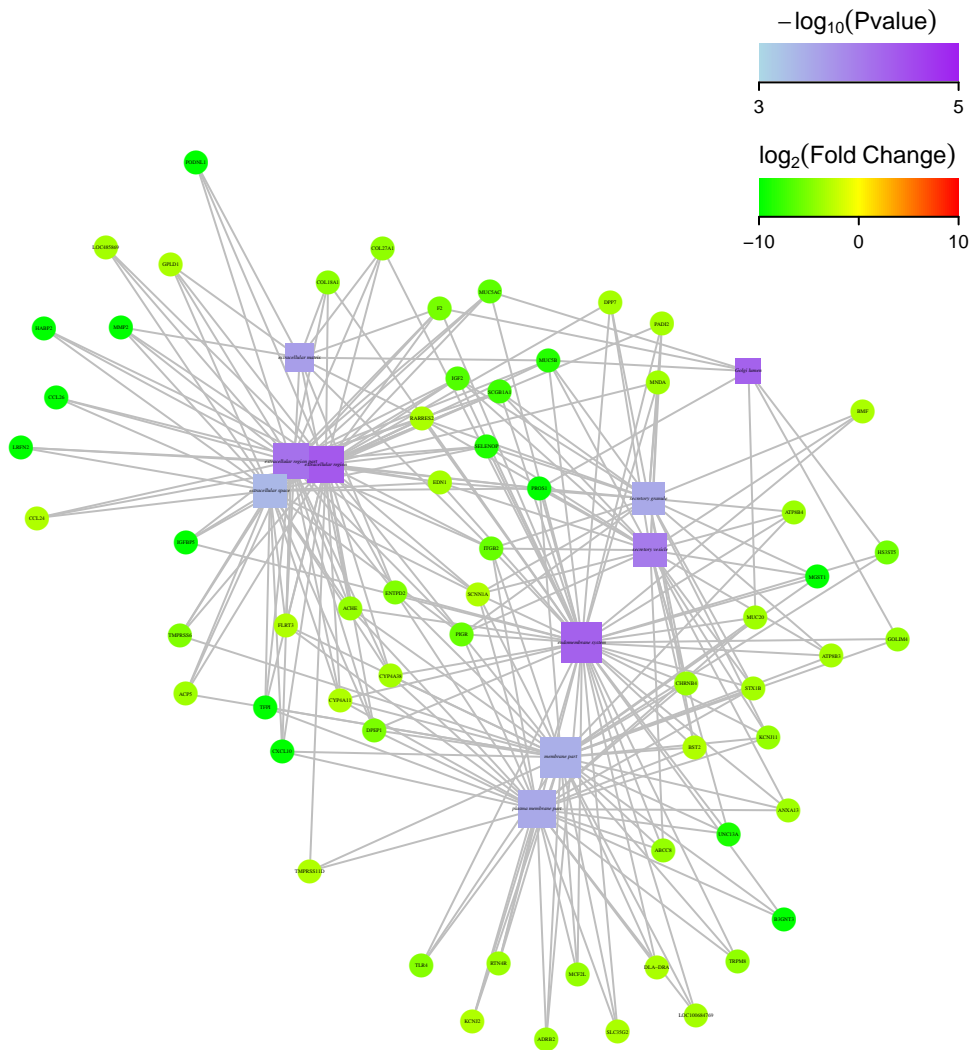

Supplement: S1 File — (ZIP) [file pone.0311069.s002.zip › S2_File/2_GO_enrichment/network/B_vs_A.down_CC_enrichment_network.pdf]

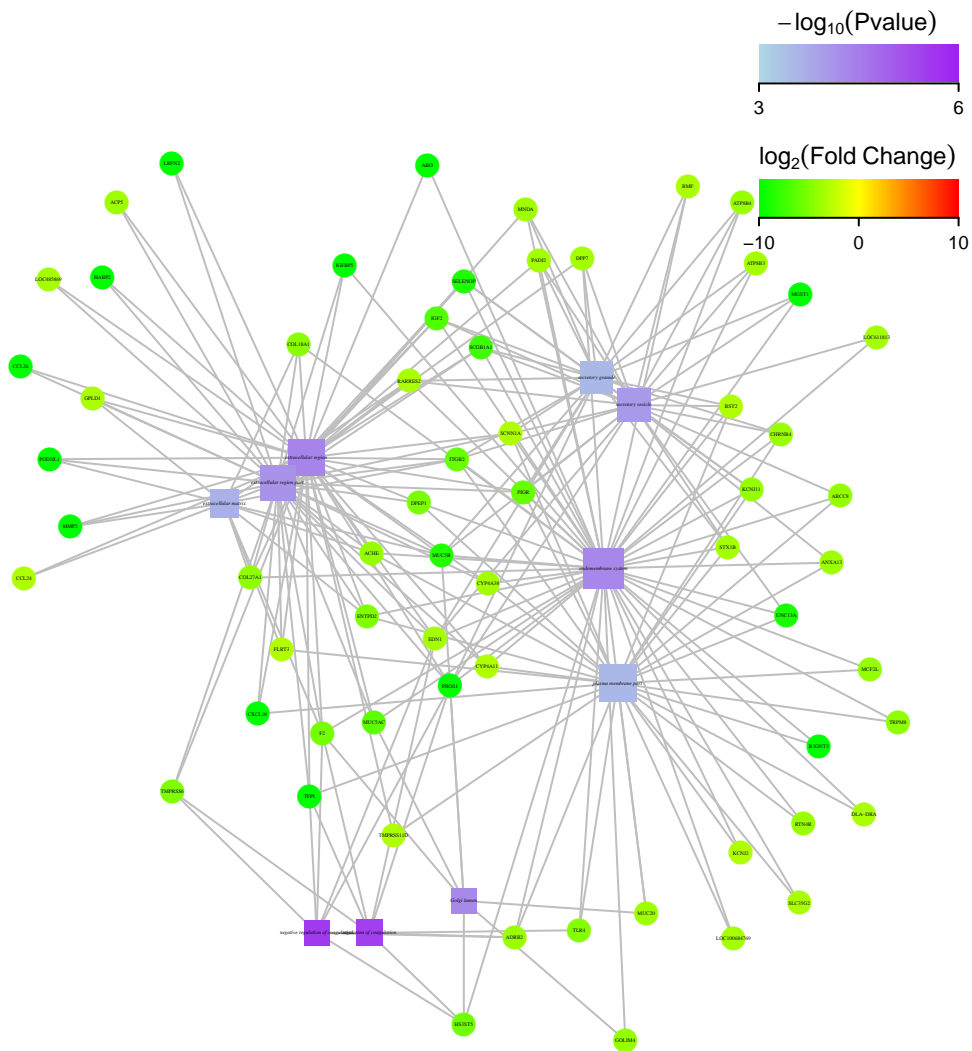

Supplement: S1 File — (ZIP) [file pone.0311069.s002.zip › S2_File/2_GO_enrichment/network/B_vs_A.down_GO_enrichment_network.pdf]

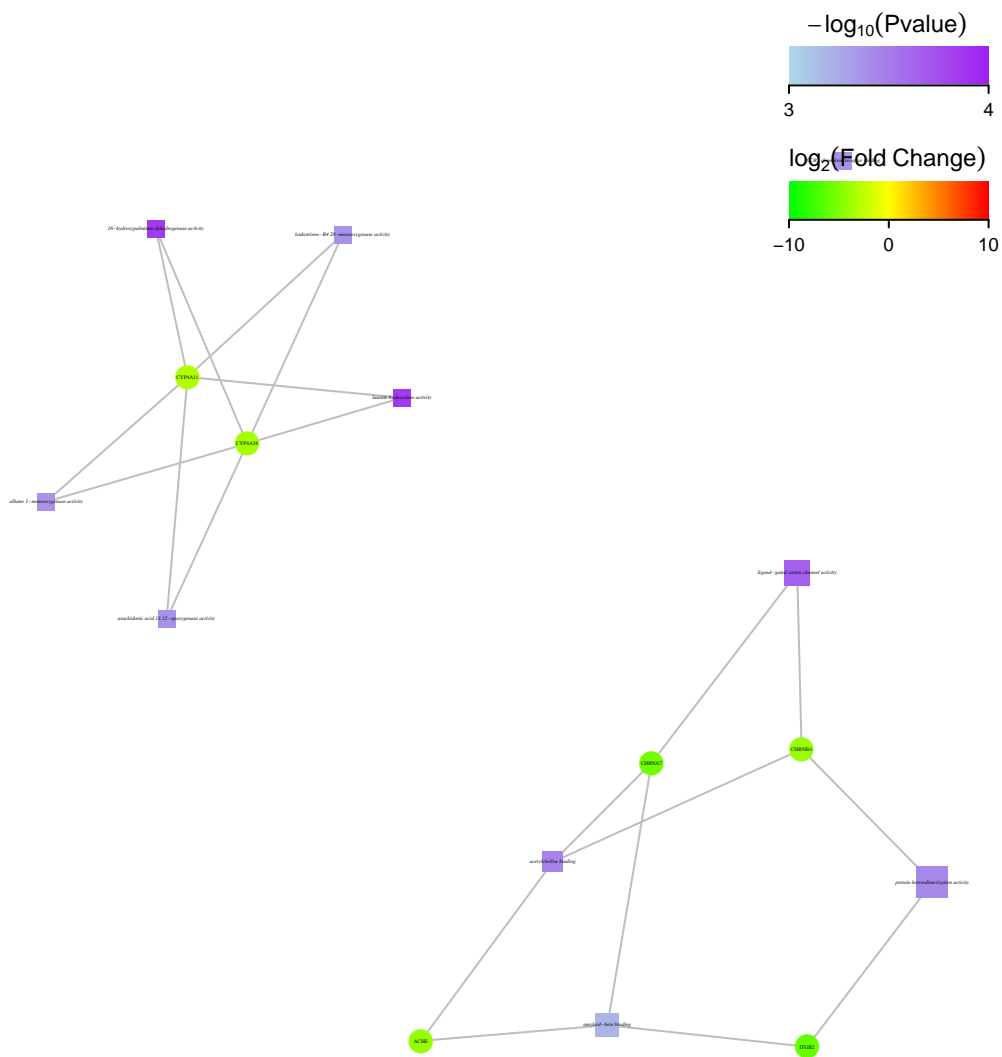

Supplement: S1 File — (ZIP) [file pone.0311069.s002.zip › S2_File/2_GO_enrichment/network/B_vs_A.down_MF_enrichment_network.pdf]

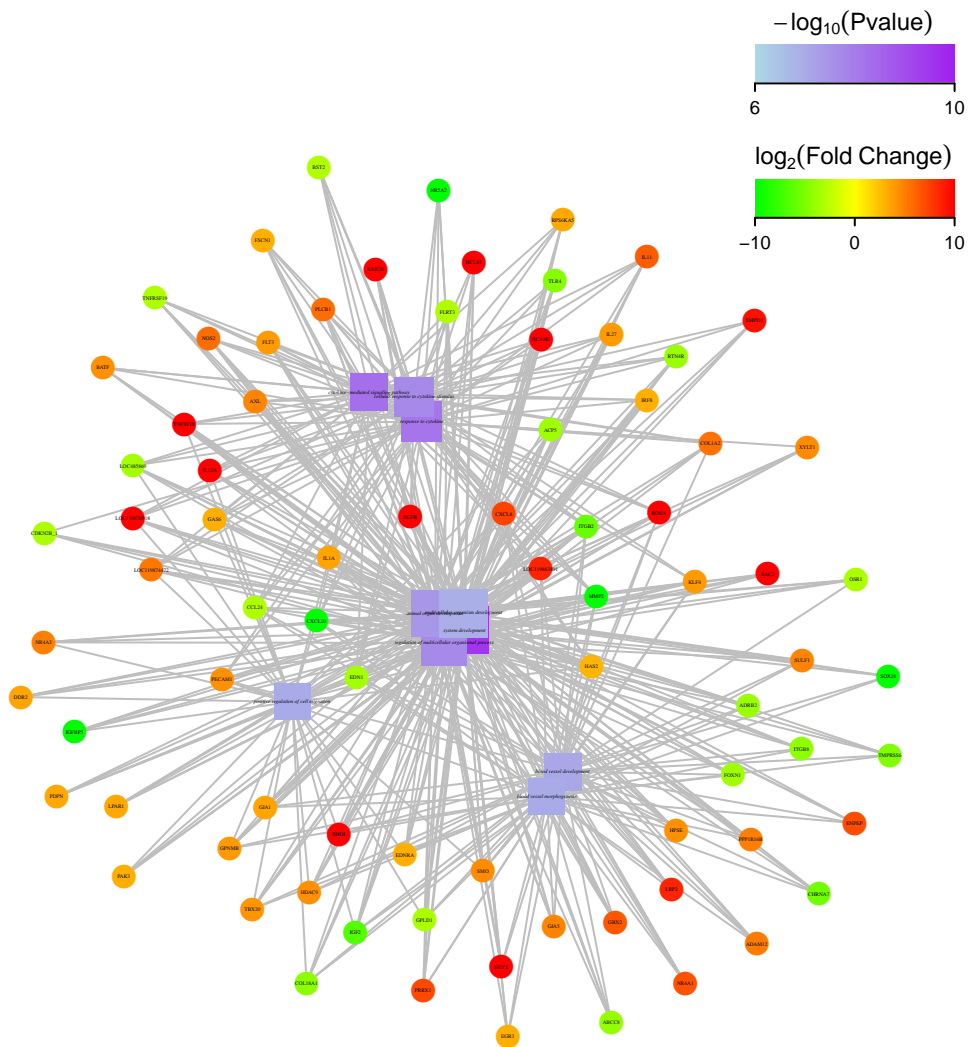

Supplement: S1 File — (ZIP) [file pone.0311069.s002.zip › S2_File/2_GO_enrichment/network/B_vs_A.sign_BP_enrichment_network.pdf]

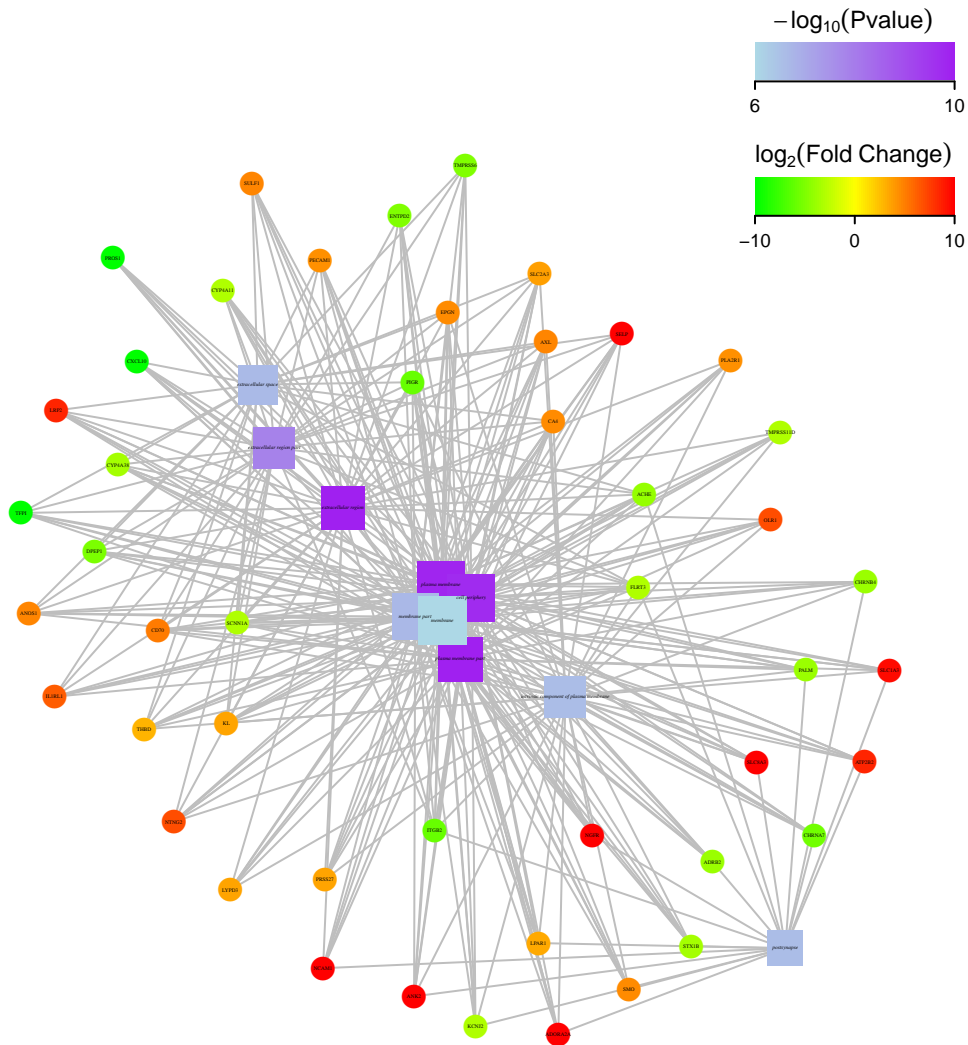

Supplement: S1 File — (ZIP) [file pone.0311069.s002.zip › S2_File/2_GO_enrichment/network/B_vs_A.sign_CC_enrichment_network.pdf]

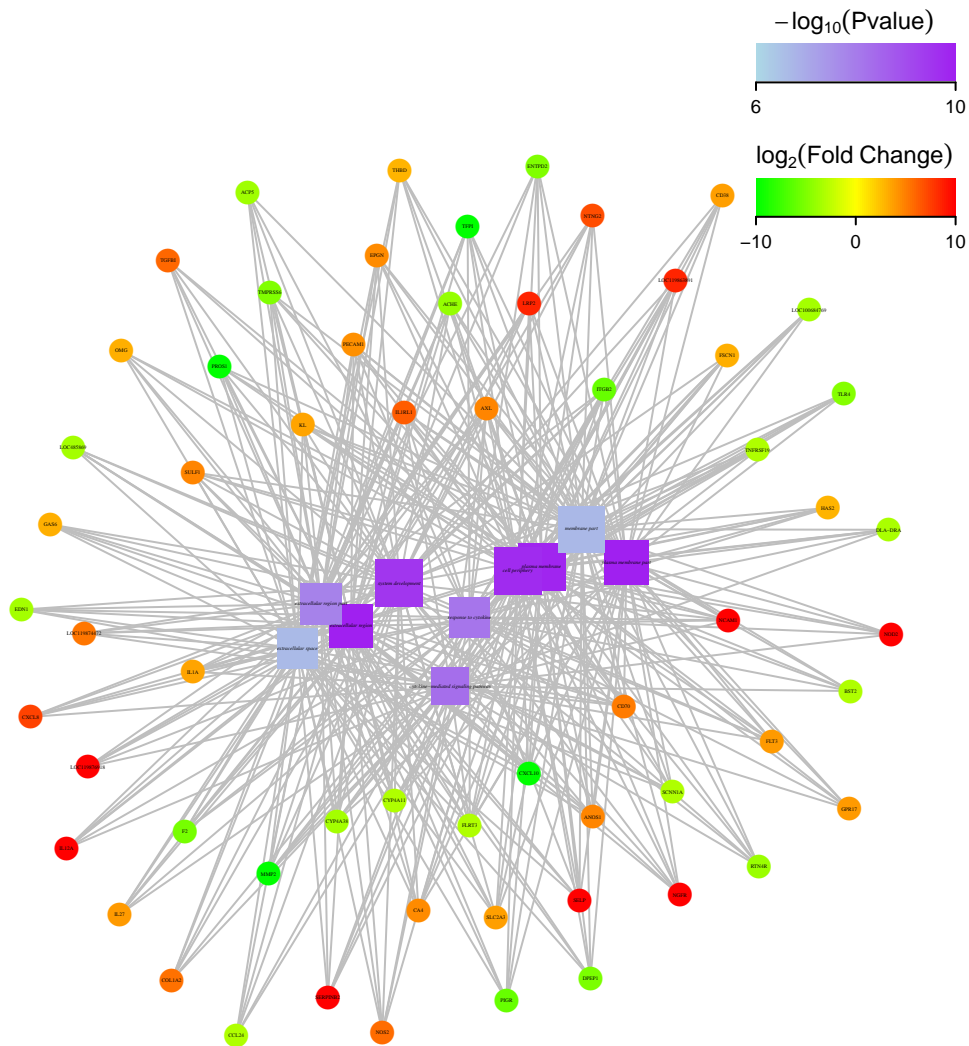

Supplement: S1 File — (ZIP) [file pone.0311069.s002.zip › S2_File/2_GO_enrichment/network/B_vs_A.sign_GO_enrichment_network.pdf]

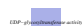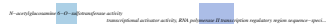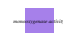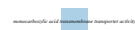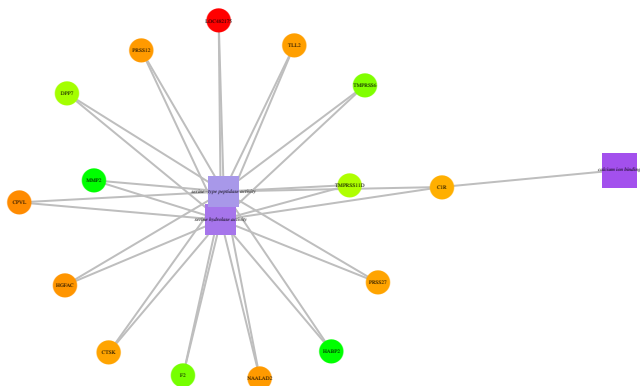

Supplement: S1 File — (ZIP) [file pone.0311069.s002.zip › S2_File/2_GO_enrichment/network/B_vs_A.sign_MF_enrichment_network.pdf]

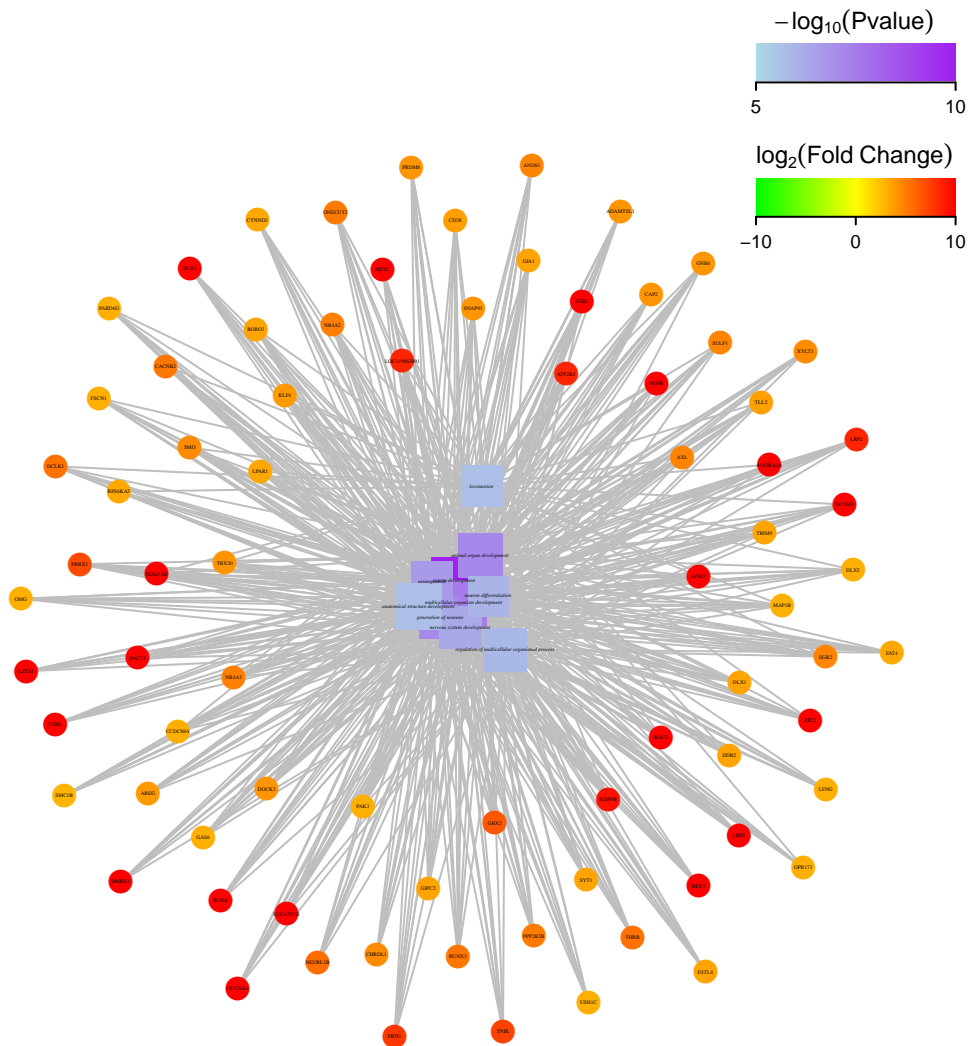

Supplement: S1 File — (ZIP) [file pone.0311069.s002.zip › S2_File/2_GO_enrichment/network/B_vs_A.up_BP_enrichment_network.pdf]

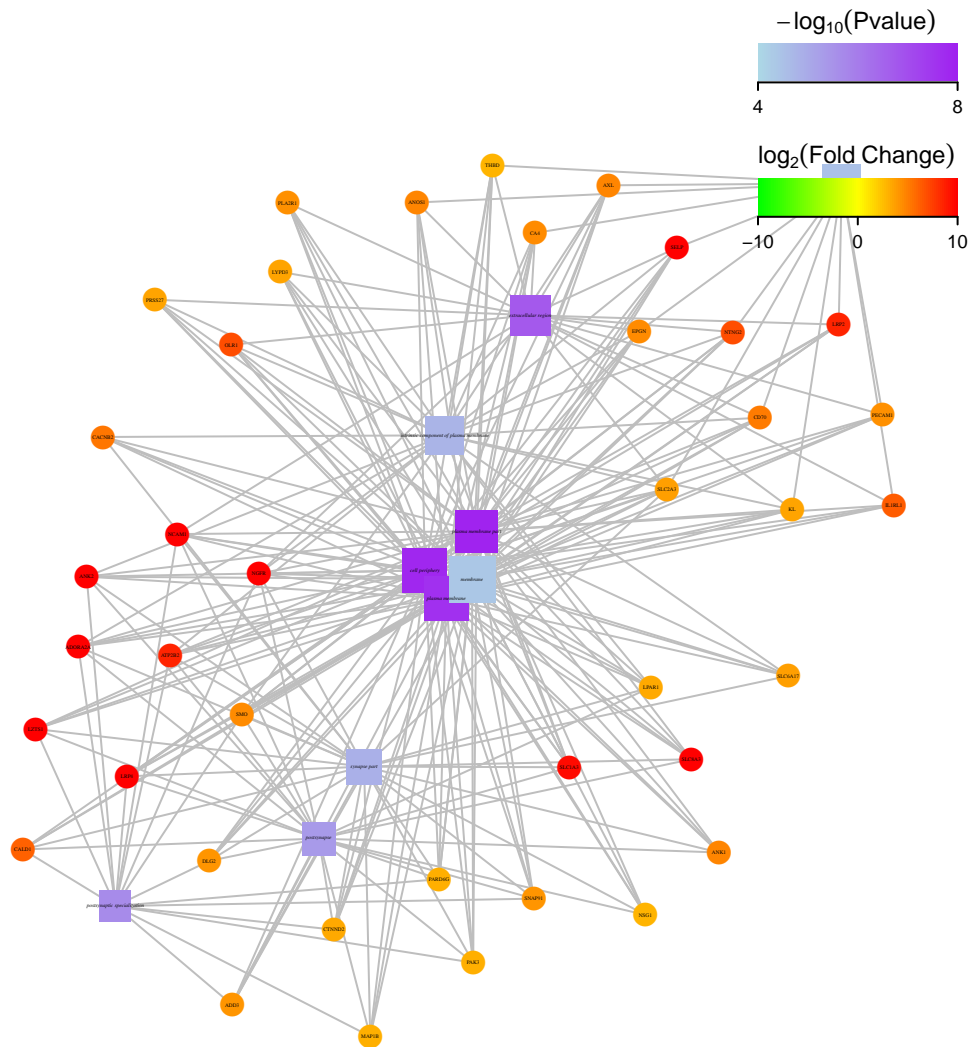

Supplement: S1 File — (ZIP) [file pone.0311069.s002.zip › S2_File/2_GO_enrichment/network/B_vs_A.up_CC_enrichment_network.pdf]

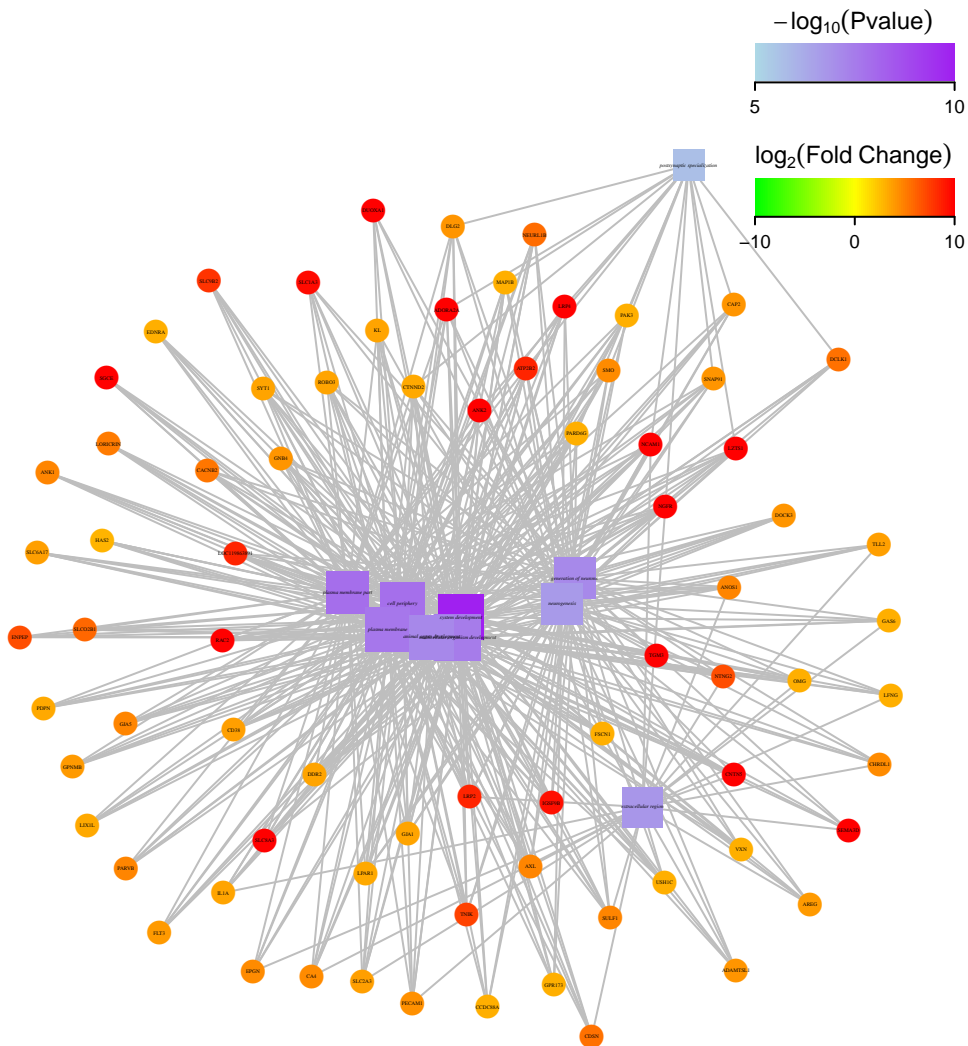

Supplement: S1 File — (ZIP) [file pone.0311069.s002.zip › S2_File/2_GO_enrichment/network/B_vs_A.up_GO_enrichment_network.pdf]

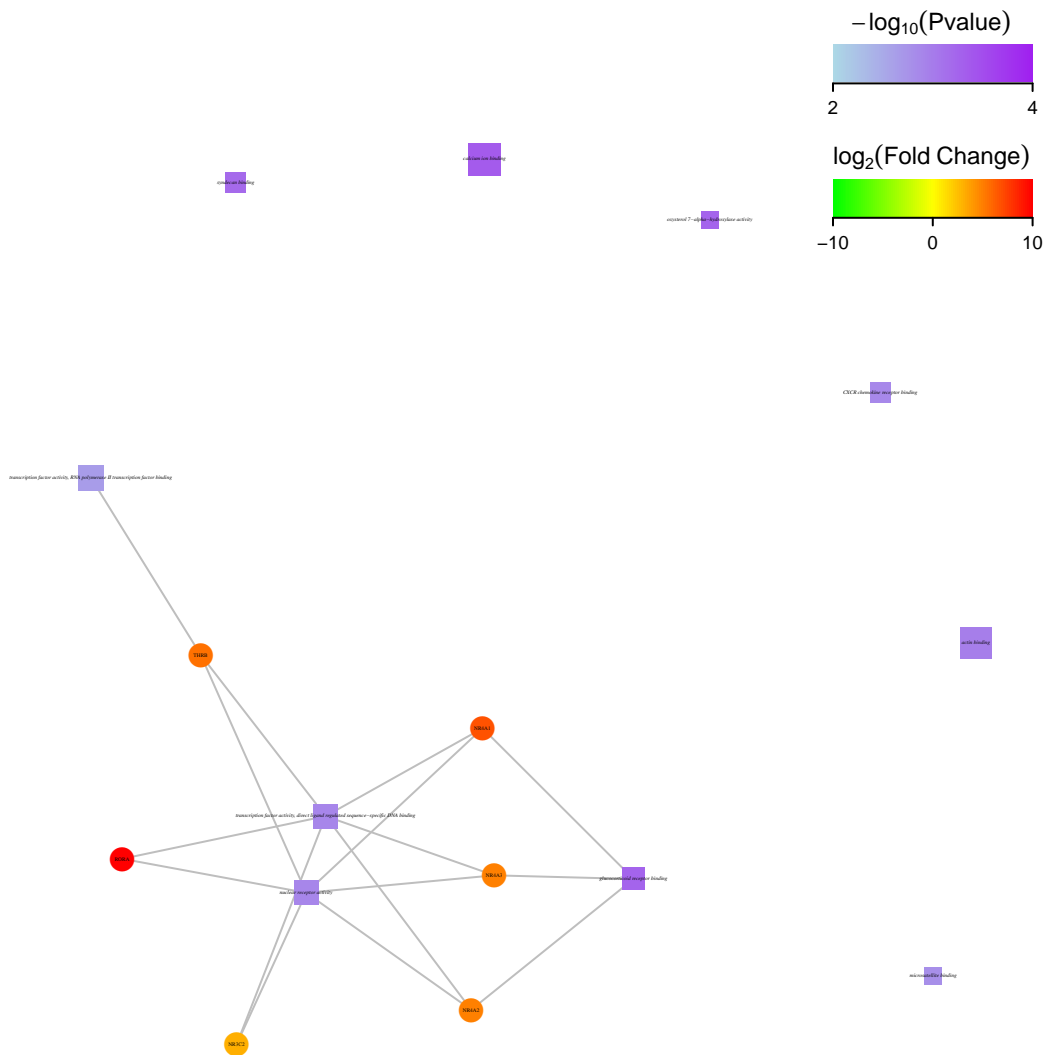

Supplement: S1 File — (ZIP) [file pone.0311069.s002.zip › S2_File/2_GO_enrichment/network/B_vs_A.up_MF_enrichment_network.pdf]

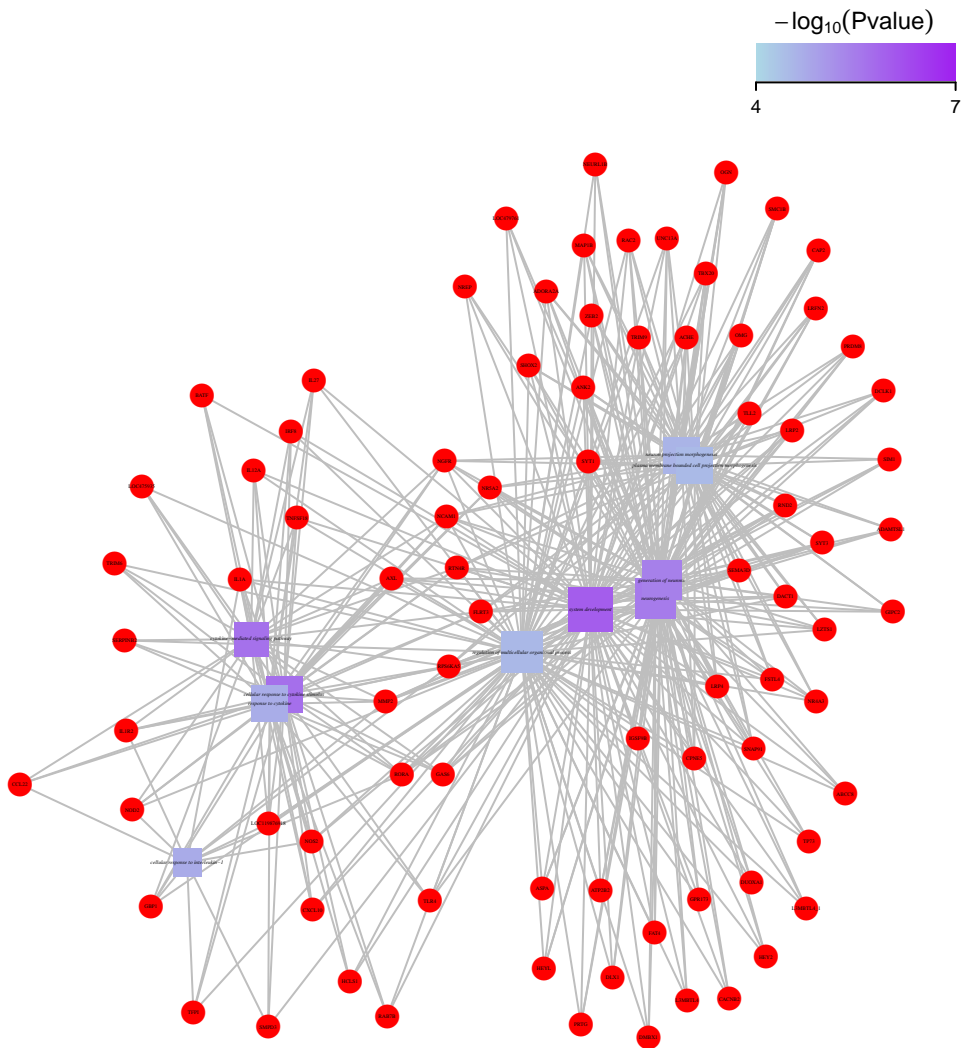

Supplement: S1 File — (ZIP) [file pone.0311069.s002.zip › S2_File/2_GO_enrichment/network/union.DE_gene.subcluster1_BP_enrichment_network.pdf]

$-\log_{10}(\text{Pvalue})$

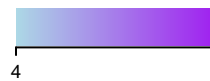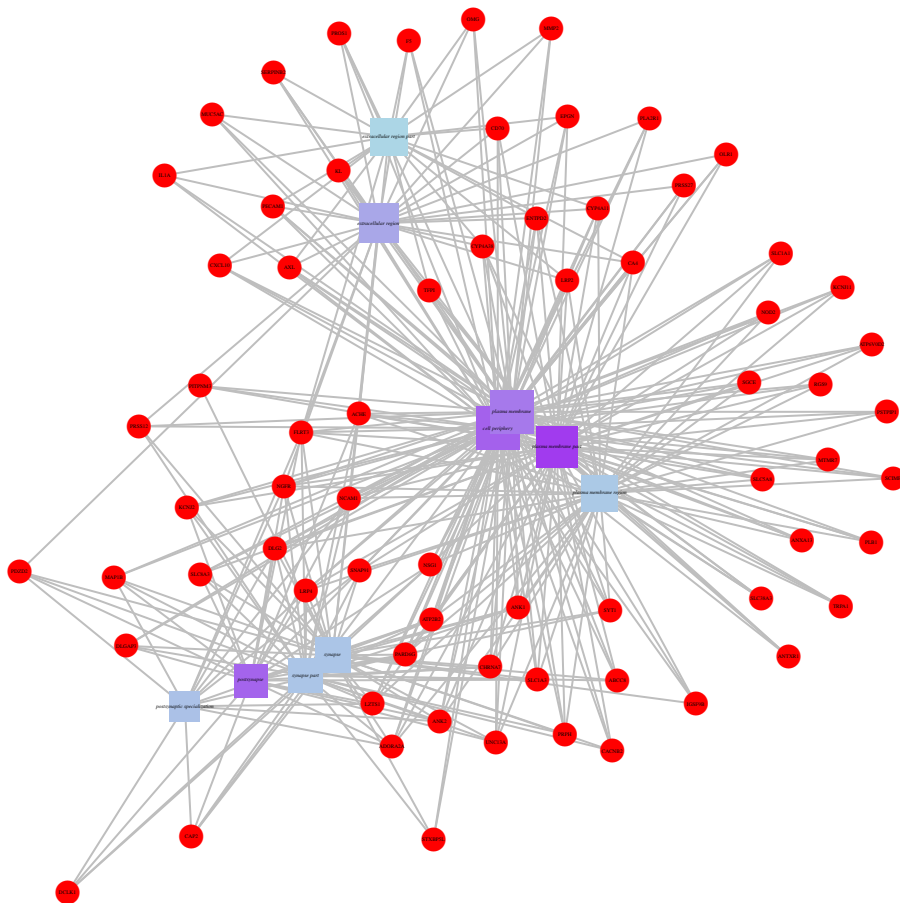

Supplement: S1 File — (ZIP) [file pone.0311069.s002.zip › S2_File/2_GO_enrichment/network/union.DE_gene.subcluster1_CC_enrichment_network.pdf]

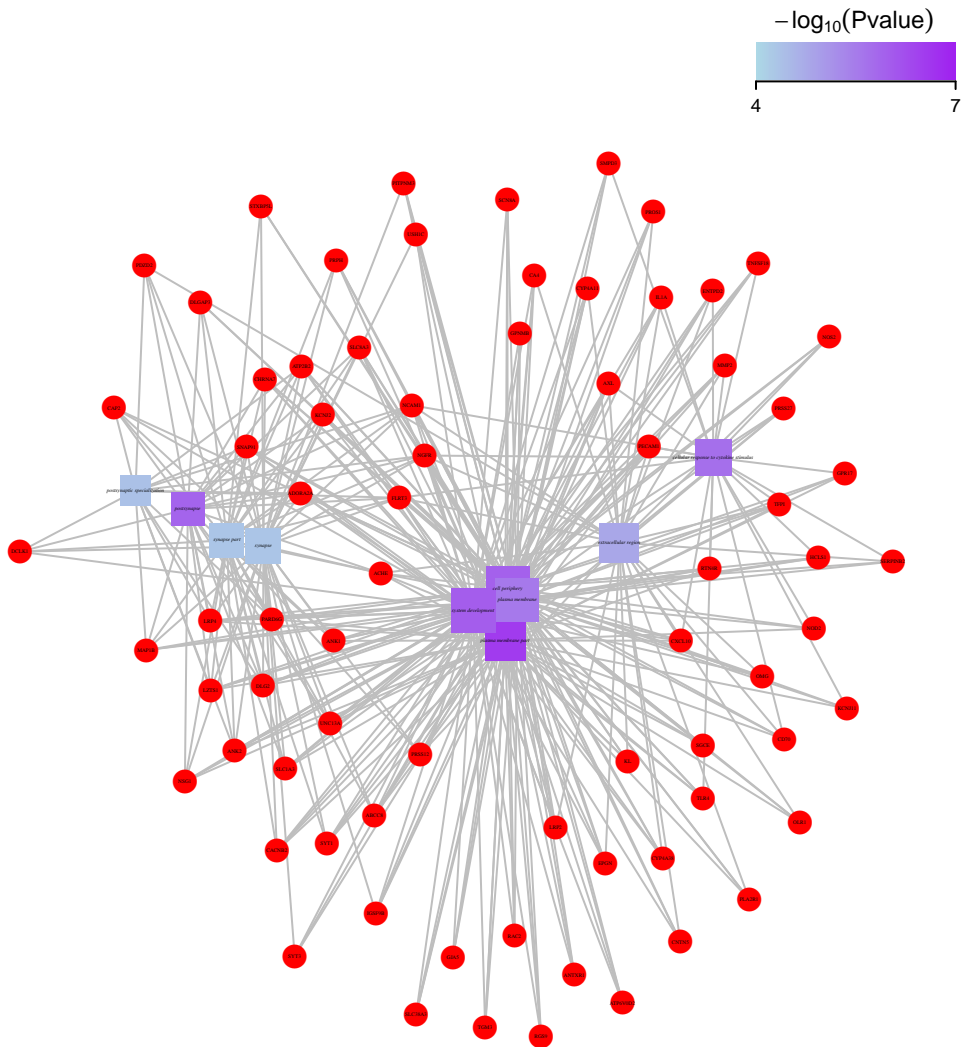

Supplement: S1 File — (ZIP) [file pone.0311069.s002.zip › S2_File/2_GO_enrichment/network/union.DE_gene.subcluster1_GO_enrichment_network.pdf]

# BP Enrichment

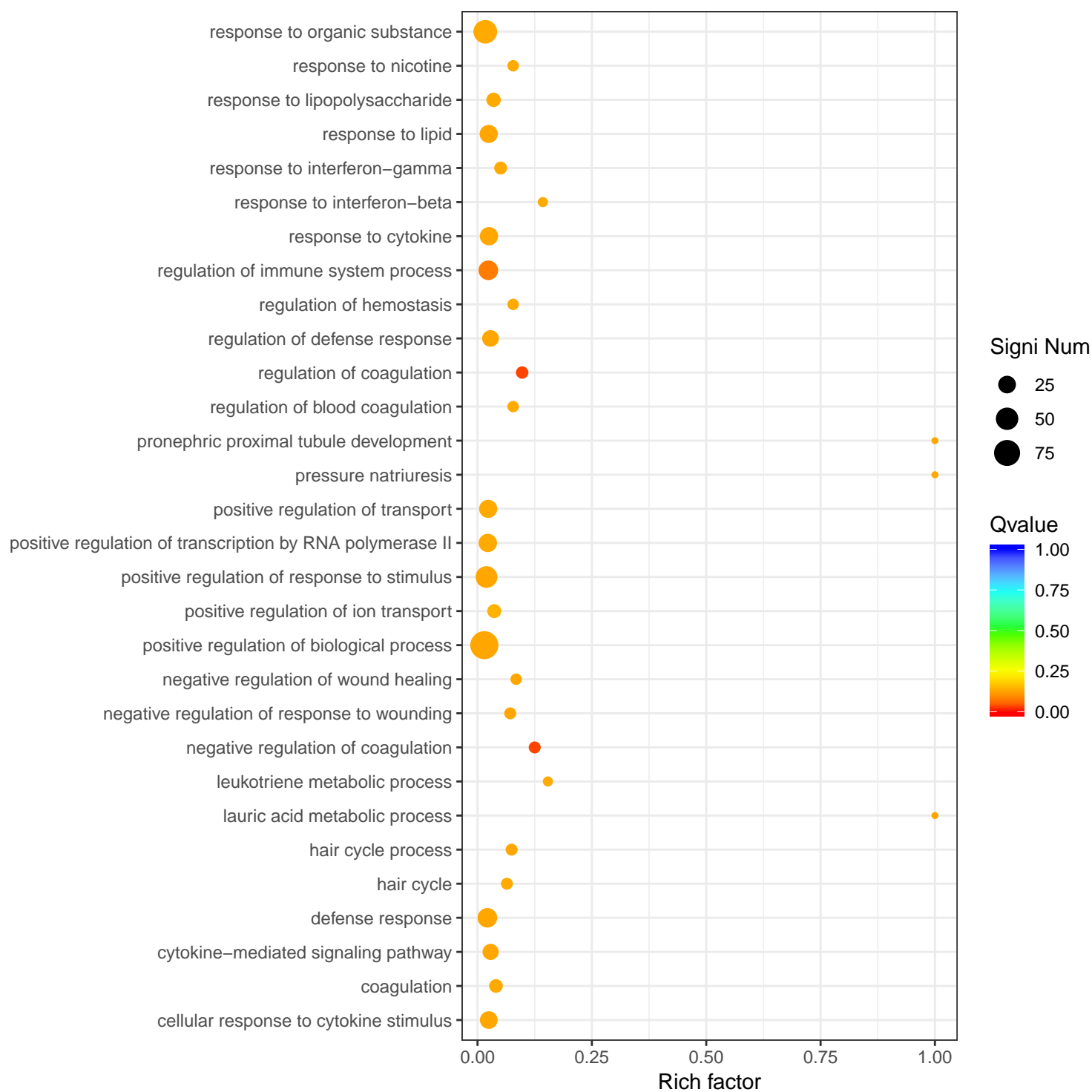

Supplement: S1 File — (ZIP) [file pone.0311069.s002.zip › S2_File/2_GO_enrichment/scatter/B_vs_A.down_BP_enrichment_scatter.pdf]

# CC Enrichment

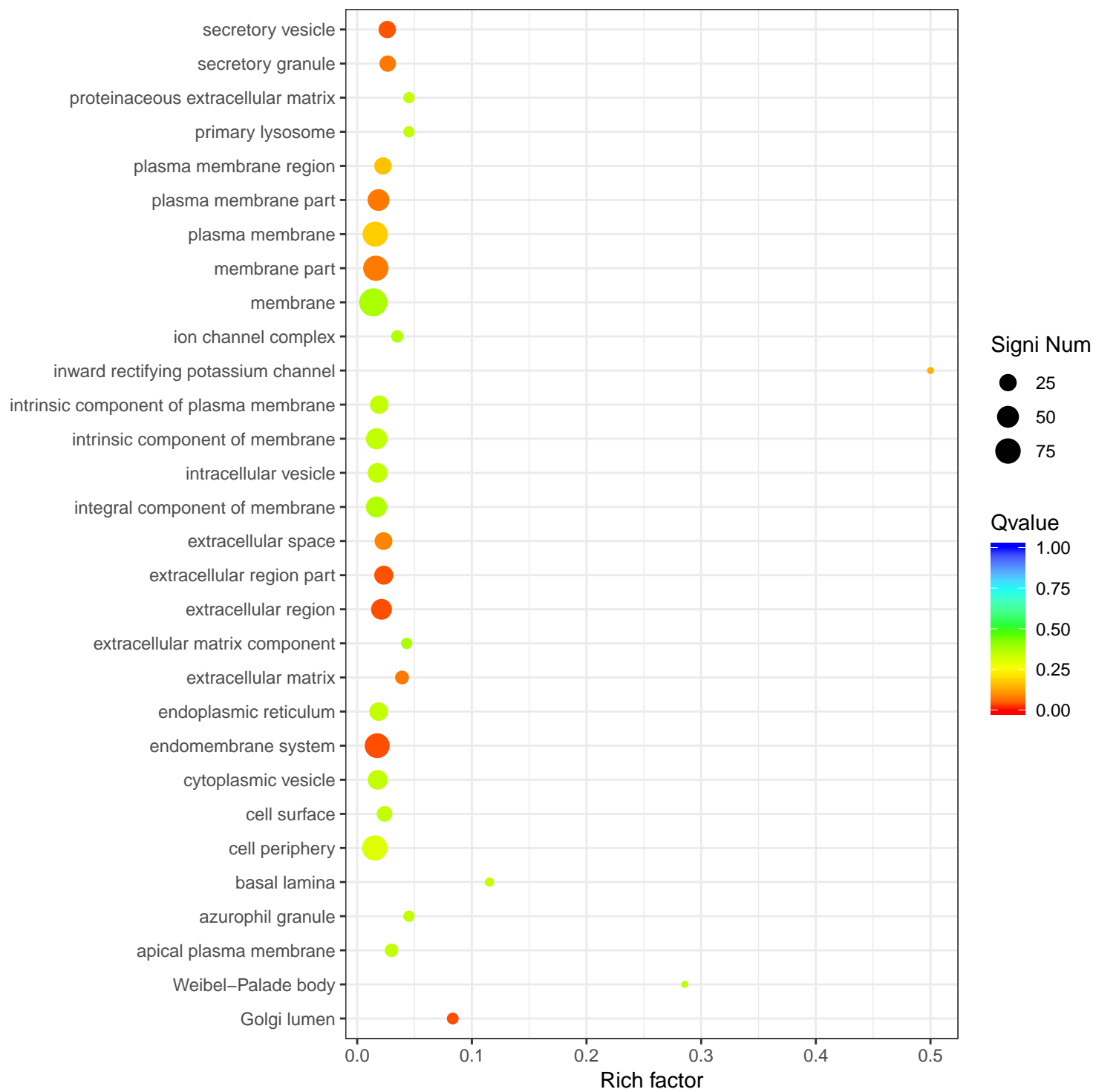

Supplement: S1 File — (ZIP) [file pone.0311069.s002.zip › S2_File/2_GO_enrichment/scatter/B_vs_A.down_CC_enrichment_scatter.pdf]

# GO Enrichment

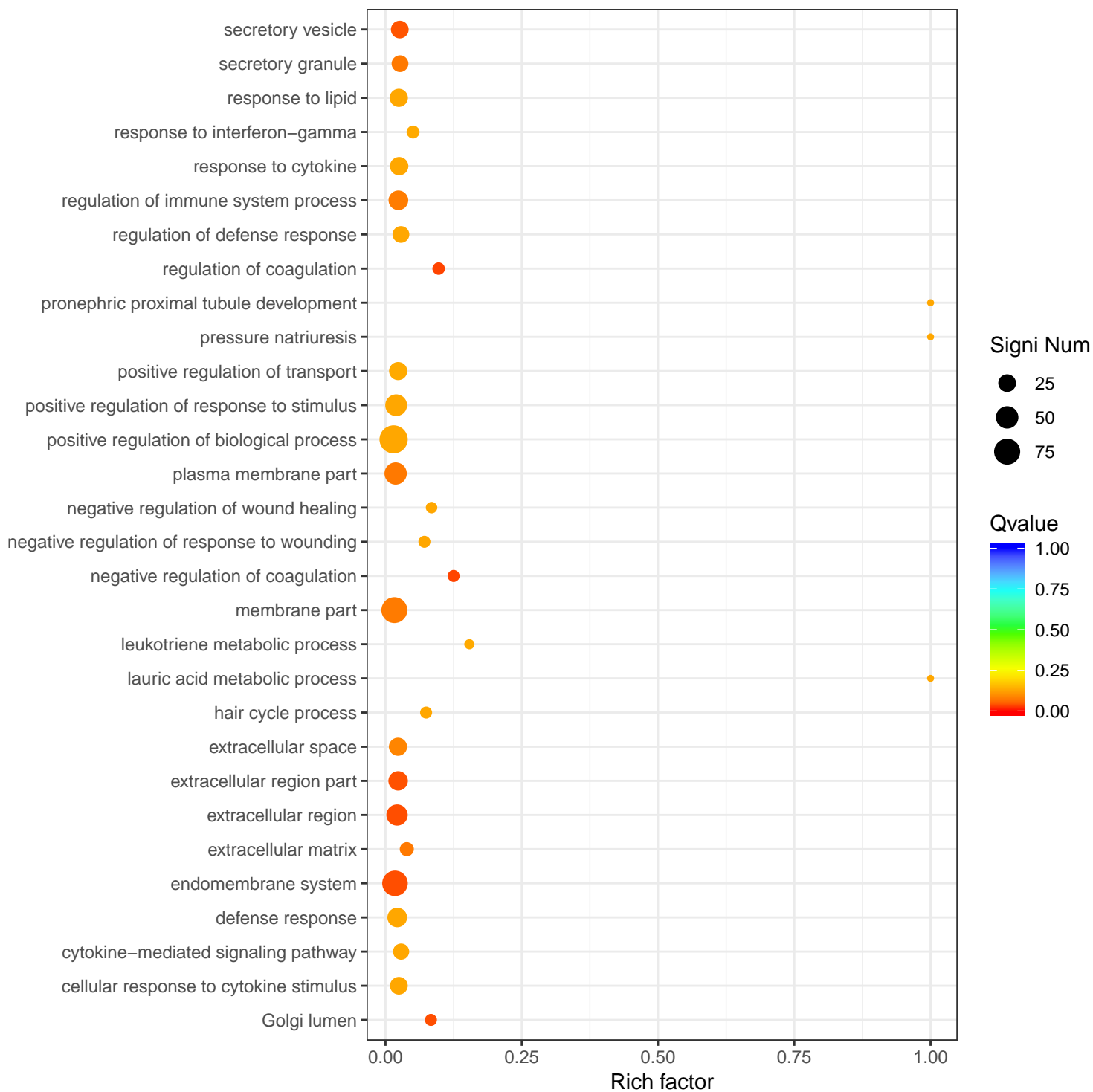

Supplement: S1 File — (ZIP) [file pone.0311069.s002.zip › S2_File/2_GO_enrichment/scatter/B_vs_A.down_GO_enrichment_scatter.pdf]

# MF Enrichment

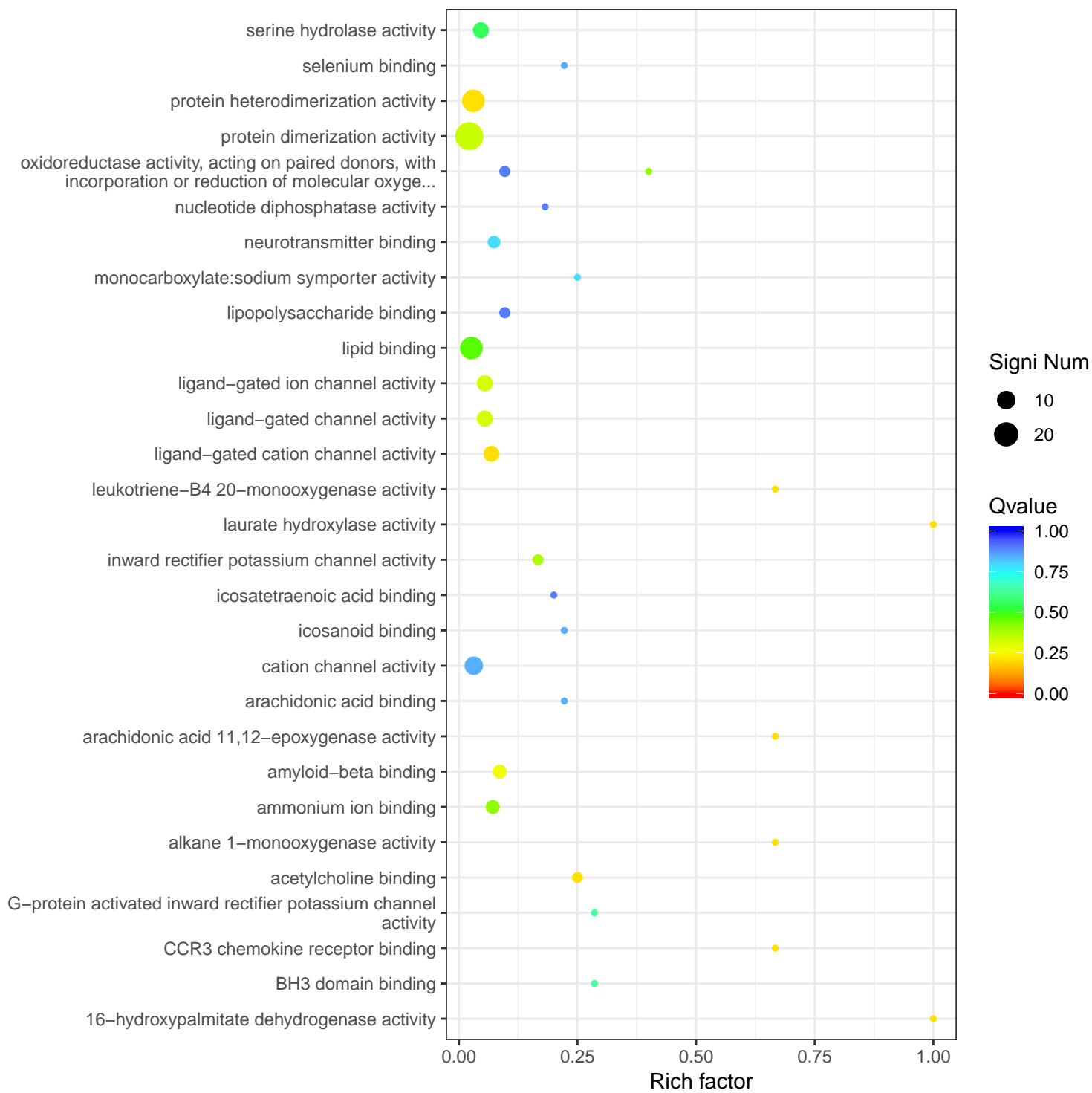

Supplement: S1 File — (ZIP) [file pone.0311069.s002.zip › S2_File/2_GO_enrichment/scatter/B_vs_A.down_MF_enrichment_scatter.pdf]

# BP Enrichment

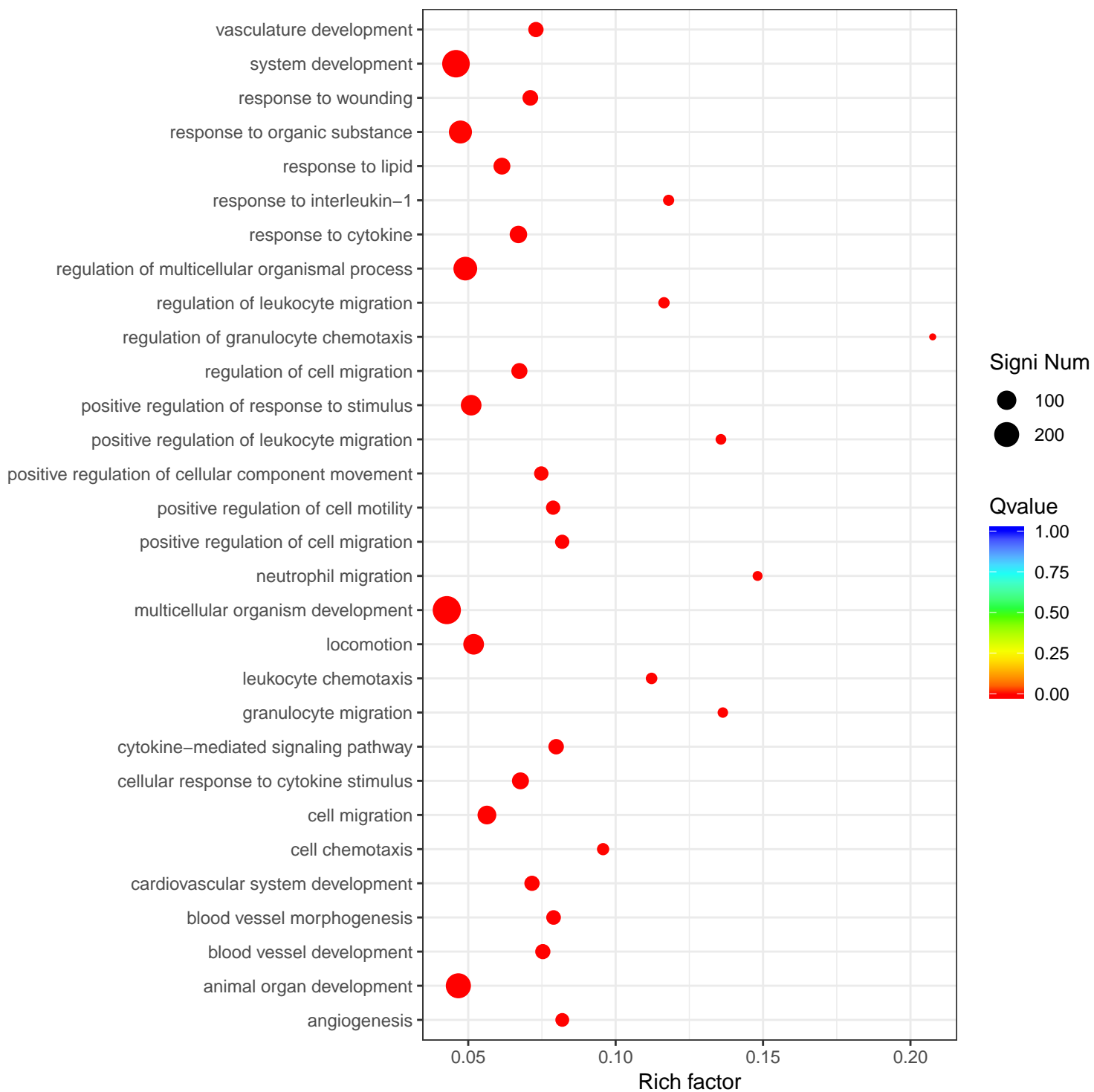

Supplement: S1 File — (ZIP) [file pone.0311069.s002.zip › S2_File/2_GO_enrichment/scatter/B_vs_A.sign_BP_enrichment_scatter.pdf]

# CC Enrichment

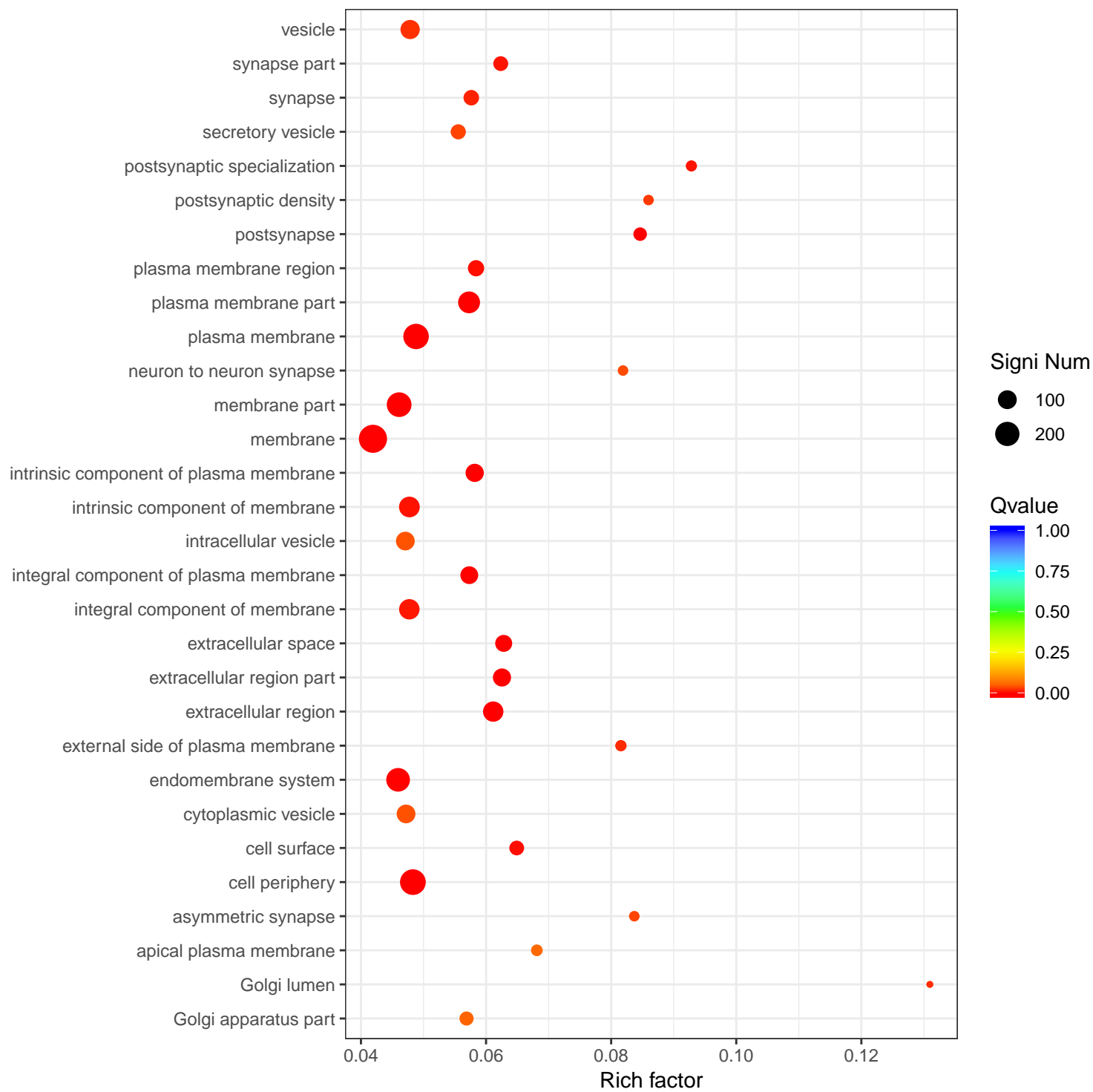

Supplement: S1 File — (ZIP) [file pone.0311069.s002.zip › S2_File/2_GO_enrichment/scatter/B_vs_A.sign_CC_enrichment_scatter.pdf]

# GO Enrichment

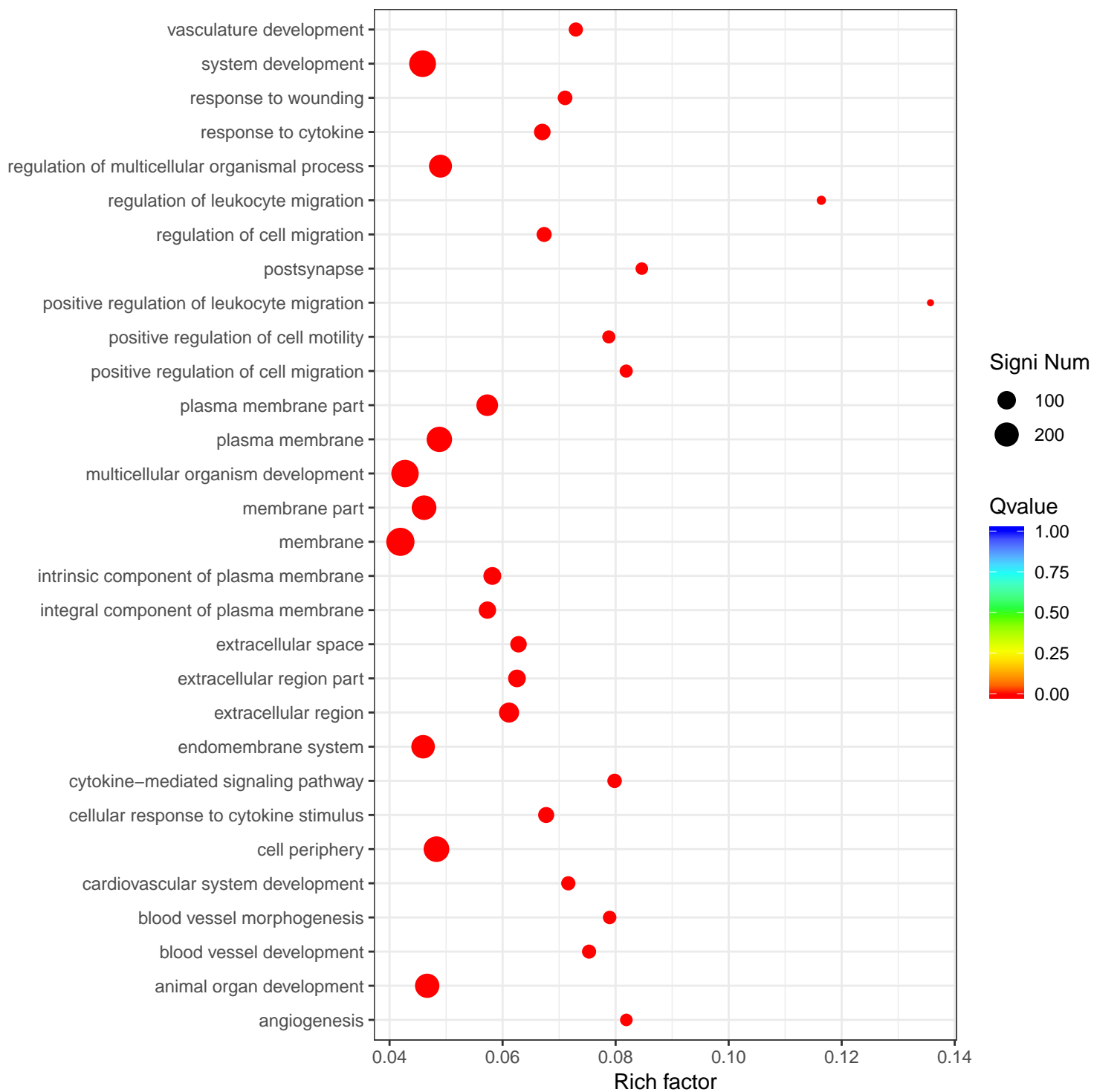

Supplement: S1 File — (ZIP) [file pone.0311069.s002.zip › S2_File/2_GO_enrichment/scatter/B_vs_A.sign_GO_enrichment_scatter.pdf]

# MF Enrichment

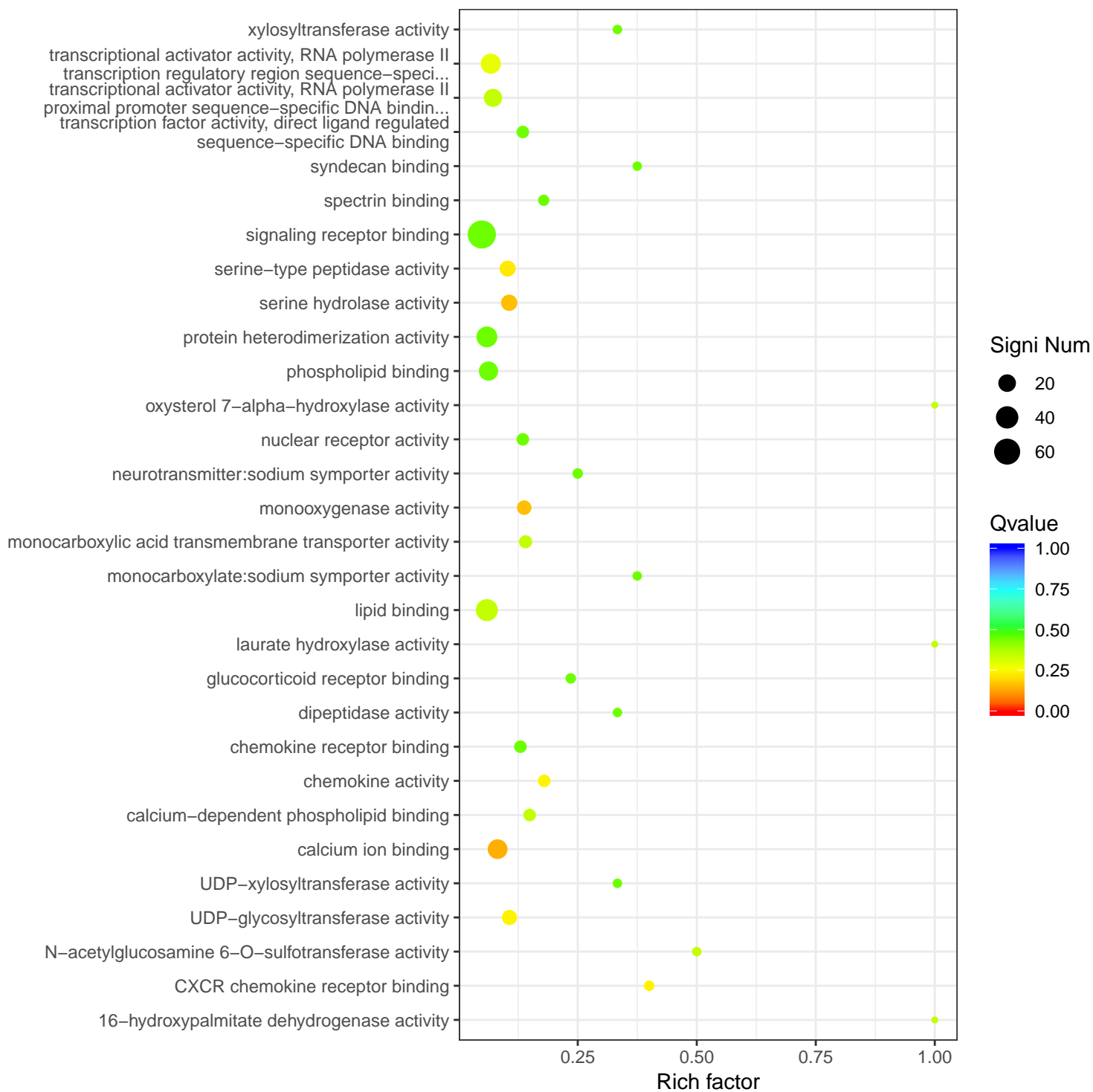

Supplement: S1 File — (ZIP) [file pone.0311069.s002.zip › S2_File/2_GO_enrichment/scatter/B_vs_A.sign_MF_enrichment_scatter.pdf]

# BP Enrichment

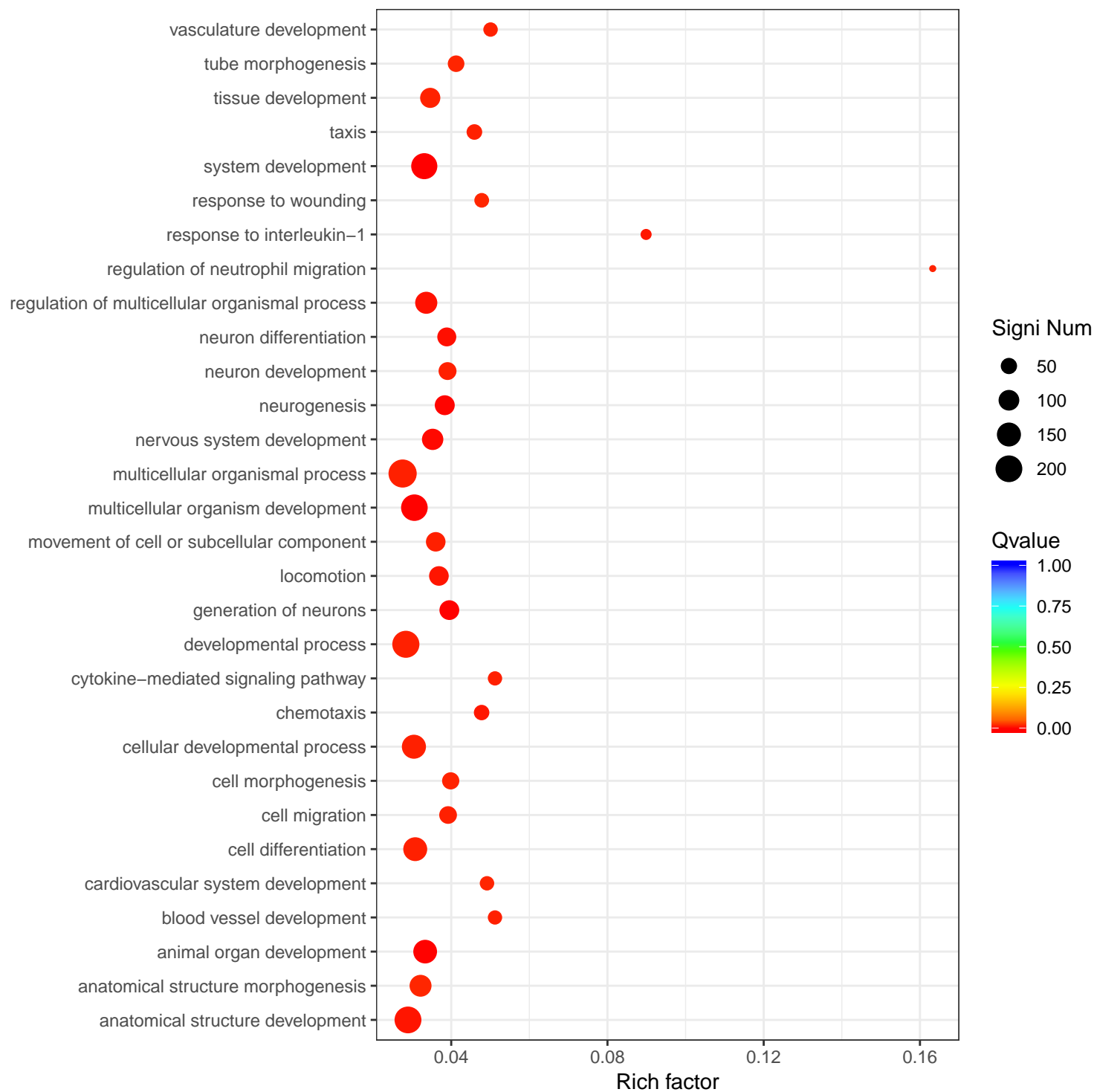

Supplement: S1 File — (ZIP) [file pone.0311069.s002.zip › S2_File/2_GO_enrichment/scatter/B_vs_A.up_BP_enrichment_scatter.pdf]

# CC Enrichment

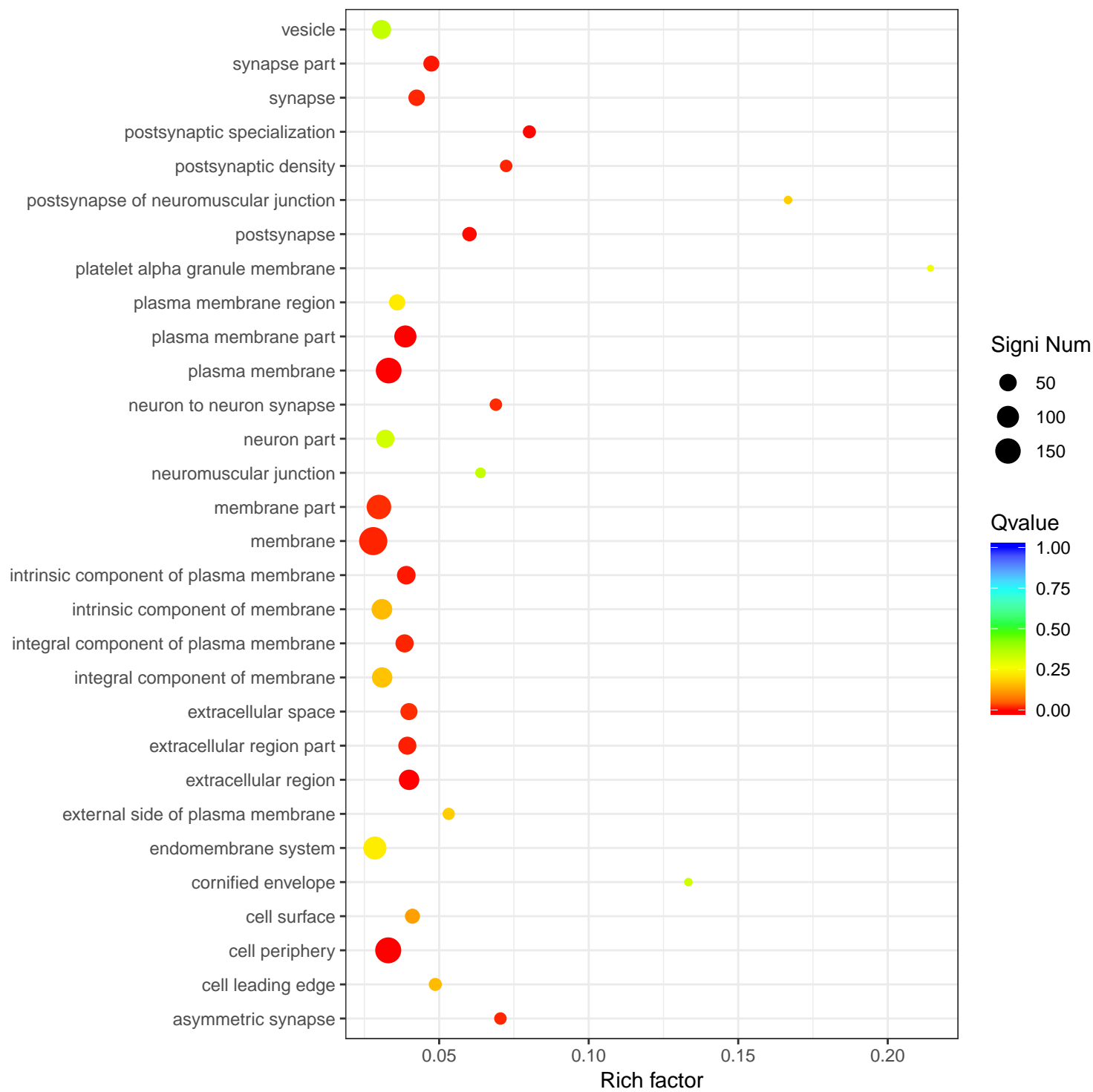

Supplement: S1 File — (ZIP) [file pone.0311069.s002.zip › S2_File/2_GO_enrichment/scatter/B_vs_A.up_CC_enrichment_scatter.pdf]

# GO Enrichment

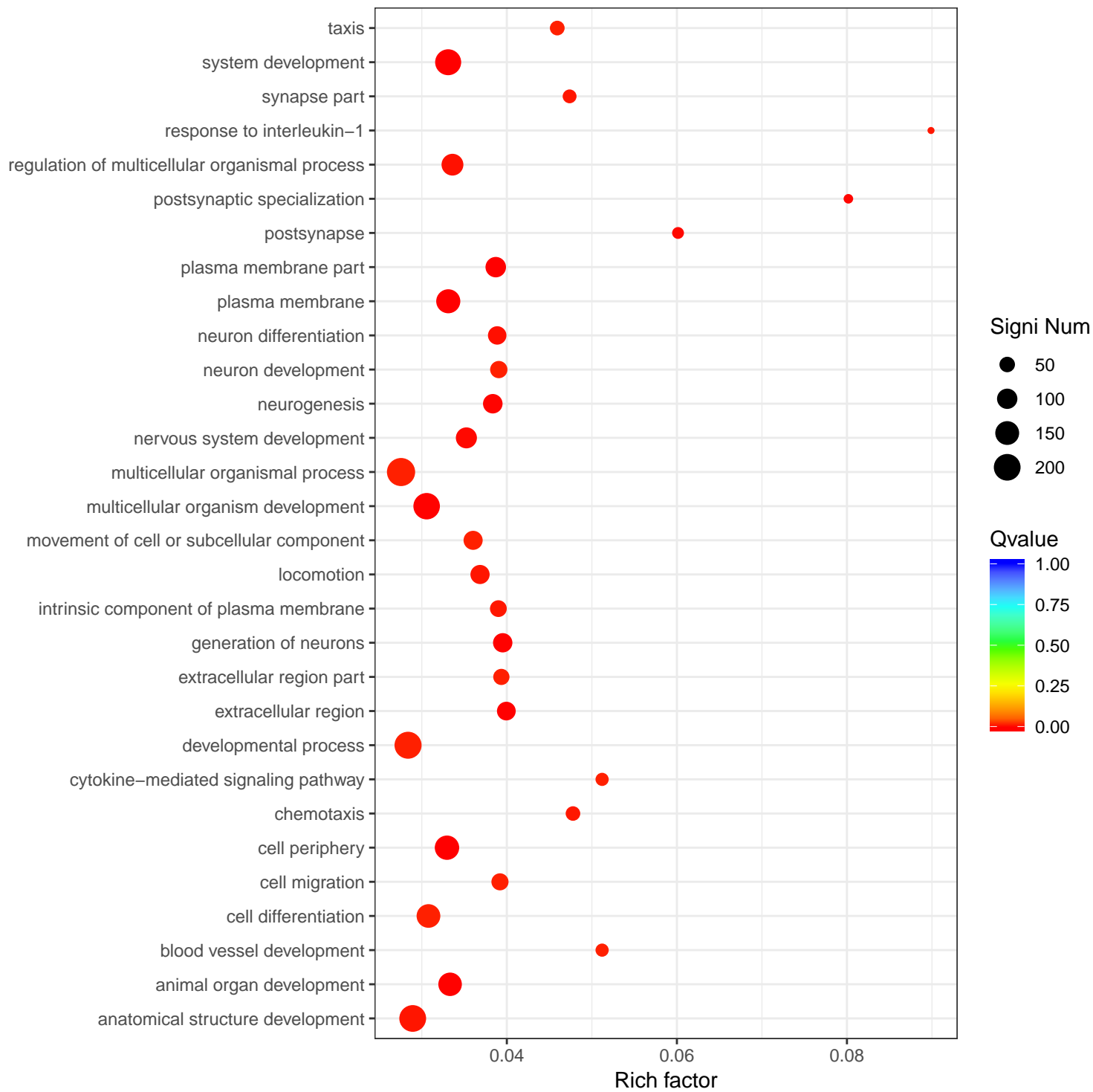

Supplement: S1 File — (ZIP) [file pone.0311069.s002.zip › S2_File/2_GO_enrichment/scatter/B_vs_A.up_GO_enrichment_scatter.pdf]

# MF Enrichment

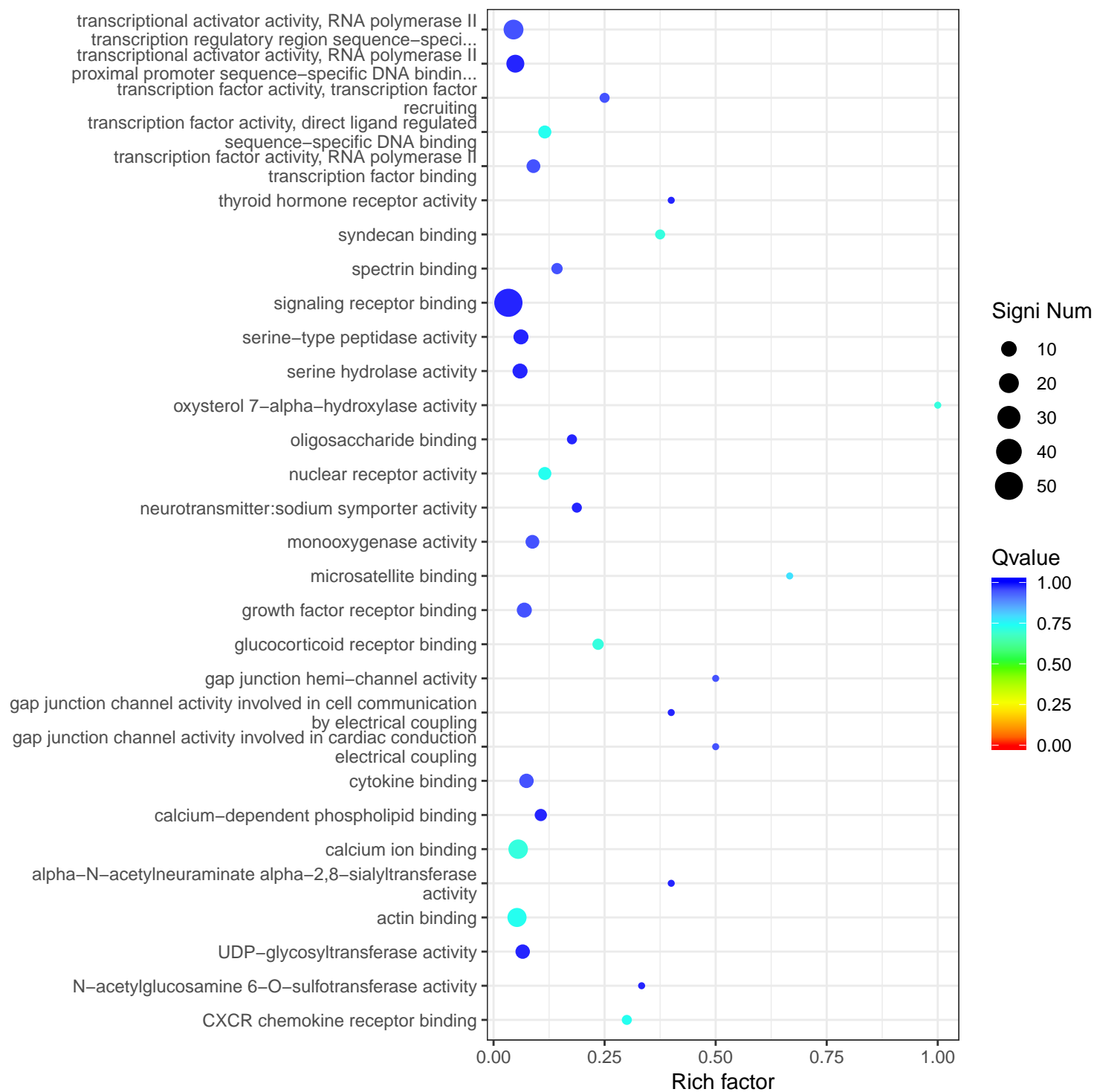

Supplement: S1 File — (ZIP) [file pone.0311069.s002.zip › S2_File/2_GO_enrichment/scatter/B_vs_A.up_MF_enrichment_scatter.pdf]

# BP Enrichment

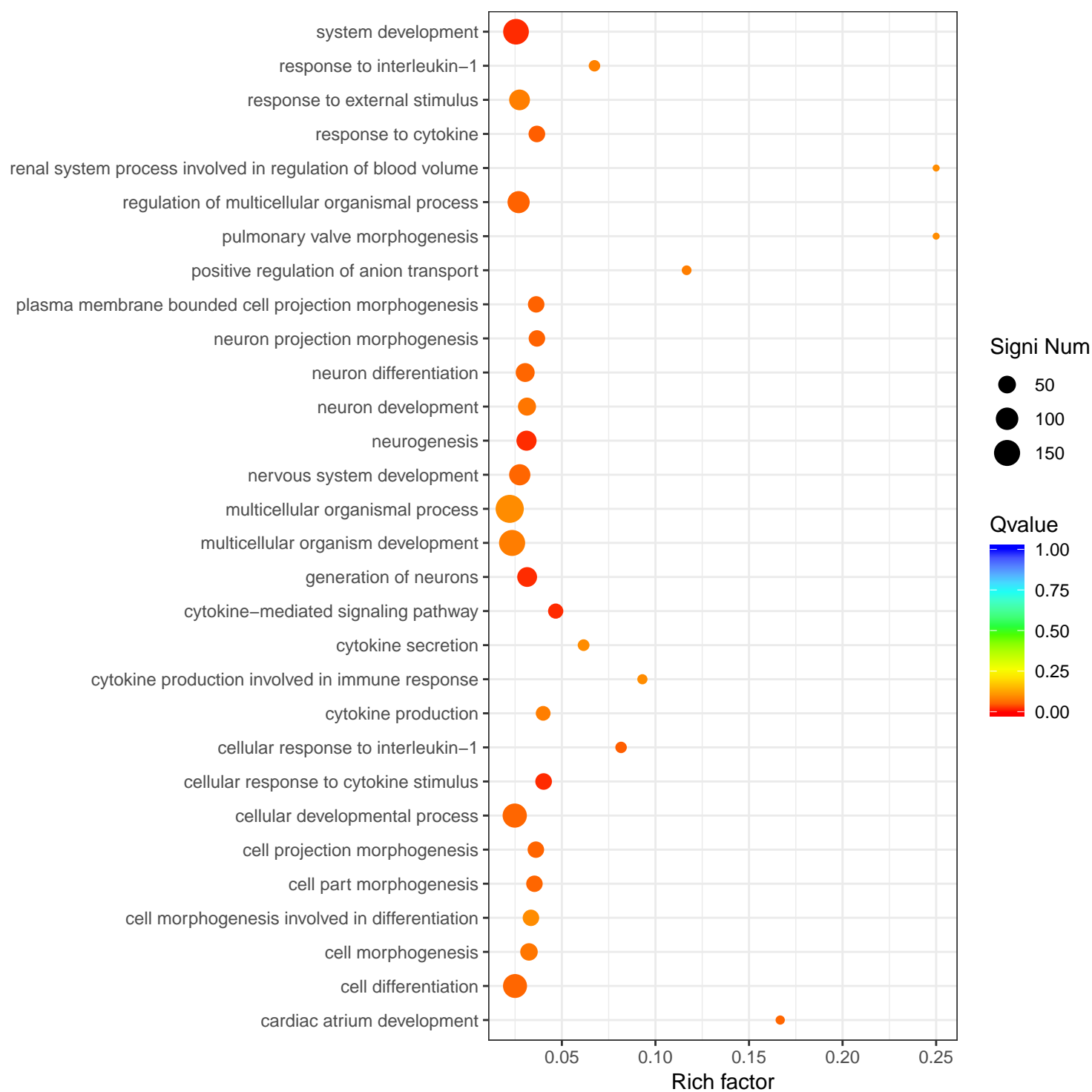

Supplement: S1 File — (ZIP) [file pone.0311069.s002.zip › S2_File/2_GO_enrichment/scatter/union.DE_gene.subcluster1_BP_enrichment_scatter.pdf]

# CC Enrichment

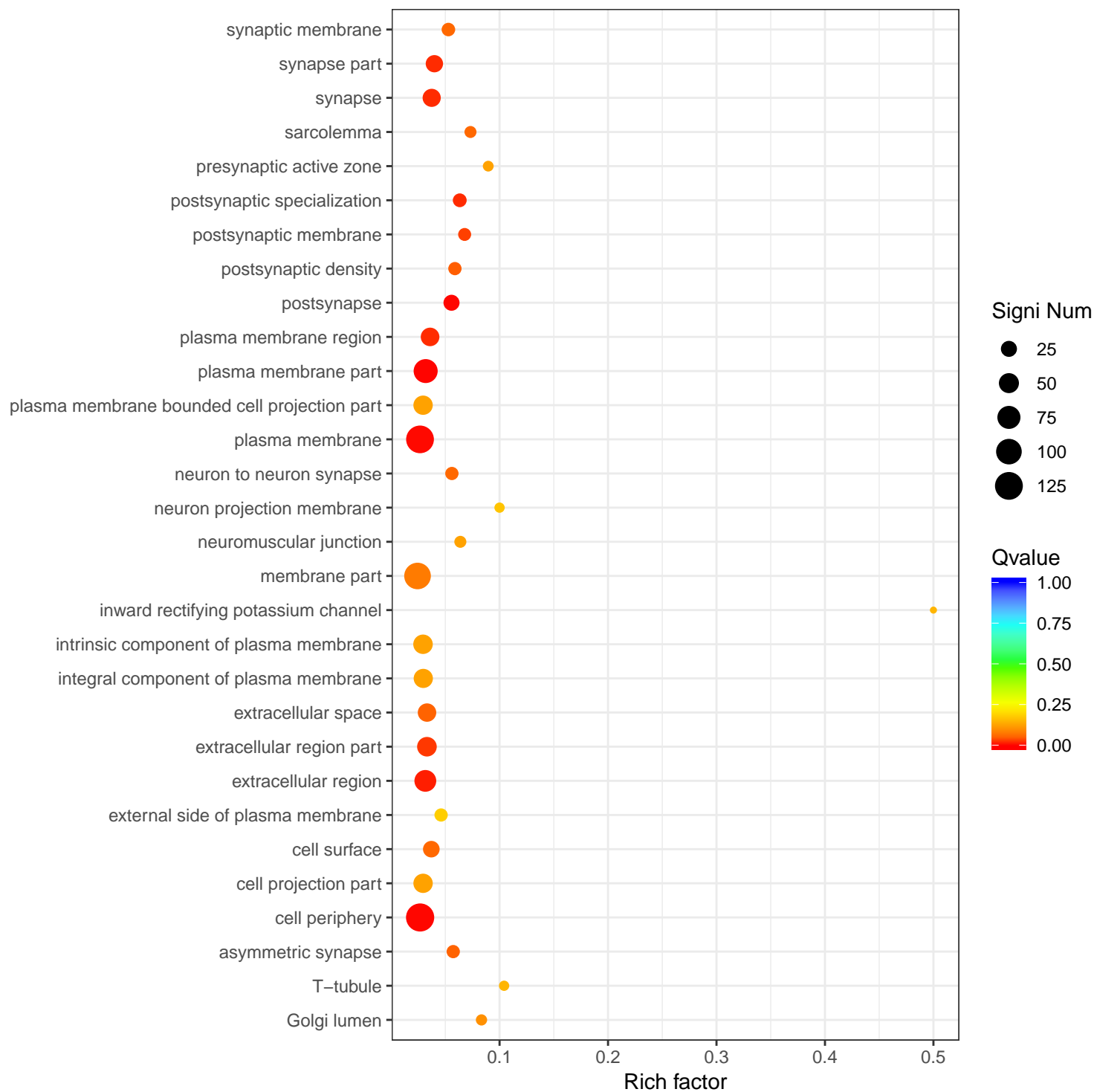

Supplement: S1 File — (ZIP) [file pone.0311069.s002.zip › S2_File/2_GO_enrichment/scatter/union.DE_gene.subcluster1_CC_enrichment_scatter.pdf]

# GO Enrichment

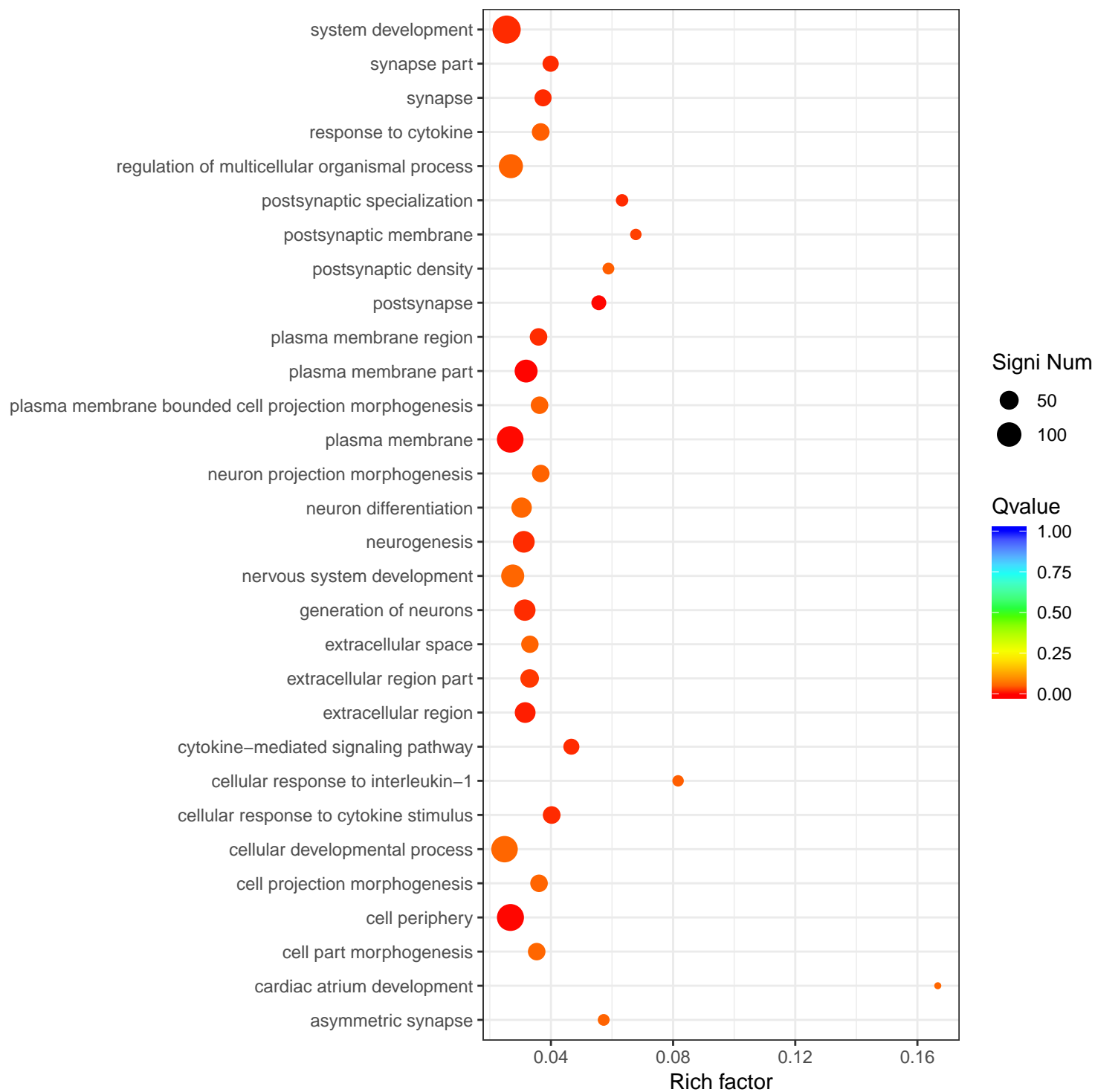

Supplement: S1 File — (ZIP) [file pone.0311069.s002.zip › S2_File/2_GO_enrichment/scatter/union.DE_gene.subcluster1_GO_enrichment_scatter.pdf]

# MF Enrichment

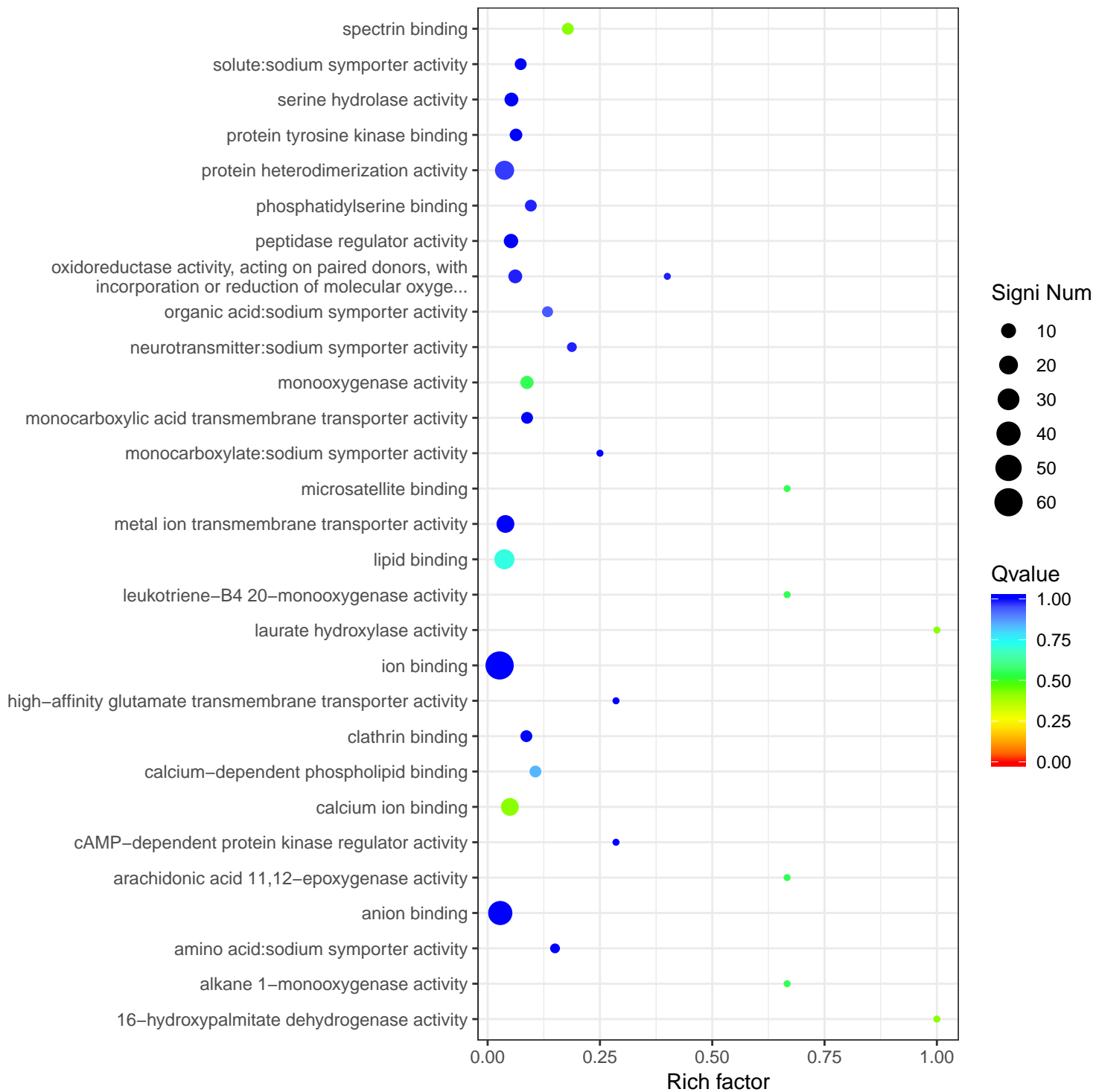

Supplement: S1 File — (ZIP) [file pone.0311069.s002.zip › S2_File/2_GO_enrichment/scatter/union.DE_gene.subcluster1_MF_enrichment_scatter.pdf]

# KEGG Pathway Classification

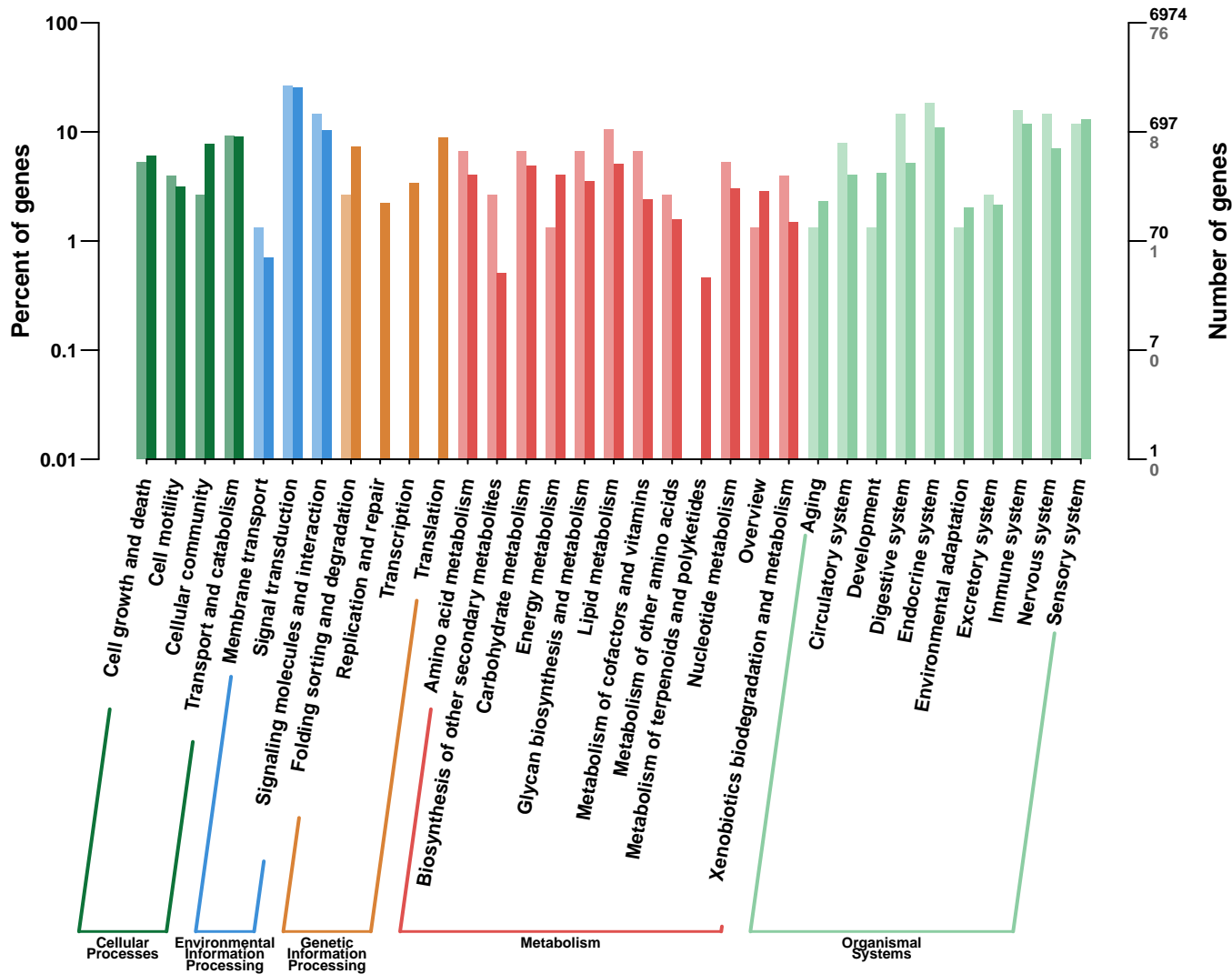

Supplement: S1 File — (ZIP) [file pone.0311069.s002.zip › S2_File/3_KEGG_enrichment/barplot/B_vs_A.down_pathway_categorie.pdf]

# KEGG Pathway Classification

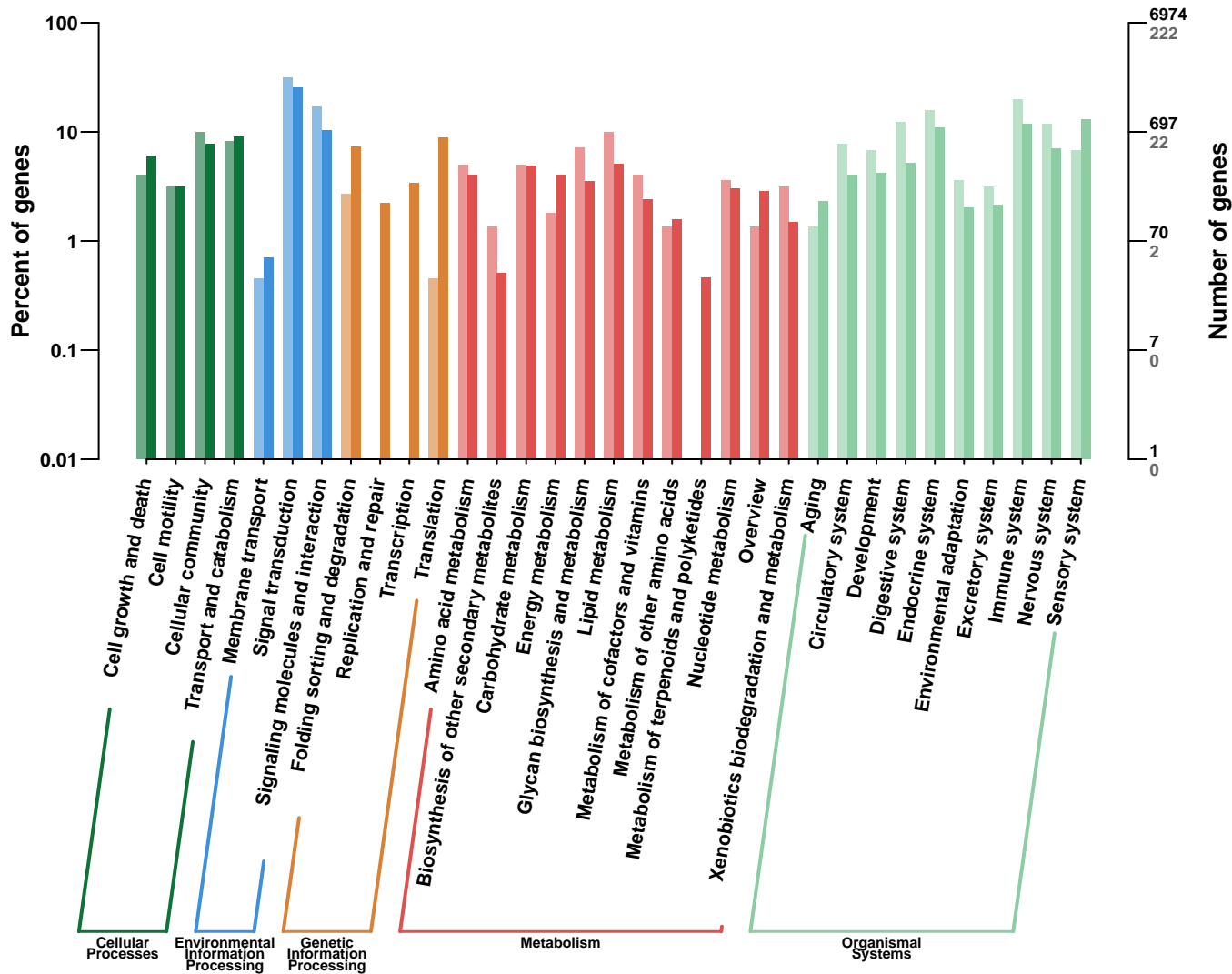

Supplement: S1 File — (ZIP) [file pone.0311069.s002.zip › S2_File/3_KEGG_enrichment/barplot/B_vs_A.sign_pathway_categorie.pdf]

# KEGG Pathway Classification

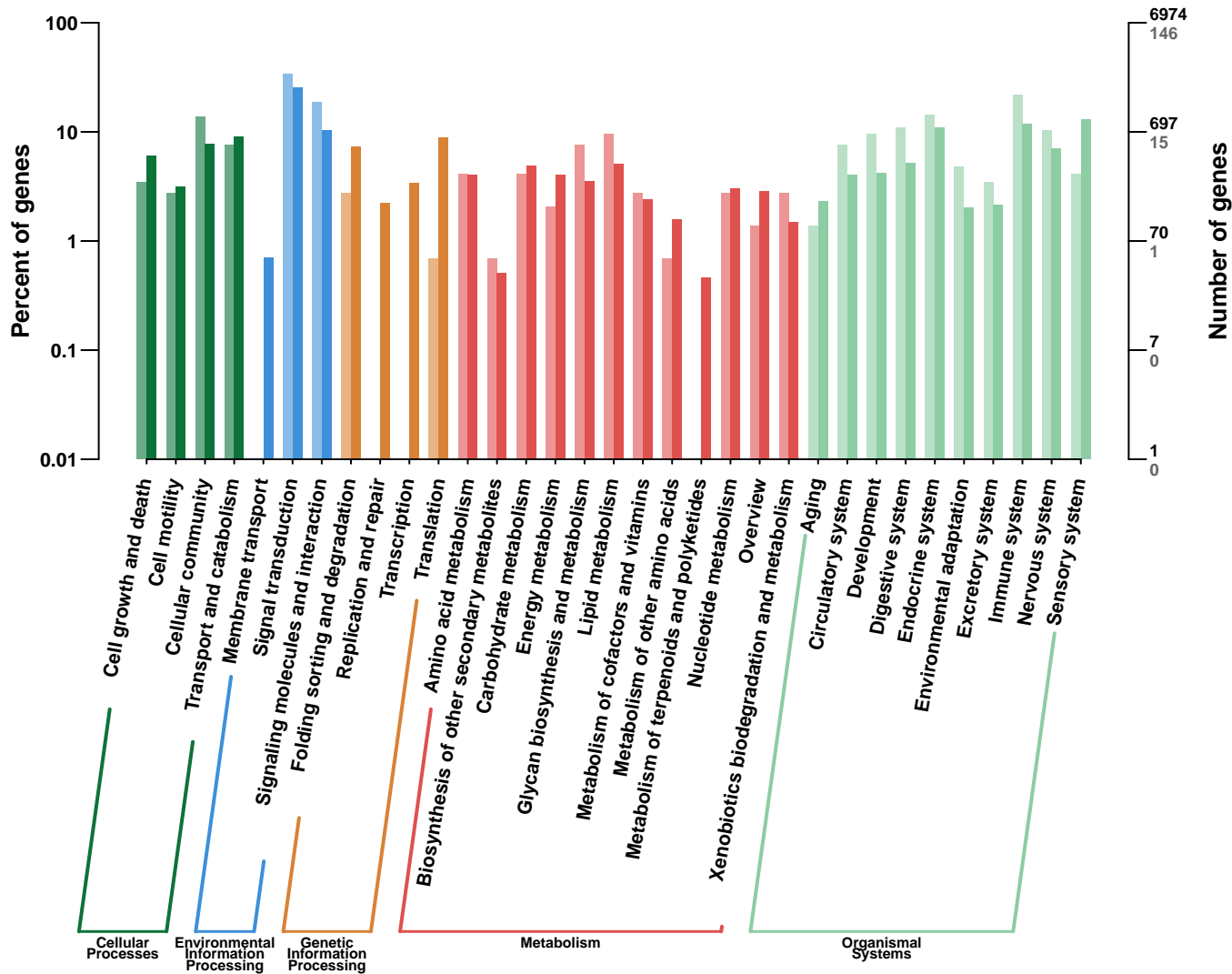

Supplement: S1 File — (ZIP) [file pone.0311069.s002.zip › S2_File/3_KEGG_enrichment/barplot/B_vs_A.up_pathway_categorie.pdf]

# KEGG Pathway Classification

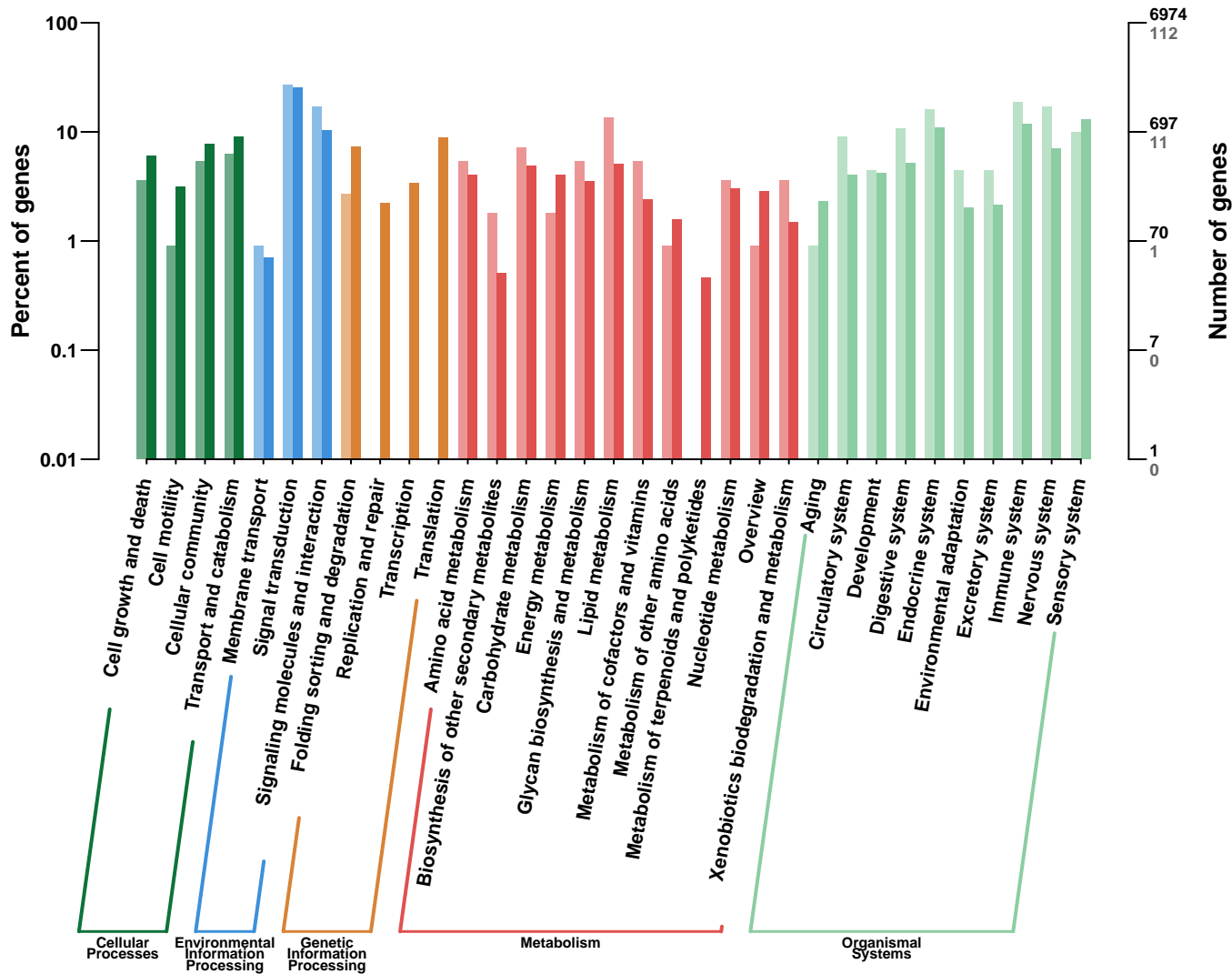

Supplement: S1 File — (ZIP) [file pone.0311069.s002.zip › S2_File/3_KEGG_enrichment/barplot/union.DE_gene.subcluster1_pathway_categorie.pdf]

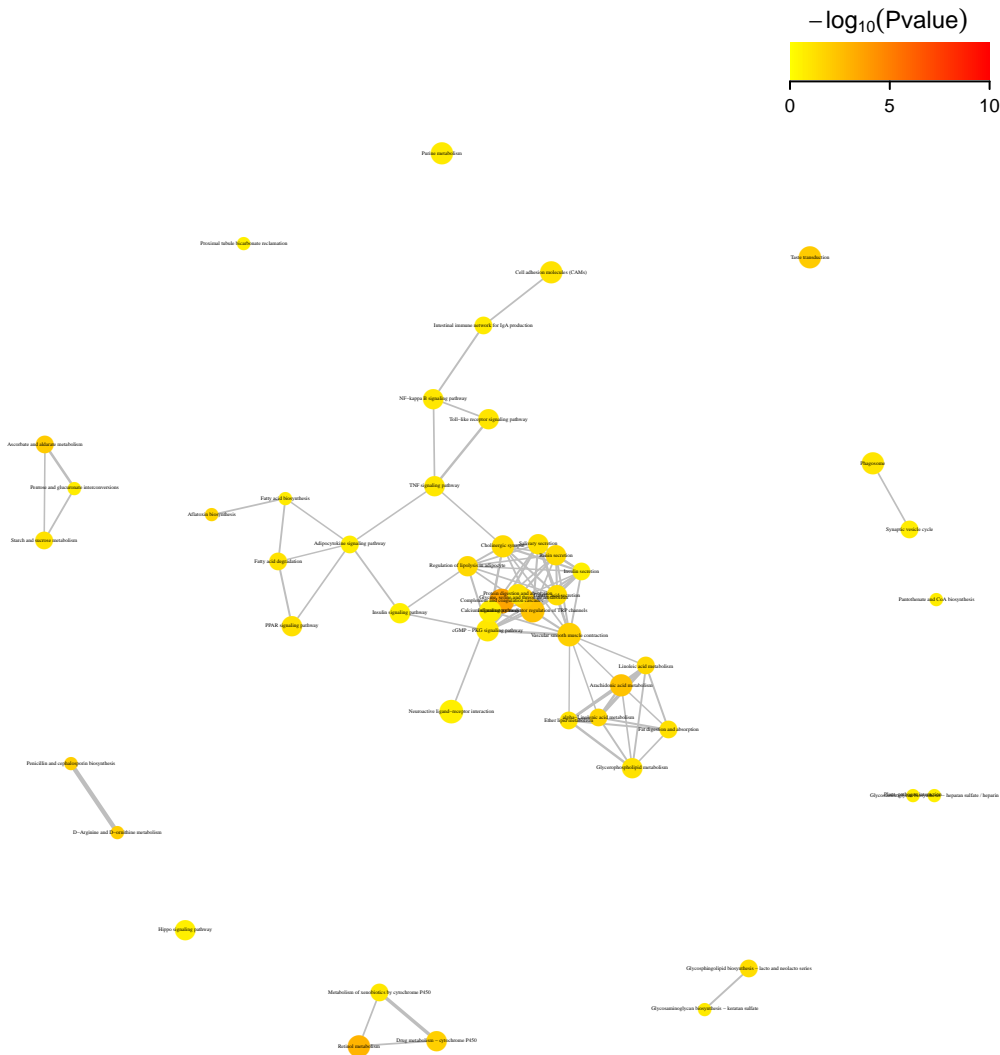

Supplement: S1 File — (ZIP) [file pone.0311069.s002.zip › S2_File/3_KEGG_enrichment/enrichmap/B_vs_A.down_KEGG_enrichment_map.pdf]

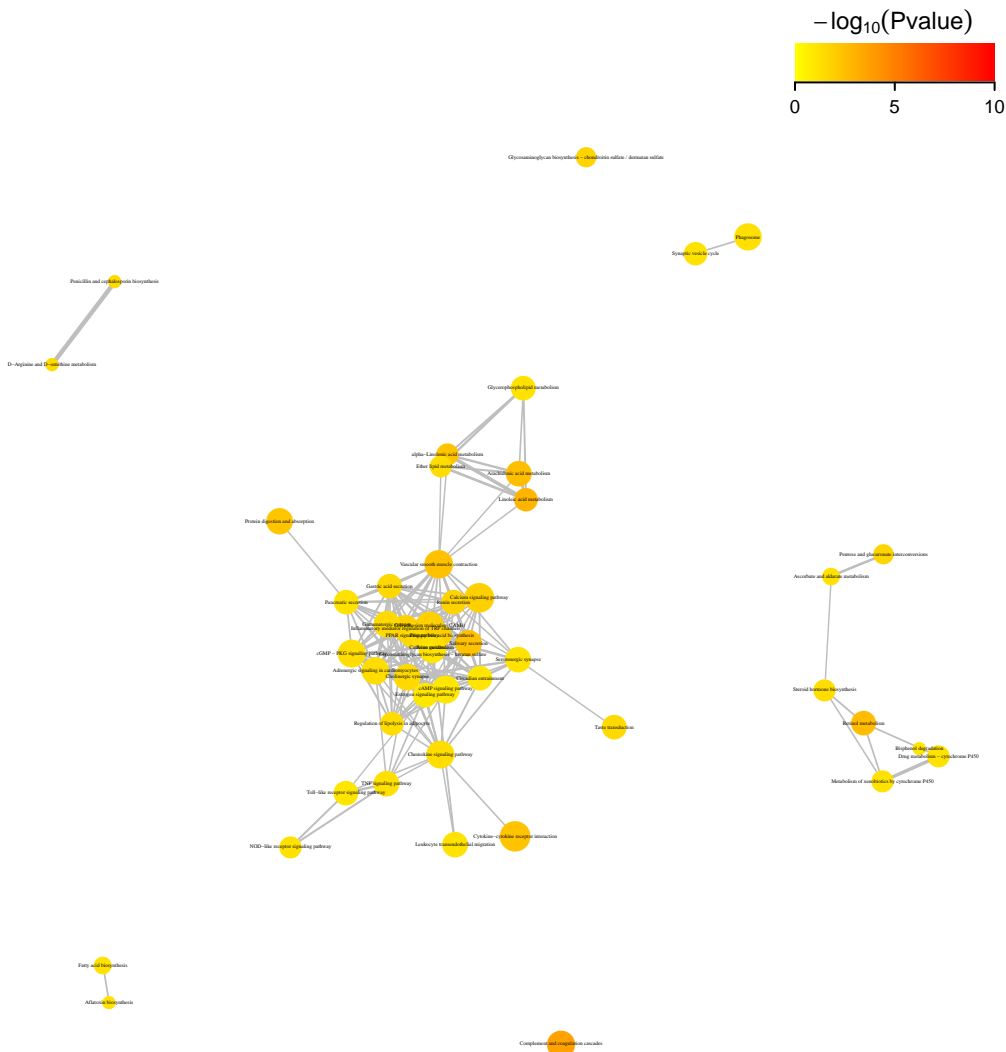

Supplement: S1 File — (ZIP) [file pone.0311069.s002.zip › S2_File/3_KEGG_enrichment/enrichmap/B_vs_A.sign_KEGG_enrichment_map.pdf]

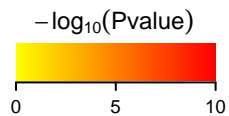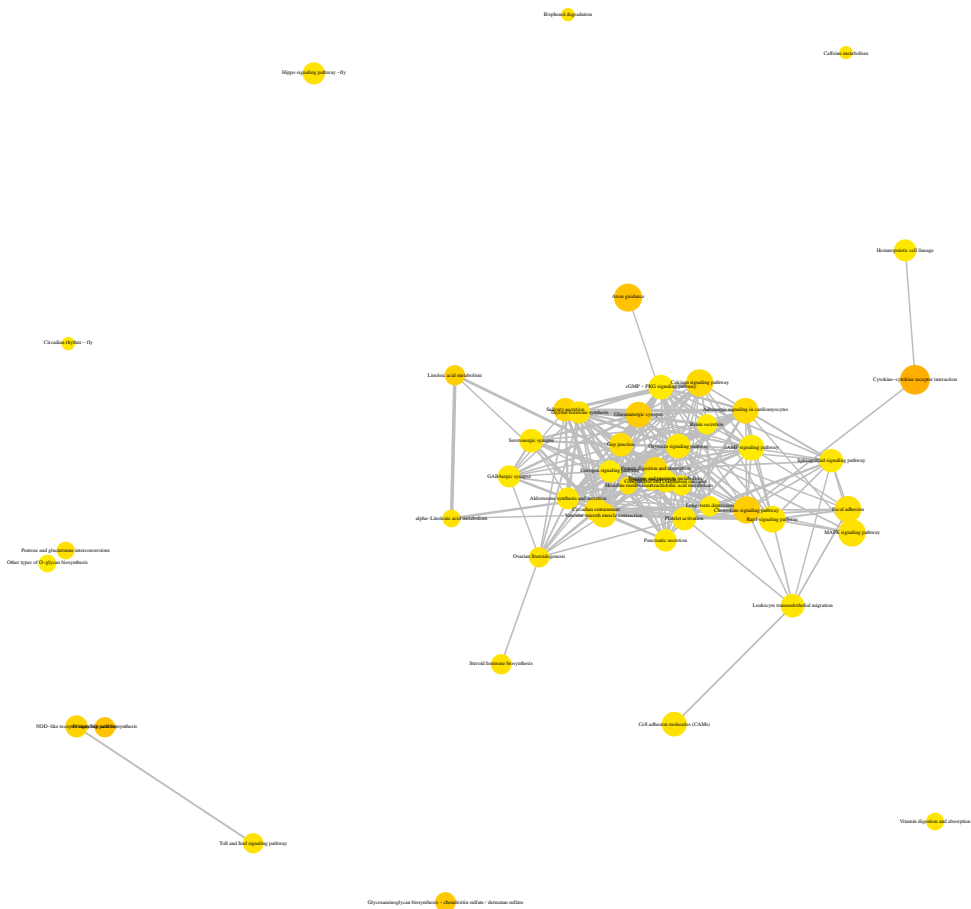

Supplement: S1 File — (ZIP) [file pone.0311069.s002.zip › S2_File/3_KEGG_enrichment/enrichmap/B_vs_A.up_KEGG_enrichment_map.pdf]

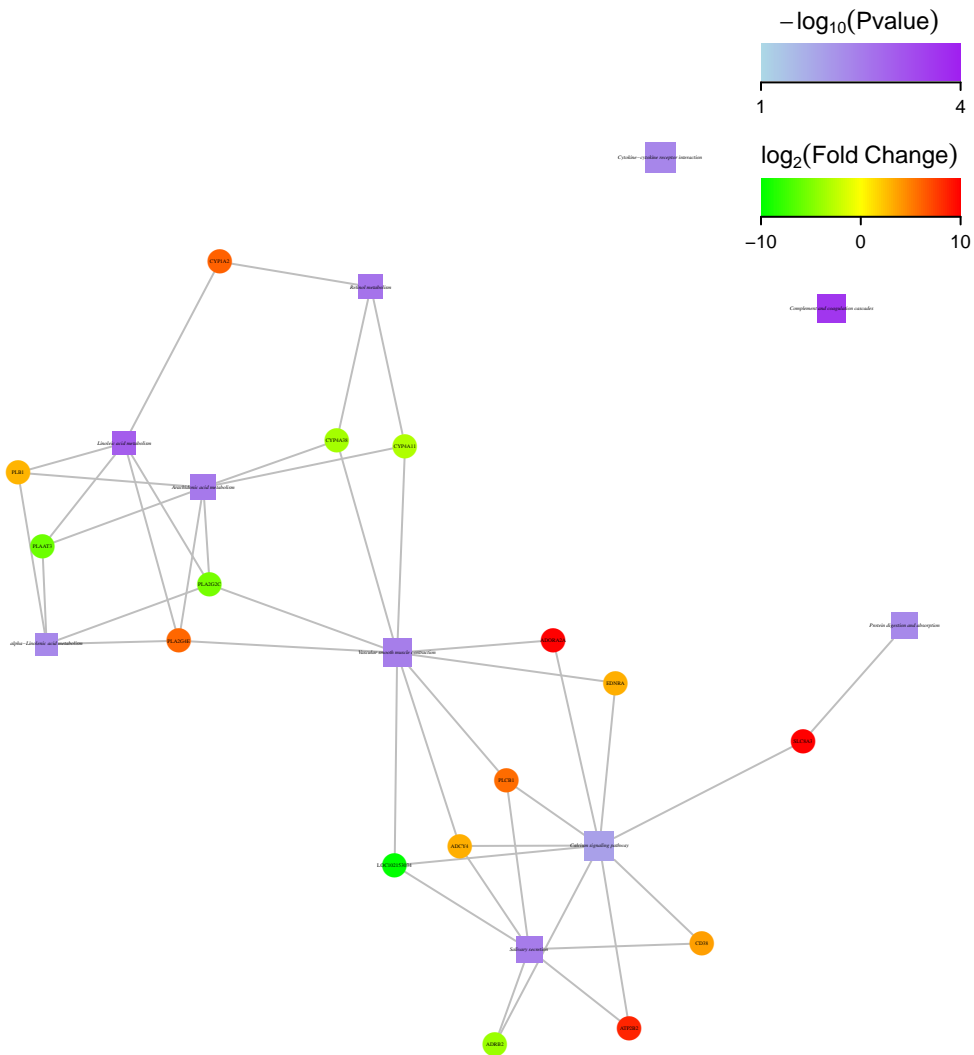

Supplement: S1 File — (ZIP) [file pone.0311069.s002.zip › S2_File/3_KEGG_enrichment/network/B_vs_A.sign_KEGG_enrichment_network.pdf]

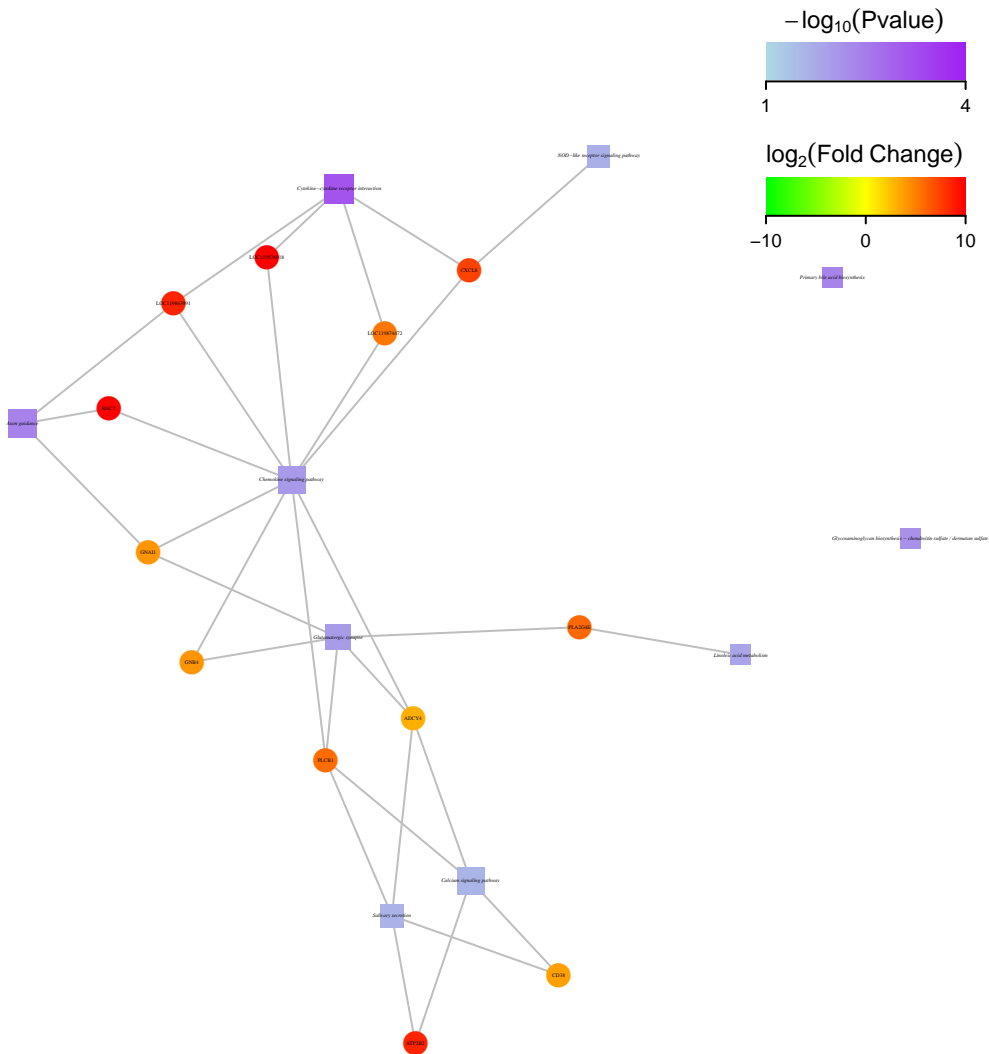

Supplement: S1 File — (ZIP) [file pone.0311069.s002.zip › S2_File/3_KEGG_enrichment/network/B_vs_A.up_KEGG_enrichment_network.pdf]

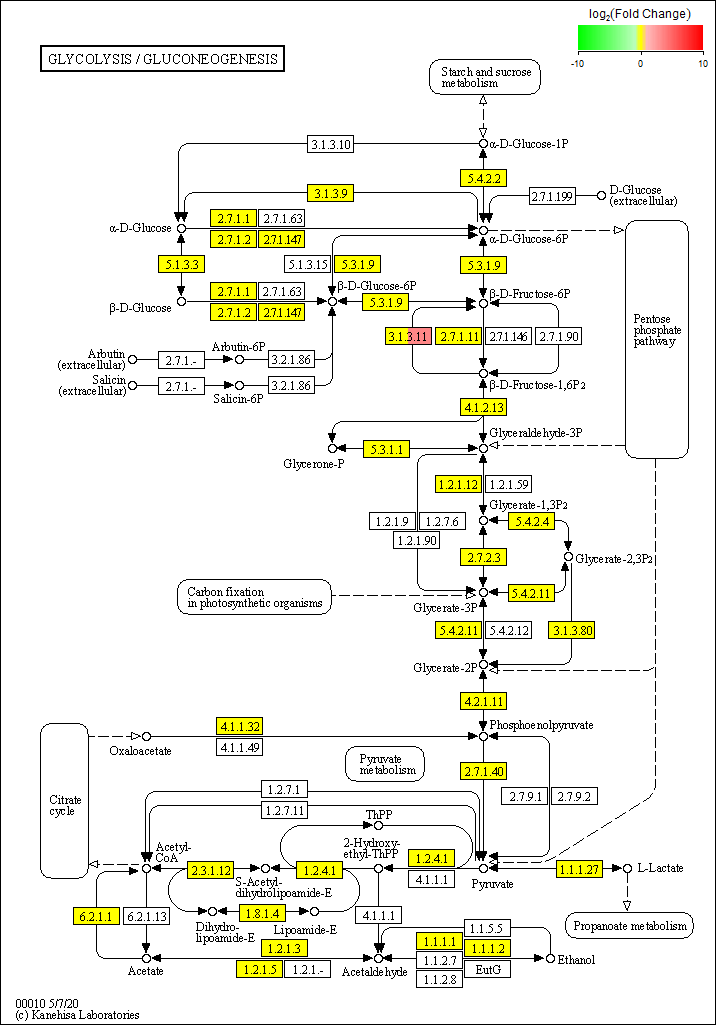

Supplement: S1 File — (ZIP) [file pone.0311069.s002.zip › S2_File/3_KEGG_enrichment/pathwaymap/B_vs_A.maps/map00010.png]

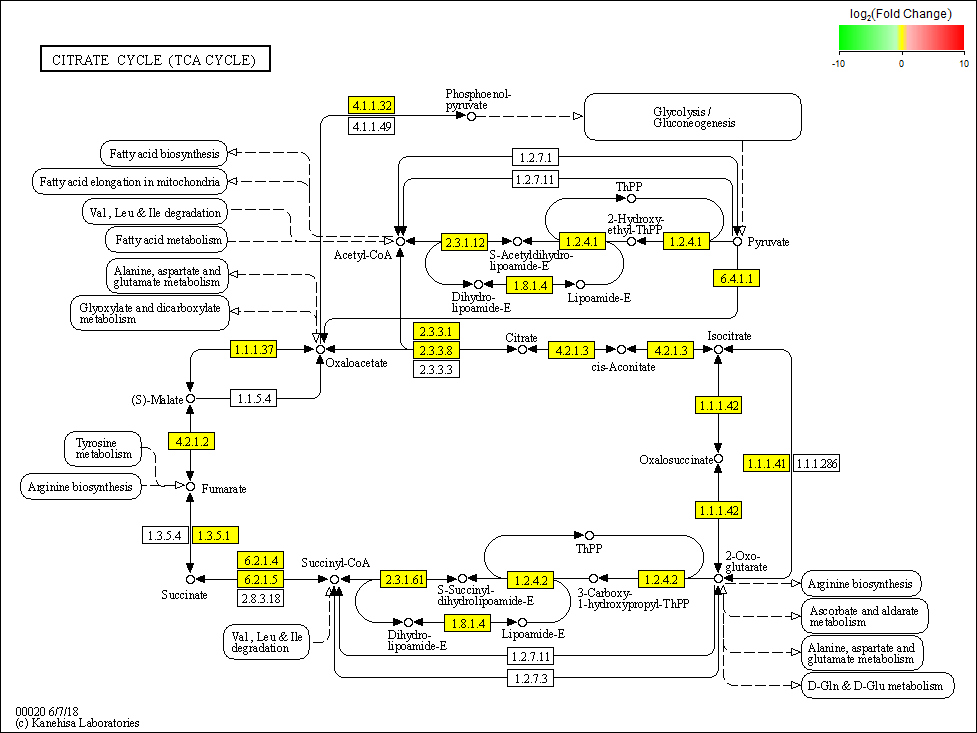

Supplement: S1 File — (ZIP) [file pone.0311069.s002.zip › S2_File/3_KEGG_enrichment/pathwaymap/B_vs_A.maps/map00020.png]

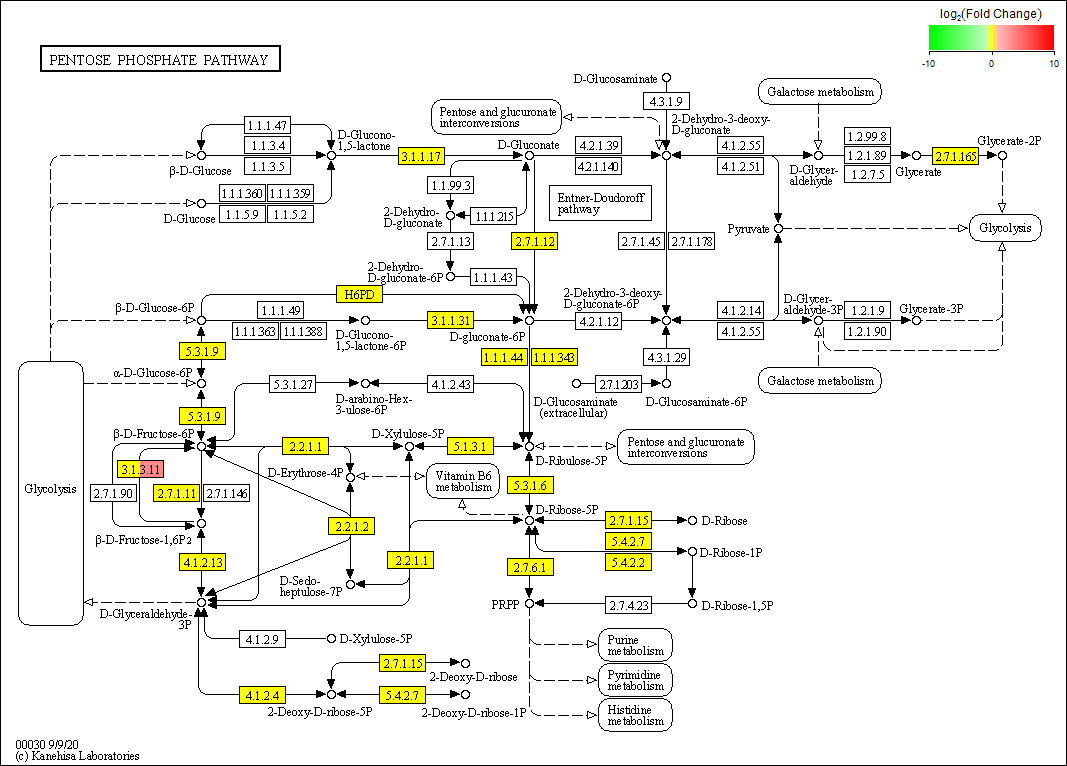

Supplement: S1 File — (ZIP) [file pone.0311069.s002.zip › S2_File/3_KEGG_enrichment/pathwaymap/B_vs_A.maps/map00030.png]

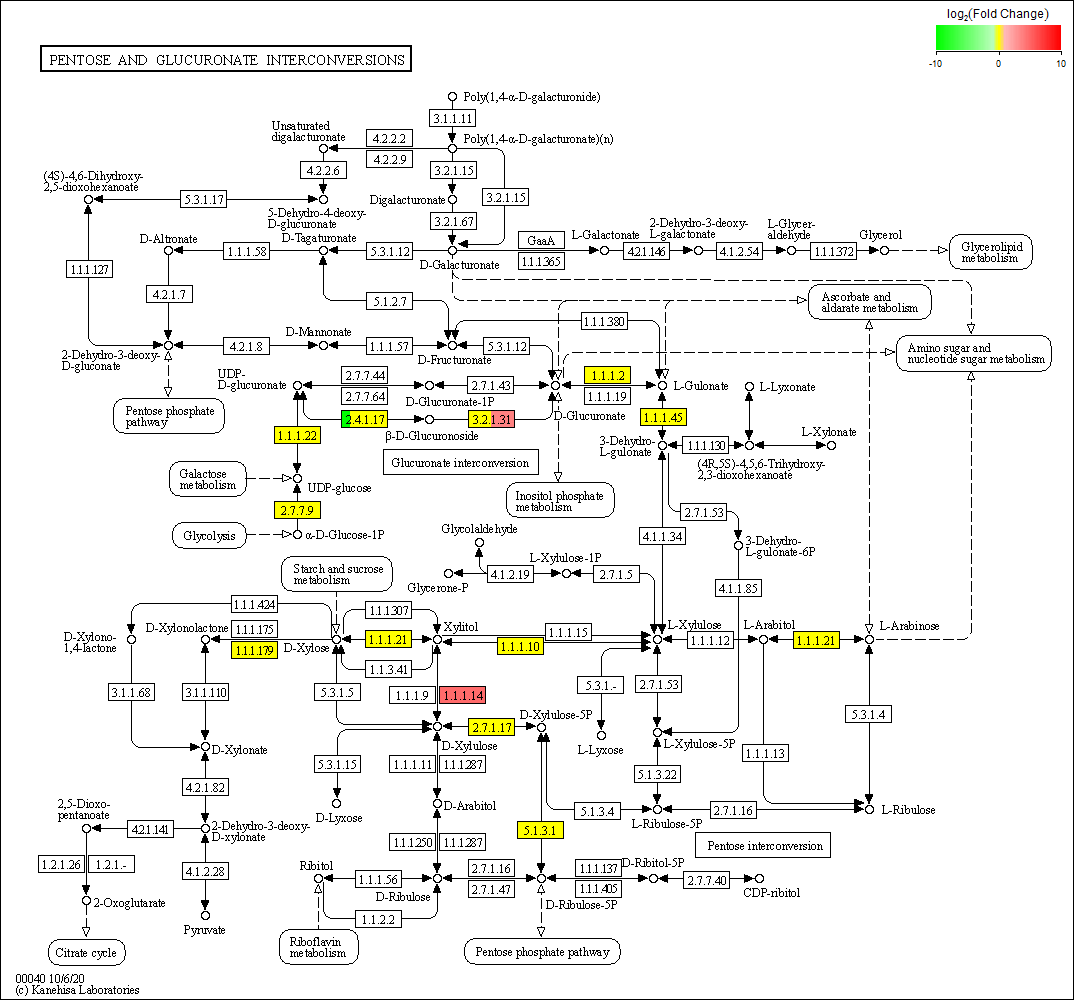

Supplement: S1 File — (ZIP) [file pone.0311069.s002.zip › S2_File/3_KEGG_enrichment/pathwaymap/B_vs_A.maps/map00040.png]

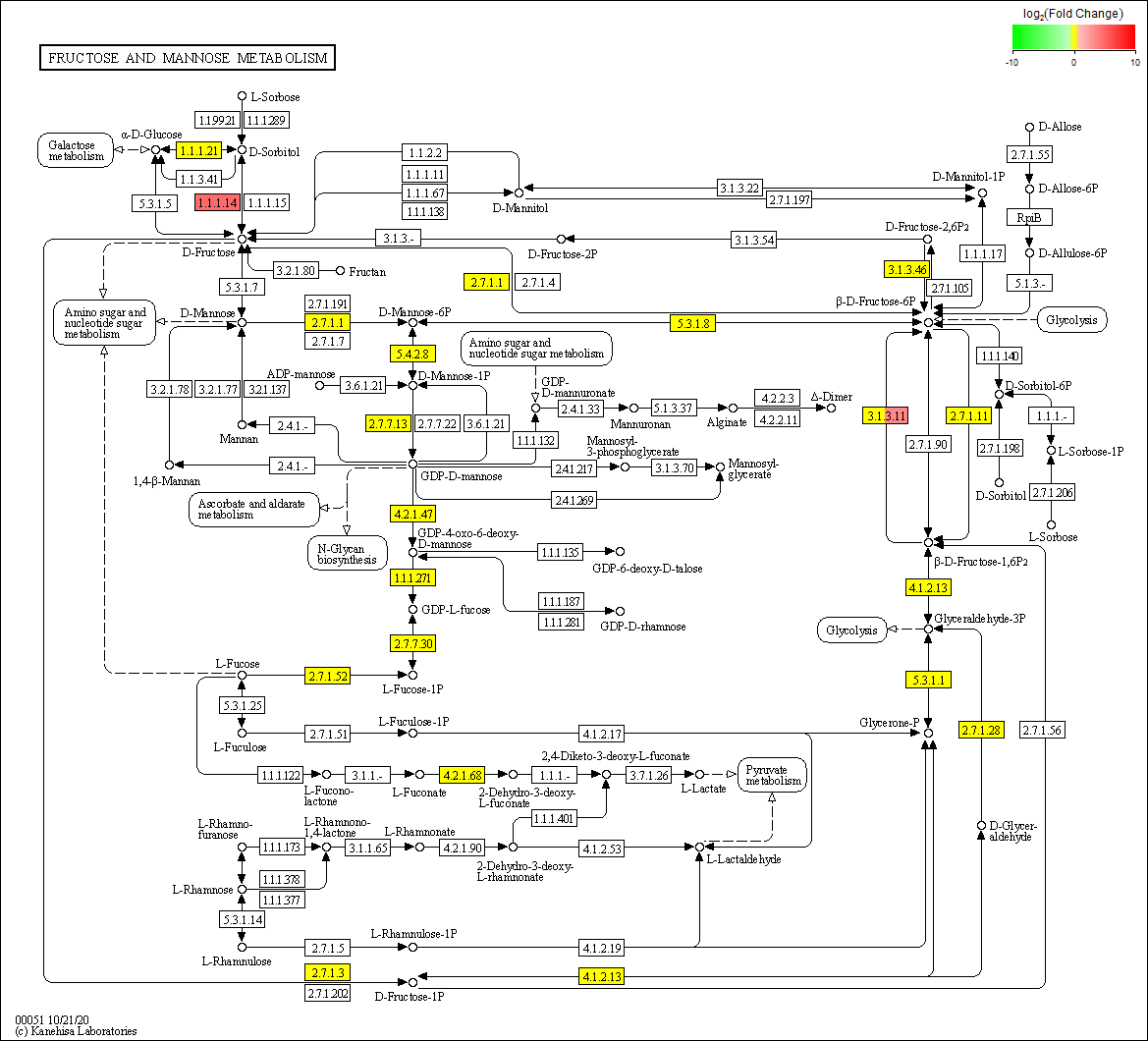

Supplement: S1 File — (ZIP) [file pone.0311069.s002.zip › S2_File/3_KEGG_enrichment/pathwaymap/B_vs_A.maps/map00051.png]

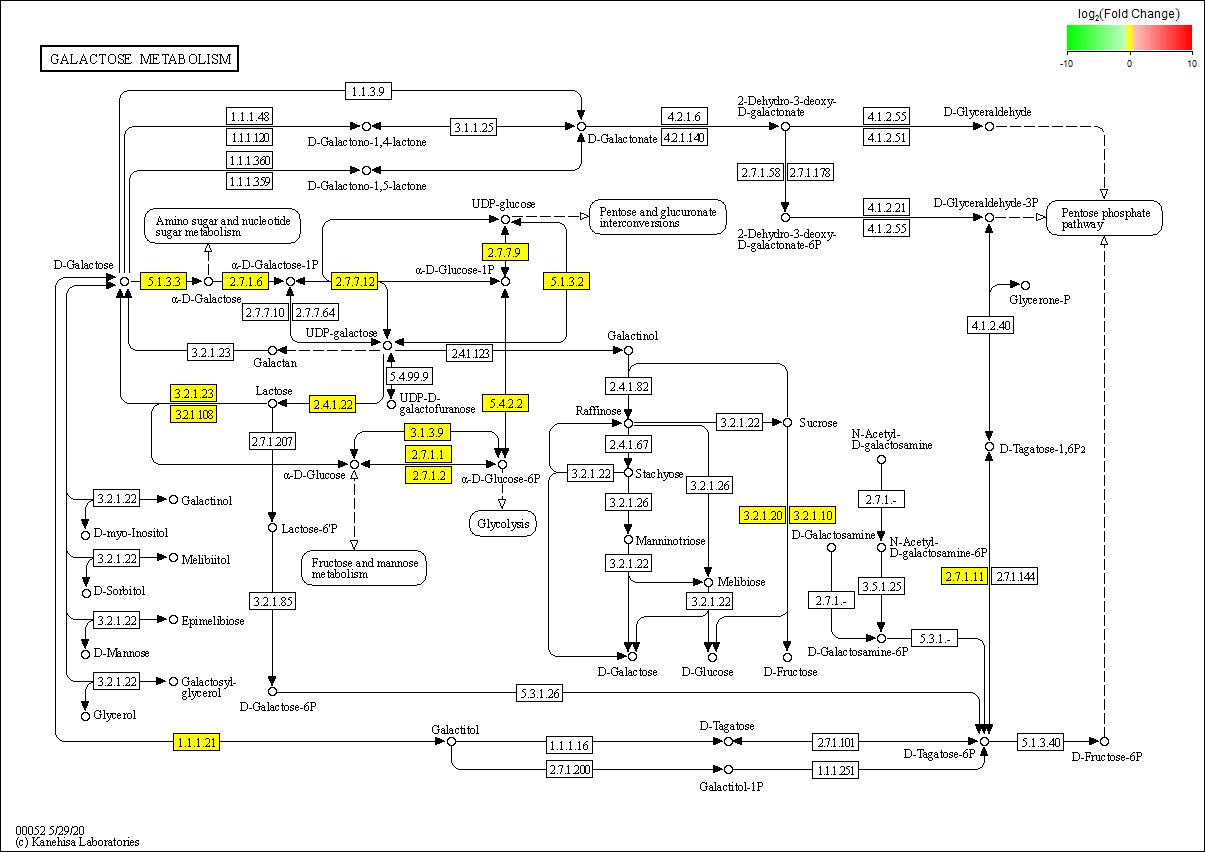

Supplement: S1 File — (ZIP) [file pone.0311069.s002.zip › S2_File/3_KEGG_enrichment/pathwaymap/B_vs_A.maps/map00052.png]

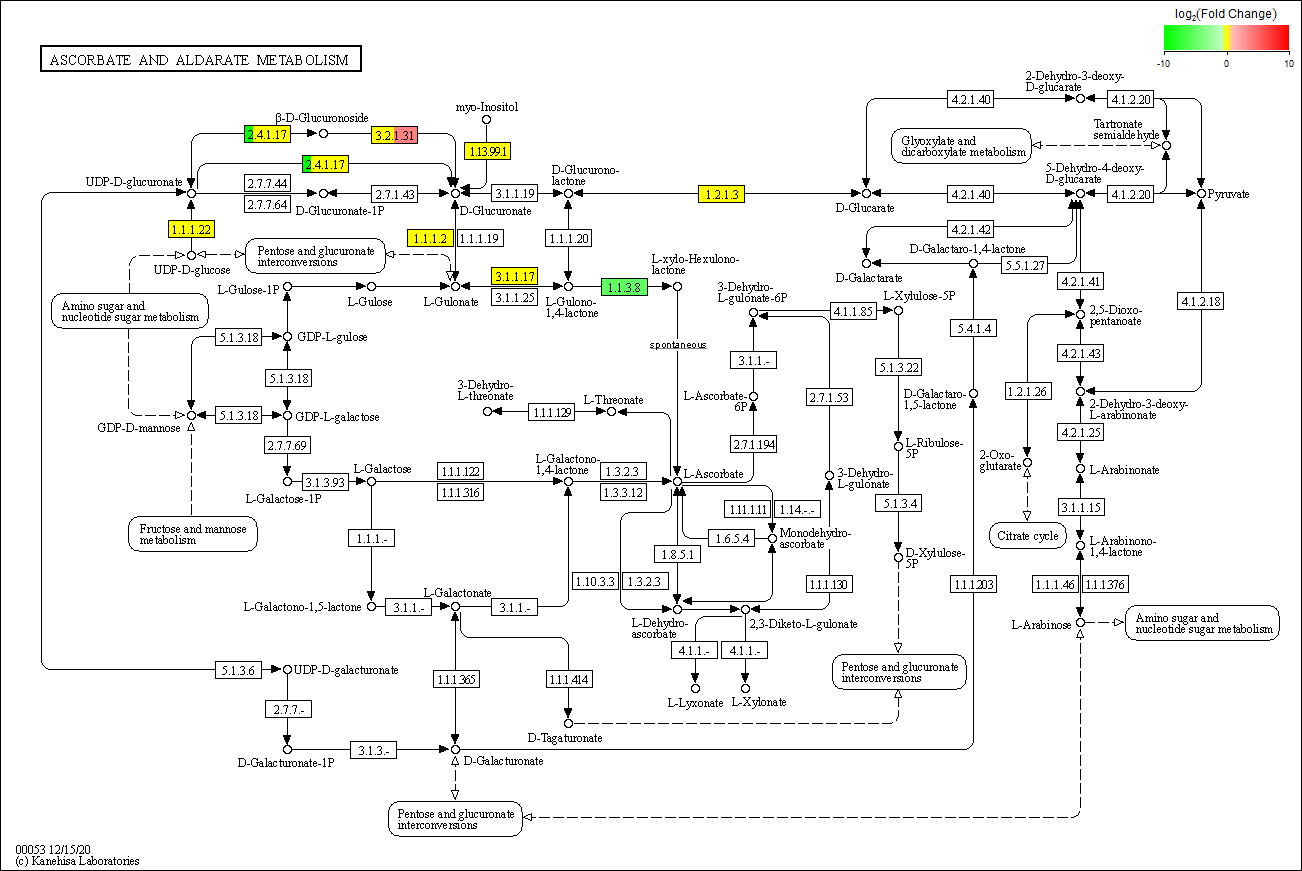

Supplement: S1 File — (ZIP) [file pone.0311069.s002.zip › S2_File/3_KEGG_enrichment/pathwaymap/B_vs_A.maps/map00053.png]

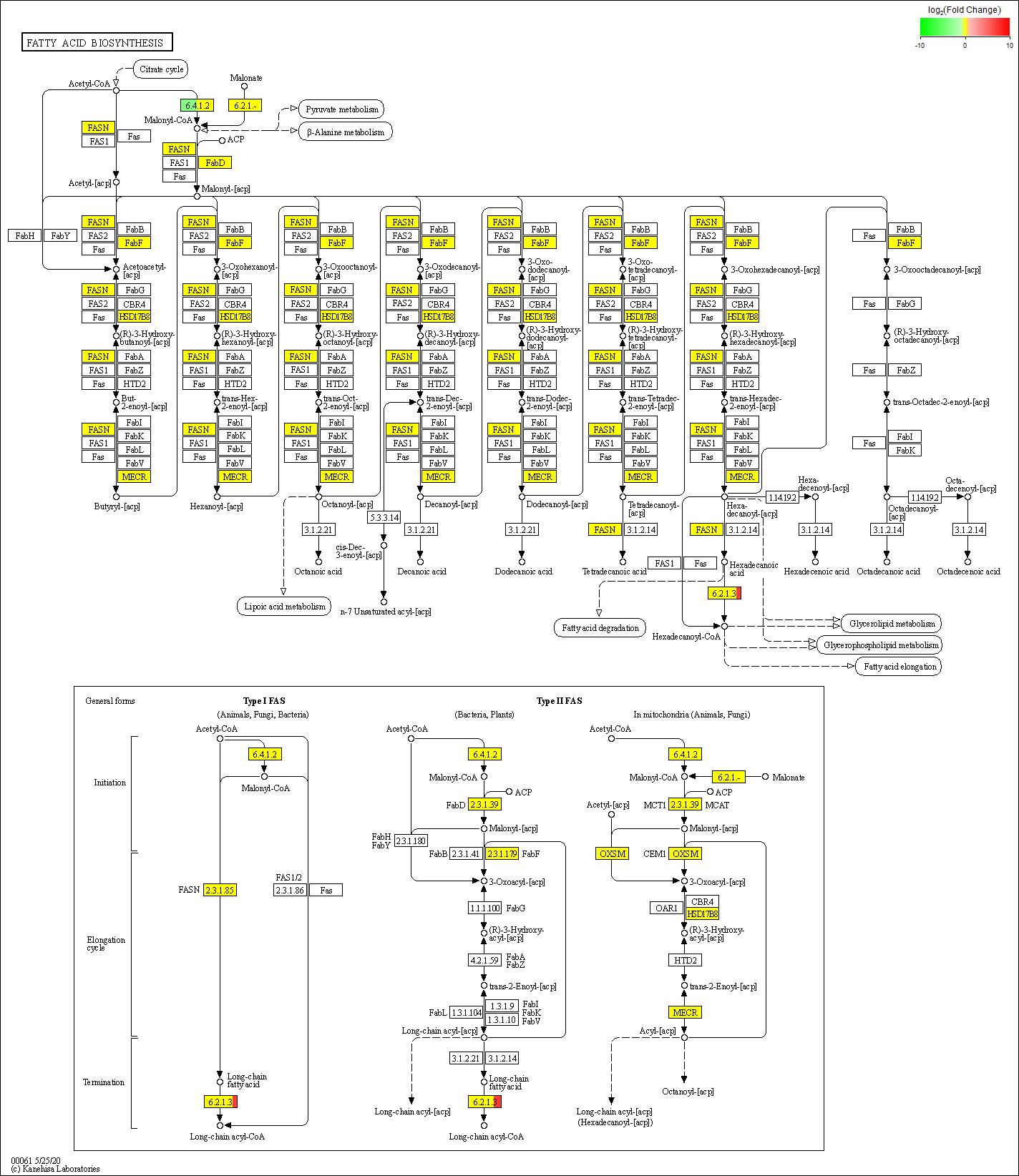

Supplement: S1 File — (ZIP) [file pone.0311069.s002.zip › S2_File/3_KEGG_enrichment/pathwaymap/B_vs_A.maps/map00061.png]

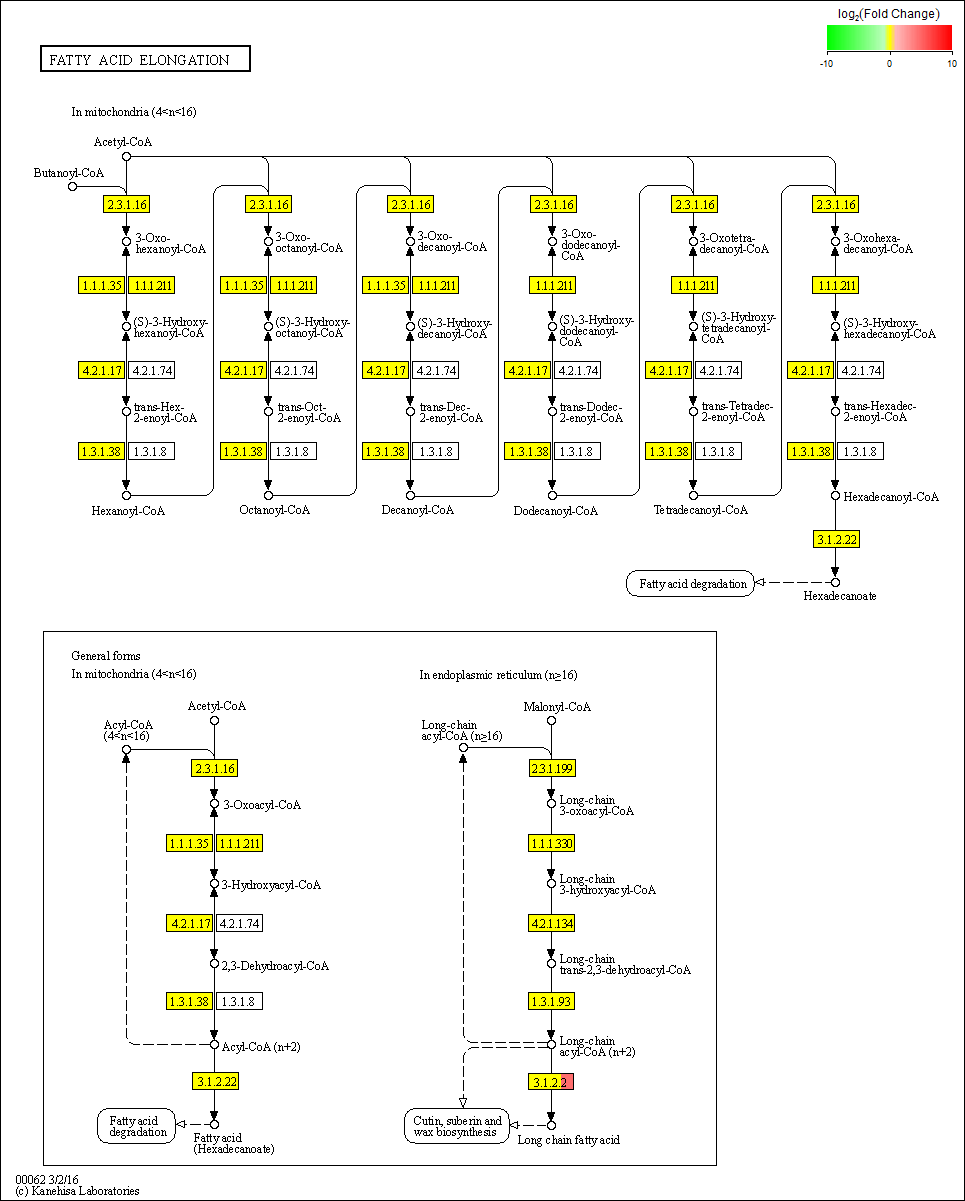

Supplement: S1 File — (ZIP) [file pone.0311069.s002.zip › S2_File/3_KEGG_enrichment/pathwaymap/B_vs_A.maps/map00062.png]

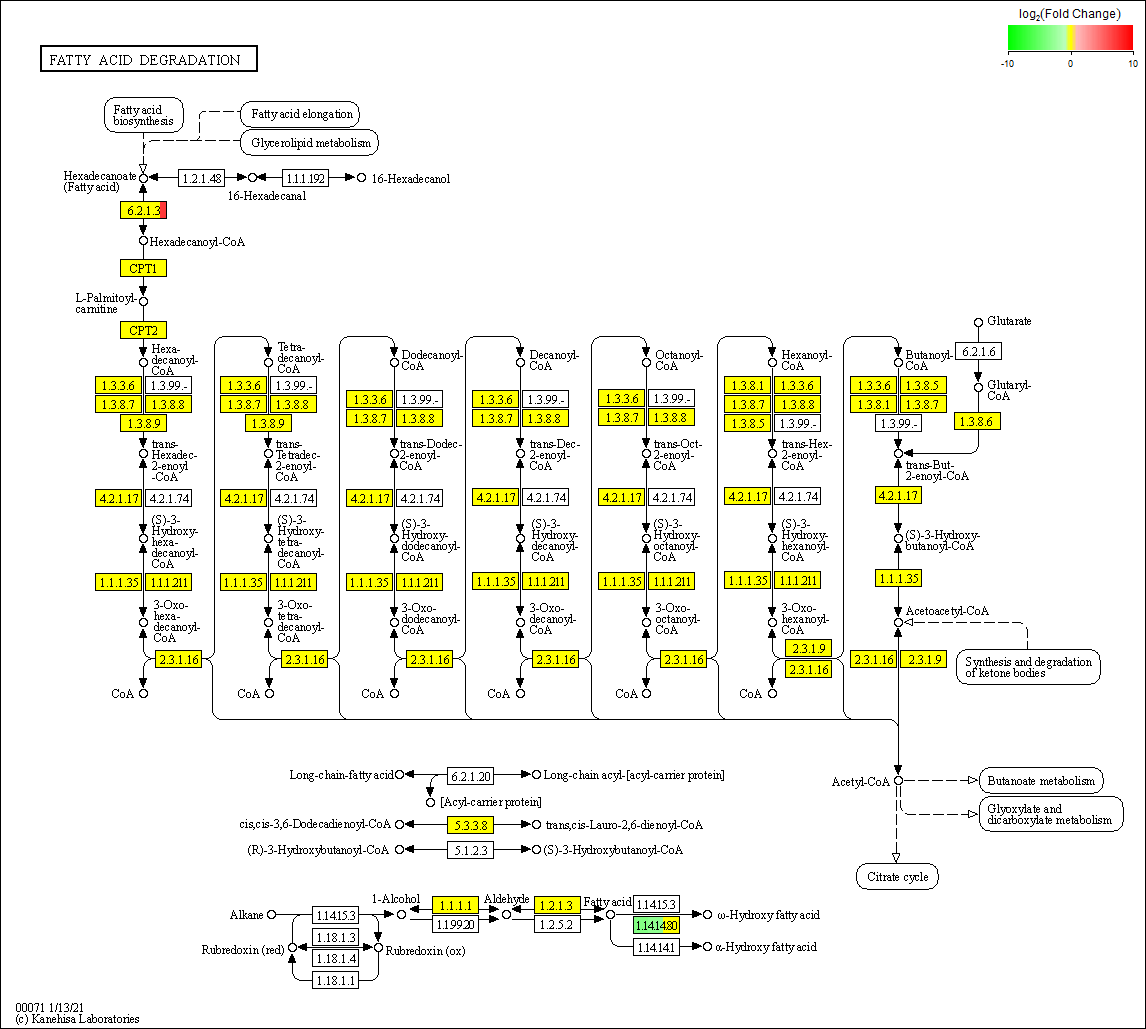

Supplement: S1 File — (ZIP) [file pone.0311069.s002.zip › S2_File/3_KEGG_enrichment/pathwaymap/B_vs_A.maps/map00071.png]

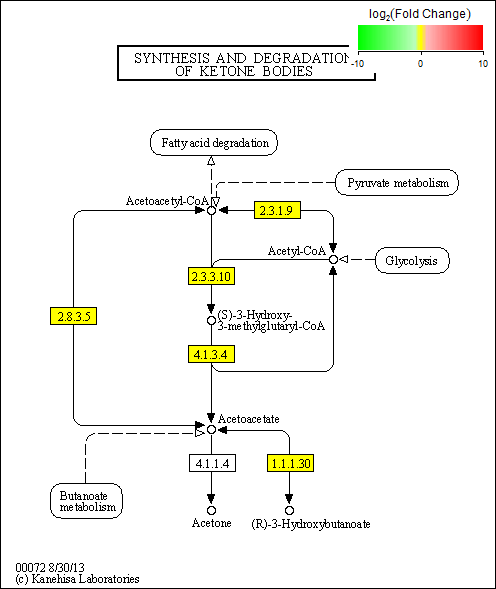

Supplement: S1 File — (ZIP) [file pone.0311069.s002.zip › S2_File/3_KEGG_enrichment/pathwaymap/B_vs_A.maps/map00072.png]

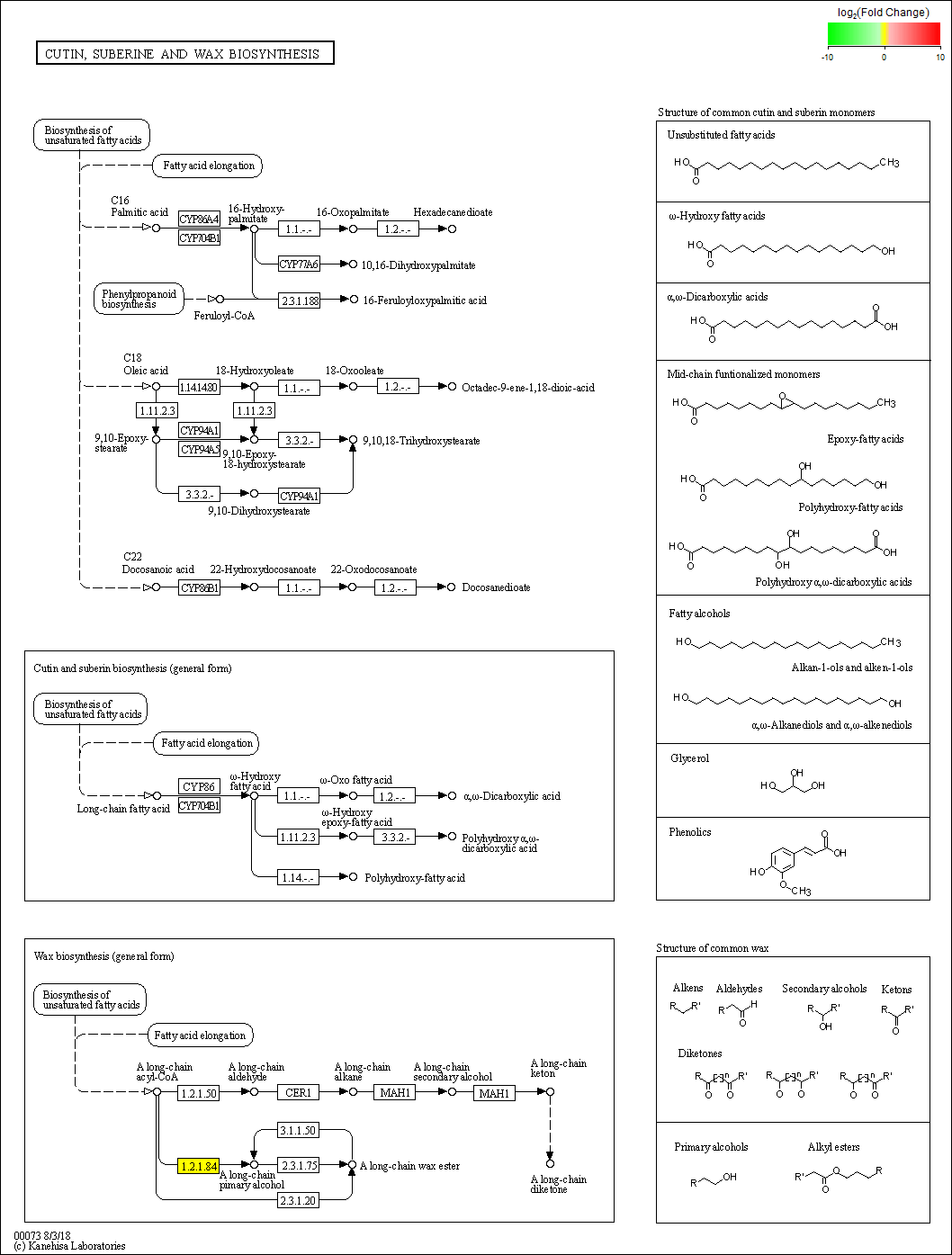

Supplement: S1 File — (ZIP) [file pone.0311069.s002.zip › S2_File/3_KEGG_enrichment/pathwaymap/B_vs_A.maps/map00073.png]
