# Supplementary material for: Tumorigenicity decrease in Bcl-xL deficient MDCK cells ensuring the safety for influenza vaccine production
Source: PLoS One. 2024 Dec 16;19(12):e0311069. doi: 10.1371/journal.pone.0311069 (PMC11649150; doi:10.1371/journal.pone.0311069)

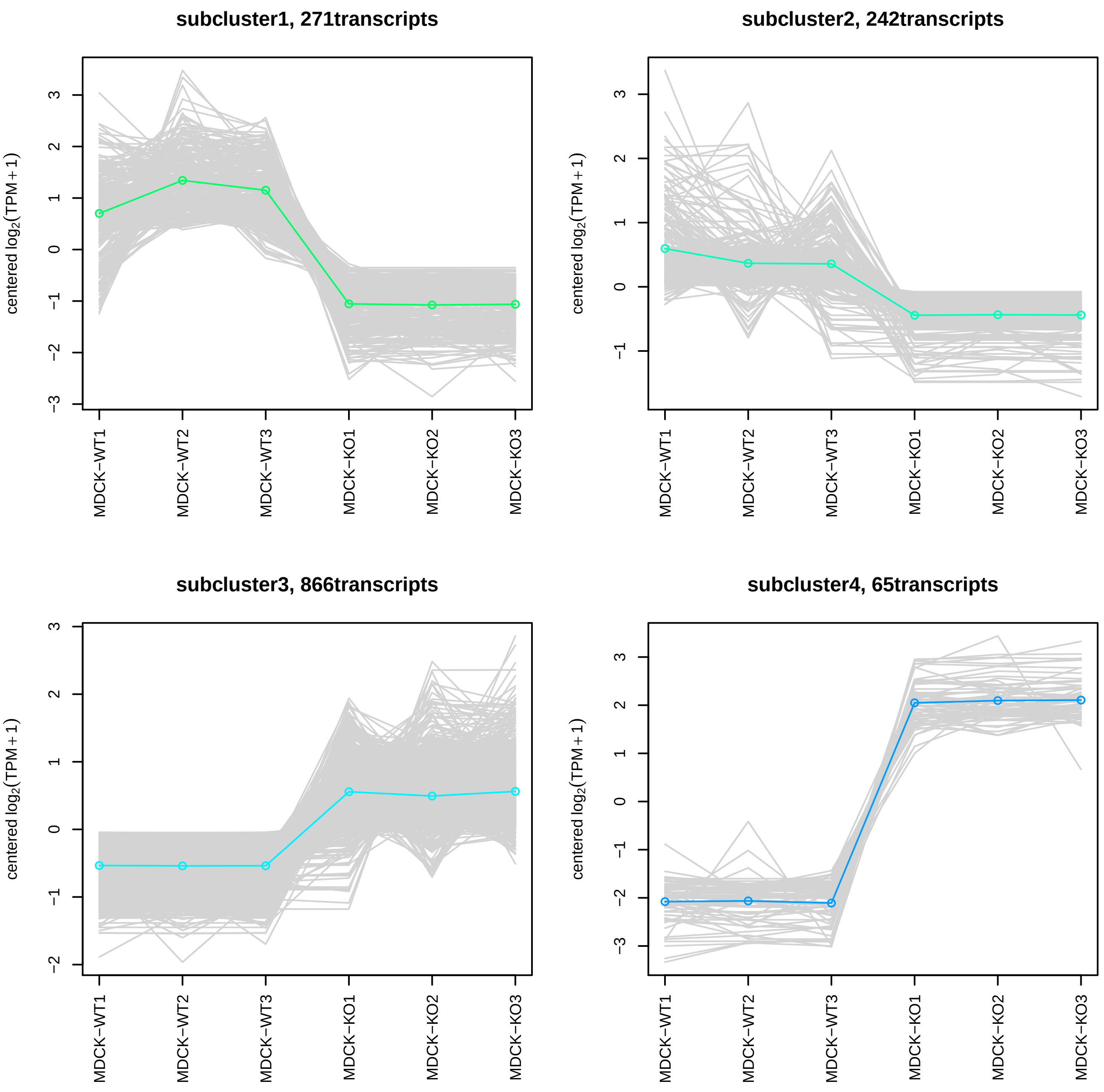

subcluster1, 271transcripts
subcluster2, 242transcripts
subcluster3, 866transcripts
subcluster4, 65transcripts
centered log2(TPM+1)
MDCK−WT1
MDCK−WT2
MDCK−WT3
MDCK−KO1
MDCK−KO2
MDCK−KO3

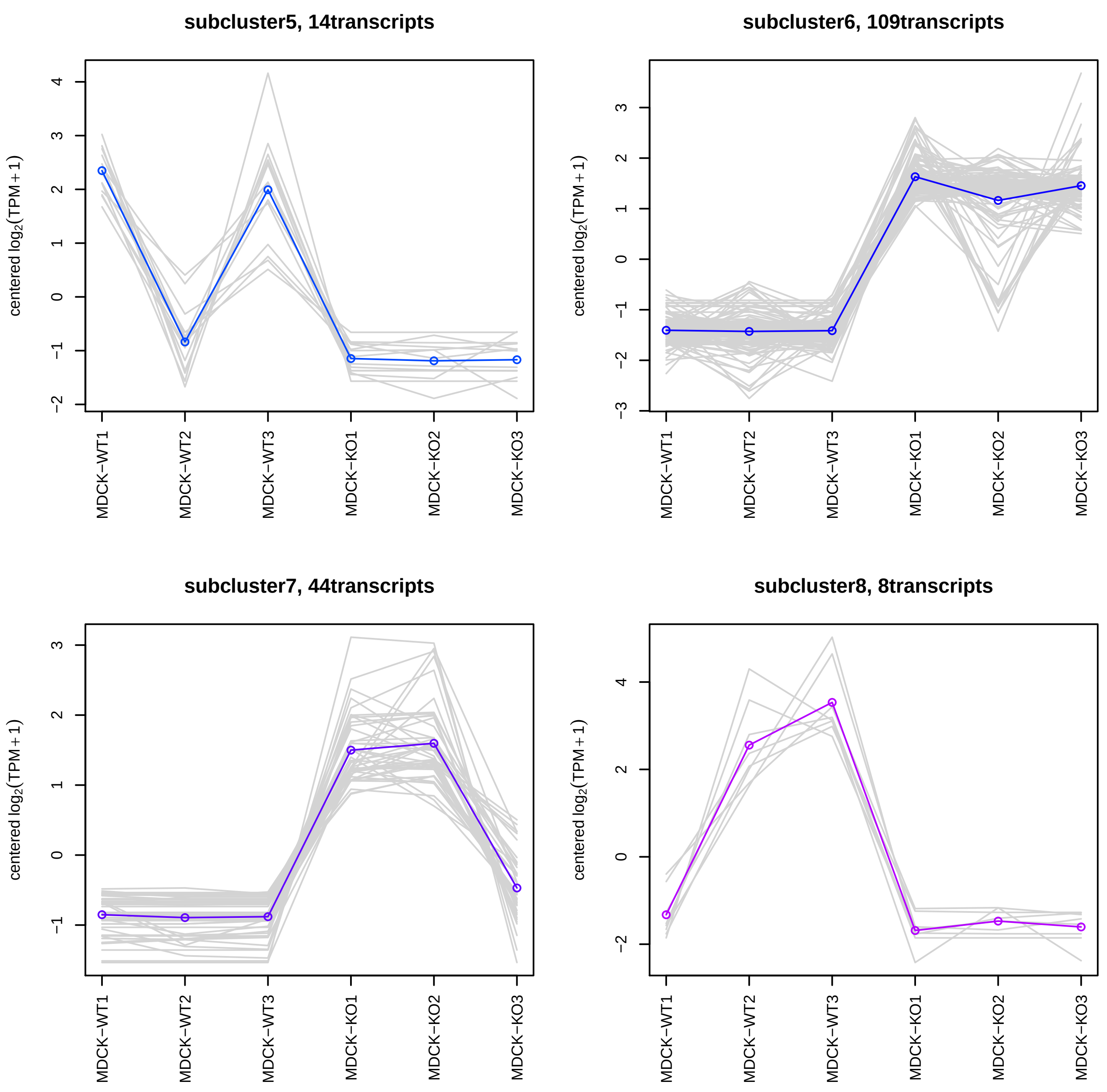
subcluster5, 14transcripts
centered log2(TPM+1)
−2
−1
0
1
2
3
4
MDCK−WT1
MDCK−WT2
MDCK−WT3
MDCK−KO1
MDCK−KO2
MDCK−KO3
subcluster6, 109transcripts
centered log2(TPM+1)
−3
−2
−1
0
1
2
3
MDCK−WT1
MDCK−WT2
MDCK−WT3
MDCK−KO1
MDCK−KO2
MDCK−KO3
subcluster7, 44transcripts
centered log2(TPM+1)
−1
0
1
2
3
MDCK−WT1
MDCK−WT2
MDCK−WT3
MDCK−KO1
MDCK−KO2
MDCK−KO3
subcluster8, 8transcripts
centered log2(TPM+1)
−2
0
2
4
MDCK−WT1
MDCK−WT2
MDCK−WT3
MDCK−KO1
MDCK−KO2
MDCK−KO3

subcluster9, 13transcripts

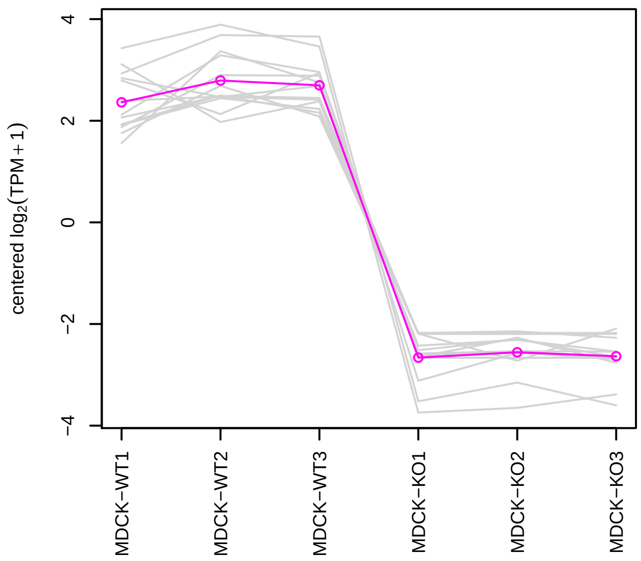

subcluster10, 2transcripts

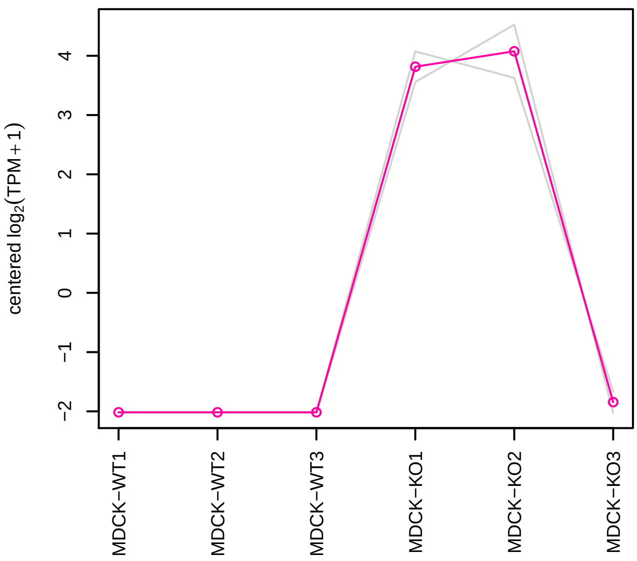

subcluster11, 1transcripts

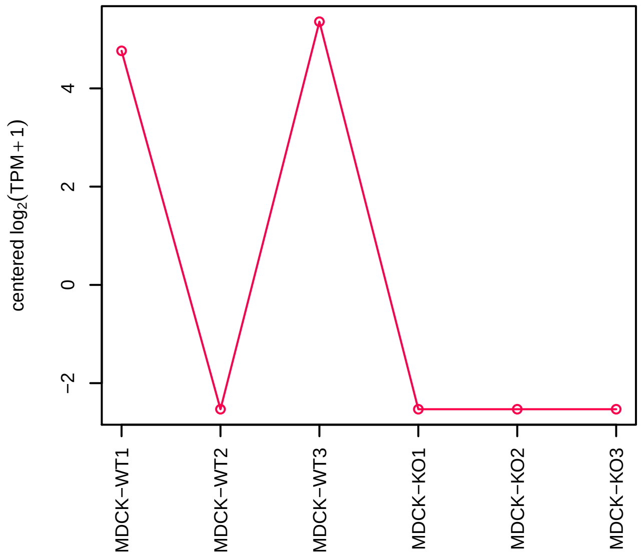

Supplement: S1 File — (ZIP) [file pone.0311069.s002.zip › S2_File/1_DEA/subcluster/union.DE_transcript_subcluster.pdf]
