# Supplementary material for: Tumorigenicity decrease in Bcl-xL deficient MDCK cells ensuring the safety for influenza vaccine production
Source: PLoS One. 2024 Dec 16;19(12):e0311069. doi: 10.1371/journal.pone.0311069 (PMC11649150; doi:10.1371/journal.pone.0311069)
Supplement: S1 File — (ZIP) [file pone.0311069.s002.zip › S2_File/3_KEGG_enrichment/pathwaymap/B_vs_A.DEG_Pathway.html]

DEG KEGG Pathway Statistic


Different Expressed Gene Pathway Annotation of B\_vs\_A

| #Pathway | Genes involved in the pathway |
| Bile secretion | EPHX1;FXYD2;HMGCR;LOC102154822;BAAT;LOC480777;ADCY1\_1;UGT2A3;LDLR;SLC4A5;SLC4A4;SLCO1B3;UGT1A6(down);LOC102154742;SLC4A2;SLC5A1;SLC10A2;RXRA;SLC9A1;SLC9A3;NR0B2;NCEH1;ABCB1;SLC22A7;ABCB4;NR1H4;PRKACA;CYP7A1;ABCG2;LOC119869567;AQP4;AQP1;ATP1B4;ATP1B2;ATP1B3;AQP8;AQP9;ADCY4(up);ABCC4;ABCB11;KCNN2;SLCO1A2;SCT;SLC27A5;ATP1B1;ABCG5;ADCY5;ADCY6;ADCY7;ADCY1;ADCY2;GNAS;ABCG8;SCARB1;ADCY3;ADCY8;ADCY9;SLC2A1;SLC51A;SLC51B;ATP1A4;ATP1A3;SLC22A1;ATP1A1;PRKACB;SCTR;SLC10A1(up);SLC22A8;CFTR;ABCC2;ABCC3 |
| Long-term depression | PPP2R1A;PPP2R1B;GRIA2;GRIA3;HRAS;GRIA1;IGF1R;MAP2K2;MAP2K1;IGF1;ARAF;ITPR3;LOC485435;CRH;GNAI2;GNAI3;RYR1;CACNA1A;NRAS;GUCY1A1;MAPK3;GUCY1A2;PRKG1;RAF1;GNAO1;LYN;BRAF;GRM1;GUCY1B1;NOS1;PLCB2;PLCB3;PLCB4;GRID2;PRKCA;PRKCB;PLA2G4E(up);GNAI1(up);PLCB1(up);PRKCG;PPP1R17;ITPR2;PLA2G4B;PLA2G4A;ITPR1;KRAS;PLA2G4F;PLA2G4D;GNAQ;GNAS;PPP2CA;PRKG2;PPP2CB;GNAZ;GNA12;GNA13;GNA11;MAPK1;CRHR1 |
| Plant-pathogen interaction | SUGT1;CALM2;CALM3;CALM1;CTSF;LOC100687242;GK2;LOC102153034(down);CALML6;CALML5;CALML4;HSP90AB1;MAP2K1;HSP90B1;HSP90AA1;LOC612100;GK |
| Gastric acid secretion | KCNE2;CALML5;KCNQ1;ACTG1;KCNJ16(down);ADCY1\_1;LOC102153034(down);ATP1B2;CAMK2G;KCNJ10;CALM3;MYLK3;SLC4A2;CALML6;GNAI2;GAST;CCKBR;CAMK2A;CALM2;SLC9A1;CALM1;ATP4A;KCNJ1;KCNK2;GNAI3;CAMK2D;SSTR2;HRH2;CAMK2B;ATP1A3;KCNJ2(down);LOC119869567;SLC9A4;PLCB2;PLCB3;PLCB4;KCNJ15;PRKCA;ATP4B;ATP1B4;PRKCB;CHRM3;ATP1B3;PRKCG;ATP1B1;ADCY4(up);PLCB1(up);LOC488190;KCNK10;SLC26A7;ITPR2;ITPR3;ITPR1;MYLK;GNAI1(up);GNAQ;ADCY5;ADCY6;ADCY7;ADCY1;ADCY2;ADCY3;SST;GNAS;ADCY8;ADCY9;EZR;CALML4;ATP1A4;PRKACA;ATP1A1;PRKACB;MYLK2;ACTB;CFTR |
| p53 signaling pathway | SERPINB5;FAS;CCND2;PMAIP1;SESN1;SESN2;SESN3;CDKN1A;ATM;LOC487020;BAX;LOC100683637;CCNG1;CCNG2;ATR;RRM2;BBC3;THBS1;TSC2;SERPINE1;RCHY1;PERP;PPM1D;CDK1;CCNE2;GADD45A;GADD45B;CCNE1;LOC100689004;LOC612475;GADD45G;COP1;RRM2B;CASP9;DDB2;CHEK2;ADGRB1;APAF1;CHEK1;CDKN2B;IGF1;SIAH1;TP73(down);TP53I3;IGFBP3;BCL2L1(down);CYCS;MDM2;SHISA5;MDM4;EI24;CCNB1;TP53;CASP3;CCND1;CCND3;GTSE1;ZMAT3;CASP8;PIDD1;SFN;PTEN;CDK2;CDK4;CDK6;RPRM;GORAB;STEAP3;BID;BCL2 |
| Proteasome | PSMD8;PSMB11;PSMB10;LOC119880346;PSMD4;PSMD7;PSMD6;PSMD1;PSMD3;PSMD2;LOC610188;LOC100687772;LOC100684844;LOC100687152;PSMF1;LOC100687556;PSMA2;PSMA3;PSMA1;PSMA6;LOC100686569;PSMA4;PSMA5;PSMC1;PSMC2;PSMC3;PSMC4;PSMC5;PSMC6;PSMA7;PSMB8(down);IFNG;PSME4;ADRM1;PSME2;PSME3;PSME1;LOC100687259;PSMA8;SEM1;LOC100686432;LOC477325;LOC100687060;PSMD14;LOC100683681;PSMD11;PSMD13;PSMD12;PSMB9;PSMB7;PSMB6;PSMB5;PSMB4;PSMB3;PSMB2;PSMB1 |
| Carbon fixation pathways in prokaryotes | MMUT;MTHFR;IDH2;IDH1;ACO1;ACAT1;ACAT2;PC;ACSS2;FH;ACLY;MCEE;ACO2;ACSS1 |
| AMPK signaling pathway | PCK2;LOC100856339;PRKAG1;PPP2R3C;PRKAG3;PRKAG2;CD36;AKT1;AKT2;AKT3;FBP2;ADIPOQ;EEF2K;PIK3CA;PIK3CB;PIK3CD;MAP3K7;LOC102154144;PPP2R3A;PPP2R3B;RAB11B;ADRA1A;IGF1R;STK11;LIPE;PPP2R2D;CREB5;PPP2R2A;PPP2R2C;SCD5;ACACB(down);HMGCR;LOC608800;CFTR;CAB39L;PPP2CA;SLC2A4;PCK1;CCND1;STRADA;STRADB;PPP2CB;ELAVL1;G6PC1;G6PC3;G6PC2;CRTC2;SCD;PPARGC1A;AKT1S1;PIK3R2;PIK3R1;SIRT1;IGF1;PRKAB2;PRKAB1;LOC119870217;LOC119870216;LOC119870215;GYS1;PPARG;GYS2;HNF4A;INS;CAMKK2;EIF4EBP1;PPP2R5D;PPP2R5E;PPP2R5A;PPP2R5B;PPP2R5C;LOC100683203(up);TSC2;TSC1;RAB8A;ADIPOR2;ADIPOR1;TBC1D1;RPS6KB1;RPS6KB2;FASN;RAB10;RAB14;ACACA;LEPR;MTOR;IRS4;IRS1;IRS2;LEP;SREBF1;MLYCD;RHEB;PPP2R1A;PPP2R1B;CAB39;PRKAA2;PRKAA1;FOXO3;RAB2A;FOXO1;LOC111096471(down);RPTOR;INSR;CCNA2;CCNA1;PFKP;PFKM;PFKL;CPT1A;CPT1C;CPT1B;LOC119874067;CREB3;CREB1;LOC480466;PFKFB1;PFKFB3;PFKFB2;PFKFB4;CREB3L2;CREB3L3;CREB3L1;FBP1(up);CREB3L4;PDPK1;EEF2;PPP2R2B(up) |
| beta-Alanine metabolism | AOC3;ACADS;ALDH3A1;AOC2;ABAT;GADL1;GAD1;CARNS1;ALDH1A3;SMOX;DPYD;HADHA;DPYS;ALDH3B1;ALDH6A1;ALDH9A1;ALDH7A1;ALDH1B1;EHHADH;CNDP1;CNDP2;MLYCD;ACOX3;ACOX1;ALDH2;ECHS1;UPB1(up);LOC483960;HIBCH |
| mTOR signaling pathway | LOC100856339;STK11;IKBKB;AKT1;AKT2;AKT3;DVL2;DVL3;PIK3CA;PIK3CB;PIK3CD;CLIP1;LOC102154144;WNT16;FLCN;IGF1R;DDIT4;SLC38A9;LOC119868328;KRAS;CAB39L;TTI1;GRB10;WDR59;GSK3B;PTEN;GRB2;WNT7A;STRADA;STRADB;WNT7B;ATP6V1D;ATP6V1F;ATP6V1A;SEH1L;HRAS;SLC7A5;ATP6V1H;IRS1;FZD10;RICTOR;NPRL3;NPRL2;TSC2;LOC481939;AKT1S1;PIK3R2;PIK3R1;WNT10A;LOC119870215;IGF1;FZD1;LOC119870217;LOC119870216;SEC13;CHUK;LOC119868276;EIF4E;TELO2;RHOA;DVL1;EIF4B;RPS6KA1;RPS6KA2;DEPTOR;RPS6KA6;TNFRSF1A;SLC3A2;INS;LOC100686736;EIF4EBP1;ATP6V1G2;ATP6V1G1;DEPDC5;LAMTOR4;LAMTOR5;WNT5A;LAMTOR1;LAMTOR2;LAMTOR3;WNT3A;SESN2;RPS6;MIOS;RPS6KB2;RAF1;TSC1;FNIP1;FNIP2;FZD3;SKP2;FZD6;FZD7;FZD8;FZD9;RPS6KB1;TBC1D7;MAPK3;WDR24;MAPK1;WNT6;RNF152;FZD2;ATP6V1G3;LOC102156380;MTOR;PRKAA1;MLST8;LRP6;SOS2;SGK1;LRP5;WNT3;WNT2;WNT1;ATP6V1E1;MAP2K1;ATP6V1E2;WNT4;WNT2B;LOC111090910;EIF4E1B;LOC119870343;RHEB;FZD4;LOC106559872;WNT10B;CAB39;PRKAA2;LOC491854;MAP2K2;INSR;RPTOR;LPIN2;LPIN3;LPIN1;NRAS;PRR5;MAPKAP1;WNT8A;WNT8B;LOC119863906;ATP6V1B1;ATP6V1B2;WNT9B;ULK2;RRAGD;RRAGA;RRAGC;RRAGB;PRKCA;PRKCB;PRKCG;LOC100685582;TNF;PDPK1;EIF4E2;LOC608772;WNT5B;BRAF;WNT9A;ATP6V1C2;ATP6V1C1;WNT11 |
| Circadian rhythm - fly | ARNTL;HLF(up);CLOCK;GSK3B;PER2;CSNK1E |
| Circadian rhythm | FBXW11;CLOCK;PRKAG1;PRKAG3;PRKAG2;PRKAA2;PRKAA1;RORB;NR1D1;RORA(up);ARNTL;SKP1;BHLHE40;BHLHE41;RORC;CRY1;CRY2;CSNK1D;CSNK1E;CUL1;PRKAB2;PRKAB1;CREB1;PER2;RBX1;FBXL3;BTRC;NPAS2;LOC111090648 |
| Circadian entrainment | ADCY1\_1;CACNA1H;CACNA1I;CALM2;CALM3;CALM1;RYR1;RYR3;CACNA1C;GNG10;GNG11;GNG12;CACNA1G;LOC119863873;GNB4(up);GRIN1;CAMK2D;CAMK2G;CAMK2A;CAMK2B;ADCY4(up);PER2;ADCY5;ADCY6;ADCY7;ADCY1;ADCY2;ADCY3;ADCY8;ADCY9;GNG7;GNG4;GNG5;GNG2;GNG3;GNG8;ADCYAP1;RASD1;GRIA2;GRIA3;GRIA1;GRIA4;LOC100855681;GUCY1A1;GUCY1A2;PRKG1;PRKG2;GUCY1B1;LOC119869567;PLCB2;PLCB3;PLCB4;FOS;GNAI1(up);MTNR1B;ITPR3;ITPR1;GNAQ;GNAS;CALML6;CALML5;CALML4;GNG14;LOC609484;RYR2;CACNA1D;LOC102153034(down);ADCY10;GNG13;GNAI2;GNAI3;MTNR1A;MAPK3;MAPK1;GNAO1;LOC485435;GNB5;GNB1;GNB3;GNB2;PLCB1(up);KCNJ3;KCNJ6;KCNJ5;KCNJ9;PRKACA;PRKACB;ADCYAP1R1;NOS1;GRIN2D;PRKCA;PRKCB;PRKCG;CREB1;RPS6KA5(up);GRIN2B;GRIN2C;GRIN2A;GNGT2;GNGT1 |
| Phagosome | SFTPD;TAP1;TAP2;ACTG1;DLA-64;CD36;LOC607467;CTSS;FCGR1A;CANX;MSR1;C1R(up);MRC2;LOC100686787;LOC106559121;ATP6V0D1;FCAR;LOC100856137;RAB5A;RAB5C;RAB5B;DLA-79;HLA-DRB1;LOC119879788;PIK3C3;CTSV;LOC106559212;TUBB4B;TUBB4A;LOC106559111;TLR2;TLR6;COLEC11;ACTB;LOC478702;ATP6V1D;ATP6V1F;ATP6V1A;RILP;ITGA2;ITGA5;CD209;MARCO;TUBA1C;THBS4;PIKFYVE;THBS2;THBS3;THBS1;LOC481939;TFRC;COLEC12;LOC100855928;RAB7A;ITGAM;MRC1;TUBB6;LOC610540;DLA88;TUBB3;TUBB1;DYNC1LI1;DYNC1LI2;TUBB2A;TLR4(down);TUBAL3;LOC610636;OLR1(up);MPO;TUBB;ITGAV;ATP6V0A4;CD14;ATP6V1G3;ATP6V1G2;ATP6V0A2;LOC477570;NCF2;NCF1;TUBA8;SCARB1;NCF4;DYNC1H1;SEC61G;LOC102153980;DYNC2H1;TCIRG1;LOC106559577;C3;ATP6V0B;ATP6V0C;COMP;LOC111097181;ATP6V1H;RAB7B(up);LAMP2;LAMP1;LOC491231;LOC119869025;ITGB2(down);DLA-DOA;DLA-DOB;ATP6V1E1;STX7;ATP6V1E2;STX12;LOC111090910;STX18;LOC119870343;LOC478984;LOC479260;ATP6V1G1;ATP6V0D2(up);LOC102154760;CORO1A;PLA2R1(up);LOC106557476;CLEC7A(down);EEA1;DYNC1I2;LOC608051;ATP6V1B1;ATP6V1B2;ATP6V0A1;ITGB1;NOS1;ITGB3;ITGB5;RAC3;RAC1;LOC119881611;FCGR2B;HGS;LOC102152234;CYBA;LOC119869046;ATP6V0E1;TUBA4A;SEC61B;ATP6V0E2;SEC22B;VAMP3;CALR;DLA-DRA(down);DLA-DMB;DYNC1I1;DLA-DMA;ATP6V1C2;ATP6V1C1;LOC119866377;SEC61A1;SEC61A2;M6PR |
| ErbB signaling pathway | SHC4;PTK2;MYC;CRKL;SRC;CDKN1B;CDKN1A;EGFR;MAP2K1;GAB1;PLCG2;BAD;LOC100856339;MAP2K7;MAPK10;MAP2K4;ABL1;CRK;ABL2;RAF1;EGF;MAP2K2;TGFA;SHC2;HBEGF;PIK3CA;PIK3CB;NRAS;RPS6KB1;ARAF;RPS6KB2;CAMK2D;ELK1;PIK3R2;PIK3R1;NRG1;NRG3;NRG2;NRG4;MAPK1;PAK3(up);ERBB2;ERBB3;CAMK2G;SHC1;ERBB4;SHC3;PRKCA;LOC119870216;LOC119870215;PRKCB;AREG(up);CAMK2A;PRKCG;AKT2;JUN;MTOR;CAMK2B;HRAS;KRAS;BRAF;LOC119870217;MAPK8;SOS2;MAPK3;MAPK9;NCK1;NCK2;AKT1;PIK3CD;PLCG1;CBL;GSK3B;AKT3;STAT5B;STAT5A;GRB2;EIF4EBP1;EREG;BTC;PAK4;PAK5;PAK6;PAK1;PAK2 |
| MAPK signaling pathway - fly | CIC;PPM1B;BMPR1B;TEC;MAPK12;MAP3K7;DUOX1;MAP2K4;EGFR;UBE2D3;TBX2;UBE2D1;MAP2K3;SPRY2;MAP2K1;MAP2K7;MAPK11;MAPK10;PFN3;RREB1;MAP3K5;ROS1;MAPK14;SHC1;YWHAZ;STRN4;MAP3K9;STRN3;RASA1;YWHAQ;JUN;PFN2;MAP3K4;DUSP10;DOK2;MAPK3;GRB2;LOC100855903;YWHAB;CYLD;KSR2;KRAS;MEF2A;ETS1;STRN;XPO1;DUOX2;SOD2;SIAH1;TRAF6;TBL1XR1;FRK;LOC100683481;MAPK8;SRC;LOC106558389;BMP4;NR2E1;IPO7;PROX1;RASA3;LOC479459;MAPK9;TBL1X;SOS2;ETV7;ETV6;PPP2CA;TAB2;PTPN11;PPP2CB;TNIK(up);LOC111094784;MAPK13;PFN1;UBE2D2;LOC609669;PFN4;BRAF;RAC1;MAPK1;RAC3;ATF2 |
| MAPK signaling pathway | HSPA2;PPP3R2;CRKL;MKNK1;HSPA8;CASP3;IKBKB;AKT1;AKT2;AKT3;ARAF;RAPGEF2;LOC607182;BDNF;NR4A1(up);EGF;MAP4K3;CACNA1H;CACNA1I;MAP3K8;PPP3R1;GNG14;CACNA1A;CACNA1B;CACNA1C;CACNA1D;NTRK1;NTRK2;CACNA1G;LOC119866928;RELB;RELA;FGFR1;MAP3K20;IRAK4;ERBB2;ERBB3;IGF1R;DDIT3;LOC119873859;ERBB4;FGF18;PLA2G4E(up);EPHA2;STK3;FASLG;CACNA2D2;CACNA2D3;FGFR4;CACNA2D1;FGFR2;FGFR3;CACNA2D4;KRAS;MYD88;MAX;CSF1;KIT;GRB2;ANGPT4;ELK4;ARRB2;RAC2(up);PAK1;PAK2;MAPK11;RASGRP2;RASGRP3;NGF;RASGRP1;STMN1;RASGRP4;HRAS;RRAS2;PDGFB;RRAS;FGF17;PPP3CA;CACNB2(up);MAPKAPK5;DUSP2(up);MAPKAPK3;MAPKAPK2;IL1R1;TGFA;DAXX;TGFB2;PPM1B;PPM1A;FGF10;RAC1;JUN;MAP3K7;MECOM;ELK1;NF1;LOC119870216;ANGPT2;PLA2G4F;ANGPT1;VEGFB;EREG;JUND;IGF1;FOS;LOC119870217;CDC25B;LOC119870215;MAP3K4;FGF7;CHUK;MRAS;EFNA4;VEGFD;VEGFA;ECSIT;FGF9;FGF8;CRK;FGF6;FGF5;FGF4;PTPN7;FGF2;PTPN5;ARRB1;RPS6KA1;RPS6KA2;RPS6KA4;MAPK8IP1;TNFRSF1A;PPP5C;FLNC(up);INS;MEF2C;MAP3K2;CD14;GADD45A;FLNA;FLNB;LOC474850;FGF22;GADD45B;FGF20;LAMTOR3;PDGFRB;PDGFRA;MAP3K1;MAPK8IP2;MYC;NFATC1;MKNK2;MAP3K6;IL1RAP;RAP1B;RAP1A;CACNA1E;MAPK14;AREG(up);TGFB3;MAPK10;GNG12;MAPK12;RASGRF1;RPS6KA6;FLT4;CACNB4;RAF1;FLT1;STK4;MAPK8IP3;TEK;ATF2;CACNB3;FGF3;CACNB1;CSF1R;TRADD;DUSP10;MAPK3;MAPK1;DUSP9;DUSP8;DUSP5;NFKB1;NFKB2;MAPK9;FGF1;FGF16;DUSP3;MAPK7;TGFBR2;TGFBR1;NTF3;SRF;TAOK1;MAP3K5;EFNA5;MAPK8;EFNA3;EFNA2;EFNA1;MAP3K3;FGF11;FLT3LG;TP53;DUSP7;TAOK2;RPS6KA5(up);CACNA1S;SOS2;RASGRF2;VEGFC;GADD45G;INSR;MET;PRKACA;NGFR(up);PRKACB;IL1B;CDC42;MAP4K1;TGFB1;TAOK3;MAP4K2;MAP4K4;CACNA1F;EGFR;FLT3(up);MAP2K3;MAP2K2;MAP2K1;MAP2K7;MAP2K6;MAP2K5;IL1A(up);HSP70;PGF;PPP3CB;MAP2K4;FAS;NRAS;IRAK1;MAP3K13;NFATC3;MAP3K14;PDGFA;TRAF2;PDGFC;PDGFD;RAC3;TRAF6;HSPB1;PRKCA;PRKCB;PRKCG;TNF;ATF4;HGF;PLA2G4B;PLA2G4A;DUSP16;RASA1;RASA2;PLA2G4D;PPP3CC;KITLG;PTPRR;NLK;TAB2;TAB1;MAPK13;MAP3K11;MAP3K12;KDR;GNA12;BRAF;MAPT;CACNG1;CACNG2;CACNG3;CACNG4;CACNG5;CACNG6;CACNG7 |
| Methane metabolism | AGXT;LOC482320;PGAM2;FBP2;PGAM1;PFKP;PFKM;PFKL;ACSS2;ACSS1;ENO1;ENO2;ESD;ENO4;PHGDH;ALDOA;LOC608800;ALDOC;ALDOB;SHMT2;SHMT1;ADH5;PSPH;GLYCTK;TKFC;PSAT1;ENO3;FBP1(up) |
| Homologous recombination | ATM;MUS81;BRIP1;XRCC3;XRCC2;RAD54B;RAD54L;SYCP3;POLD1;POLD2;POLD3;NBN;BRCA1;BRCA2;MRE11;BLM;SSBP1;RAD51D;RAD51C;RAD51B;SEM1;LOC100683112;LOC611847;TOP3A;PALB2;RPA1;RPA3;RPA2;RAD52;RAD51;RAD50;EME1;TOP3B |
| Osteoclast differentiation | LOC100856339;PPP3R2;PPP3R1;IKBKB;AKT1;AKT2;IFNAR1;FCGR1A;TYK2;TYROBP;PIK3CA;PIK3CB;PIK3CD;CAMK4;LOC484309;RELB;RELA;LOC484306;BLNK;SIRPA;IFNG;LOC485809;CREB1;IFNAR2;SOCS1;SOCS3;SYK;CSF1;AKT3;CYLD;NFATC1;TNFSF11;NFKBIA;GAB2;ITGB3;IFNGR1;IFNGR2;IL1A(up);IL1R1;OSCAR;JUN;TAB1;GRB2;PIK3R2;PIK3R1;JAK1;FOS;LOC119870217;LOC119870216;LOC119870215;CHUK;PPARG;LOC609523;TNFRSF1A;IRF9;CYBA;LOC611446;LCK;NCF2;NCF1;NCF4;NFATC2;MAP3K7;MAPK14;MAPK10;MAPK11;MAPK12;MAPK13;IL1B;SPI1;STAT2;STAT1;TEC;CSF1R;IFNB1;FHL2;MAPK3;MAPK1;FOSL1;ACP5(down);FOSL2;NFKB1;MAPK8;MAPK9;TGFBR2;TGFBR1;CTSK(up);FOSB(up);NFKB2;LOC478984;TGFB1;TGFB2;JUNB;JUND;PLCG2;MAP2K1;MAP2K7;MAP2K6;MITF;FYN;TRAF2;RAC3;TRAF6;RAC1;FCGR2B;TNF;TNFRSF11A;TNFRSF11B;LCP2;SQSTM1;TAB2;CALCR;MAP3K14;PPP3CA;PPP3CB;PPP3CC |
| Sulfur metabolism | PAPSS1;PAPSS2;BPNT1;BPNT2;MPST;TST;SUOX;ETHE1 |
| Rap1 signaling pathway | LOC100856339;ACTG1;CRKL;TLN2;ADCY1\_1;RAPGEF4;RAPGEF5;RAPGEF6;AKT3;RAPGEF1;RAPGEF2;RAPGEF3;THBS1;EGF;LOC111090226;CALM2;CALM3;CALM1;PIK3CA;PIK3CB;PIK3CD;LOC102156776;PARD6A;PRKD2;MAGI3;MAGI2;MAGI1;CDH1;GRIN1;KRAS;LOC106559087;LOC102156003;IGF1R;ADCY6;EPHA2;ADCY4(up);FARP2;KITLG;FGFR2;FGFR3;FGFR1;ADCY5;LOC487387;ADCY7;ADCY1;ADCY2;ADCY3;ADCY8;CSF1;PLCG1;KIT;F2R;LAT;ACTB;RASGRP2;RASGRP3;NGF;APBB1IP;CTNND1;GNAO1;ENAH;SKAP1;RASSF5;ITGB3;DOCK4;ADCY9;RRAS;PLCB3;CRK;ADORA2B;CDC42;ITGAL;GNAI2;LOC102156901;GNAI3;TIAM1;AKT1;PIK3R2;PIK3R1;ANGPT2;ANGPT1;ANGPT4;LOC119869567;PLCB2;IGF1;PLCB4;LOC119870217;LOC119870216;LOC119870215;MRAS;GNAI1(up);VEGFD;AKT2;VEGFA;LOC489647;VEGFC;VEGFB;FGF7;FGF6;FGF5;FGF4;FGF3;FGF2;FGF1;LPAR2;RAC2(up);GNAQ;GNAS;VAV1;VAV3;VAV2;CALML6;CALML5;INS;PFN1;PFN2;PFN3;PFN4;ITGAM;PRKD3;FGF22;RALB;FGF20;PDGFRB;PDGFRA;FGF9;FGF8;LOC102153034(down);RAP1B;RAP1A;PARD6B;MAPK14;KRIT1;BCAR1;PLCE1;MAPK11;MAPK12;MAPK13;FLT4;LOC119872979;RAF1;RHOA;FLT1;CNR1;TEK;ITGA2B;CSF1R;PRKCB;MAPK3;MAPK1;SIPA1;FGF11;FGF10;FGF17;FGF16;KDR;SRC;RALGDS;EFNA5;EFNA4;EFNA3;EFNA2;EFNA1;LOC106558389;PARD6G(up);LOC119866928;PRKCI;CTNNB1;RGS14;ITGB2(down);AFDN;INSR;ID1;MET;NGFR(up);F2RL3;LPAR3;PRKD1;LPAR4;LPAR5;LOC119869603;EGFR;LOC119869607;MAP2K3;MAP2K2;MAP2K1;MAP2K6;CALML4;FYB1;PGF;ADORA2A(up);ARAP3;NRAS;TLN1;LOC609053;SIPA1L1;FGF18;SIPA1L3;SIPA1L2;BRAF;ITGB1;PDGFA;PDGFB;PDGFC;PDGFD;RAC3;RAC1;PRKCA;LPAR1(up);PRKCG;PRKCZ;HGF;LCP2;VASP;P2RY1;PARD3;HRAS;PLCB1(up);LOC480788;DRD2;GRIN2B;GRIN2A;FGFR4;RALA |
| Glycosphingolipid biosynthesis - globo and isoglobo series | ST3GAL2;ST3GAL1;FUT1;B3GALNT1;B3GALT5;HEXA;A4GALT(up);HEXB;GBGT1;ST8SIA1;FUT2;LOC102151792;NAGA;FUT9 |
| Quorum sensing | SRP54;OXA1L;ACSL3;ACSL1;ACSL6;ACSL4;LOC483960;GAD1;ACSL5(up) |
| Glycosphingolipid biosynthesis - lacto and neolacto series | GGTA1;B3GALT5;LOC111095419;LOC119881513;LOC119880300;B3GALT1;ABO(down);B3GNT5;B3GNT3(down);B3GNT2;GCNT2;B4GALT1;FUT1;B4GALT2;B4GALT4;FUT2;ST3GAL3;B3GNT4;ST3GAL6;B3GALT2;ST3GAL4;B4GALT3;B3GALNT1;LOC476397;ST8SIA1;FUT7;FUT4;FUT9;LOC102151792;A4GALT(up);LOC100685413 |
| Sphingolipid metabolism | SPHK2;UGCG;LOC100855615;GBA;UGT8;SPHK1;SMPD4;SMPD1;SGMS2;SGMS1;SMPD2;GAL3ST1;SGPP2(down);CERS6;ACER3;CERS4;CERS5;CERS2;ASAH1;ASAH2;CERS1;SPTLC2;SPTLC3;SPTLC1;GBA2;SGPP1;B4GALT6;PLPP1;ACER2;PLPP3;PLPP2;CERS3(up);SMPD3(up);ENPP7;KDSR;ACER1;GALC;DEGS1;DEGS2;ARSA;SGPL1;PSAP;CERK;GLB1;LOC119864605;NEU4;NEU1;NEU2;NEU3 |
| Two-component system | UQCRFS1;CYTB;CYC1;LOC612295;CYCS;ACAT1;ACAT2;LOC100689004;GLUL;ME2;ALPI;GLS;COX15;GLS2;ALPL |
| Glycosphingolipid biosynthesis - ganglio series | B3GALT4;ST3GAL2;ST3GAL1;B4GALNT1;ST3GAL5;HEXA;ST8SIA5(up);HEXB;ST8SIA1;SLC33A1;GLB1;ST6GALNAC6;ST6GALNAC5;ST6GALNAC4;ST6GALNAC3 |
| Cell cycle | BUB1B;PLK1;PKMYT1;CDKN2B\_1(down);GADD45A;GADD45B;ANAPC11;ANAPC10;ANAPC13;GADD45G;ESPL1;LOC100855903;WEE2;WEE1;CUL1;CDKN2B;CDKN2C;SMC1B(up);CDKN2D;MDM2;SMAD3;LOC479459;CCND1;CCND3;FZR1;GSK3B;SFN;LOC609669;DBF4;RBX1;CCNH;CCND2;CDKN1C;CDKN1B;CDKN1A;CDC16;CCNE2;CCNE1;TTK;STAG2;CHEK2;CHEK1;SMC1A;BUB1;BUB3;CDC25C;CDC25B;CDC25A;STAG1;LOC100856295;LOC100685549;ANAPC1;ZBTB17;ANAPC2;ANAPC5;ANAPC4;ANAPC7;TP53;CDC27;CDC26;CDC23;CDC14B;CDC14A;CDC20;LOC476070;TFDP2;CREBBP;HDAC1;HDAC2;SMAD4;ATM;LOC487020;SMAD2;PRKDC;ATR;ABL1;CDC6;CDC7;SKP2;SKP1;SMC3;PTTG1;RBL1;RBL2;E2F5;LOC612475;EP300;MYC;CDK1;CDK2;CDK4;CDK6;CDK7;CDC45;MAD2L2;TGFB1;TGFB2;TGFB3;LOC102156563;YWHAZ;CCNA2;CCNA1;MAD1L1;YWHAQ;RB1;YWHAH;YWHAB;YWHAG;YWHAE;PCNA;RAD21;ORC6;ORC4;ORC5;ORC2;ORC3;ORC1;MCM6;MCM5;MCM4;MCM3;MCM2;LOC111090648;CCNB1;E2F4;E2F3;E2F2;E2F1;TFDP1;LOC608573 |
| Penicillin and cephalosporin biosynthesis | DAO(down) |
| Lysine degradation | COLGALT2;AASS(down);BBOX1;KMT5B;KMT5C;KMT5A;ASH1L;SETD1A;SETD1B;GCDH;CAMKMT;EHMT1;EHMT2;SETMAR;MECOM;PLOD3;DLD;ACAT1;ACAT2;DOT1L;SETDB1;SETDB2;KMT2C;KMT2B;KMT2A;AADAT;KMT2E;KMT2D;SUV39H1;SUV39H2;COLGALT1;HADHA;HYKK;ALDH9A1;ALDH7A1;ALDH1B1;SETD2;EHHADH;SETD7;PHYKPL;HADH;PIPOX;DLST;PLOD1;ALDH2;ECHS1;NSD1;NSD3;NSD2;TMLHE |
| Fluid shear stress and atherosclerosis | LOC100856339;LOC479911;ACTG1;LOC479912;GSTA4;SUMO3;SUMO2;IL1R2(up);NQO1;IKBKB;AKT1;AKT2;AKT3;LOC611366;ASS1;CAV3;LOC119867218;CALM2;TP53;CALM1;PIK3CA;PIK3CB;PIK3CD;LOC486404;MAP3K5;HSP90AB1;SUMO1;TRPV4;RELA;CDH5;IFNG;MGST1(down);LOC490297;CTSV;MMP2(down);BMP4;BMPR2;LOC481841;ACTB;TNF;CCL2;ITGB3;HSP90B1;MGST2;MGST3;IL1A(up);IL1R1;PECAM1(up);CAV2;JUN;CALML5;PIK3R2;PIK3R1;CAV1;NPPC;FOS;LOC119870217;LOC119870216;LOC119870215;CHUK;VEGFA;RHOA;LOC612100;CALM3;GSTO2;GSTO1;GSTT2B;TNFRSF1A;SDC1;HMOX1;CALML6;ITGAV;CALML4;MEF2C;MEF2A;PRKCZ;LOC100856518;NFE2L2;THBD(up);NCF2;NCF1;LOC102153034(down);MAP3K7;MAPK14;MAPK10;MAPK11;MAPK12;MAPK13;IL1B;LOC100687242;ITGA2B;MAPK7;ICAM1;NFKB1;MAPK8;MAPK9;LOC119872870;KDR;ACVR2A;ACVR2B;VCAM1;RAC2(up);KEAP1;LOC477556;PIAS4;LOC610304;HSP90AA1;LOC477558;PTK2;ACVR1;SRC;SDC4;LOC100685896;PRKAA2;PRKAA1;CTNNB1;BMPR1A;BMPR1B;MAP2K7;MAP2K6;MAP2K5;MAP2K4;LOC476006;EDN1(down);KLF2;PDGFA;PDGFB;NOS3;RAC3;RAC1;LOC119871262;PLAT;CYBA;SQSTM1;LOC474938;SELE;GSTP1;MMP9;BCL2 |
| Neuroactive ligand-receptor interaction | AVPR1B;CHRNB4(down);AVPR1A;CHRNA2;GHR;PRLH;LTB4R2;CHRNA6;POMC;MC2R;PTH;P2RY13;C5AR1;CCK;HCRT;UTS2R;P2RY6;GHSR;LOC100686142;DRD4;HTR1E;CHRNB1;RXFP1;PRL;RXFP3;RXFP2;PTGER1;PTGER2;THRA;AGT;EDN1(down);GLP1R;HTR2A;GRIN1;EDN2;TRPV1;LOC475521;GNRH1;HTR1D(down);ADRA2C;MC1R;GIP;FPR2;FSHR;MCHR2;MCHR1;CTSG;HTR6;HTR7;HTR4;BRS3;SCT;GABRG2;CALCB;ADRA1D;ADRA1A;PYY;ADRA1B;GH1;CRSP-2;AVPR2;NPY1R;CHRNA10;NPB;GALR3;F2R;GALR1;NMBR;OPRK1;HCRTR1;PTAFR;HCRTR2;NTS;HTR5A;GHRHR;KNG1;SSTR1;GRIA2;TSHB;GABRG1;GRIA1;NTSR2;GRIA4;NTSR1;TRHR;ADM;CALCA;PTGDR;GABBR1;EDNRB;CYSLTR2;MC4R;CYSLTR1;CCKBR;PLG;ADORA2B;C3AR1;GCG;GALR2;PRLR;GPR35;ADRB3;VIP;OXT;HRH4;SSTR2;GRM8;F2(down);HTR2C;HTR2B;GRM4;GRM5;GRM6;GRM7;GRM1;GRM2;GRM3;TRH;CHRM4;GRP;CCKAR;GAL;PTGER3;MC3R;NPFFR2;NPFFR1;F2RL3;HRH1;APLN;F2RL2;MTNR1B;MTNR1A;TACR3;TACR2;TACR1;APLNR;GZMA;CHRNA9;LPAR6;GABRQ;GABRP;EDN3;S1PR3;AGTR2;LPAR4;NPBWR1;GABRE;GABRD;GABBR2(up);NPY;LOC102151131;PTGFR;GHRH;GHRL;GABRR1;GABRR2;GABRR3;ADRA2A;HRH2;LTB4R;GNRHR;ADRA2B;ADCYAP1;GRPR;GABRA5;GABRA4;GABRA6;GABRA1;GABRA3;GABRA2;CNR1;TAAR5;CNR2;CGA;S1PR2;CHRNB3;CHRNB2;P2RY10;P2RY11;S1PR5;S1PR4;GABRG3;GRIA3;C3;CHRNA7(down);GPR83;ADORA3;LHCGR;P2RY14;TBXA2R;GRID1;GRID2;MLN;LOC116183080;LEPR;CHRNA4;CHRNA5;OPRM1;CHRNG;CHRNA1;MAS1;CHRNA3;GABRB1;GABRB2;GABRB3;TSPO;MC5R;GIPR;BDKRB1;TAAR4;BDKRB2;GPR156;NPY5R;GRIK1;GRIK2;GRIK3;GRIK4;GRIK5;LOC106560021;LEP;ADRB1;SSTR3;GPR50;SSTR5;C5;OXTR;LPAR2;LPAR3;F2RL1;MLNR;CRHR2;CALCR;GLP2R;CRHR1;PTH1R;ADRB2(down);AVP;DRD3;UCN2;PRSS2;CRH;TSHR;P2RX1;UCN3;P2RX3;P2RX2;P2RX5;P2RX4;P2RX7;P2RX6;ADORA2A(up);ADCYAP1R1;HRH3;FSHB;TAC1;CHRM2;TAC4;OPRL1;GRIN3A;SST;LOC489082;GRIN3B;VIPR2;VIPR1;LOC100686959;PPY;OPRD1;UTS2;KISS1R;NR3C1;CHRND;PTGIR;CALCRL;NMB;CHRM5;LPAR1(up);CHRM3;GLRB;CHRM1;PTH2R;SCTR;P2RY8;EDNRA(up);P2RY2;GCGR;P2RY1;THRB(up);AGTR1;P2RY4;NMUR2;NMUR1;PARD3;GLRA3;GLRA2;GLRA1;DRD5;DRD2;NPY2R;DRD1;GRIN2B;GRIN2C;GRIN2A;PRLHR;GRIN2D;PTGER4;CRSP-3;ADORA1;HTR1F;HTR1A;HTR1B;CRSP-4 |
| Human immunodeficiency virus 1 infection | TAP1;LOC100856339;PPP3R2;DLA-64;AP1M2;AP1M1;IKBKB;AKT1;AKT2;AKT3;CGAS;WDFY1;B2M;CALM2;PPP3R1;CALM1;GNG14;PIK3CA;PIK3CB;GNG10;GNG11;GNG12;GNG13;LOC119863873;FADD;WEE2;RELA;IRAK1;CUL1;IRAK4;DLA-79;LOC487020;FASLG;KRAS;MAPK14;AP1G2;PTK2B;PLCG1;BAK1;TLR2;GNG7;GNG4;GNG5;GNG2;GNG3;PAK4;ATR;PAK6;PAK3(up);CD3D;PAK1;GNG8;MAPK11;NFATC1;NFATC2;NFATC3;NFATC4;GNAO1;HRAS;NFKBIA;LOC119866339;CRK;LOC100687971;RAC3;TAP2;RBX1;BCL2L1(down);RNF7;GNAI2;LOC100855681;LOC119870849;JUN;LOC102156901;CRKL;CDC25C;ELOC;PIK3R2;PIK3R1;LOC119876828;MYD88;LOC102154266;CHEK1;LOC119872979;FOS;LOC119870217;LOC119870216;LOC119870215;DLA88;CHUK;GNAI1(up);LOC111090226;PRKCG;PAK5;ITPR2;ITPR3;ITPR1;CALM3;IRF3;TLR4(down);LOC100686162;RAC2(up);GNAQ;TNFRSF1A;LOC100683567;AP1G1;LOC102156776;CALML6;CALML5;CALML4;PAK2;STING1;CD3G;LOC611406;GNB4(up);ATM;LOC102153034(down);MAP3K7;CD4;FBXW11;MAPK10;CCR5;MAPK12;MAPK13;IFNB1;RAF1;GNAI3;LOC106559087;TBK1;SKP1;RPS6KB1;TRADD;RPS6KB2;MAPK3;MAPK1;NFKB1;MAPK8;MAPK9;GNB5;GNB1;GNB3;GNB2;MTOR;CYCS;CUL5;TNFRSF1B;WEE1;MAP2K3;DDB1;PLCG2;IFNA7;BAD;IFNA5;TAPBP;CDK1;LOC102156003;TAB1;AP1S1;PTK2;LIMK2;LOC119869603;LIMK1;LOC100685367;LOC119869607;BAX;RIPK1;MAP2K2;MAP2K1;CD247;MAP2K7;MAP2K6;AP1S2;ELOB;LOC102152609;FAS;BID;NRAS;PXN;LOC609053;AP1B1;TRAF2;TRAF5;TRAF6;RAC1;PRKCA;PRKCB;PDIA3;LOC100689004;CD3E;TNF;PIK3CD;CFL2;CFL1;LOC111090648;CCNB1;CASP3;LOC480788;TAB2;CALR;BTRC;LOC119863891(up);CASP8;CASP9;CUL4A;CUL4B;GNGT2;GNGT1;GNA11;PPP3CA;PPP3CB;PPP3CC;BCL2 |
| Coronavirus disease - COVID-19 | LOC119870304;LOC608876;LOC489638;IL6ST;IFNAR2;IFNAR1;LOC111096925;TYK2;LOC481722;LOC607189;LOC106558130;PIK3CA;PIK3CB;PIK3CD;LOC106558139;LOC119863879;IRAK1;LOC119872934;IRAK4;F2(down);LOC102154836;LOC119876828;LOC106559212;LOC102152540;LOC106559359;LOC106558632;ACE2;LOC106559375;LOC100856351;RPS27L;LOC100683928;RPS27A;LOC119868360;RPL7A;LOC106559452;LOC475097;ISG15;JAK1;LOC106559922;LOC100856037;LOC106559926;LOC106559924;LOC119871462;LOC480235;CHUK;TLR4(down);VWF;LOC119867317;LOC102153980;LOC100687749;LOC111095093;LOC100687065;C8B;LOC478576;LOC100683963;IFIH1;LOC100687833;EGFR;PLCG2;PLCG1;LOC486061;LOC486183;LOC119864693;LOC119869838;LOC106558701;LOC102155971;LOC106558923;NRP1;LOC119864697;C3AR1;SELP(up);LOC100687566;LOC100688232;IKBKB;C5AR1;IKBKE;FAU;LOC611750;RELA;FGG;FGA;FGB;LOC119872856;LOC111094581;LOC607191;LOC100683749;LOC106560108;LOC100686416;LOC119870571;C1QC;C1QB;CSF2;CSF3;UBA52;LOC607844;MAVS;CCL2;RPL37A;LOC100856286;RPL27A;TAB2;LOC106559265;DDX58;LOC100687735;ADAR;LOC106558744;LOC119876411;IRF3;LOC100686162;LOC100687051;LOC106559461;LOC100683567;LOC106559504;IRF9;LOC100683563;LOC100686736;RSL24D1;LOC119864483;LOC100855547;LOC100682542;RPLP2;RPLP1;RPLP0;LOC481399;LOC492065;LOC611406;LOC119871822;NLRP3;MAPK3;LOC100855538;LOC608684;CFB(down);F13B;IL6R;MAS1;RPS4X;LOC100686899;LOC119872019;LOC119869025;LOC100686354;LOC119870530;RPS3A;RPL10L;LOC102154760;F13A1;LOC102153530;PRKCA;PRKCB;PRKCG;RPL32\_1;LOC608772;MMP3;LOC106557682;MMP1;LOC100856339;LOC102151361;LOC100687285;LOC102153812;LOC119871977;MAP3K7;LOC106559121;LOC106559944;LOC100682801;LOC119871403;LOC100682691;RPL14;RPL15;RPL17;RPL11;RPL12;RPL13;LOC102155634;RPL18;RPL19;SYK;MASP1;MASP2;LOC106559752;LOC477105;LOC106559055;LOC119869344;LOC479033;LOC100683287;JUN;LOC480918;LOC119877709;LOC607833;RPL10A;LOC100856299;LOC119870219;FOS;LOC106557880;LOC106559371;LOC100682679;LOC100687042;TNFRSF1A;LOC111096979;LOC100856611;STING1;LOC479087;AGTR1;LOC100856758;LOC100856208;LOC106558887;RPL18A;RPS15A;RPS7;RPS6;RPS5;RPS3;RPS2;LOC106559080;RPL36AL;IL1B;RPS9;RPS8;STAT3;STAT2;STAT1;IFNB1;RPL13A;C9;C3;C2;C7;C6;C5;LOC100686620;RPS26;RPS27;RPS24;RPS25;RPS23;RPS20;RPS21;RPS28;RPS29;LOC100685924;LOC100684485;RPSA;EIF2AK2;IL6;IL2;LOC106559872;LOC106557704;C8A;LOC100685611;C8G;LOC106557771;RPL29;RPL28;LOC100686364;RPL24;RPL27;RPL26;RPL21;RPL23;RPL22;LOC119870132;LOC100685685;LOC106559787;LOC490332;LOC119873934;LOC100687764;LOC106557609;LOC106558083;LOC106560120;LOC119864957;C1S;LOC607467;CGAS;RPL22L1;C1R(up);LOC106558335;LOC111090701;LOC119879788;LOC102154256;LOC100686103;LOC608162;LOC106559111;TLR2;TLR3;TLR7;LOC111098653;TLR8;LOC100686712;LOC483242;RPL9;NFKBIB;CXCL8(up);RPL4;NFKBIA;LOC100685244;RPL3;IL12A(up);C1QA;LOC488356;PIK3R2;PIK3R1;MYD88;LOC119864713;LOC119873878;LOC119866623;LOC106557643;LOC612456;LOC106558045;LOC100683111;LOC100682831;LOC100684245;ACE;RPL35A;MAPK14;MAPK10;MAPK11;MAPK12;MAPK13;LOC106558762;RPL8;TBK1;HBEGF;LOC106560013;RPL6;MAPK1;RPL7;NFKB1;MAPK8;MAPK9;LOC106560154;RPL5;LOC486972;LOC111097181;LOC111096443;LOC102156381;LOC102154292;LOC106559099;LOC119866847;CXCL10(down);LOC608750;LOC119865194;IFNA7;IFNA5;LOC100687236;CFD(down);LOC479260;RPS13;RPS12;RPS11;RPS10;RPS17;RPS16;RPS15;RPS14;RPS19;RPS18;IL12B;LOC491854;RPL3L;LOC106559895;ADAM17;LOC111096198;LOC488981;LOC608703;LOC106558419;TRAF3;TRAF6;LOC106559613;TNF;LOC119869046;LOC111090092;LOC119872639;RPL38;RPL36;RPL37;RPL34;RPL35;RPL30;RPL31;LOC119871302 |
| Axon guidance | LOC100856339;PPP3R2;PPP3R1;NRAS;LOC119871983;MYL5;L1CAM;SEMA4A;SEMA4B;SEMA4C;SEMA4D;SEMA4F;SEMA4G;CXCL12;NTNG1;PIK3CA;PIK3CB;PIK3CD;PARD6B;PARD6A;LOC607207;ARHGEF12;LRRC4;SRGAP1;CAMK2G;CAMK2A;CAMK2B;ILK;SSH2;SRGAP3;NTNG2(up);SSH1;SEMA3E;SRGAP2;SEMA7A;KRAS;BMP7;LRRC4C;ROBO1;ABLIM2;PTPN11;ROBO2;GSK3B;BMPR2;PLXNA2;PAK4;PAK5;PAK6;PAK1;PAK2;NFATC2;NFATC3;NFATC4;HRAS;RRAS;DPYSL5;SEMA3D(up);GDF7;LOC100687971;PLXNB2;PLXNB3;PLXNB1;ABLIM1;RAF1;PIK3R2;PIK3R1;LOC100683481;MYL9;LOC102154266;PAK3(up);EPHB2;EPHB3;EPHB1;DPYSL2;EPHB4;UNC5B;UNC5C;UNC5A;UNC5D;GNAI1(up);PARD6G(up);SHH;RHOA;ABLIM3;RHOD;PLXNC1;RAC2(up);SEMA5B;SEMA5A;WNT5B;WNT5A;EFNB2;EFNB3;EFNB1;EPHA8;EPHA7;EPHA6;EPHA5;EPHA4;EPHA3;EPHA2;EPHA1;DCC;TRPC3;TRPC1;TRPC6;TRPC4;TRPC5;ABL1;GNAI2;GNAI3;FZD3;NRP1;MAPK3;MAPK1;ENAH;SRC;EFNA5;EFNA4;EFNA3;EFNA2;EFNA1;FES;LOC119866928;SEMA3A;SEMA3C;SEMA3B;NGEF(up);MYL12B;SEMA3G;SEMA3F;NTN1;PLXNA1;NTN3;NTN4;PLCG1;PLXNA4;MET;WNT4;CDK5;CDC42;PTK2;LIMK2;LIMK1;LOC102152609;PTCH1;PLCG2;BMPR1B;SEMA6D;SEMA6B;SEMA6C;SEMA6A;BOC;FYN;SMO(up);NEO1;SLIT3;SLIT2;SLIT1;RGS3;CAMK2D;ITGB1;RAC3;RAC1;PRKCA;PRKCZ;CFL2;CFL1;ROBO3(up);RYK;RASA1;PARD3;NCK1;NCK2;LOC119863891(up);ROCK1;ROCK2;PDK1;RND1;PPP3CA;PPP3CB;PPP3CC;SSH3 |
| Axon regeneration | DUSP8;RPS6KB1;MAP2K4;PRKAA2;PRKAA1;NOTCH1;MAPK14;LOC475367;INSR;PLCB3;MAP2K7;MAPK11;MAPK12;MAPK13;MAPKAPK2;DGKZ;MAP3K9;PTEN;PIK3CA;PSEN1;DGKQ;CACNA1D;RPS6KB2;PSD3;DGKK;DGKH;DGKI;SLIT2;GNAO1;DGKB;PRKACA;MAPK8;MAPK9;DGKG;DGKD;DGKE;ARHGEF12;EPHB1;PLCB4;RAC3;DUSP5;PSD4;PRKCD;HTR7;TPH1;MTOR;HIF1A;DDC;RHOA;DUSP16;DUSP7;PSD2;FOXO3;DGKA;FAAH;GNAQ;NCK2;GNAS;PLCB2;NTN1;ROBO2;PIK3CD;ADCY9;PSD;DUSP2(up);TPH2;MET;MAP3K13;GNA12;RAC1;PRKACB;MAPK10;PIK3CB;PLCB1(up) |
| Glycolysis / Gluconeogenesis | PCK2;LOC479379;LOC100856533;ADPGK;BPGM;NUDT11;GAPDHS;ALDH3A1;LDHAL6B;LOC119867230;G6PC3;G6PC2;LOC482320;ALDOA;TPI1;AKR1A1;PGAM2;FBP2;PGAM1;PGK1;LOC100686034;LOC111096459;GALM;ALDH1A3;PKLR;G6PC1;FBP1(up);HKDC1;PGM1;LOC100684709;PFKP;GAPDH;PFKM;PFKL;LOC111089999;LOC610338;LOC119876526;PDHB;LOC477441;LDHA;LDHB;LDHC;PDHA2;ALDH3B1;LOC100683724;DLD;HK2;HK3;GCK;HK1;LOC609911;PGM2;LOC102153001;ENO1;ENO2;DLAT;ENO4;ALDH9A1;ALDH7A1;ALDH1B1;LOC608800;ALDOC;ALDOB;LOC102151775;ADH5;ADH4;LOC102154094;PGK2;LOC490690;ACSS1;ALDH2;LOC119870407;PCK1;ENO3;LOC111094513;LOC102152592;MINPP1;LOC106558345;ACSS2;PKM;LOC481849;GPI |
| Aflatoxin biosynthesis | ACACB(down);ACACA |
| Meiosis - yeast | PPP2R1A;PPP2R1B;RAD17;RAD1;GNAI2;GNAI3;CDC6;CDC7;ANAPC2;CDC16;LOC476070;MAD1L1;SMC3;ANAPC11;ANAPC10;STAG2;ESPL1;DMC1;PPP2R5D;CHEK1;SMC1A;SMC1B(up);BUB1;STAG1;ORC6;ORC4;ORC5;ORC2;ORC3;ORC1;MCM6;MCM5;MCM4;MCM3;MCM2;PPP2R5E;ANAPC1;GNAI1(up);ANAPC5;ANAPC4;ANAPC7;KRAS;PPP1CB;PPP1CC;PPP1CA;CDC27;CDC26;PPP2CB;CDC23;CDC14B;CDC14A;CDC20;PPP2CA;SPO11;PRKACA;PRKACB;CDC45;MTOR;PPP2R5A;PPP2R5B;PPP2R5C |
| Phototransduction - fly | MYO3A;MYO3B;ACTG1;MYO3A\_1;LOC102153034(down);DAGLB;TRPC4;CALM2;CALM3;CALM1;GRK2;GRK3;GNG13;CAMK2D;DAGLA;PLCB2;PLCB3;PLCB4;PRKCA;CAMK2G;PPEF2;CAMK2A;CAMK2B;PLCB1(up);ITPR1;GNAQ;CALML6;CALML5;CALML4;ACTB |
| Alanine, aspartate and glutamate metabolism | NIT2;FOLH1B(up);AGXT2;LOC612295;ABAT;ADSL;GLS;ASS1;GAD1;CAD;GLUL;GOT2;IL4I1;GOT1;ASL;ASPA(up);LOC482436;RIMKLA;RIMKLB;DDO;ALDH5A1;GPT;NAT8L;ALDH4A1;GPT2;AGXT;ASNS;GLUD1;PPAT;ADSS2;ADSS1;LOC483960;GFPT2;GFPT1;CPS1;GLS2 |
| PPAR signaling pathway | PCK2;CPT2;PCK1;UBC;ME3;HMGCS1;SCD;SCP2;CPT1C;CD36;FABP6;FABP7;FABP4;FABP5;ME1;FABP3;ACSBG2;FABP1;APOA5;PLIN4;LOC100683335;PLIN2;ACADM;APOA2;PLIN1;FADS2;LOC102153461;LOC608150;GK;GK2;APOA1;EHHADH;ACAA1;ADIPOQ;HMGCS2;CYP8B1;ACOX1;ACSL5(up);LOC485024;NR1H3;CPT1A;AQP7;LOC102155424;CPT1B;ACSL1;RXRG;PLIN5(down);RXRA;RXRB;ILK;LOC102154292;PPARG;PPARD;LPL;PPARA;SLC27A5;SLC27A4;SCD5;SLC27A6;SLC27A1;LOC106559802;SLC27A2;DBI;CYP7A1;APOC3;OLR1(up);SORBS1(up);ACSL3;CYP4A11(down);ACSL6;ACSL4;CYP27A1;ACOX2;ACADL;ANGPTL4;PLTP;ACOX3;PDPK1;MMP1;CYP4A38(down);UCP1;ACSBG1 |
| cAMP signaling pathway | LOC100856339;FXYD2;FXYD1;ADCY1\_1;MC2R;RAPGEF4;AKT1;AKT2;AKT3;RAPGEF3;BDNF;MYL9;CALM2;CALM3;CALM1;CNGB1;PIK3CA;PIK3CB;CACNA1C;CACNA1D;CAMK4;CACNA1F;PTGER3;GRIN3B;GLP1R;RELA;CACNA1S;HTR1D(down);NPR1;LIPE;GIP;CAMK2D;ATP1B4;CAMK2G;ATP1B2;ATP1B3;CREB1;ATP1B1;ADCY4(up);HTR4;ATP2B1;ATP2B3;ATP2B4;PLD1;ADCY5;ADCY6;ADCY7;ADCY1;ADCY2;ADCY3;NPY1R;ADCY8;ADCY9;F2R;VIP;NPY;GPR119;MAPK10;CFTR;HHIP;NFATC1;HCN2;EDN3;ATP2A1;ATP2A2;ADORA1;GRIA3;RRAS2;GRIA4;HCN4;RRAS;GABBR1;RAC3;LHCGR;PPP1R1B;SLC9A1;RAF1;RAC1;JUN;TIAM1;ATP1A4;PIK3R2;PIK3R1;PRKACA;GRIN2B;NPPA;LOC119869567;ORAI1;GCG;FOS;LOC119870217;LOC119870216;LOC119870215;OXTR;GNAI1(up);HCAR2;HCAR1;CNGA3;CNGA4;PDE4A;RHOA;ATP2B2(up);GLI1;RAC2(up);GNAS;VAV1;SST;VAV3;ACOX1;CALML6;CALML5;CALML4;EDNRA(up);GABBR2(up);CNGB3;CREBBP;RYR2;ATP2A3(down);GHRL;PIK3CD;PPARA;LOC102153034(down);RAP1B;RAP1A;PLCE1;PTGER2;ADCY10;CNGA1;GNAI2;GNAI3;GRIN1;CGA;PDE3A;EDN1(down);PDE3B;MAPK3;MAPK1;PPP1R12A;TSHB;CNGA2;NFKB1;MAPK8;MAPK9;SUCNR1;GRIA1;POMC;GHSR;EP300;PDE10A;EDN2;ACOX3;AFDN;BAD;SSTR1;ADRB1;SSTR2;SSTR5;ATP1A1;PRKACB;PLN;PDE4B;PDE4C;PDE4D;ADRB2(down);VAV2;GRIA2;MAP2K2;MAP2K1;SOX9;FSHB;TSHR;GIPR;ADCYAP1R1;PLD2;ADORA2A(up);ARAP3;GRIN2C;ATP1A3;GRIN3A;GLI3;GRIN2A;VIPR2;TNNI3;FSHR;CREB5;PAK1;NFKBIA;GRIN2D;AMH;CREB3;OXT;CHRM2;CHRM1;CAMK2A;CAMK2B;FFAR2;ADCYAP1;HTR6;PPP1CB;PPP1CC;PPP1CA;DRD5;DRD2;ROCK1;ROCK2;DRD1;CREB3L2;CREB3L3;CREB3L1;BRAF;CREB3L4;ABCC4;HTR1E;HTR1F;PTCH1;HTR1A;HTR1B |
| Natural killer cell mediated cytotoxicity | LOC100856339;RAET1E;PPP3R2;PPP3R1;FYN;IFNAR2;LOC607467;PTK2B;IFNAR1;ARAF;TYROBP;PIK3CA;PIK3CB;PIK3CD;LOC106559121;KRAS;SHC4;SHC1;SHC2;SHC3;IFNG;FASLG;PRF1;LOC119879788;LOC106559212;SYK;PTPN11;CSF2;LOC106559111;GRB2;LAT;PAK1;NFATC1;NFATC2;TNFSF10;HRAS;IFNGR1;IFNGR2;RAC3;PIK3R2;PIK3R1;LOC119876828;LOC100683481;NCR1;PTPN6;LOC100686162;LOC100683403;RAC2(up);GZMB;VAV1;VAV3;VAV2;ITGAL;LCK;SH2D1B;SH2D1A;LOC119866425;SH3BP2;RAF1;LOC611406;KLRD1;FCER1G;LOC102153980;LOC609192;IFNB1;MAPK3;MAPK1;ICAM1;LOC486692;LOC111097181;LOC119869025;ITGB2(down);SOS2;MAP2K2;IFNA7;PLCG1;IFNA5;LOC100683567;LOC609023;LOC478984;HCST;CD244;PLCG2;MAP2K1;CD247;FAS;BID;NRAS;ZAP70;CD48;RAC1;PRKCA;PRKCB;NCR3;PRKCG;TNF;LOC119869046;LCP2;CASP3;KLRK1;BRAF;PPP3CA;PPP3CB;PPP3CC;LOC102154760 |
| cGMP-PKG signaling pathway | FXYD2;PPP3R2;PPP3R1;ADCY1\_1;AKT1;AKT2;AKT3;ATP2B3;MYL9;CALM2;ADCY6;CALM1;CNGB1;CACNA1C;CACNA1D;ADRA2A;CACNA1F;ADRA2C;KCNU1;CACNA1S;NPR1;NPR2;ADCY7;CREB5;CREB3;ATP1B3;CREB1;ATP1B1;ADCY4(up);ADRB3;KCNMB2;ATP2B1;GATA4;ATP2B4;KCNMB4;ADRA1A;ADCY5;KCNMB3;ADRA1B;ADRA1D;ADCY2;ADCY3;ADCY8;ADCY9;GUCY1B1;ADCY1;NFATC1;NFATC2;NFATC3;NFATC4;ATP2A1;ATP2A2;ADORA1;ADORA3;MYLK;SLC25A31;LOC609798;EDNRB;GNAI2;EDNRA(up);KCNMA1;LOC488190;GUCY1A1;GUCY1A2;PRKG1;PRKG2;GTF2I;NPPB;NPPC;PDE5A;NPPA;LOC119869567;PLCB2;PLCB3;PLCB4;LOC119870217;LOC119870216;LOC119870215;GTF2IRD1;GNAI1(up);CNGA1;PPIF;ITPR2;ITPR3;RHOA;ITPR1;CALM3;GNAQ;CALML6;CALML5;CALML4;MEF2C;MEF2B;MEF2A;MEF2D;KCNMB1;AGTR1;ATP2A3(down);LOC102153034(down);IRS1;TRPC6;ADRA2B;VDAC3;VDAC2;VDAC1;PDE3B;RAF1;GNAI3;ATP2B2(up);MYH7;PDE3A;IRAG1;MAPK3;MAPK1;PPP1R12A;SLC8A1;SLC8A2;SRF;MYLK2;MYLK3;PLCB1(up);BDKRB2;IRS4;KCNJ8;BAD;IRS2;ADRB1;ATP1A4;ATP1A3;ATP1A1;PLN;ADRB2(down);INSR;MAP2K2;MAP2K1;LOC111096471(down);LOC485435;SLC8A3(up);INS;RGS2;ATP1B4;GNA13;NOS3;ATP1B2;PRKCE;SLC25A6;SLC25A5;PPP3CA;VASP;SLC25A4;PPP3CB;PPP1CB;PPP1CC;PPP1CA;KNG1;ROCK1;ROCK2;PDE2A;CREB3L2;CREB3L3;CREB3L1;GNA12;OPRD1;CREB3L4;GNA11;ATF4;ATF6B;PPP3CC;ATF2 |
| Calcium signaling pathway | AVPR1B;AVPR1A;PPP3R2;PPP3R1;LTB4R2;ADCY1\_1;PTGFR;ADRB2(down);LOC607467;PTK2B;TNNC1;TNNC2;LOC488190;ADRA1D;CACNA1H;CACNA1I;LOC111090226;CALM2;CALM3;CALM1;CAMK1;CACNA1A;RYR3;RYR2;CACNA1D;NTRK1;NTRK2;NTRK3;LOC106559121;ATP2B1;HRH2;GRIN1;LOC106559212;LOC102156003;ERBB2;ERBB3;ADCY7;ERBB4;CAMK2D;CAMK2G;CAMK2A;CAMK2B;SLC25A4;ADCY4(up);HTR7;HTR4;BDKRB2;LOC119879788;HTR5A;FGFR4;FGFR2;FGFR3;FGFR1;CHRNA7(down);ADRA1B;ADCY1;ADCY2;ADCY3;ADCY8;MYLK;LOC106559111;GRM1;F2R;CACNA1E;PTAFR;LOC102156776;ORAI2;NGF;ATP2B3;ATP2A1;ATP2A2;PDGFA;PTGER1;CCKAR;TRHR;ORAI1;SLC25A31;LOC609798;PDGFD;EDNRB;LOC106560021;CYSLTR2;FLT4;CYSLTR1;CCKBR;LHCGR;ADORA2B;PDGFC;ADRB1;FGF10;LOC102156901;EGF;ITPKA;ITPKC;ITPKB;HTR2A;HTR2C;HRH1;GRM5;LOC106559087;PHKG2;VEGFB;LOC119869567;ORAI3;PLCB2;PLCB3;PLCB4;PRKACB;PHKA1;LOC119872979;VEGFD;VEGFA;CHRM1;FGF9;PLN;ITPR2;ITPR3;FGF5;ITPR1;TACR3;TACR2;TACR1;ADRA1A;GNAQ;GNAS;CALML6;CALML5;CALML4;RYR1;GNAL;EDNRA(up);AGTR1;FGF22;CACNA1B;FGF20;PDGFRB;PDGFRA;CACNA1C;ATP2A3(down);VEGFC;HTR2B;PLCZ1;FGF8;LOC102153034(down);STIM2;RET;CAMK4;STIM1;FGF7;PLCE1;CACNA1F;FGF6;VDAC3;VDAC2;VDAC1;PTGER3;FLT1;ATP2B2(up);FGF4;LOC102153980;FGF3;FGF2;FGF18;FGF1;SLC8A1;FGF11;SLC8A2;FGF17;FGF16;KDR;TBXA2R;NOS2(up);ATP2B4;MYLK2;LOC111097181;PLCB1(up);PLCD4;PLCD3;PLCD1;NTSR1;LOC119869025;BDKRB1;CACNA1S;TPCN2;PLCG1;ADCY9;MET;ADRB3;PRKACA;OXTR;PPIF;SPHK2;SPHK1;CAMK1D;CAMK1G;LOC119869603;EGFR;LOC119869607;CD38(up);PLCG2;LOC102154760;P2RX1;SLC8A3(up);P2RX2;P2RX5;P2RX4;P2RX7;P2RX6;ADORA2A(up);PDE1C;PDE1B;PDE1A;PHKB;GNA15;LOC609053;NOS1;PDGFB;NOS3;CACNA1G;PRKCA;CHRM5;PRKCB;CHRM3;CHRM2;PRKCG;MYLK3;MCOLN1;SLC25A5;LOC119869046;HGF;GRPR;SLC25A6;HTR6;LOC480788;DRD5;LOC119863891(up);DRD1;GRIN2C;GNA14;GRIN2A;PHKG1(up);GRIN2D;GNA11;PPP3CA;PPP3CB;PPP3CC;P2RX3 |
| Spliceosome | HSPA2;NCBP1;NCBP2;HSPA8;LOC488929;PPIL1;PUF60;DHX16;SRSF10;DHX15;PRPF3;PRPF4;PRPF6;SNRNP200;DDX39B;PRPF31;LOC119870156;CCDC12;MAGOH;DHX8;HNRNPU;LOC119865916;PCBP1;LOC475399;USP39;LOC102152884;THOC2;THOC1;CWC15;SNRPD3;SNRPD2;SNRPD1;PRPF8;DDX42;PQBP1;DDX46;DDX5;SNRNP27;LOC119874304;LOC102153257;LOC608314;LOC100856126;LOC102154724;NCBP2L;LOC607182;DHX38;LOC102151489;LOC480188;SRSF5;SRSF4;SRSF7;SRSF6;SRSF1;KBTBD2;SRSF3;SRSF2;U2AF1;LOC609886;U2AF2;LOC490565;LOC102154080;EFTUD2;RP9;LOC100686524;SF3A1;SF3A2;SF3A3;ALYREF;LOC119870192;LOC102154981;SART1;SNRPB2;MAGOHB;XAB2;RBM25;RBM22;BUD31;PHF5A;LOC474850;EIF4A3;LOC102153757;LOC491817;LOC102153759;TCERG1;PRPF40B;LOC100684102;BCAS2;LOC608729;LOC100687756;LOC609245;RBM8A;PLRG1;SNRPA1;PRPF40A;HNRNPK;RBM17;HNRNPM;LOC100687510;LOC119869303;SMNDC1;HNRNPA3;HNRNPA1;U2SURP;WBP11;LOC100687940;LOC111098753;LOC111098242;CRNKL1;CTNNBL1;ZMAT2;ISY1;PPIH;ACIN1;PPIE;CDC40;LOC490173;LOC119866566;SF3B5;SF3B4;SF3B6;SF3B1;SF3B3;SF3B2;LOC102154571;RBMXL2;TXNL4A;HSP70;LOC119873859;LOC119881680;AQR;PRPF19;PRPF18;LOC610488;LOC608117;LOC478183;LOC478184;TRA2A;TRA2B;CDC5L;SNU13;LSM8;LSM4;LSM5;LSM6;LSM7;LSM2;LSM3;U2AF1L4;SNRPB;SNRPC;SNRPA;SNRPF;SNRPG;SNRPE;DDX23;SNRNP40;SNW1;PRPF38A;PRPF38B;SLU7;RBMX;CHERP |
| Antifolate resistance | DHFR;LOC100684665;IKBKB;ALOX12;IL1B;LOC609612;NFKB1;RELA;GGH;MTHFR;CHUK;IZUMO1R;LOC476816;FPGS;GART;SHMT2;SHMT1;ABCG2;ATIC;IL6;TYMS;TNF;ABCC2;LOC609621;ABCC4;ABCC5;SLC19A1;ABCC1;LOC609048;ABCC3 |
| Th1 and Th2 cell differentiation | PPP3R2;PPP3R1;IKBKB;TYK2;LOC111090226;IL12RB1;RBPJ;IL12RB2;LOC102156776;RELA;LOC102156003;LOC100856137;IFNG;HLA-DRB1;CD4;LAT;CD3D;CD3E;CD3G;NFATC1;NFATC2;NFATC3;NFKBIB;NFKBIA;IL12A(up);NFKBIE;IFNGR1;IFNGR2;MAML2;MAML1;JUN;LOC102156901;RBPJL;JAK2;JAK3;JAK1;IL4R;FOS;CHUK;LOC119872979;LOC119863905;LCK;MAPK14;MAPK10;MAPK11;MAPK12;MAPK13;LOC106559087;STAT6;STAT4;STAT1;MAPK3;MAPK1;NFKB1;MAPK8;MAPK9;IL12B;DLL1;DLL3;DLL4;IL2RA;IL2RB;IL2RG;DLA-DOA;DLA-DOB;IL13;IL4;STAT5B;STAT5A;IL5;IL2;LOC119869603;LOC119869607;PLCG1;CD247;ZAP70;LOC609053;JAG1;LOC119881611;PRKCQ;LOC480788;NOTCH1;DLA-DMB;DLA-DMA;PPP3CA;PPP3CB;PPP3CC;DLA-DRA(down) |
| Th17 cell differentiation | PPP3R2;PPP3R1;IL6ST;IKBKB;IL22;TYK2;LOC111090226;IL12RB1;RORC;LOC102156776;HSP90AB1;RELA;LOC102156003;LOC100856137;IFNG;HLA-DRB1;CD4;LAT;CD3D;CD3E;CD3G;NFATC1;NFATC2;NFATC3;IL21;NFKBIB;NFKBIA;HIF1A;NFKBIE;IFNGR1;IFNGR2;IL1R1;JUN;LOC102156901;JAK2;JAK3;JAK1;IL4R;FOS;CHUK;LOC119872979;LOC612100;LCK;SMAD4;SMAD2;SMAD3;MAPK14;MAPK10;MAPK11;MAPK12;MAPK13;IL1B;LOC100687242;RORA(up);LOC106559087;STAT6;STAT3;STAT1;MAPK3;MAPK1;NFKB1;MAPK8;MAPK9;IL17D;TGFBR2;TGFBR1;IL17A;RXRG;RXRA;RXRB;IL6R;MTOR;IL2RA;IL2RB;IL2RG;DLA-DOA;DLA-DOB;IL21R;IL4;IL6;STAT5B;STAT5A;IL2;HSP90AA1;TGFB1;IL23A;LOC119869603;LOC119869607;PLCG1;CD247;IL23R;ZAP70;LOC609053;LOC119881611;IL1RAP;PRKCQ;LOC480788;IL27RA;DLA-DMB;DLA-DMA;PPP3CA;PPP3CB;PPP3CC;DLA-DRA(down) |
| Signaling pathways regulating pluripotency of stem cells | LIF;LOC100856339;ISL1;IL6ST;AKT1;AKT2;AKT3;LIFR;LHX5;WNT8A;WNT10A;WNT10B;PIK3CD;INHBA;KLF4(up);NODAL;MYC;KRAS;SETDB1;JAK3;IGF1R;STAT3;PCGF2;ZFHX3;FGFR4;FGFR2;FGFR3;FGFR1;PCGF1;PCGF3;BMP4;PCGF5;PCGF6;GSK3B;GRB2;BMPR2;APC;WNT7A;ID4;WNT7B;ZIC3;ACVR1C;ACVR1B;HESX1;HRAS;WNT6;LOC481709;FZD10;ID1;OTX1;MEIS1;JAK2;PIK3R2;PIK3R1;JAK1;SMARCAD1;NEUROG1;TCF7;IGF1;TCF3;ID3;LOC119870217;LOC119870216;LOC119870215;LOC490387;PAX6;NANOG;FGF2;LOC119863906;PIK3CA;WNT5B;WNT5A;PIK3CB;WNT3A;SMAD9;SMAD4;SMAD5;ONECUT1;SMAD1;SMAD2;SMAD3;MAPK14;TBX3;MAPK11;MAPK12;MAPK13;RAF1;FZD1;FZD2;FZD3;FZD4;FZD6;FZD7;FZD8;FZD9;MAPK3;MAPK1;LOC102151783;DUSP9;ACVR2A;ACVR2B;RIF1;BMPR1A;WNT3;WNT2;WNT1;ID2;BMPR1B;WNT4;ACVR1;MYF5;JARID2;CTNNB1;MAP2K2;MAP2K1;HAND1;SOX2;AXIN2;AXIN1;NRAS;DVL2;DVL3;WNT8B;DVL1;DLX5(up);WNT2B;ESRRB;BMI1;REST;SKIL;APC2;WNT9B;WNT9A;WNT16;KAT6A;WNT11 |
| PI3K-Akt signaling pathway | THBS2;STK11;IFNAR2;IFNAR1;PIK3CA;PIK3CB;PIK3CD;LOC119863873;IGF1R;PPP2R2D;PPP2R2A;PPP2R2C;CSF3R;LOC106559212;CCND1;CCND3;CCND2;PTEN;ITGA8;ITGA9;CDKN1B;ITGA2;ITGA3;ITGA4;ITGA5;ITGA6;ITGA7;BCL2L1(down);MTCP1;JAK2;JAK3;JAK1;EREG;CHUK;VEGFD;COL4A5;COL4A4;COL4A3;COL4A2;VEGFC;VEGFB;TLR4(down);ITGAV;INS;VWF;CD19;AREG(up);TSC2;PIK3AP1;TSC1;ITGB8(down);LOC612100;LOC102153980;CDC37;LAMA1;LAMA2;LAMA3;LAMA5;RXRA;IL3RA;IL2RA;IL2RB;IL2RG;INSR;SPP1;BRCA1;EGFR;FLT3(up);BAD;PGF;ITGA10(up);ITGB1;RPTOR;ITGB3;NOS3;ITGB5;ITGB4;ITGB7;ITGB6;LPAR1(up);LOC100685582;HGF;TCL1B;GNB2;EIF4E2;PPP2R2B(up);PCK2;PCK1;GHR;PDGFRB;PPP2R3B;PPP2R3C;PPP2R3A;IKBKB;MDM2;MYB(up);GNG14;PRL;GNG10;GNG11;GNG12;GNG13;RELN;LOC100855903;RELA;RPS6KB1;KITLG;LOC479459;GH1;FN1;CSF1;CSF3;GNG7;GNG4;GNG5;GNG2;GNG3;GNG8;NGF;LAMB1;LAMB3;LAMB2;IBSP;LAMB4;CCNE2;CCNE1;ANGPT2;LOC119876828;ANGPT1;ANGPT4;IGF1;EIF4E;EIF4B;EPOR;LOC100686162;LOC100683567;LOC100686736;IL7R;PDGFRA;CHAD;GNB4(up);FLT4;LOC100687242;LOC611406;FLT1;ITGA2B;CSF1R;RPS6KB2;FGF18;ITGA1;CDKN1A;NTF3;GNB5;GNB1;TCL1A;GNB3;VTN;MTOR;HSP90B1;LOC612475;LOC119869025;IRS1;CDK2;CRTC2;CDK4;CDK6;EIF4E1B;LPAR2;LPAR3;LPAR6;LPAR4;LPAR5;PTK2;PRKAA2;PRKAA1;LOC102154760;MCL1;YWHAZ;YWHAQ;YWHAH;YWHAB;YWHAG;YWHAE;RAC3;RAC1;PRKCA;CHRM2;CHRM1;LOC111097181;IL6R;COL6A3;LOC608772;EPO;BCL2;LOC100856339;MLST8;LOC106559121;COL1A2(up);MAGI2;MAGI1;ERBB2;ERBB3;ERBB4;CREB5;CREB3;CREB1;FGFR4;FGFR2;FGFR3;FGFR1;LOC607467;SYK;LOC610614;KIT;F2R;HRAS;ITGA11;THEM4;PRLR;EGF;LOC119870217;LOC119870216;LOC119870215;LAMC2;LAMC3;LAMC1;TP53;COL4A6;EIF4EBP1;PPP2R5D;VEGFA;FGF22;PPP2R5A;FGF20;PPP2R5C;COL4A1;RPS6;RAF1;IFNB1;KDR;COMP;EFNA5;EFNA4;EFNA3;EFNA2;EFNA1;FLT3LG;SGK3;SGK2;SGK1;IL6;IL7;IL4;IL2;IL3;COL1A1;RHEB;LOC106559872;PPP2R1A;PPP2R1B;FOXO3;MAP2K2;MAP2K1;PKN1;PKN3;PKN2;PPP2R5E;CASP9;PPP2R5B;COL6A1;ATF6B;COL6A2;COL6A5;COL6A6;AKT1;AKT2;AKT3;BDNF;NR4A1(up);NTRK1;NTRK2;HSP90AB1;MYC;DDIT4;FASLG;LOC119879788;KRAS;PPP2CA;PPP2CB;LOC106559111;TLR2;LOC609669;G6PC1;G6PC3;G6PC2;GSK3B;TGFA;COL2A1;LOC100855681;TNXB;THBS3;THBS1;GRB2;OSM;PIK3R2;PIK3R1;IL4R;GYS1;GYS2;FGF9;FGF8;FGF7;FGF6;FGF5;FGF4;FGF3;FGF2;FGF1;LOC119869046;EPHA2;TEK;MAPK3;MAPK1;NFKB1;FGF11;FGF10;FGF17;FGF16;RBL2;COL9A2;COL9A3;COL9A1;PHLPP2;PHLPP1;SOS2;IFNA7;IFNA5;MET;NGFR(up);HSP90AA1;LOC491854;BCL2L11;NRAS;OSMR;PDGFA;PDGFB;PDGFC;PDGFD;GNGT1;TNR;TNC;PDPK1;TNN;LAMA4(up);CREB3L2;CREB3L3;CREB3L1;GNGT2;CREB3L4;ATF4;LOC608573;THBS4;ATF2 |
| O-Antigen nucleotide sugar biosynthesis | TGDS;MPI;UGDH;GALE;GMDS;GFUS;UGP2 |
| Endocytosis | HSPA2;WASHC1;ARFGEF2;DLA-64;WWP1;ACAP1;HSPA8;CHMP2B;ZFYVE9;CHMP2A;CAPZA1;LOC607182;DAB2;CAV2;FGFR4;CAV1;SMAP1;SMAP2;WASHC4;SPG21;GRK6;GRK7;GRK4;GRK5;GRK2;PARD6B;PARD6A;GRK1;PSD3;PSD2;RAB11B;PSD4;VPS35;RAB11FIP3;IGF1R;RAB5A;LOC119873859;RAB5C;RAB5B;DLA-79;ARFGAP1;SH3GLB1;IZUMO1R;VPS26A;MDM2;SMAD3;FGFR2;FGFR3;ARPC5;IL2RG;PLD1;PLD2;IST1;KIF5A;KIF5B;RBSN;ARPC1B;PARD6G(up);GBF1;ARPC1A;TSG101;CAPZB;SNX1;KIF5C;SNX6;SNX5;SNX4;HRAS;WASHC3;DNM1;ARFGAP3;DNM3;CLTB;IQSEC1;IQSEC3;STAM;AP2B1;SMURF1;AP2S1;SMURF2;VPS26B;CHMP7;VPS37D;VPS37C;VPS37B;VPS37A;CAV3;RAB11A;TFRC;SH3GL3;ARPC2;SNX32;VTA1;WASHC5;RAB7A;IL2RB;ARPC3;DLA88;EPN1;RNF41;EPN2;EPN3;EPS15L1;RHOA;ARPC4;SNX12;AMPH;DNAJC6;CBL;ASAP1;GIT1;GIT2;LOC474850;SNX3;PDGFRA;EHD4;EHD3;EHD2;EHD1;SNX2;ZFYVE16;SMAD2;GRK3;ASAP3;ARRB2;CCR5;ARRB1;CHMP3;IGF2R;SH3GL1;CHMP6;CHMP5;SH3GL2;VPS45;RAB8A;SPART;ARF3;ARF1;NEDD4;ARF6;ARF5;AP2M1;RAB22A;RABEP1;PML;RAB10;AGAP2;SRC;TGFBR1;LOC100684665;AGAP1;LDLR;AGAP3;VPS4A;VPS4B;MVB12B;CHMP1B;CHMP1A;MVB12A;LOC119866928;CAPZA3;RAB11FIP2;RAB11FIP1;ZFYVE27;RAB11FIP5;VPS36;PIP5K1C;PIP5K1B;PIP5K1A;ARPC5L;LOC609621;RAB4A;CLTA;LDLRAP1;ASAP2;CDC42;USP8;LOC102151523;CLTC;ITCH;TGFBR2;CHMP4A;CHMP4B;CHMP4C;EGFR;PSD;RAB11FIP4;HSP70;BIN1;EPS15;RAB35;IQSEC2;ARAP3;ARAP2;ARAP1;VPS25;EEA1;LOC609612;STAM2;VPS29;IL2RA;CXCR2;CXCR1;PIP5KL1;CAPZA2;PRKCI;TRAF6;RUFY1;HGS;ACAP2;ACAP3;LOC100684058;PRKCZ;AP2A1;SNF8;WASHC2C;AP2A2;CYTH1;CLTCL1;CYTH3;CYTH2;CYTH4;LOC612266;PARD3;RAB31;LOC119863891(up);PDCD6IP;NEDD4L;ARFGAP2;LOC476816;WIPF3;WIPF2;WIPF1;VPS28;ARFGEF1 |
| Mismatch repair | RFC5;RFC4;MSH2;MSH3;RFC1;RFC3;RFC2;EXO1;MLH3;MLH1;POLD1;POLD2;POLD3;LIG1;PCNA;MSH6;SSBP1;PMS2;LOC611847;RPA1;RPA3;RPA2 |
| Peroxisome | PECR;AGPS;ACOT8;ACSL3;PAOX;PMVK;CROT;SCP2;EPHX2;PRDX1;CAT;PEX19;MPV17;IDH1;MPV17L2;SLC25A17;PEX10;PEX13;PEX12;PEX14;PEX16;PIPOX;PXMP4;DHRS4;SOD1;DAO(down);XDH;HAO1;ACAA1;HMGCLL1;ABCD1;ABCD3;LOC477770;HSD17B4;ABCD4;LOC478000;CRAT;NOS2(up);ACSL1;SOD2;MPV17L;PEX26;ACSL6;HMGCL;IDH2;NUDT19;LOC608697;FAR2;PRDX5;LOC100686190;PEX11A;AMACR;PEX11B;DDO;PEX11G;GNPAT;PXMP2;MVK;EHHADH;LOC106558006;NUDT7;LGALS4;PEX1;NUDT12;PEX3;PEX2;PEX5;SLC27A2;PEX7;PEX6;ACOX2;PEX5L;ACOX1;AGXT;ACSL4;DECR2;ECI2;ACSL5(up);FAR1;ACOX3;MLYCD;HAO2;ABCD2;BAAT;GSTK1 |
| Nitrogen metabolism | CA5B;CA5A;CA8;CA4(up);CA13;CA1;CA14;CA7;CA6;GLUL;CA12;CA9;LOC612295;GLUD1;LOC100686148;CA15;CPS1 |
| Autophagy - animal | LOC100856339;STK11;LOC100682891;AKT1;AKT2;AKT3;PIK3CA;PIK3CB;PIK3CD;MAP3K7;GABARAP;PIK3R4;MTMR4;MTMR3;IGF1R;DDIT4;CTSB;CTSD;SH3GLB1;PIK3C3;CTSV;KRAS;PPP2CA;PPP2CB;SNAP29;PTEN;HRAS;RRAS2;HIF1A;IRS1;RRAS;IRS2;BCL2L1(down);TANK;RRAGC;TSC2;AKT1S1;PIK3R2;PIK3R1;RAB7A;BECN1;BECN2;LOC119870217;LOC119870216;LOC119870215;MRAS;ATG10;ATG12;ATG13;ATG14;ITPR1;BNIP3;DEPTOR;INS;CAMKK2;LOC609805;MAPK10;RAF1;TSC1;RAB8A;TBK1;CFLAR;RPS6KB1;RPS6KB2;MAPK3;MAPK1;MAPK8;MAPK9;ERN1;MTMR14;RAB7B(up);MTOR;LAMP2;LAMP1;MLST8;IRS4;EIF2AK3;EIF2AK4;BAD;PRKACA;STX17;PRKACB;RHEB;ATG101;HMGB1;PRKAA2;PRKAA1;MAP2K2;MAP2K1;LOC111096471(down);RB1CC1;EIF2S1;RPTOR;GABARAPL1;GABARAPL2;ATG16L1;NRAS;ATG3;ATG7;ATG5;ULK2;RRAGD;RRAGA;TRAF6;RRAGB;PRKCD;ATG4C;ATG4B;ATG4A;ATG4D;PRKCQ;SQSTM1;VAMP8;PDPK1;BCL2 |
| Protein processing in endoplasmic reticulum | RPN1;RPN2;UBE2J1;HSPA5;TUSC3;SEC24B;LOC607182;TXNDC5;CANX;UBE2J2;DNAJB12;STUB1;NFE2L2;DNAJB11;MAP3K5;HSP90AB1;CAPN2;CAPN1;LOC119863874;UFD1;CUL1;DDIT3;LOC119873859;ERLEC1;HSPA8;SEC24D;EDEM2;EDEM3;SEC24C;EDEM1;MAN1A1;MAN1A2;DNAJA2;UBE4B;MBTPS1;RNF185;BAK1;SEC24A;RBX1;WFS1;LOC102154649;UBE2D2;UBE2D3;SEC62;SEC63;PREB;NGLY1;TRAM1;HSPH1;LOC100687242;DERL1;DERL2;DERL3;DNAJC10;DAD1;OS9;HSPBP1;DNAJC5B;ERP29;SEC13;HSPA4L;DNAJC5G;STT3B;STT3A;LOC612100;RNF5;LOC100688277;MOGS;HYOU1;DNAJC1;DNAJC3;ATXN3;LOC102152482;BAG2;GANAB;BAG1;RAD23B;RAD23A;LOC487024;FBXO2;PRKCSH;MAPK10;FBXO6;NPLOC4;MARCHF6;NSFL1C;HSPA2;SEC23A;SEC23B;SKP1;DNAJB2;DNAJB1;LOC106559577;SYVN1;MAPK8;MAPK9;UGGT2;ERN1;UGGT1;SEL1L;UBXN6;PRKN;AMFR;HSP90B1;SAR1A;SAR1B;UBE2D1;BCAP31;SIL1;LOC100687064;EIF2AK1;EIF2AK3;EIF2AK2;ERO1B;EIF2AK4;ERO1A;PPP1R15A;HSP90AA1;LOC474850;CRYAB;LOC119876190;BAX;CRYAA;MAP2K7;SVIP;HSP70;EIF2S1;SEC31B;SEC31A;XBP1;SEL1L2;HERPUD1;SSR1;UBQLN2;UBQLN3;UBQLN1;UBQLN4;UBE2G2;UBE2G1;P4HB;TRAF2;LOC480667;PDIA6;PDIA4;PDIA3;VCP;MAN1C1;LMAN1;RRBP1;LMAN2;SEC61G;PLAA;SEC61B;LOC111090648;YOD1;LMAN1L(up);CALR;SELENOS;SSR2;SSR3;SSR4;CKAP4;ATF4;ATF6B;ATF6;SEC61A1;SEC61A2;BCL2 |
| Lysosome | MAN2B1;GNPTAB;AP1M1;AGA;LIPA;NAPSA;PPT1;PPT2;AP1M2;CLN5;CLN3;ATP6V0D1;GUSB;CTSH;ENTPD4;CTSO;CTSA;CTSB;CTSC;CTSD;CTSE;CTSF;CTSG;CTSZ;CTSS;LOC119876012;CTSV;CTSW;MANBA;HYAL3;SCARB2;HYAL1;GM2A;AP1B1;HYAL2;AP3M1;LITAF;GBA;ATP6V1H;CLTA;CLTC;CLTB;AP4B1;DNASE2B;SUMF1;AP4S1;NPC2;NPC1;SPAM1;PLA2G15;LGMN;AP1G1;AP1G2;PSAP;ATP6V0A4;ATP6V0A1;ATP6V0A2;SORT1;LOC100684998;ACP2;IDS;AP3M2;LAPTM5(up);SMPD1;HGSNAT;IGF2R;ACP5(down);AP3D1;ATP6V0B;ATP6V0C;CTSK(up);CD63;LOC609321;NAGLU;CD68;GGA1;GGA3;GGA2;LAMP3;LAMP2;LAMP1;GALC;IDUA;SLC17A5;GLB1;LOC119871594;ABCA2;SGSH;NEU1;GNS;NAGPA;LAPTM4A;LAPTM4B;AP3S2;ATP6V0D2(up);AP3S1;AP1S1;AP1S2;AP3B1;TCIRG1;AP3B2;ASAH1;DNASE2;ABCB9;HYAL4;GAA;MFSD8;WDFY1;GNPTG;TPP1;GALNS;AP4E1;MCOLN1;NAGA;CLTCL1;CTNS;ARSG;ARSA;ARSB;HEXA;HEXB;CD164;FUCA1;FUCA2;M6PR |
| Steroid hormone biosynthesis | HSD17B12;CYP2D15;LOC480777;SRD5A1;UGT2A3;CYP17A1;UGT1A6(down);LOC102154742;AKR1C3;CYP11A1;CYP1B1;LOC102154822;CYP2B6;CYP7B1(up);HSD17B8;SRD5A3;HSD17B1;HSD17B2;HSD17B3;HSD11B2;LOC106560171;HSD17B6;HSD17B7;SULT2B1;CYP21A2;COMT;STS;LOC489851;CYP1A1(up);LOC482182;CYP7A1;CYP19A1;AKR1D1;SRD5A2;CYP2E1;CYP1A2(up);CYP2C18;HSD3B2;LOC100688697;CYP11B2;HSD11B1 |
| Intestinal immune network for IgA production | TNFSF13;TGFB1;CD40LG;LOC119869046;LOC111097181;LOC119869603;ITGA4;LOC119869607;LOC102156776;TNFRSF17;LOC102154760;LOC100856137;PIGR(down);CCR9;LOC106559111;LOC106559087;LTBR;DLA-DMB;LOC111090226;ICOSLG;LOC102153980;CD80;LOC102156901;CD86;CCL28;LOC106559121;DLA-DMA;LOC609053;MADCAM1;MAP3K14;TNFSF13B;CCL25;CCR10;LOC102156003;LOC119869025;CD28;ITGB7;LOC119881611;HLA-DRB1;LOC119872979;CD40;LOC119879788;CXCL12;TNFRSF13B;TNFRSF13C;LOC106559212;LOC607467;LOC480788;DLA-DOA;DLA-DOB;LOC119863891(up);IL15;AICDA;IL6;IL10;IL4;IL5;IL2;ICOS;IL15RA;DLA-DRA(down) |
| Vitamin B6 metabolism | AOX2;PDXK;AOX4;PSAT1;PNPO;PDXP;PHOSPHO2 |
| Thermogenesis | LOC100683828;LOC100682956;ACTG1;NDUFB9;NDUFB8;LOC100855618;NDUFB6;PRKAG3;ADCY1\_1;NDUFB3;NDUFB2;NDUFB1;UQCRQ;LOC102151856;LOC119866697;LOC100685227;UQCRH;LOC100688835;LOC100683222;PLIN1;NDUFAB1;LOC102152785;NDUFA4L2(down);CPT2;NDUFB11;MAP3K5;PRKAG1;LOC119872525;LOC100684983;NDUFA10;NDUFA11;NDUFA12;NDUFA13;LOC610725;KRAS;NDUFB5;NPR1;PRKAG2;LIPE;CREB5;CREB3;ATP6;CREB1;ADCY4(up);LOC100685720;LOC119874008;FGFR1;ADCY5;ADCY6;ADCY7;ADCY1;ADCY2;ADCY3;ACSL3;ACSL1;ACSL6;ADCY9;ACSL4;GRB2;LOC119864666;ACTB;LOC102151506;UQCRC2;UQCRC1;LOC100685007;LOC476372;LOC119868043;MAPK12;ATP5J2;NDUFC1;ADCY8;HRAS;NDUFC2;FRS2;LOC119870901;NDUFB7;COX7B2;LOC102153059;PPARGC1A;LOC477508;PNPLA2;AKT1S1;PRKG1;PRKG2;LOC119870103;LOC100684842;NPPB;LOC102154486;NPPA;LOC119869567;NDUFV3;PRKAB2;GCG;PRKAB1;COX8A;PPARG;COX7A1;LOC111096466;UQCRFS1;ATP8;RPS6KA1;RPS6KA2;ATP5PO;RPS6KA6;GNAS;ATP5F1D;ATP5F1E;ATP5F1A;ATP5F1B;LOC102155410;NDUFB4;LOC100686736;LOC100684996;COX10;COX11;COX15;COX17;LOC102156885;LOC119866701;LOC102154025;MAPK14;RPS6;MAPK11;ADCY10;MAPK13;NDUFB10;TSC2;TSC1;CNR1;NDUFV2;NDUFV1;LOC100855914;RPS6KB1;LOC100686500;RPS6KB2;ND1;LOC609990;ND3;ND2;ND5;ND4;ND6;NDUFA6;NDUFA7;NDUFA4;NDUFA5;NDUFA2;NDUFA3;NDUFA1;LOC100688796;NDUFA8;NDUFA9;COX4I2;COX4I1;MTOR;PRKAA1;LOC100683076;MLST8;LOC100684768;ACSL5(up);LOC106557778;LOC100682717;SOS2;LOC100686510;ND4L;ADRB3;PRKACA;PRKACB;RHEB;LOC102154590;LOC100855425;LOC106559872;LOC487689;LOC102151754;COX5A;COX5B;UQCR10;UQCR11;LOC102156968;MGLL;PRKAA2;LOC491854;MAP2K3;LOC607768;LOC102154615;ATP5MC1;LOC100686889;ATP5F1C;LOC119865581;ATP5MC3;RPTOR;LOC119863903;ATP5MC2;LOC111092022;LOC119870484;NDUFS1;COX2;COX3;CYTB;COX1;NRAS;NDUFS3;LOC608048;ATP5MG;ATP5ME;LOC102156642;CPT1A;LOC100688724;CPT1C;CPT1B;NDUFS6;LOC612644;SDHA;UCP1;LOC102154372;COX7A2L;LOC608772;COX6A2;LOC100686830;CYC1;CREB3L2;CREB3L3;NDUFS2;CREB3L1;NDUFS4;NDUFS5;CREB3L4;NDUFS7;NDUFS8;SDHC;SDHB;SDHD;ATF2 |
| Serotonergic synapse | PTGS2;PTGS1;SLC6A4;ARAF;RAPGEF3;GNG14;CACNA1A;CACNA1B;CACNA1C;GNG10;GNG11;CACNA1F;GNG13;LOC119863873;PRKACB;GNB4(up);CACNA1S;HTR1D(down);ALOX15B(up);ALOX5;PLA2G4E(up);HTR6;HTR7;HTR4;KCNN2;KRAS;ADCY5;APP;GNG7;GNG4;GNG5;GNG2;GNG3;LOC488101;GNG8;HTR5A;GNAO1;HRAS;RAF1;LOC100855681;HTR2A;HTR2C;HTR2B;PRKACA;PLCB2;PLCB3;PLCB4;HTR3A;GNAI1(up);DDC;ITPR3;ITPR1;GNAQ;GNAS;MAOB;MAOA;LOC478649;SLC18A2;SLC18A1;CYP2J2;LOC100856260;CACNA1D;ITPR2;TRPC1;GNG12;CYP4X1(down);GNAI2;GNAI3;MAPK3;MAPK1;BRAF;ALOX12B;GNB5;GNB1;GNB3;GNB2;PLCB1(up);LOC607881;GABRB1;GABRB2;GABRB3;KCNJ3;KCNJ6;KCNJ5;KCNJ9;MAP2K1;LOC106560021;HTR3B;HTR3C;LOC100688697;CYP2D15;ALOX15;ALOX12;NRAS;CYP2C18;KCND2;PRKCA;PRKCB;PRKCG;TPH1;TPH2;PLA2G4B;PLA2G4A;PLA2G4F;PLA2G4D;CASP3;GNGT2;GNGT1;HTR1E;HTR1F;HTR1A;HTR1B |
| Biofilm formation - Escherichia coli | PYGB;PYGL;PYGM |
| Lysine biosynthesis | AADAT |
| Wnt signaling pathway | PPP3R2;PPP3R1;INVS;RYK;WNT8A;WNT10A;WNT10B;MAP3K7;LOC479600;CSNK1E;MYC;CUL1;SFRP5;APC;CAMK2D;DAAM1;DAAM2;CAMK2G;CAMK2A;CAMK2B;BAMBI(up);CSNK2A2;CSNK2A1;NOTUM;CCND1;CCND3;CCND2;TCF7L1;TCF7L2;GSK3B;CTBP1;CTBP2;WNT7A;WNT7B;NFATC1;NFATC2;NFATC3;NFATC4;PLCB2;WIF1;VANGL1;FZD10;RUVBL1;CHD8;JUN;LOC106557930;SOX17;NLK;TCF7;PLCB3;PLCB4;CXXC4;SENP2;PPARD;RBX1;RHOA;RAC2(up);CSNK1A1;SKP1;FRAT1;LOC119863906;WNT5B;WNT5A;CREBBP;WNT3A;SOST;SMAD4;FBXW11;MAPK10;FZD1;FZD2;FZD3;FZD4;FZD6;FZD7;FZD8;FZD9;PSEN1;FOSL1;MAPK8;MAPK9;NKD1;NKD2;SIAH1;PLCB1(up);SERPINF1;TP53;LEF1;EP300;SFRP2;PORCN;SFRP1;LRP6;TBL1XR1;SFRP4;LRP5;WNT3;WNT2;WNT1;WNT6;WNT4;PRKACA;PRKACB;CSNK2B;CTNNB1;CER1;CACYBP;VANGL2;PRICKLE1;PRICKLE2;PRICKLE3;PRICKLE4;AXIN2;AXIN1;DVL2;DVL3;WNT8B;DVL1;WNT2B;RAC3;RAC1;PRKCA;PRKCB;PRKCG;GPC4;PPP3CA;APC2;PPP3CB;TBL1X;DKK4;BTRC;DKK2;ROCK2;DKK1;WNT9B;WNT9A;WNT16;MMP7;LOC111090648;PPP3CC;WNT11 |
| Citrate cycle (TCA cycle) | PCK2;PCK1;LOC102152275;CS;MDH2;MDH1;FH;IDH3A;OGDH;IDH3B;IDH3G;PC;PDHB;OGDHL;PDHA2;DLD;IDH2;IDH1;SUCLG2;SUCLG1;LOC119866771;ACO1;ACO2;SDHC;DLST;SUCLA2;DLAT;SDHA;ACLY;LOC102151507;SDHB;SDHD |
| Tryptophan metabolism | AOC1;AOX2;IDO1;AOX4;KMO;CAT;ALDH1B1;HAAO;ECHS1;AANAT;GCDH;KYNU;CYP1B1;ASMT;HADHA;AFMID;MAOA;ACAT1;ACAT2;KYAT1;IL4I1;KYAT3;TDO2;AADAT;ALDH2;CYP1A1(up);LOC482436;TPH1;TPH2;ALDH9A1;ALDH7A1;DDC;EHHADH;ACMSD;HADH;DLST;IDO2(down);DLD;MAOB;CYP1A2(up) |
| Arginine biosynthesis | NOS1;NOS2(up);NOS3;NAGS;GPT2;ARG1;ARG2;OTC;GLUL;GLUD1;GOT2;LOC612295;GOT1;GPT;LOC476602;GLS;ASS1;CPS1;GLS2;ASL |
| Hematopoietic cell lineage | CSF3R;CD34;IL1R2(up);CD36;CD37;EPO;FCGR1A;MS4A1;DNTT;LOC106559121;GP9;LOC100856137;CD22;HLA-DRB1;CD24;LOC119879788;KITLG;LOC106559212;LOC100687704;LOC607467;FCER2;CSF1;CSF2;KIT;CD7;CD1A8;CD1A6;CD3D;CD3E;CD3G;IL7R;CD1D;CSF2RA;CD1B;CD1C;ITGA1;ITGA2;ITGA3;ITGA4;ITGA5;ITGA6;IL1A(up);LOC106559111;IL1R1;TFRC;IL5RA;IL4R;EPOR;CD14;ANPEP;IL11(up);IL11RA;CD19;CSF1R;CD4;CD5;CD1E;CD2;IL1B;LOC490269;CD9;LOC102153980;ITGA2B;GP1BA;GP1BB;LOC111097181;IL6R;IL3RA;CD8A;CD8B;LOC608848;LOC119869025;IL2RA;LOC100686511;DLA-DOA;DLA-DOB;IL6;IL7;IL4;IL5;IL3;MME;CD55;CD59;FLT3(up);CD38(up);LOC102154760;CSF3;LOC119863892;THPO;ITGAM;ITGB3;CD44;LOC119881611;TNF;LOC119869046;DLA-DMB;DLA-DMA;FLT3LG;DLA-DRA(down) |
| Estrogen signaling pathway | HSPA2;LOC100856339;GNAI2;ADCY5;ADCY1\_1;GPER1;AKT1;AKT2;AKT3;LOC607182;CALM2;CALM3;CALM1;PIK3CA;PIK3CB;PIK3CD;HSP90AB1;MMP2(down);SHC4;HSPA8;LOC119873859;SHC2;SHC3;SP1;CREB5;CREB3;CREB1;ADCY4(up);KRAS;SOS2;FKBP4;ADCY6;ADCY7;ADCY1;ADCY2;FKBP5;ADCY3;ADCY8;ADCY9;GRB2;GNAO1;HRAS;HSP90B1;GABBR1;TGFA;LOC100687242;JUN;PIK3R2;PIK3R1;GRM1;LOC119869567;PLCB2;PLCB3;PLCB4;FOS;LOC119870217;LOC119870216;LOC119870215;GNAI1(up);NCOA1;ITPR2;ITPR3;LOC612100;ESR2;GNAQ;ESR1;CALML6;CALML5;CALML4;GABBR2(up);LOC102153034(down);RAF1;GNAI3;NCOA2;NCOA3;ITPR1;HBEGF;MAPK3;MAPK1;SRC;PLCB1(up);OPRM1;POMC;KCNJ3;KCNJ6;KCNJ5;KCNJ9;GNAS;PRKACA;PRKACB;HSP90AA1;LOC474850;EGFR;MAP2K2;MAP2K1;HSP70;SHC1;NRAS;PGR;NOS3;CTSD;PRKCD;CREB3L2;CREB3L3;CREB3L1;CREB3L4;MMP9;ATF4;ATF6B;BCL2;ATF2 |
| NOD-like receptor signaling pathway | IKBKB;IFNAR1;IKBKE;TYK2;SUGT1;MAP3K7;GABARAP;HSP90AB1;FADD;RELA;IRAK4;TRPV2;CTSB;IFNAR2;TRPM7;TRIP6;MAVS;CCL2;CCL5;NFKBIB;CXCL8(up);NFKBIA;PSTPIP1(up);VDAC1;BCL2L1(down);TANK;NLRX1;JUN;CARD9;CARD8;JAK1;LOC119876828;MYD88;CARD6;TRPM2;PLCB2;PLCB3;PLCB4;CHUK;ATG12;ITPR2;ITPR3;RHOA;LOC612100;IRF3;TLR4(down);LOC100686162;IRF7;TBK1;NOD2(up);LOC100683567;ERBIN;IRF9;TNFAIP3;TNF;STING1;NAMPT;LOC119874472(up);MAPK14;XIAP;MAPK10;MAPK11;MAPK12;MAPK13;VDAC3;VDAC2;IL1B;LOC100687242;LOC611406;TICAM1;ITPR1;STAT2;NLRP3;IFNB1;MAPK3;MAPK1;NFKB1;MAPK8;MAPK9;PLCB1(up);IL18;LOC119876918(up);IFNA7;IFNA5;STAT1;IL6;MFN2;HSP90AA1;BIRC3;BIRC2;RIPK1;RIPK3;RIPK2;P2RX7;GABARAPL1;GABARAPL2;ATG16L1;CAMP;NOD1;ATG5;PYCARD;YWHAE;TRAF2;TRAF3;NLRP1(up);TRAF5;TRAF6;PKN1;PKN3;PKN2;PRKCD;CYBA;TAB3;TAB2;TAB1;CASP8;DNM1L;LOC608573;LOC102153882;BCL2 |
| Toll-like receptor signaling pathway | LOC100856339;IFNAR2;AKT1;AKT2;AKT3;IKBKE;MAP3K8;PIK3CA;PIK3CB;PIK3CD;MAP3K7;FADD;RELA;IRAK1;IRAK4;LOC480600;LY96;IKBKB;TLR2;TLR3;IFNAR1;TLR6;TLR7;TLR5;TLR8;CCL3;CCL4;CCL5;CXCL8(up);NFKBIA;IL12A(up);TLR9\_1;TIRAP;JUN;TLR1;PIK3R2;PIK3R1;LOC119876828;MYD88;FOS;LOC119870217;LOC119870216;LOC119870215;CHUK;IRF3;TLR4(down);LOC100686162;IRF7;STAT1;TOLLIP;LOC100683567;CD14;MAPK14;MAPK10;MAPK11;MAPK12;MAPK13;IL1B;IFNB1;LOC611406;TICAM1;TICAM2;TBK1;CD80;CD86;MAPK3;MAPK1;NFKB1;MAPK8;MAPK9;CTSK(up);LOC485869(down);CXCL10(down);MAP2K3;IRF5;IFNA7;IFNA5;IL6;SPP1;IL12B;RIPK1;MAP2K2;MAP2K1;MAP2K7;MAP2K6;MAP2K4;TRAF3;RAC3;TRAF6;RAC1;CD40;TNF;TAB2;TAB1;CASP8 |
| Adherens junction | AFDN;IGF1R;ACTG1;SRC;SMAD4;EGFR;TGFBR1;CTNNB1;INSR;WASL;CTNND1;FARP2;LMO7;PTPN1;YES1;IQGAP1;EP300;WASF1;WASF2;PTPRF;FYN;MAP3K7;CSNK2A2;NECTIN2;MAPK3;PTPN6;MAPK1;CSNK2A1;NECTIN4;CDH1;NECTIN1;NLK;FGFR1;ERBB2;TGFBR2;TCF7;LOC119866928;RAC3;RAC1;RHOA;VCL;SSX2IP;PARD3;LOC489647;SNAI2;LEF1;SNAI1;WAS;CTNNA1;CTNNA2;CTNNA3;ACTN1;TJP1;RAC2(up);ACTB;ACTN4;SORBS1(up);WASF3;TCF7L1;TCF7L2;PTPRJ;MET;NECTIN3(down);PTPRB;BAIAP2;PTPRM;CSNK2B;CREBBP;CDC42 |
| RIG-I-like receptor signaling pathway | IFIH1;NFKBIB;CXCL8(up);OTUD5;NFKBIA;IL12B;IL12A(up);CASP10;MAPK10;IFNA5;MAPK12;IKBKE;IFNK;PIN1;NLRX1;LOC611406;MAPK14;TKFC;DDX58;TANK;TBK1;MAP3K1;MAP3K7;TRADD;IFNB1;AZI2;ISG15;CXCL10(down);FADD;NFKB1;RELA;MAPK9;RIPK1;MAPK8;TRAF2;TRAF3;DDX3X;TRAF6;SIKE1;TRIM25;ATG5;CHUK;ATG12;TNF;LOC119876828;TBKBP1;IRF3;IKBKB;LOC100686162;IRF7;DHX58;LOC100683567;IFNA7;CASP8;MAPK13;CYLD;STING1;RNF125;MAVS;MAPK11 |
| Terpenoid backbone biosynthesis | HMGCS1;HMGCS2;RCE1;FNTA;FNTB;FDPS;PCYOX1;IDI1;PMVK;DHDDS;PDSS1;PDSS2;ACAT1;ACAT2;MVD;HMGCR;GGPS1;ZMPSTE24;MVK;ICMT;NUS1 |
| Indole alkaloid biosynthesis | DDC |
| Limonene and pinene degradation | ALDH1B1;ALDH2 |
| Gap junction | ADCY1\_1;EGF;MAP3K2;CSNK1D;HTR2A;GJD2;HTR2B;ADCY4(up);KRAS;ADCY5;ADCY6;ADCY7;ADCY1;ADCY2;ADCY3;TUBB4B;TUBB4A;ADCY8;ADCY9;GRB2;GUCY1B1;LOC478702;HRAS;CDK1;TUBA1C;RAF1;GUCY1A1;GUCY1A2;PRKG1;HTR2C;PRKG2;GRM5;LOC100855928;GRM1;LOC119869567;PLCB2;PLCB3;PLCB4;TUBB6;TUBB3;GNAI1(up);GJA1(up);ITPR2;ITPR3;TUBB1;TUBB2A;TUBAL3;GNAQ;GNAS;TUBB;LOC477570;PDGFRB;PDGFRA;TUBA8;GNAI2;GNAI3;ITPR1;MAPK3;MAPK1;MAPK7;SRC;LOC485435;PLCB1(up);LOC491231;LPAR1(up);LOC610636;TJP1;SOS2;ADRB1;PRKACA;PRKACB;EGFR;MAP2K2;MAP2K1;MAP2K5;LOC106557476;NRAS;LOC608051;PDGFA;PDGFB;PDGFC;PDGFD;PRKCA;PRKCB;PRKCG;TUBA4A;DRD2;DRD1;GNA11;LOC119866377 |
| Circadian rhythm - plant | COP1;CSNK2A2;CSNK2A1;CSNK2B |
| Zeatin biosynthesis | TRIT1 |
| Sesquiterpenoid and triterpenoid biosynthesis | FDFT1;SQLE |
| Porphyrin and chlorophyll metabolism | ALAD;EARS2;LOC102154822;LOC480777;UGT2A3;EPRS1;UROS;PPOX;UGT1A6(down);LOC102154742;BLVRB;UROD;FECH;HMBS;HCCS;ALAS1;ALAS2;GUSB;CPOX;FXN;HEPH;BLVRA;CP;LOC611632;HMOX1;COX10;MMAB;COX15 |
| Glycosaminoglycan degradation | GNS;ARSB;HYAL3;HYAL2;HYAL1;HEXB;GALNS;IDS;GLB1;HYAL4;HPSE(up);HEXA;SPAM1;NAGLU;HGSNAT;LOC609321;SGSH;HPSE2;IDUA;GUSB |
| RNA transport | AAAS;NCBP1;NCBP2;SUMO1;SUMO3;SUMO2;LOC492024;LOC100687327;EIF4EBP2;EIF3H;EIF3I;EIF3J;DDX39B;RAN;EIF3A;EIF3B;EIF3D;EIF3E;NDC1;STRAP;SAP18;NUP214;NUP210;MAGOH;RPP40;LOC100685407;NUP98;ACIN1;NUP93;RANGAP1;PRMT5;FXR1;FXR2;TPR;THOC2;THOC1;LOC490297;THOC6;THOC5;LOC102151333;LOC611908;EIF4G3;EIF4G2;EIF4G1;POP1;POP7;POP5;LOC111096123;NUP205;NUP88;POM121C;NCBP2L;SEH1L;PABPC1;PABPC4;NUP85;PAIP1;EIF5B;XPOT;XPO1;XPO5;THOC7;PABPC1L2A;TACC3;LOC100684705;CLNS1A;NXF1;NXF3;SEC13;RPP25;ALYREF;SENP2;RPP25L;LOC100684788;PYM1;EIF4E;RPP30;EIF4B;UBE2I;MAGOHB;NUP153;EIF4EBP1;NUP155;LOC119871262;EIF4A1;EIF4A3;EIF4A2;LOC612587;LOC475517;NUP133;LOC479795;EIF2B5;EIF2B2;EIF2B3;EIF2B1;SMN;UPF1;EIF3F;UPF2;EIF3G;NUP62;RBM8A;PABPC1L;PHAX;LOC102153597;EIF1;LOC119872870;RANBP2;NUP107;LOC486757;SNUPN;PABPC5;TGS1;NUP50;NUP210L;NUP54;GEMIN2;NUP58;GEMIN6;GEMIN7;GEMIN4;GEMIN5;NXT2;NXT1;EIF4E1B;TRNT1;LOC106559713;CYFIP1;CYFIP2;EEF1A1;EEF1A2;LOC481575;PABPC4L;LOC100684102;LOC100685896;FMR1;LOC611386;EIF2S1;EIF2S2;EIF1B;ELAC1;ELAC2;NUP42;NUP43;NUP188;RNPS1;EIF4EBP3;EIF2B4;NUP160;KPNB1;EIF4E2;CASC3;LOC100685582;NMD3;RPP21\_1;DDX20;SRRM1;RPP38;NUP62CL;NUP35;NUP37;UPF3B;UPF3A;LOC102153953;RPP14;PNN;EIF5;RAE1 |
| Glycosaminoglycan biosynthesis - keratan sulfate | ST3GAL3;ST3GAL2;ST3GAL1;B3GNT7;CHST1(down);B3GNT2;LOC476397;CHST2(up);CHST4;LOC489707;B4GALT1;B4GALT3;B4GALT2;B4GALT4;FUT8 |
| Glycosaminoglycan biosynthesis - chondroitin sulfate / dermatan sulfate | CHST11;CHST13;B3GALT6;CHST12;CHST15;CHST14;CSGALNACT1(up);CHST3(up);XYLT2;CHSY3;CHSY1;UST;CHST7;DSE;B3GAT3;CHPF;CSGALNACT2;B4GALT7;XYLT1(up) |
| Glycosaminoglycan biosynthesis - heparan sulfate / heparin | HS3ST2;HS3ST1;HS2ST1;B3GAT3;LOC489516;NDST1;NDST2;NDST3;NDST4;B4GALT7;XYLT1(up);B3GALT6;EXT2;EXT1;EXTL1;EXTL2;EXTL3;XYLT2;GLCE;HS3ST5(down);HS6ST1;HS6ST2;HS6ST3;HS3ST3A1 |
| Hippo signaling pathway - fly | ZDHHC14;PPP2R1B;YWHAQ;FJX1;PPP2R1A;LOC100855903;RASSF2;ZDHHC18;RASSF4;SAV1;LATS2;LATS1;DCHS2;MAPK10;MPP5;TEAD2;TEAD3;MOB1A;MOB1B;TEAD1;ACTG1;DLG1;TP63;RERE;FAT4(up);CCNE2;PPP2R2A;WWC1;CCNE1;PARD6B;PARD6A;MAPK8;CSNK1E;NF2;PPP2R2D;YWHAB;LIMD1;TEAD4;AJUBA;MPDZ;LOC478670;PRKCI;YWHAZ;LOC608573;TSHZ2;TSHZ3;TSHZ1;CRB1;PPP2R2C;SMAD1;STK3;CRB2;MYC;YAP1;LOC479459;PARD3;LLGL2;ACTB;LLGL1;ZDHHC9;MAPK9;WNT1;PPP2CB;SCRIB;PPP2CA;LOC609669;DCHS1;PARD6G(up);WTIP;YWHAE;LIX1L(up);PPP2R2B(up) |
| Hippo signaling pathway | ACTG1;CCN2;LOC100855618;SAV1;LOC607768;DLG1;GLI2;DLG4;DVL3;WNT10A;WWC1;PARD6B;PARD6A;CSNK1D;CSNK1E;LOC100855903;CDH1;MYC;FRMD1(down);BMP6;TP73(down);CRB1;PPP2R2C;CRB2;STK3;SNAI2;LLGL2;BMP7;LOC479459;BMP5;BMP4;CCND1;PPP2CA;CCND3;CCND2;TCF7L1;TCF7L2;GSK3B;WNT3;LOC609669;BMPR2;PARD6G(up);APC;ACTB;WNT7A;WNT7B;WNT2B;FBXW11;RASSF1;RASSF6;FZD10;ID1;SERPINE1;AMH;DLG2(up);NF2;TCF7;PPP2CB;LOC489647;SOX2;FRMD6;AFP;FGF1;BMPR1A;LOC119863906;WNT5B;WNT5A;WNT10B;WNT3A;PPP2R1A;SMAD4;SMAD7;SMAD1;SMAD2;SMAD3;AREG(up);MPP5;WNT8B;FZD1;FZD2;FZD3;FZD4;FZD6;FZD7;FZD8;FZD9;LOC102151783;LIMD1;NKD1;TGFBR2;TGFBR1;NKD2;LEF1;YAP1;ITGB2(down);LLGL1;WWTR1;WNT2;WNT1;ID2;WNT6;WNT4;WTIP;AMOT;TGFB1;TGFB2;TGFB3;BIRC5;BIRC3;BIRC2;PPP2R1B;CTNNB1;LATS2;LATS1;MOB1A;TEAD3;TEAD2;MOB1B;PATJ;TEAD1;TEAD4;YWHAZ;AXIN2;AXIN1;YWHAQ;GDF7;GDF5;DVL2;WNT8A;YWHAH;DVL1;PPP2R2D;YWHAB;AJUBA;YWHAG;YWHAE;PRKCI;BMPR1B;PPP2R2A;PRKCZ;TP53BP2;APC2;CTNNA1;CTNNA2;CTNNA3;PARD3;PPP1CB;PPP1CC;PPP1CA;BTRC;BBC3;SCRIB;WNT9B;WNT9A;WNT16;LOC608573;WNT11;PPP2R2B(up) |
| Taurine and hypotaurine metabolism | GGT5;BAAT;CDO1;CSAD;GGT1;GGT6;GGT7;ADO;GADL1;LOC483960;GAD1 |
| SNARE interactions in vesicular transport | STX1A;STX4;STX7;LOC100688933;BET1;STX1B(down);LOC480074;BET1L;STX17;STX8;STX16;YKT6;GOSR2;VAMP2;BNIP1;LOC102151885;VAMP1;SEC22B;VAMP3;LOC100856502;VAMP5;VAMP4;VAMP7;SNAP23;VAMP8;STX3;STX2;STX5;SNAP29;LOC100683684;STX6;STX11;STX10;VTI1A;USE1;VTI1B;STX19;STX18;GOSR1 |
| Nucleotide excision repair | ERCC8;LOC100685309;RAD23A;RFC5;RFC4;ERCC2;ERCC3;RFC1;ERCC5;RFC3;RFC2;POLE;RAD23B;LOC478089;CETN2;POLD1;POLD2;POLE4;LIG1;ERCC1;DDB2;DDB1;XPA;PCNA;GTF2H3;ERCC4;GTF2H1;POLD3;GTF2H5;LOC483061;ERCC6;LOC482008;MNAT1;LOC611847;RPA1;RPA3;RPA2;CUL4A;CUL4B;POLE3;POLE2;CDK7;XPC;RBX1;CCNH |
| DNA replication | POLA2;RFC5;RFC4;RFC1;RFC3;RFC2;POLE;FEN1;DNA2;POLD1;POLD2;POLD3;PRIM1;LIG1;PRIM2;PCNA;RNASEH1;MCM6;MCM5;MCM4;MCM3;MCM2;SSBP1;LOC611847;RNASEH2A;RNASEH2C;RNASEH2B;RPA1;RPA3;RPA2;POLE4;POLE3;POLE2 |
| Mitophagy - animal | USP8;JUN;HRAS;RRAS2;PRKN;HIF1A;RRAS;FOXO3;PGAM5;MAPK10;RHOT1;MITF;BCL2L1(down);GABARAPL1;TBK1;GABARAPL2;NRAS;GABARAP;ATG5;RELA;KRAS;SRC;RAB7A;RPS27A;BECN1;BECN2;SP1;MAPK8;MRAS;RAB7B(up);CSNK2A2;CSNK2A1;BNIP3;SQSTM1;MAPK9;TP53;E2F1;EIF2AK3;MFN2;UBA52;FIS1;UBC;ATF4;CSNK2B |
| Autophagy - other | ATG101;RPTOR;MLST8;GABARAPL1;GABARAPL2;ATG16L1;GABARAP;ATG3;PIK3R4;ATG7;ATG5;ULK2;BECN1;BECN2;ATG4C;ATG4B;ATG4A;ATG13;MTOR;ATG4D;PIK3C3;ATG10;PPP2CA;PPP2CB;ATG12 |
| Biosynthesis of cofactors | LOC480777;MAT1A;NQO1;MOCS2;AK6;PMM2;LIPT2;PMM1;UGP2;ASPDH;SPR;DHRS3;LOC100688420;LOC100687518;NADK2;GUSB;BCAT2;BCAT1;PPCS;LOC102152410;SHMT2;SHMT1;NME4;NME6;NME7;NME1;NME2;NME3;LOC102151128(down);DHODH;NFS1;PNPO;COQ2;MMAB;LOC102156116;GGCX;LOC102154822;LOC102154109;EPRS1;UROS;PPOX;AKR1A1;UGT1A6(down);UROD;RGN;PANK1;PANK3;PANK2;DLD;PTS;PKM;UMPS;PKLR;MTHFD2L;RFK;GPHN;MTHFD2;MTHFD1;GCH1;MPI;NAPRT;COX10;LOC483943;COX15;NADK;ALAD;EARS2;KMO;AFMID;UGT2A3;PDXK;HAAO;LOC102154742;MTHFD1L;ADSL;FECH;HMBS;QPRT;COASY;CTPS2;AK5(up);SDR16C5;VKORC1;OXSM;LOC611724;ALAS1;ALAS2;BCO1;NMNAT2;NMNAT3;NMNAT1;GGH;PPCDC;UGDH;GMPPA;GMPPB;CPOX;TPK1;AK9;AK8;AK3;AK2;AK1;LOC486100(down);AK7;LOC102156938;ALDH2;AK4;ADSS2;LOC102155863;ADSS1;IDO1;DHFR;FLAD1;NADSYN1;PDXP;LOC608853;LOC102157398;LOC480491;CAD;GCLM;CMPK1;CMPK2;GCLC;VKORC1L1;PHOSPHO2;HSD17B6;RDH11;TDO2;MAT2A;LIAS;MAT2B;KYNU;FPGS;ALDH1B1;ALPI;HPD;LOC111091468;IDO2(down);PSAT1;CTPS1;LOC609048;RDH12;COQ5;COQ7;COQ6;COQ3;ALPL |
| Ribosome | LOC119870304;MRPS11;LOC106558744;LOC608876;LOC489638;LOC102152540;LOC111096925;LOC100687285;RPL22;LOC607189;LOC100685924;RPL22L1;LOC102153812;LOC106558130;LOC119869838;LOC119871462;LOC106558887;LOC106558139;RPSA;RPLP2;LOC106559944;LOC119863879;LOC119872934;RPLP1;LOC111090701;LOC106558701;MRPL36;LOC119872856;MRPL34;MRPL35;MRPL32;LOC607833;LOC119871403;LOC100682691;LOC111094581;MRPS5;MRPL30;LOC100686899;RPL35A;RPL14;RPL15;RPL17;RPL11;RPL12;RPL13;LOC102154256;LOC102155634;RPL18;RPL19;LOC100683749;LOC102155220;LOC106560108;LOC100686416;LOC119864483;MRPS18A;LOC100686103;MRPL12;LOC608162;LOC106559359;LOC488356;UBA52;LOC607844;LOC111098653;LOC106559922;LOC106559055;LOC119870571;LOC100686712;RPS2;LOC100856351;MRPL27;RPL37A;LOC483242;MRPL20;RPL8;RPL9;RPL6;RPL7;RPL27A;RPS27A;LOC100685244;RPL3;LOC119868360;LOC106559752;LOC477105;MRPS10;LOC478576;MRPL1;LOC100683928;LOC106559265;RPS16;LOC479033;LOC106559613;RPL7A;MRPL16;LOC100687735;LOC106559452;LOC102151361;LOC480918;RPL5;MRPL28;LOC119877709;LOC475097;LOC100682801;MRPL19;RPL10A;LOC119876411;LOC119864713;LOC106559926;LOC106559924;MRPL10;MRPL11;LOC119873878;MRPL13;MRPL14;MRPL15;LOC119870219;MRPL17;MRPL18;LOC480235;LOC100856286;MRPS9;MRPL4;MRPL2;MRPL3;LOC119866623;MRPL33;LOC106557643;LOC102155750;LOC612456;LOC106557880;LOC100683111;LOC100682679;LOC100687042;LOC106559375;LOC106559504;LOC111096979;LOC100856611;MRPS21;LOC100682831;RSL24D1;LOC479087;LOC100856758;LOC106558045;LOC608703;LOC100683963;LOC100684245;LOC100855547;LOC100682542;MRPL24;LOC106558335;LOC607191;RPLP0;RPL18A;LOC481399;LOC100688232;LOC492065;RPS7;RPS6;RPS5;RPS3;LOC119867317;LOC106559080;RPL36AL;LOC106559099;LOC111096443;RPS9;RPS8;LOC119871822;LOC106558762;RPS27L;LOC100687051;MRPL22;LOC100688077;LOC106560013;RPL13A;LOC100855538;LOC119872019;LOC608684;LOC100687833;LOC106560154;LOC100686620;RPS26;RPS27;RPS24;RPS25;RPS23;RPS20;RPS21;RPS15A;LOC486972;LOC119872639;RPS28;RPS29;LOC100687749;LOC106559371;RPS4X;LOC106557704;LOC119866847;LOC111095093;LOC100684485;LOC608750;LOC100686354;RPL4;LOC100687065;LOC119865194;LOC106559461;LOC100856299;LOC100685611;LOC119870530;RPS3A;LOC100684084;RPL37;RPL10L;LOC106559872;RPS13;RPS12;RPS11;RPS10;RPS17;LOC486061;RPS15;RPS14;LOC100683563;RPS19;RPS18;MRPL9;MRPS15;LOC491854;MRPS16;RPL3L;MRPS14;MRPS12;LOC106559895;LOC100856037;LOC106557771;LOC106558632;LOC486183;LOC111096198;LOC119864693;LOC611750;RPL29;RPL28;LOC100686364;LOC488981;RPL24;RPL27;RPL26;RPL21;MRPS2;RPL23;FAU;LOC119870132;MRPS7;LOC100686736;LOC106558419;LOC102155971;LOC100685685;LOC102153530;LOC106559787;LOC490332;LOC102154836;LOC119873934;LOC106558923;LOC100688637;LOC100687764;MRPS18C;LOC111090092;RPL32\_1;LOC102156381;MRPL23;LOC119864697;LOC119871977;LOC608772;RPL38;RPL36;LOC100687236;RPL34;RPL35;MRPL21;LOC106557609;RPL30;RPL31;LOC106558083;LOC106560120;LOC100687566;LOC119864957;LOC100683287;LOC119871302;LOC106557682 |
| Long-term potentiation | PPP3R2;PPP3R1;GRIA2;HRAS;ADCY1\_1;LOC102153034(down);RAP1B;RAP1A;MAP2K2;MAP2K1;GNAQ;ARAF;RAPGEF3;RAF1;PPP1R1A;ITPR1;CALM2;CALM3;CALM1;GRM1;CACNA1C;NRAS;CAMK4;CAMK2D;MAPK1;GRM5;GRIN1;KRAS;MAPK3;LOC119869567;PLCB2;PRKCB;PLCB4;PRKCA;CAMK2G;CAMK2A;PRKCG;PLCB3;PLCB1(up);GRIA1;CAMK2B;ATF4;ITPR2;ITPR3;EP300;ADCY1;RPS6KA1;RPS6KA2;PPP1CB;PPP1CC;RPS6KA6;PPP1CA;ADCY8;CALML6;CALML5;CALML4;GRIN2B;GRIN2C;GRIN2A;PRKACA;BRAF;GRIN2D;PRKACB;PPP3CA;PPP3CB;PPP3CC;CREBBP |
| Mineral absorption | VDR;MT2A;ATP1A3;SLC31A1;SLC26A9;FXYD2;S100G;CYBRD1;SLC30A1;SLC8A3(up);LOC119864140;ATP7B;SLC9A3;ATP7A;FTL;SLC6A19;SLC8A1;LOC100684085;TRPM6;SLC8A2;LOC100688200;LOC477071;LOC477072;STEAP1(up);CLCN2;ATP1B4;ATP1B2;ATP1B3;ATP1B1;SLC26A3;LOC119873292;MT1E;ATP2B1;HEPH;ATP2B3;ATP2B2(up);SLC39A4;ATP2B4;LOC119870853;TRPM7;SLC40A1;ATOX1;SLC5A1;LOC102154317;LOC484507;HMOX1;LOC102153158;LOC102151236;ATP1A4;LOC487855;ATP1A1;LOC100686073;STEAP2(up);LOC403631 |
| Riboflavin metabolism | ACP2;FLAD1;ENPP3(down);ENPP1;ACP5(down);RFK;BLVRB |
| Apoptosis - multiple species | PMAIP1;BIRC7;BIRC6;BIRC5;BIRC3;BIRC2;CYCS;XIAP;BOK;MAPK10;DIABLO;BBC3;BAK1;HTRA2;BCL2L1(down);BCL2L11;BID;FADD;MAPK8;MAPK9;APAF1;BECN1;BECN2;LOC100689004;BAX;CASP7;CASP3;TNFRSF1A;CASP8;CASP9;SEPTIN4;NGFR(up);BCL2 |
| Prodigiosin biosynthesis | MCAT |
| C5-Branched dibasic acid metabolism | ACOD1 |
| Vitamin digestion and absorption | PLB1(up);FOLH1B(up);PNLIP;SLC5A6;APOA4;APOA1;AWAT2;SLC52A3;APOB;CBLIF;LRAT;LMBRD1;CUBN;RBP2;MMACHC;LOC486151;LOC486150;SCARB1;TCN2;BTD;SLC19A2;ABCC1;SLC19A1 |
| Arginine and proline metabolism | AOC1;SAT2;GAMT;CKM;LAP3;AGMAT;P4HA2;P4HA3;OAT;PYCR3;PYCR2;PYCR1;ODC1;CARNS1;L3HYPDH;SMOX;PRODH2;DAO(down);GOT2;LOC102153601;ALDH18A1;GOT1;LOC477562;AMD1;NOS1;NOS2(up);NOS3;LOC100855552;SRM;ALDH9A1;ALDH7A1;ALDH1B1;ALDH4A1;CNDP1;CNDP2;LOC100688166;HOGA1;LOC478277;CKMT2;ARG1;ARG2;ALDH2;AZIN2;MAOB;MAOA;P4HA1;LOC100685416 |
| Metabolism of xenobiotics by cytochrome P450 | EPHX1;LOC100856533;LOC479911;LOC479912;GSTA4;LOC480777;ALDH3A1;UGT2A3;LOC119867230;MGST2;MGST3;LOC611366;UGT1A6(down);LOC102154742;CYP2S1;LOC119867218;CYP1B1;HSD11B1;CYP2F1;LOC476006;LOC102154822;LOC486404;CBR3;LOC100688446;ALDH1A3;LOC477556;LOC610164;ALDH3B1;CYP2A13;CYP1A2(up);MGST1(down);LOC474938;AKR7A2;HPGDS;GSTO2;CYP1A1(up);GSTO1;GSTT2B;ADH5;ADH4;LOC607806;CYP2E1;GSTP1;LOC481841;LOC100856518;LOC610304;DHDH;LOC477558;GSTK1 |
| Insect hormone biosynthesis | ALDH1B1;ALDH2 |
| Drug metabolism - cytochrome P450 | AOX2;LOC100856533;LOC479911;LOC479912;GSTA4;LOC480777;ALDH3A1;UGT2A3;LOC119867230;MGST2;MGST3;LOC611366;UGT1A6(down);LOC102154742;LOC490346;AOX4;ALDH1A3;LOC476006;LOC102154822;LOC486404;MAOA;LOC119867218;LOC478994;LOC481841;FMO2;FMO3;ALDH3B1;FMO1;FMO4;FMO5(down);MGST1(down);LOC474938;HPGDS;GSTO2;GSTO1;GSTT2B;ADH5;ADH4;CYP2E1;MAOB;CYP1A2(up);LOC477556;LOC100856518;LOC610304;GSTP1;LOC477558;GSTK1 |
| Drug metabolism - other enzymes | LOC102156116;LOC479911;RRM1;LOC102154822;GSTA4;LOC480777;UGT2A3;LOC486404;NME1;MGST2;MGST3;LOC611366;UGT1A6(down);LOC102154742;LOC102154109;GSTP1;LOC119867218;DPYD;LOC476006;CMPK1;HPRT1;LOC479912;IMPDH1;IMPDH2;XDH;DPYS;LOC100688420;RRM2B;GSTO1;GUSB;GSTO2;LOC481841;TPMT;UMPS;UCK1;UCK2;NME6;CES1;CES2;TK2;TK1;LOC474938;DUT;GMPS;NME4;LOC111091468;UPP1;NME7;GSTT2B;CDA;NME2;UCKL1;LOC102151128(down);MPO;LOC477556;NME3;CYP2E1;ITPA;LOC102155863;LOC100856518;LOC610304;UPB1(up);MGST1(down);LOC477558;LOC483943;RRM2 |
| Necroptosis | LOC100682891;IFNAR2;IFNAR1;TYK2;HSP90AB1;PYGB;CAPN2;CAPN1;FADD;PYGL;PYGM;IFNG;CAMK2G;SLC25A6;SLC25A5;SLC25A4;FASLG;CHMP2B;LOC102154317;CAMK2B;LOC612295;LOC102153158;TLR3;CHMP2A;CYLD;SMPD1;LOC403631;TNFSF10;VDAC3;GLUL;SLC25A31;IFNGR1;LOC609798;IL1A(up);PLA2G4E(up);VDAC1;ZBP1;CHMP6;JAK2;JAK3;JAK1;LOC100684085;LOC119876828;TRPM7;LOC100684252;STAT6;FAF1;IFNA5;LOC612100;FTL;LOC119870853;TLR4(down);LOC100686162;NLRP3;TNFRSF1A;LOC100683567;LOC484507;IRF9;TNFAIP3;AIFM1;LOC609805;XIAP;PGAM5;MAPK10;CHMP3;VDAC2;IL1B;LOC100687242;LOC611406;CHMP5;TICAM1;TICAM2;STAT4;STAT3;STAT2;STAT1;TRADD;IFNB1;CFLAR;MAPK8;MAPK9;CHMP4B;VPS4A;VPS4B;CHMP1B;CHMP1A;EIF2AK2;IFNA7;ALOX15;GLUD1;STAT5B;STAT5A;LOC487855;CHMP7;HSP90AA1;IFNGR2;CHMP4A;HMGB1;CHMP4C;BIRC3;BIRC2;BAX;RIPK1;RIPK3;FAS;BID;CAMK2D;PYCARD;LOC100688200;TRAF2;TRAF5;CAMK2A;TNF;IL33;PLA2G4B;PLA2G4A;PLA2G4F;PLA2G4D;SQSTM1;CASP8;DNM1L;BCL2 |
| Ubiquinone and other terpenoid-quinone biosynthesis | TAT;NQO1;COQ6;GGCX;VKORC1;VKORC1L1;COQ5;COQ7;COQ2;COQ3;HPD |
| Spinocerebellar ataxia | LOC100856339;AKT1;AKT2;AKT3;LOC100687152;RYR1;CACNA1A;PIK3CB;PIK3CD;MAP3K5;RELN;GRIN1;RBPJL;SP1;SLC25A6;SLC25A5;SLC25A4;PIK3C3;LOC610188;ADRM1;ATP2A1;ATP2A2;GRIA2;GRIA3;GRIA1;SLC25A31;LOC609798;PLCB4;GTF2B;PIK3R4;PIK3R2;PIK3R1;GRM1;PLCB2;PLCB3;BECN1;BECN2;LOC119870217;LOC119870216;LOC119870215;ATG13;ATG14;LOC100689004;PPIF;ITPR2;ITPR3;ITPR1;LOC100686432;TBPL2;TBPL1;GNAQ;TBP;PSMD11;PSMD13;PSMD12;LOC100687556;PIK3CA;RBPJ;ATP2A3(down);TRPC3;MAPK10;VDAC3;VDAC2;VDAC1;RORA(up);PSMA2;PSMA3;PSMA1;PSMA6;PSMA7;PSMA4;PSMA5;MAPK8;MAPK9;ERN1;LOC100687259;PLCB1(up);MTOR;CYCS;PSMA8;LOC100687060;LOC100687064;PSMD14;PSMB7;PSMB6;PSMB5;PSMB4;PSMB3;PSMB2;PSMB1;CIC;PSMD8;ATG101;PSMD4;PSMD7;PSMD6;PSMD1;PSMD3;PSMD2;NFYA;RB1CC1;XBP1;NOP56;GRIN3A;GRIN3B;LOC100686569;PSMC1;PSMC2;PSMC3;PSMC4;PSMC5;PSMC6;ULK2;TRAF2;PRKCA;PRKCB;PRKCG;SEM1;LOC100683681;ATXN3;GRIN2B;GRIN2C;GRIN2A;GRIN2D;SLC1A6 |
| Caffeine metabolism | CYP1A2(up);UOX;XDH |
| Microbial metabolism in diverse environments | PCK2;GRHPR;PCK1;LOC611563;PCCB;PCCA;TPI1;FBP2;LOC100686034;ENOSF1;OGDH;ALLC;HKDC1;TKTL1;FAH;MPST;KHK;PGAM2;GOT2;RPE;GOT1;LOC477441;GPI;ADPGK;HK2;HK3;IDH1;HK1;LOC111094513;ACACB(down);PHGDH;LOC490690;GPT;LOC608800;EHHADH;SHMT2;SHMT1;HGD;ACYP1;ACYP2;LOC106558345;LOC102154094;LDHA;ECHS1;PNPO;ALDH5A1;LOC102151507;PGK1;CPS1;GPT2;ACADS;GSTZ1;UOX;GAPDH;EPRS1;UROS;LOC482320;GLUL;AKR1A1;GLUD1;TST;IDH3B;RGN;PDHX;XDH;PC;TKT;PDHB;PGK2;PKM;DLD;GCK;PGM1;PGM2;LOC100684709;DLAT;LOC483960;ACO1;ACO2;ETHE1;HADH;LOC102151775;MTHFD1;GLYCTK;TKFC;LOC481849;SUCLG1;EPHX2;ALAD;EARS2;ALDOA;LOC612295;ME1;ME3;PGAM1;FH;AGXT;GAD1;HMBS;ACAT1;FAHD1;ACAT2;LOC610338;ACLY;PDHA2;ALDH3A1;ACACA;LOC102152592;MTHFR;ACSS2;ACSS1;MMUT;ENO2;NUDT11;ENO4;ALDH9A1;ALDH7A1;CS;GALM;LOC102152275;CMBL;ADH5;PSPH;DLST;SUCLA2;BPNT2;PGLS;ALDH2;LOC119876526;AOX2;AOX4;MDH1;BPGM;TALDO1;LDHAL6B;MDH2;ABAT;SUOX;LOC111096459;GCDH;ALDH1A3;PAPSS1;PGD;PAPSS2;PRPS2;IDH3G;PRPS1;HADHA;PFKP;LOC479379;PFKM;PFKL;RPIA;LOC111089999;IDNK;IDH2;MCEE;OGDHL;LOC119870407;LDHB;LDHC;IDH3A;ALDH3B1;LOC100683724;LOC609911;LOC102153001;SUCLG2;ESD;LOC119866771;ALDH1B1;ALDOC;ALDOB;SDHC;BPNT1;ENO1;PSAT1;ENO3;FBP1(up);SDHA;HAO1;HAO2;SDHB;SDHD |
| Purine metabolism | LOC611563;LOC485179;ADCY1\_1;URAD;ADCY2;ALLC;DCK;PDE10A;PDE7A;LOC100688420;PDE8B;PFAS;ENTPD6;ENTPD5;ENTPD4;ENTPD3;ENTPD1;ENPP4;ADCY6;ENTPD8;ADCY4(up);APRT;ADCY7;GUK1;LOC102152410;PDE8A;GMPR;GMPS;NME4;ADCY5;NME6;NME7;NME1;NME2;ADCY3;LOC102151128(down);NME3;ADCY8;ADCY9;GUCY1B1;NT5C3A;ADCY1;LOC102156116;FHIT(down);UOX;AK6;ADSL;NT5M;NT5C;NT5E;XDH;GUCY1A1;PAICS;GUCY1A2;RRM2B;NUDT2;PDE5A;PKM;LOC119869567;NUDT9;GUCY2C;GUCY2D;GUCY2F;ADA2;DGUOK;NUDT5;PGM2;PGM1;GART;PDE6B;PDE6C;PDE6A;PDE6G;PDE6D;PDE6H;LOC483943;AMPD2;AMPD1;ADCY10;LOC102154109;PDE11A;NT5C3B;ADPRM;PKLR;AK5(up);HPRT1;PDE3A;LOC611724;PDE3B;LOC100687518;PDE7B(up);LOC485435;NUDT16;ADA;ADK;AK9;AK8;AK3;AK2;AK1;AK7;LOC102156938;AK4;GDA;PPAT;ADSS2;LOC102155863;ADSS1;PDE4A;PDE4B;PDE4C;PDE4D;LOC608853;ATIC;LOC102157398;NPR1;NPR2;PDE9A;RRM2;RRM1;NTPCR;LOC480491;PAPSS1;PAPSS2;PRPS2;PRPS1;PDE1C;PDE1B;PDE1A;IMPDH1;IMPDH2;NT5C1B;NT5C1A;GMPR2;NT5C2;ENTPD2(down);ENPP3(down);ENPP1;LOC111091468;PNP;PDE2A;ITPA;CANT1;PRUNE1 |
| Insulin signaling pathway | PCK2;PCK1;CRKL;PRKAG1;PRKAG3;PRKAG2;IKBKB;AKT1;AKT2;AKT3;ARAF;RAPGEF1;FBP2;PPP1R3F;PPP1R3E;PPP1R3D;PPP1R3C;PPP1R3B;PPP1R3A;CALM1;HKDC1;PIK3CA;PIK3CB;PIK3CD;PYGB;PYGL;PYGM;SHC4;LIPE;SHC1;SHC2;SHC3;HK2;HK3;HK1;ACACB(down);KRAS;SOCS4;SOCS1;SOCS3;SOCS2;INPP5J;INPP5K;GSK3B;ELK1;LOC100856339;SLC2A4;HRAS;G6PC1;G6PC3;G6PC2;IRS2;CRK;RAF1;PPARGC1A;GRB2;PIK3R2;PIK3R1;PHKG2;PRKAB2;PRKAB1;LOC119870217;LOC119870216;LOC119870215;PRKACB;PHKA1;GYS1;GYS2;EIF4E;CALM2;PTPN1;CALM3;GCK;RHOQ;CALML6;CALML5;CALML4;LOC100686736;EIF4EBP1;FLOT1;FLOT2;MKNK2;MKNK1;LOC102153034(down);IRS1;PRKAR2A;RPS6;PRKAR2B;MAPK10;LOC102154108;PDE3B;TSC2;TSC1;PKLR;RPS6KB1;RPS6KB2;MAPK3;MAPK1;FASN;MAPK8;MAPK9;ACACA;MTOR;LOC491854;PRKAR1B;PPP1CC;SOS2;IRS4;FOXO1;BAD;SREBF1;PRKACA;EIF4E1B;RHEB;LOC106559872;PRKAA2;PRKAA1;MAP2K2;MAP2K1;CBL;LOC111096471(down);INS;RPTOR;INPPL1;NRAS;PHKB;FBP1(up);PRKCI;INPP5A;LOC100685582;PRKCZ;EXOC7;PDPK1;TRIP10;EIF4E2;PPP1CB;LOC608772;PPP1CA;SORBS1(up);INSR;PTPRF;BRAF;SH2B2;PHKG1(up);PRKAR1A |
| Pentose phosphate pathway | LOC611563;GLYCTK;H6PD;LOC482320;RBKS;FBP2;DERA;PGD;PRPS2;PRPS1;TKTL1;TALDO1;PFKM;PFKP;LOC479379;TKT;PFKL;RPIA;RGN;IDNK;RPE;GPI;PGM1;PGM2;NUDT11;ALDOA;LOC608800;ALDOC;ALDOB;PGLS;FBP1(up) |
| Longevity regulating pathway | LOC100856339;PRKAG1;PRKAG3;STK11;AKT1;AKT2;AKT3;ADIPOQ;PIK3CA;PIK3CB;PIK3CD;CAMK4;RELA;IGF1R;ADCY1\_1;SOD2;CREB5;CREB3;CREB1;ADCY4(up);KRAS;ADCY5;ADCY6;ADCY7;ADCY1;ADCY2;ADCY3;ADCY8;ADCY9;HRAS;IRS1;PPARGC1A;AKT1S1;PIK3R2;PIK3R1;LOC119869567;SIRT1;IGF1;PRKAB2;PRKAB1;LOC119870217;LOC119870216;LOC119870215;PRKACB;PPARG;ATG13;EIF4E;TP53;KL(up);INS;CAMKK2;EIF4EBP1;SESN1;SESN2;SESN3;TSC2;TSC1;EHMT1;EHMT2;ADIPOR2;ADIPOR1;RPS6KB1;RPS6KB2;NFKB1;APPL1;MTOR;BAX;IRS4;INSR;IRS2;PRKACA;EIF4E1B;RHEB;ATG101;PRKAA2;PRKAA1;CAT;FOXO3;FOXO1;LOC111096471(down);RB1CC1;RPTOR;NRAS;ATG5;PRKAG2;LOC100685582;EIF4E2;CREB3L2;CREB3L3;CREB3L1;CREB3L4;ATF4;ATF6B;ATF2 |
| Aminoacyl-tRNA biosynthesis | SARS2;FARS2;SARS1;EARS2;PARS2;EPRS1;CARS2;DARS2;RARS2;RARS1;LARS1;LARS2;QRSL1;IARS1;IARS2;WARS2;MARS1;MARS2;WARS1;VARS2;VARS1;LOC119870113;AARS1;AARS2;GATB;GATC;TARS1;TARS3;TARS2;LOC475406;NARS1;YARS1;YARS2;SEPSECS;MTFMT;KARS1;QARS1;FARSB;FARSA;CARS1;NARS2;PSTK;HARS2;HARS1;GARS1 |
| JAK-STAT signaling pathway | LIF;LOC100856339;CRLF2;GHR;PDGFRB;IL6ST;IFNAR2;AKT1;AKT2;AKT3;LIFR;TYK2;EGF;IFNLR1;IL21;IL12RB1;PIK3CA;PIK3CB;IL12RB2;PIK3CD;OSM;MYC;PIK3R2;IFNK;IL19;IFNG;CSF3R;LOC119876828;SOCS5;SOCS4;SOCS7;SOCS6;SOCS1;GH1;SOCS3;SOCS2;CCND1;CCND3;CCND2;CSF2;CSF3;IFNAR1;PTPN11;IL7R;IL20RB;IL10RA;IL10RB;PDGFA;CDKN1A;IL12A(up);IFNGR1;IFNGR2;STAM;IL6;BCL2L1(down);STAT5B;PRLR;IFNL1;IL4;IL7;GRB2;JAK2;JAK3;PIK3R1;JAK1;IL22RA2;IL22RA1;IL5RA;IL4R;LOC119870217;LOC119870216;LOC119870215;LOC100684658;PTPN2;PTPN6;EPOR;IL13RA2;IL13RA1;LOC100683567;IRF9;IL5;PRL;IL11RA;IL9;IL15RA;CREBBP;IL20RA;PDGFRA;CNTFR;CSF2RA;LOC102154108;LEP;RAF1;LOC611406;STAT6;STAT4;STAT3;STAT2;STAT1;IL11(up);CSF2RB;IFNB1;FHL1;CISH;HRAS;IL17D;IL29L;CTF1;LOC100686162;IL6R;IL3RA;EP300;IL2RA;IL2RB;SOS2;IL2RG;IL10;IL21R;IFNA7;IL15;IFNA5;PIAS2;PIAS3;STAT5A;PIAS1;IL2;IL3;PIAS4;MPL;MCL1;AOX2;AOX4;IL23A;EGFR;IL12B;IL23R;TSLP;STAM2;THPO;OSMR;LOC100683481;GFAP;CNTF;PDGFB;PIM1;LEPR;IL22;IL13;IL27RA;EPO;MTOR;BCL2 |
| Base excision repair | LOC609805;HMGB1;LOC100682891;MUTYH;POLE;FEN1;POLB;XRCC1;POLL;OGG1;APEX1;POLD1;POLD2;APEX2;LIG1;LIG3;NEIL1;PCNA;PARP4;PARP1;PARP2;PARP3;UNG;SMUG1;MPG;LOC611847;MBD4;POLE4;POLE3;POLE2;NEIL3;NEIL2;POLD3;NTHL1 |
| Yersinia infection | LOC100856339;ACTG1;CRKL;IKBKB;AKT1;PTK2B;AKT3;LOC111090226;ARHGEF7;PIK3CB;PIK3CD;MAP3K7;ARHGEF1;LOC106559121;RELA;IRAK1;LOC102156003;ZAP70;ARHGEF12;LOC119879788;LOC106559212;CD4;FN1;LOC607467;ARPC1B;ARPC1A;AKT2;GSK3B;PIP5K1C;LAT;ACTB;ELMO1;CCL2;NFATC1;NFATC2;NFATC3;CXCL8(up);DOCK1;ITGA4;ITGA5;CRK;LOC106559111;JUN;LOC102156901;LOC119870217;PIK3R2;PIK3R1;LOC119870216;MYD88;LOC119869025;FOS;ARPC3;ARPC2;LOC119870215;LOC119872979;ARPC5;ARPC4;RHOA;BCAR1;IRF3;RPS6KA1;RPS6KA2;RAC2(up);TBK1;RPS6KA6;VAV1;VAV3;VAV2;GIT2;PIK3CA;BAIAP2;CDC42;LCK;LOC102156776;MAPK14;MAPK10;MAPK11;MAPK12;MAPK13;IL1B;LOC106559087;TICAM1;NLRP3;LOC102153980;ARF6;IFNB1;MAPK3;MAPK1;NFKB1;MAPK8;MAPK9;SRC;NFKBIA;TLR4(down);LOC111097181;CHUK;CD8A;CD8B;WAS;LOC119866928;GNAQ;IL18;IL10;PIP5K1B;PIP5K1A;ARPC5L;MAP2K1;IL6;IL2;IRAK4;MAP2K6;PTK2;LOC119869603;LIMK1;LOC119869607;MAP2K3;MAP2K2;PLCG1;MAP2K7;WASL;MAP2K4;FYB1;WASF2;PXN;LOC609053;PYCARD;ITGB1;TRAF2;RAC3;TRAF6;RAC1;PKN1;PKN3;PKN2;TNF;LOC119869046;LCP2;LOC480788;TAB2;TAB1;ROCK1;ROCK2;WIPF3;WIPF2;WIPF1;LOC102153882;LOC102154760 |
| Thyroid hormone synthesis | DUOXA2;FXYD2;HSPA5;DUOX1;TSHB;ADCY1\_1;TTF2;HSP90B1;TTF1;GSR;SLC5A5;TSHR;CANX;GPX1;LRP2(up);GPX7;SERPINA7;CGA;ATP1A3;PRKCA;CREB3L1;DUOX2;TTR;IYD(up);TG;LOC489461;ALB;CREB5;GPX6;LOC119869567;PLCB2;PLCB3;PLCB4;CREB3L4;GPX2;TPO;CREB3;ATP1B4;PRKCB;ATP1B2;ATP1B3;PRKCG;ATP1B1;ADCY4(up);PLCB1(up);ASGR2;SLC26A4;CREB1;GPX8;ITPR2;ITPR3;ITPR1;PAX8;PDIA4;ADCY1;ADCY5;ADCY6;ADCY7;GNAQ;ADCY2;GNAS;ADCY3;ADCY8;ADCY9;CREB3L2;CREB3L3;GPX3;ATP1A4;PRKACA;ATP1A1;PRKACB;GPX5;ATF4;ATF6B;ATF2 |
| Polyketide sugar unit biosynthesis | TGDS |
| Amino sugar and nucleotide sugar metabolism | GALT;CYB5R4;CYB5R3;CYB5R2;CYB5R1;GNPDA2;GNPDA1;GFUS;GNE;PMM2;GNPNAT1;CHIA;UGP2;PMM1;LOC483833;HKDC1;FCSK;LOC479379;UGDH;AMDHD2;GMDS;NANP;NANS;MPI;RENBP;HK1;LOC610444;GPI;UXS1;HK2;HK3;GCK;UAP1;GMPPA;PGM1;PGM2;PGM3;GALE;FPGT;LOC488248;GMPPB;NAGK;GALK1;HEXA;NPL(up);HEXB;LOC606974;NUDT11;GFPT1;GFPT2;CYB5RL |
| Streptomycin biosynthesis | ISYNA1;TGDS;HK2;HK3;GCK;HK1;PGM1;IMPA1;IMPA2;HKDC1 |
| Dopaminergic synapse | SLC6A3;PPP2R3B;PPP2R3C;PPP2R3A;AKT1;AKT2;AKT3;CALM2;CALM3;CALM1;GNG14;CACNA1A;CACNA1B;CACNA1C;GNG10;GNG11;GNG12;GNG13;LOC119863873;CAMK2D;PPP2R2D;CREB5;CAMK2G;CREB3;CAMK2A;CREB1;ADCY5;PPP2CA;KIF5A;KIF5B;PPP2CB;GSK3A;GSK3B;GNG7;GNG4;GNG5;GNG2;GNG3;ARRB2;GNG8;KIF5C;GRIA2;GNAO1;ARRB1;GRIA4;ARNTL;PPP1R1B;LOC100855681;LOC106560171;PLCB2;PLCB3;PLCB4;FOS;LOC119870217;LOC119870216;LOC119870215;GNAI1(up);DDC;ITPR3;ITPR1;GNAQ;GNAS;CALML6;CALML5;CALML4;MAOB;MAOA;GNAL;SLC18A2;SLC18A1;PPP2R5A;PPP2R5B;PPP2R5C;CLOCK;GNB4(up);LOC102153034(down);MAPK14;ITPR2;MAPK10;MAPK11;MAPK12;MAPK13;GNAI2;GNAI3;GRIA3;MAPK8;MAPK9;GRIA1;GNB5;GNB1;COMT;GNB3;GNB2;PLCB1(up);PPP2R2C;KCNJ3;KCNJ6;KCNJ5;KCNJ9;PRKACA;PRKACB;PPP2R1A;PPP2R1B;GRIN2B;GRIN2A;TH;PPP2R2B(up);CACNA1D;GNGT1;PRKCA;PPP2R2A;PRKCB;PRKCG;PPP2R5D;CAMK2B;ATF4;PPP2R5E;ATF6B;CALY;PPP1CB;PPP1CC;PPP1CA;DRD4;DRD5;DRD2;DRD3;DRD1;CREB3L2;CREB3L3;CREB3L1;GNGT2;CREB3L4;SCN1A;PPP3CA;PPP3CB;PPP3CC;ATF2 |
| Neomycin, kanamycin and gentamicin biosynthesis | HK2;HK3;GCK;HK1;HKDC1 |
| Acarbose and validamycin biosynthesis | TGDS |
| Sulfur relay system | NFS1;CTU2;URM1;MOCS3;MOCS2;MPST;CTU1;LOC484342;TST |
| Cholinergic synapse | CHRNB4(down);LOC100856339;FYN;ADCY1\_1;AKT1;AKT2;AKT3;ACHE(down);GNG14;CACNA1A;CACNA1B;CACNA1C;GNG10;GNG11;GNG12;GNG13;LOC119863873;CACNA1S;CAMK2D;CREB5;CAMK2G;CREB3;CAMK2A;CREB1;ADCY4(up);KRAS;CHRNA7(down);ADCY5;ADCY6;ADCY7;ADCY1;ADCY2;ADCY3;ADCY8;ADCY9;GNG7;GNG4;GNG5;GNG2;GNG3;GNG8;GNAO1;HRAS;LOC119870215;LOC100855681;JAK2;PIK3R2;PIK3R1;CHRM5;LOC119869567;PLCB2;PLCB3;PLCB4;FOS;LOC119870217;LOC119870216;KCNQ4;KCNQ5;KCNQ2;KCNQ3;KCNQ1;PRKCG;ITPR2;ITPR3;GNAI1(up);GNAQ;PIK3CA;SLC18A3;PIK3CB;GNB4(up);CAMK4;CACNA1F;CHAT;GNAI2;GNAI3;ITPR1;CHRNB2;MAPK3;MAPK1;GNB5;GNB1;GNB3;GNB2;CHRNA4;PLCB1(up);CHRNA6;CHRNA3;KCNJ3;KCNJ6;KCNJ4;CHRM4;PRKACA;PRKACB;MAP2K1;SLC5A7;NRAS;KCNJ12;GNGT1;KCNJ14;PRKCA;KCNJ2(down);PRKCB;CHRM3;CHRM2;CHRM1;PIK3CD;CAMK2B;CACNA1D;CREB3L2;CREB3L3;CREB3L1;GNGT2;CREB3L4;GNA11;ATF4;BCL2 |
| Ubiquitin mediated proteolysis | UBE2Q1;LOC119870849;WWP2;UBE2J1;UBE2J2;WWP1;HUWE1;PPIL2;UBE2Q2;NHLRC1;STUB1;MAP3K1;ANAPC11;ANAPC10;ANAPC13;LOC119863875;LOC119870395;CUL5;CUL7;CUL1;CUL2;CUL3;BRCA1;UBE2G1;LOC610206;UBE2R2;MDM2;UBR5;KLHL9;LOC481325;UBE4B;UBE4A;SOCS1;SOCS3;FZR1;XIAP;UBA52;UBC;RNF7;FBXW11;LOC102155658;TRIM32;RPS27A;TRIM37;UBE2D3;LOC119866339;UBE2D1;UBE2L6;UBE2L3;UBE2A;SMURF1;SMURF2;CDC16;KEAP1;UBE3B;UBE3C;UBE3A;UBE2E3;LOC475563;UBE2E1;LOC102155320;LOC100685549;UBE2E2;UBA6;UBA7;HERC3;HERC2;UBA2;HERC4;RBX1;ANAPC1;ANAPC2;ANAPC5;ANAPC4;ANAPC7;UBE2I;HERC1;UBE2K;UBE2M;UBE2O;UBE2N;CDC27;CDC26;UBE2S(up);UBE2B;CDC23;CBL;CDC20;UBE2D2;UBE2Z;LOC111094434(up);UBE2U;UBE2W;UBA3;CDC34;UBA1;FBXO2;FBXO4;ELOC;FBXW8;SKP2;SKP1;LOC607817;SYVN1;KLHL13(up);LOC102156563;DDB2;DDB1;FBXW7;SIAH1;UBE2H;BIRC3;PRKN;PML;DET1;LOC106558148;FANCL;PIAS2;PIAS3;PIAS1;PIAS4;MGRN1;UBE2C;ERCC8;ITCH;BIRC7;BIRC6;LOC100685367;BIRC2;VHL;UBE2F;UBE2QL1;MID1;RCHY1;PRPF19;COP1;UBE2G2;ELOB;NEDD4;UBOX5;TRAF6;LOC100856010;RHOBTB2;RHOBTB1;TRIP12;AIRE;BTRC;RNFT2;CUL4A;NEDD4L;SAE1;CUL4B;LOC111090648 |
| GABAergic synapse | SLC6A1;ADCY1\_1;GNG14;CACNA1A;CACNA1B;CACNA1C;GNG10;GNG11;GNG12;GNG13;LOC119863873;GABRR1;CACNA1S;GABRR2;SLC38A5;SLC38A1;SLC38A2;ADCY4(up);GABRG2;ADCY5;ADCY6;ADCY7;ADCY1;ADCY2;ADCY3;ADCY8;ADCY9;GNG7;GNG4;GNG5;GNG2;GNG3;LOC483960;GNG8;GABRG3;GNAO1;GABRG1;GLUL;GABBR1;GLS;GABRA1;LOC100855681;GAD1;LOC119869567;GNAI1(up);GPHN;GABRQ;GABRP;GABRE;GABRD;GABBR2(up);GNB4(up);SLC32A1;GABRR3;LOC612295;CACNA1F;GABRA5;GABRA4;GABRA6;GNAI2;GNAI3;GABRA3;GABRA2;TRAK2;SRC;GNB5;GABARAP;GNB1;GNB3;GNB2;GABRB1;GABRB2;GABRB3;KCNJ6;PRKACA;PRKACB;GLS2;ABAT;GABARAPL1;GABARAPL2;NSF;SLC38A3(down);PRKCA;PRKCB;PRKCG;PLCL1;CACNA1D;HAP1;GNGT2;GNGT1 |
| Basal transcription factors | LOC100685309;TAF15;ERCC2;ERCC3;TAF13;TAF12;TAF11;TAF10;GTF2A1;GTF2A2;GTF2E1;GTF2E2;LOC100685931;TAF6L;LOC102152033;TAF4B;GTF2B;GTF2A1L;LOC119864634;GTF2I;LOC478089;GTF2H3;GTF2H1;TAF5;GTF2H5;GTF2IRD1;GTF2F1;MNAT1;LOC102152240;TAF7;TBPL2;TBPL1;TAF4;TAF3;TAF2;TAF1;GTF2F2;TBP;TAF8;CDK7;TAF9B;TAF5L;CCNH |
| Tight junction | ACTG1;HSPA4;PRKAG1;PRKAG3;STK11;MYL6;RAPGEF6;TUBA8;MYL2;RAPGEF2;MYH14;MYH15;DLG1;MYH16;TIAM1;MAP3K1;CACNA1D;PARD6B;PARD6A;MAP3K5;MAGI1;ERBB2;LOC607207;PRKAG2;PPP2R2D;PPP2R2A;PPP2R2C;ITGB1;GATA4;LOC100855928;LOC100687704;PPP2CA;ARPC1B;ARPC1A;PPP2CB;CLDN5;CCND1;CD1A8;LOC608848;PARD6G(up);ACTB;CFTR;YBX3;PCNA;CD1D;ARPC5L;CD1B;CD1C;CTTN;DLG2(up);TUBA1C;JAM3;JUN;CLDN8;CLDN9;NF2;HCLS1(up);CLDN1;CLDN2;CLDN3;CLDN4;MPDZ;CLDN6;CLDN7;PRKAB2;PRKAB1;ARPC3;ARPC2;ARPC5;ARPC4;MYH1;RHOA;CD1A6;F11R;TUBAL3;LOC610636;CDK4;AMOT;LOC477570;MYH10;MYH11;CLDN10;CLDN11;CLDN16;CLDN17;CLDN14;CLDN15;CLDN18;CLDN19;RAP1A;CD1E;MAPK10;MPP5;MYH2;MYH3;RAB8A;MYH4;CGN;MYH8;MYH9;NEDD4;RAB13;MAPK9;SRC;RDX;OCLN;LOC612475;WAS;MAPK8;LOC119866928;TJP2;TJP3;TJP1;LLGL2;MYL12B;LLGL1;AFDN;WASL;PRKACA;PRKACB;MYL6B;CDC42;AMOTL1;PPP2R1A;PPP2R1B;AMOTL2;SCRIB;MYL9;IGSF5;PRKAA2;PRKAA1;MSN;MAP2K7;ROCK2;PATJ;LOC119863892;LOC106557476;LOC608051;PRKCI;RAC3;RAC1;CLDN23;CLDN20;PRKCE;CLDN25;CLDN24;PRKCZ;CRB3;TUBA4A;VASP;TJAP1;ACTN1;PARD3;MYH7B;ACTN4;MYH13;ROCK1;EZR;SYMPK;NEDD4L;LOC482919;PPP2R2B(up);LOC119866377;LOC491914 |
| Geraniol degradation | HMGCL;HADH;HMGCLL1 |
| Valine, leucine and isoleucine degradation | AOX2;ACADS;AOX4;PCCB;PCCA;HSD17B10;AGXT2;AACS;ABAT;HIBADH;ACADM;ACAA2;HADHB;HADHA;ACAT2;ACAT1;ACAA1;HMGCLL1;OXCT1;IL4I1;HMGCS1;HMGCS2;DLD;HMGCL;AUH;LOC482436;ACSF3;BCAT2;BCAT1;MCEE;MMUT;ALDH6A1;ALDH9A1;ACADSB;ALDH7A1;ALDH1B1;EHHADH;MCCC2;MCCC1;LOC481043;IVD;HADH;ACAD8;ALDH2;HIBCH;ECHS1;BCKDHB;BCKDHA;DBT |
| MAPK signaling pathway - yeast | PI4KA;CAT;MAPK14;GPD1;MAPK11;MAPK12;MAPK13;TEAD3;TEAD2;TEAD1;TEAD4;NEDD4;MAPK3;MAPK1;MAPK7;YWHAE;PRKCA;PAF1;GNB1;PRMT5;PDPK1;RHOA;LOC119866928;PIP5K1C;PIP5K1B;PIP5K1A;GPD1L;LOC608573;PAK1;CDC42 |
| Glucagon signaling pathway | PCK2;PCK1;PPP3R2;PPP3R1;PRKAG1;PRKAG3;PRKAG2;AKT1;AKT2;AKT3;FBP2;LOC100686034;PHKB;PPP4R3A;PPP4R3B;CALM2;CALM3;CALM1;PPP4C;PYGB;PYGL;PYGM;CAMK2D;CREB5;PRMT1;CREB3;CAMK2A;CREB1;ACACB(down);LOC490690;LOC608800;ADCY2;SLC2A2;SLC2A1;PHKG2;CAMK2G;G6PC1;SIRT1;G6PC3;G6PC2;CRTC2;PRKAB2;PPARGC1A;PDHB;GCK;PKM;PLCB2;PLCB3;PLCB4;GCG;PRKAB1;LOC119870217;LOC119870216;LOC119870215;PHKA1;LOC100684709;GYS1;GYS2;PPARA;ITPR2;ITPR3;ITPR1;GNAQ;GNAS;CALML6;CALML5;CALML4;CREBBP;LOC102153034(down);PGAM2;PGAM1;SIK1;SIK2;LOC491791;PDE3B;PDHA2;ACACA;PLCB1(up);EP300;PRKACA;PRKACB;LDHAL6B;PRKAA2;PRKAA1;FOXO1;PFKP;PFKM;PFKL;LDHA;LDHB;LDHC;CPT1A;CPT1C;CPT1B;LOC609911;LOC102153001;CAMK2B;ATF4;GCGR;PFKFB1;CREB3L2;CREB3L3;CREB3L1;FBP1(up);CREB3L4;PPP3CA;PPP3CB;PPP3CC;PHKG1(up);ATF2 |
| Pantothenate and CoA biosynthesis | PANK1;PPCDC;COASY;PANK2;ALDH2;DPYD;VNN3;LOC102155332;VNN1;PANK3;ENPP3(down);ENPP1;DPYS;GADL1;ALDH1B1;BCAT1;UPB1(up);BCAT2;PPCS;AASDHPPT |
| Parathyroid hormone synthesis, secretion and action | PTH1R;MMP14;TNFSF11;VDR;BCL2;NR4A2(up);CDKN1A;ADCY1\_1;EGFR;ATF2;MEF2D;MAP2K1;SOST;ARRB2;ARRB1;RXRG;GNAI2;GNAI3;PLD2;MEF2C;JUND;RXRB;PTH;RAF1;HBEGF;ARAF;ARHGEF1;MAPK3;MAPK1;LRP5;CYP27B1;TRPV5;GNA12;FGFR1;GNA13;LOC119869567;ARHGEF11;PLCB2;PLCB3;PLCB4;FOS;PRKCA;SP1;CREB5;PRKACB;CREB3;CREB3L1;PRKCG;GNAI1(up);ADCY4(up);PLCB1(up);PRKCB;CREB1;RXRA;ATF4;ITPR2;ITPR3;RHOA;ITPR1;ATF6B;ADCY1;ADCY5;ADCY6;ADCY7;LRP6;GNAQ;ADCY2;ADCY3;GNAS;ADCY8;ADCY9;KL(up);CREB3L2;CREB3L3;MEF2A;CYP24A1(up);PRKACA;BRAF;CREB3L4;GNA11;PDE4A;PDE4B;PDE4C;PDE4D;PLD1 |
| Insulin secretion | FXYD2;ADCY5;ADCY1\_1;RAPGEF4;CCK;ADCY6;RYR2;CACNA1D;KCNJ11(down);CACNA1F;KCNU1;GLP1R;CACNA1S;GIP;CAMK2D;ATP1B4;CAMK2G;ATP1B2;CAMK2A;CAMK2B;ATP1B1;ADCY4(up);KCNN4;KCNN3;KCNN2;KCNN1;ADCY1;KCNMB1;SNAP25;KCNMB3;ADCY7;KCNMB4;ADCY2;ADCY3;ADCY8;ADCY9;SLC2A1;GPR119;SLC2A2;KCNMA1;PCLO;TRPM4;PRKACA;LOC119869567;PLCB2;PLCB3;PLCB4;GCG;GCK;ITPR3;GNAQ;GNAS;INS;PDX1;STX1A;CACNA1C;ADCYAP1;RIMS2;PLCB1(up);KCNMB2;CCKAR;ATP1A4;ATP1A3;ATP1A1;PRKACB;ABCC8(down);ADCYAP1R1;CREB5;PRKCA;CREB3;PRKCB;CHRM3;PRKCG;ATP1B3;CREB1;RAB3A;FFAR1;VAMP2;CREB3L2;CREB3L3;CREB3L1;CREB3L4;GNA11;ATF4;ATF6B;ATF2 |
| Collecting duct acid secretion | ATP6V1D;ATP6V1F;ATP6V1A;ATP6V0D2(up);SLC4A1;ATP6V0A2;ATP6V0A4;LOC100686787;ATP6V1C1;LOC481939;SLC12A7;ATP6V1B1;ATP6V1B2;ATP6V0A1;ATP6V0C;ATP6V1G1;LOC102152234;ATP6V1C2;ATP6V0E2;ATP4A;ATP4B;ATP6V1E1;ATP6V1E2;TCIRG1;ATP6V0D1;ATP6V1G3;ATP6V1G2;LOC111090910;ATP6V0E1;LOC119870343 |
| Cellular senescence | LOC100856339;PPP3R2;PPP3R1;AKT1;AKT2;AKT3;MDM2;CALM2;CALM3;CALM1;GADD45A;PIK3CB;PIK3CD;GADD45G;CAPN2;CAPN1;TRPV4;MYC;CDKN2B;DLA-79;LOC102153034(down);SLC25A6;SLC25A5;SLC25A4;GATA4;KRAS;CDKN2B\_1(down);CCND1;CCND3;CCND2;PTEN;NFATC1;NFATC2;NFATC3;NFATC4;CXCL8(up);HRAS;RRAS2;RASSF5;RRAS;SLC25A31;RAD1;IL1A(up);MAPKAPK2;CCNE2;RAF1;CCNE1;PIK3R2;PIK3R1;LOC119870216;TRPM7;CHEK2;LOC119870215;CHEK1;SIRT1;LOC119870217;MRE11;CDC25A;DLA88;MRAS;IGFBP3;ITPR2;ITPR3;ITPR1;TP53;CALML6;CALML5;CALML4;EIF4EBP1;PIK3CA;GADD45B;CACNA1D;ATM;LOC487020;SMAD2;SMAD3;MAPK14;FBXW11;ATR;MAPK11;MAPK12;MAPK13;VDAC3;VDAC2;VDAC1;TSC2;TSC1;DLA-64;MAPK3;MAPK1;NBN;NFKB1;CDKN1A;TGFBR2;TGFBR1;RBL1;RBL2;E2F5;MTOR;PPP1CB;LOC612475;RELA;E2F2;MAP2K3;FOXO1;LOC111094784;IL6;CDK1;CDK2;CDK4;CDK6;RAD50;RHEB;TGFB1;TGFB2;TGFB3;LOC609798;FOXO3;MAP2K2;MAP2K1;MAP2K6;CCNA2;CCNA1;NRAS;RB1;SERPINE1;ETS1;SQSTM1;CCNB1;E2F4;E2F3;PPP1CC;E2F1;PPP1CA;BTRC;PPP3CA;PPP3CB;PPP3CC |
| One carbon pool by folate | TYMS;LOC485342;MTHFD2L;ATIC;MTR;MTHFD2;MTHFD1;MTFMT;DHFR;MTHFS;ALDH1L1;MTHFD1L;SHMT2;MTHFR;FTCD;SHMT1;ALDH1L2;GART;AMT;LOC609048 |
| Metabolic pathways | PGM2L1;AGL;AGK;ALDH7A1;LOC102156968;PMM2;PMM1;ASS1;SQLE;HKDC1;PIK3CA;PIK3CB;PIK3CD;SPTLC2;SPTLC3;HMGCLL1;SCLY;LOC482436;PIK3C3;GK;ACLY;UGCG;ALDH3A1;LOC482320;MGST3;CYP2U1;DGAT1;DGAT2;GMDS;CYP27B1;CHST9;CHST8;MOGAT3;GART;PISD;HLCS;TCIRG1;FTCD;MINPP1;SPR;GANAB;SC5D;MIOX;CTH;LOC100855914;FAHD1;SPTLC1;COX4I2;COX4I1;LOC478277;PIPOX;LOC100686510;LOC119870343;SPHK2;SPHK1;LOC102151754;MDH1;NADSYN1;ABAT;RDH8;DPYD;NAPRT;DPYS;LOC100688446;ETNK2;ETNK1;LOC480667;ATP6V0A2;UXS1;CA15;CA14;CA13;CA12;FPGT;FPGS;GPT2;PNP;ATP6V0E1;ATP6V0E2;CHPF;PCK2;RPN1;RPN2;PCK1;LOC102156885;CDO1;NQO1;LOC100685227;GUK1;PIP4K2A;PIP4K2C;PIP4K2B;TYRP1;PDE8A;PDE8B;DBH;CSAD;HSD3B7;HSD3B2;UQCRFS1;UQCRC2;DBT;UQCRC1;GGCX;NSDHL;LOC102154822;GGCT;PPOX;TKT;PLCH1;FDFT1;LOC106560171;LOC119868057;LOC477647;NUDT9;DLD;GCK;NUDT5;TK2;TK1;CYP2R1;ATP5F1D;CYP8B1;GLYCTK;ATP5F1A;ATP5F1B;ATP5F1C;CYP27A1;BTD;COX10;COX11;LOC476602;COX15;COX17;EPHX2;APIP;AMPD2;AMPD1;UGT2A3;QPRT;MECOM;IPPK;PLPP1;PLPP3;MTMR14;UGDH;SEPHS2;SEPHS1;MGST2;DLST;LOC607806;LOC119863926;HPSE(up);PPAT;LOC111090910;ST6GAL2;ST6GAL1;PRDX6;LOC100688724;EBP;LOC119870407;ST3GAL3;ST3GAL2;ST3GAL1;ST3GAL6;ST3GAL5;CSGALNACT1(up);GALNS;BCKDHB;CANT1;BCKDHA;LOC100683828;CKM;LOC485179;KMT5A;TIGAR;UQCRQ;UQCRH;B3GAT3;B3GAT2;B3GAT1;GPAM;DCK;ACSM3;DNMT1;ACSM5;CYP51A1;GLO1;ATP6V0D1;LSS;HMGCL;ADCY4(up);LOC100685720;HMGCR;LOC119872260;PLA2G2C(down);SETD1B;LOC102152620;ACADS;GBA;GAPDH;LOC119880300;ACADL;ACADM;XDH;UMPS;SGSH;ATP5PO;ACAD8;CYP2E1;NNMT;LOC119868043;PTDSS2;KMO;PLCE1;LOC102154109;CYP2S1;ADPRM;ADI1;CTPS2;CTPS1;LOC609321;PDHA2;HMGCS1;HMGCS2;GALNTL6;PLAAT3(down);SRM;SRR;GALE;GALC;CS;GALM;CKMT2;AGPAT5;AGPAT4;AGPAT3;AGPAT2;AGPAT1;OCRL;IDO1;LDHAL6B;LOC106557778;ATP6V0D2(up);ALDH1A2;ALDH1A3;GCNT4;GCNT1;GCNT3;GCNT2;LPCAT4;LPCAT2;LPCAT1;PIGA;PIGB;PIGC;AADAT;PIGF;PIGH;PIGK;PIGL;PIGM;PIGN;PIGO;PIGQ;PIGS;PIGT;PIGU;PIGV;PIGW;PIGX;PIGY;HPD;LOC474938;RDH16;RDH10;RDH11;RDH12;ALPI;ALPL;DOLK;PTGS2;PTGS1;DMGDH;FBP2;ENOSF1;ALLC;LOC100688420;ADH5;LOC490690;MLYCD;ACYP1;ACYP2;ACSL3;ACSL1;ACSL6;MBOAT1;ACSL4;CYP1A2(up);LOC106558345;AMD1;G6PC1;G6PC3;G6PC2;GFUS;LIAS;UROC1;SCD;LOC489851;PTS;PIKFYVE;UAP1;ACO1;PTGES;ACO2;ETHE1;KDSR;LOC102151775;MPI;GAMT;UGT8;LAP3;GADL1;LOC102153059;QRSL1;KL(up);BDH2;CYP2B6;ASH1L;ATP12A;PLCD4;PLCD3;ADA;ADO;LOC102152275;ADK;B3GALNT1;ADH4;LOC476397;LOC102155863;LOC102154590;GGT1;GNMT;TALDO1;HADHB;HADHA;ITPA;IDNK;ACOT12;HSD11B2;HSD11B1;ENOPH1;ISYNA1;ESD;ALDH1B1;COX7A2L;LOC111091468;PMVK;SDHA;SDHC;SDHB;SDHD;PPCS;LOC100682956;MOCS2;OGDH;TKTL1;NADK2;B3GALT4;NPR1;NPR2;B3GALT1;B3GALT2;BCAT2;BCAT1;LOC608800;UCKL1;CYP26A1;LOC483960;LOC102151507;LOC102151506;PTGDS;CHKA;CHKB;LTC4S;MRI1;GUCY2C;GUCY2D;GUCY2F;UCK1;UCK2;DSE;CSGALNACT2;SPAM1;GSTO2;GSTO1;GFPT1;GFPT2;LOC100685416;LOC100685413;ACP2;GLS;LOC100685679;CDIPT;PLA2G7;B4GALNT3;NT5C3A;NT5C3B;ACAT1;ACAT2;LOC610338;MECR;AUH;MCAT;SLC27A5;PDE10A;GUCY1B1;AK9;AK8;LOC100682717;AK2;AK1;ATP5F1E;AK7;AK6;AK4;DHFR;PLCG2;PLCG1;BHMT2;CHDH(down);LPIN2;LPIN3;LPIN1;LOC479379;SELENOI;ATP6V1B1;ATP6V1B2;PIP5KL1;GANC;TPH2;LOC100685007;BAAT;CYP11B2;GRHPR;IDI1;IPMK;DCTPP1;B3GNT4;B3GNT5;B3GNT6;SETD2;B3GNT2;DHRS9;DHRS4;DHRS3;LOC102154025;MAN1A2;MTMR6;MTMR4;MTMR3;MTMR2;MTMR1;NME4;TPO;PCYT2;NME6;IMPA1;IMPA2;GPLD1(down);PHGDH;LOC481043;NTPCR;PTGES3;PTGES2;ECHS1;CYP4A38(down);LOC102154742;LOC485342;HNMT(up);CYP26B1;AGMAT;RFK;SDSL;ATP6V0A4;SARDH;ATP6V0A1;LOC102151792;PLCZ1;SMPD4;SMPD1;HAAO;SMPD2;PDE11A;VKORC1;OXSM;AMDHD1;AMDHD2;DOT1L;ND1;CA5B;CA5A;ENO1;ENO2;ENO3;ENO4;ACOX2;ACOX3;ACOX1;ND4L;LOC610304;IDO2(down);CEPT1;MGLL;PLOD1;HEXD;CYP17A1;ALOX15;LOC119881513;ALOX12;GAL3ST1;LOC102152523;ODC1;CERS3(up);FUT7;INPPL1;LOC476006;FUT4;LOC111089999;NANP;NANS;PNMT;MGAT4C;NAT8L;BDH1;PFKFB1;PFKFB3;PFKFB2;TYMS;GSTP1;ATP6V1C2;ATP6V1C1;PLCD1;CHSY3;CHSY1;TPI1;LOC119866697;GGPS1;PLA2G6;GSTA4;PLA2G5;LOC609990;PLA2G3;SETDB1;SETDB2;NDUFB11;HAL;TBXAS1;SMPD3(up);CAMKMT;IDH2;IDH1;MIF;EHHADH;SETD7;PCYT1A;INPP5A;INPP5B;NME7;INPP5D;INPP5E;NME2;NME3;B4GALT5;INPP5J;INPP5K;UPB1(up);PGK1;GPX4;LOC100688835;RGN;NT5M;NT5C;DCTD;NT5E;PNPLA3;PNPLA2;RRM2B;LOC100684709;LOC100688166;MOGS;ST8SIA1;DPAGT1;LOC483943;CYP26C1;LIPT2;LOC100684412;CHIA;CARNMT1;PDE3A;PDE3B;NMNAT2;NMNAT3;NMNAT1;ALOX12B;NDUFA6;PPCDC;NDUFA4;NDUFA5;NDUFA2;NDUFA3;NDUFA1;ACSS3;ACSS2;ACSS1;COMT;NDUFA8;NDUFA9;MMUT;GLCE;AGXT;GPAA1;GSTZ1;COLGALT2;GNS;SAT2;COLGALT1;BPGM;GNE;LTA4H;DAD1;DAO(down);CBR3;GBE1;NT5C1A;OGDHL;SUOX;LOC102153001;GCLM;HPGDS;SDS;A4GALT(up);NDUFS1;NDUFS2;NDUFS3;NDUFS4;NDUFS5;NDUFS6;PRUNE1;COQ5;COQ7;AHCY;COQ3;COQ2;AOC1;AOC3;AOC2;LOC479911;LOC479912;LOC102155410;PI4K2B;PI4K2A;HIBADH;LOC100686034;NDUFA4L2(down);PPT1;PPT2;INPP4B;LOC484867;NDUFB10;PYGB;COX8A;GOT2;GOT1;PYGL;PYGM;PFAS;LOC479821;LOC479822;LOC612644;PLA2G4E(up);LOC102154094;LOC481841;LOC481849;NIT2;TRAK2;EPRS1;UGT1A6(down);PGS1;LOC477508;LOC481939;PGM1;PGM2;PGM3;LOC478413;GCH1;ELOVL6;ELOVL7;ELOVL4;ELOVL5;ELOVL2;ELOVL3;ELOVL1;GGT5;MARS1;TECR;MGAT5B;BCO1;ALDH18A1;SGPP1;GBGT1;LOC485435;SHPK;QDPR;FADS1;FADS2;IDUA;GLB1;DGUOK;XYLB;TMLHE;NDUFA7;CD38(up);LOC119865581;CARNS1;IDH3A;IDH3B;IDH3G;HAGH;CMPK1;CMPK2;DGKQ;VKORC1L1;DGKK;DGKH;DGKI;GALNT5(up);ATP5MG;ATP5ME;DGKA;DGKG;DGKD;DGKE;DNMT3B;LOC111092022;DNMT3A;LOC100683724;PTGIS;ALDH6A1;INPP1;PEMT;AKR1D1;TXNDC12;LOC102152234;PLA2G12A;ACOD1;P4HA2;P4HA3;P4HA1;HPSE2;LOC119867218;TAT;LOC119872525;ATP8;ATP6;ACACB(down);PIK3C2B;PIK3C2A;PIK3C2G;FBP1(up);CNDP1;CNDP2;CDA;LOC119870901;LOC102154486;PTEN;LOC476372;TPK1;TST;ALDH1A1(down);COX7B2;CYP11A1;ST8SIA5(up);PAICS;LOC100684983;KYAT1;KYAT3;NUDT2;MTHFD1L;MBOAT2;LOC612295;KMT5B;BLVRB;HMBS;B3GNT3(down);LOC611724;ALAS1;ALAS2;CPOX;ALG11;ALG12;ALG13;ALG14;HACD4;NPL(up);LOC489397;PGLS;SI;LOC100688697;LOC119876526;GLS2;B4GALNT4;B4GALNT1;B3GALT5;B3GALT6;CAT;ATP5MC1;ATP5MC3;ATP5MC2;CAD;ACER2;ACER3;PGD;ACER1;PDE1C;PDE1B;PDE1A;IMPDH1;IMPDH2;NDUFC1;NDUFC2;ENTPD2(down);SEPSECS;LOC119866771;CA9;CA8;ARSB;HEXA;CA1;HEXB;CA7;CA6;LOC119864605;MGAT4D;LOC100856397;FUT2;FUT1;MGAT4B;FUT9;FUT8;LOC100855615;TUSC3;ATP5J2;AGXT2;PYCR3;PYCR2;PYCR1;LOC100686830;NT5C1B;LOC610444;LIPF;SCD5;AMACR;PAFAH1B1;PAFAH1B3;HOGA1;LOC102151128(down);NT5C2;LOC111094513;KMT2B;NMRK1;HDC;PLA2G1B;PLA2G12B;MGAM;SUV39H1;GATB;GATC;ADA2;HYKK;COX7A1;BLVRA;HACD1;HACD3;HACD2;PLA2G10;MTHFD2;MTHFD1;LOC477365;LOC100684996;NSD1;NSD3;NSD2;ATP6V1D;AASS(down);ADSL;COASY;LOC477562;PLOD3;LOC119874008;NUDT12;NUDT11;NUDT16;LOC100683076;PIP5K1C;PIP5K1B;PIP5K1A;LOC119870103;PLPP2;PNLIPRP1;PNLIPRP3;PNLIPRP2;RPE;GCDH;PHYKPL;MAT2A;ASPA(up);MAT2B;CYP21A2;NDUFS7;MGAT1;MGAT2;MGAT3;MGAT5;CHST10;COQ6;OTC;LOC102156642;ACADVL;NDUFB9;CA4(up);PCCB;NDUFB6;LOC480777;PCCA;NDUFB3;NDUFB2;NDUFB1;URAD;PDE7B(up);CRYL1;ACOT8;SHMT2;SHMT1;PLD4;ADCY5;ADCY6;ADCY7;PLD1;ADCY2;ADCY3;ADCY8;ADCY9;ETNPPL;LOC102152785;PNPO;PNLIP;FHIT(down);LOC119867230;ACSBG1;ACSBG2;GK2;GALT;LOC610725;LOC119869567;PLCB2;PLCB3;PLCB4;DDO;DDC;GPHN;SETMAR;ALDH4A1;ATP6V1G3;ATP6V1G2;ATP6V1G1;NADK;EARS2;NAMPT;INPP4A;ADCY10;LOC102153215;BHMT;AK5(up);LOC490770;DCXR;MTMR7(up);ND3;ND2;ND5;ND4;ND6;ACACA;PLA2G2E;PLA2G2D;PLA2G2F;LOC608048;LOC477556;LOC477558;HIBCH;HYI;MDH2;ADPGK;PDE6B;KYNU;LOC483833;SMOX;LOC119870484;CBS;FAH;PDE6A;SYNJ1;PHOSPHO2;SYNJ2;ANPEP;RENBP;PDE6D;THTPA;UPP1;BPNT1;BPNT2;CYP4A11(down);ECHDC1;LOC100856533;PHOSPHO1(up);SETD1A;LOC102151856;OAT;LOC607460;LOC100686787;AHCYL1;AHCYL2;PDE7A;PRXL2B;ENTPD6;ENTPD5;ENTPD4;ENTPD3;ENTPD1;FDPS;MTM1;ALOX5;ENTPD8;HYAL3;HYAL2;HYAL1;HYAL4;NFS1;LOC119864666;AKR1A1;AANAT;SGPP2(down);ST3GAL4;GUCY1A1;GUCY1A2;GBA2;GBA3;AMT;NDUFV3;SIRT1;GNPNAT1;NDUFV2;LOC111096466;IVD;HADH;GSTT2B;POMGNT1;TKFC;ME1;ME3;GAD1;LOC100684842;MTHFD2L;AFMID;ST6GALNAC6;ACP5(down);MTHFR;MTHFS;LOC100686148;LOC102153601;ST6GALNAC5;DEGS1;DEGS2;GDA;LALBA;RRM2;RRM1;DERA;PAPSS1;PAPSS2;GCLC;C1GALT1;TH;CYP2C18;TDO2;NAGS;MAN1C1;GGT6;GGT7;NAGA;NAGK;GLYAT;PGAP1;GPAT3;GPAT2;RIMKLB;NDUFAB1;SGPL1;NDUFB8;LOC486404;NDUFB7;KMT5C;ACSL5(up);NDUFB5;NDUFB4;APRT;COX6A2;SORD(up);L2HGDH;LOC100688796;HSD17B12;LOC102156116;HSD17B10;ATP6V1F;ATP6V1A;ENPP7;BBOX1;C1GALT1C1;UOX;ATP6V1H;UROS;PAH;UROD;PANK1;PANK3;PANK2;MGAT4A(up);MCCC2;MCCC1;RIMKLA;PAFAH2;GPX1;PDE6C;GPX3;GPX2;GPX5;PDE6G;GPX7;GPX6;GPX8;PDE6H;CYP2J2;LOC111095419;HGSNAT;FH;ABO(down);PKLR;NDUFV1;MVD;ALDH9A1;MVK;DCT;CMBL;SUCLA2;ATP6V1E1;LOC102156938;ATP6V1E2;COX5A;COX5B;FLAD1;SGMS2;SGMS1;HSD17B8;ACAA2;ACAA1;HSD17B1;HSD17B2;HSD17B3;HSD17B4;HSD17B6;HSD17B7;NOS1;ALDH3B1;NOS3;LOC609911;POMT1;PLA2G4B;HGD;ST6GALNAC4;ST6GALNAC3;PLA2G4F;ST6GALNAC1;PLA2G4D;ATP4A;ATP4B;LOC609048;TGDS;FOLH1B(up);GNPDA1;MAT1A;LOC100683222;NDUFA10;NDUFA11;NDUFA12;NDUFA13;LOC477441;XYLT1(up);EXT2;EXT1;XYLT2;MGST1(down);ADCY1;PLD2;PLD3;ALDH5A1;MMAB;TYR;ALG8;PDXK;ALG2;ALG3;ALG1;ALG6;ALG5;PDXP;ASMT;CEL;ITPKA;ITPKC;ITPKB;PDE5A;PKM;ITPK1;DLAT;GALNT8;GALNT9;GALNT6;GALNT7;GALNT1;GALNT2;GALNT3;TPH1;MAOB;MAOA;DTYMK;LOC100856518;PI4KB;PI4KA;LOC102154725;PGAM2;MARS2;PGAM1;PLA2G4A;FECH;AKR1C3;LOC102154372;FCSK;CHPT1;ASL;ATP6V0B;ATP6V0C;LOC102154615;LOC102152592;NAGLU;GMPPA;GMPPB;ACADSB;DUT;TRIT1;ALDH2;ADSS2;ADSS1;NEU4;NEU1;NEU2;NEU3;UQCR10;UQCR11;ASAH1;ASAH2;CYTB;PFKP;PFKM;PFKL;LOC487689;ACMSD;DHCR24;CYP1A1(up);AZIN2;GLDC(down);TM7SF2;PLB1(up);CERS6;ASPDH;CERS4;CERS5;CERS2;POC1B;CERS1;PRODH2;GNPDA2;GUSB;GPI;EXTL1;EXTL2;EXTL3;UPRT;GPT;MAN1A1;GMPR;GMPS;ATIC;DHODH;CYP24A1(up);BST1;PC;B4GALT1;B4GALT3;B4GALT2;PGK2;B4GALT4;B4GALT7;B4GALT6;ACSF3;MAN2A2;MAN2A1;LCT;GALK1;CYP19A1;LOC119866701;LCLAT1;EHMT1;EHMT2;HPRT1;CYP7A1;DPM3;DPM2;DPM1;NOS2(up);LOC100855552;AKR1B1;GALNT16;GALNT17;GALNT14;GALNT15;GALNT12;GALNT13;GALNT10;GALNT11;CDS1;CDS2;ASNS;GALNT18;PDE4A;PDE4B;PDE4C;PDE4D;LOC100855425;AOX2;AOX4;AACS;LOC100686889;LOC111096459;COX2;COX3;COX1;GAA;KMT2C;DGKZ;KMT2A;KMT2E;KMT2D;MTR;SUV39H2;ENPP3(down);SUCLG2;SUCLG1;ALDOA;ALDOC;ALDOB;QARS1;CYC1;PSAT1;LOC610164;HAO1;HAO2;LOC119872128;MSMO1;GSR;LOC611563;ADCY1\_1;DHCR7;LOC611366;UGP2;NDST1;NDST2;NDST3;NDST4;MPST;KHK;NNT;NME1;HK2;ALOX15B(up);ENPP4;HK1;ENPP1;DGKB;TREH;LDHA;DHDH;CPS1;HK3;H6PD;GLUL;PDHX;PDHB;ACOT4(up);GYS1;GYS2;STT3B;STT3A;ARG1;ARG2;HMOX1;LOC119863903;CRLS1;POMT2;GCSH;AGPS;ALAD;IDS;RBKS;ALG9;LOC100686500;FASN;IL4I1;LOC485024;PLCB1(up);GCAT;LOC100684768;PSPH;GLUD1;LOC606974;OXCT1;GALNTL5;NDUFS8;SCP2;SLC33A1;PDE9A;PDE2A;OPLAH;PRPS2;PRPS1;RPIA;LDHD;GMPR2;MCEE;MGAM2;LDHB;LDHC;GAPDHS;MTAP;LOC486100(down);GSTK1 |
| ABC transporters | TAP1;TAP2;LOC100683063;ABCA13;ABCC8(down);ABCA8;LOC478384;LOC479817;ABCB1;ABCB7;ABCB6;ABCB5;ABCB4;ABCD1;ABCD3;ABCD2;ABCD4;ABCA3;LOC489979;ABCA12;ABCB11;ABCB10;ABCC12;ABCB9;ABCC10;ABCC11;ABCG1;ABCG2;ABCB8;ABCG4;ABCG5;ABCG8;ABCC1;ABCA6;ABCA7;ABCA4;ABCA5;ABCA2;ABCC9;ABCA1;ABCC4;ABCC5;ABCC6;ABCA9;CFTR;ABCC2;ABCC3 |
| Phosphonate and phosphinate metabolism | PCYT1A;SELENOI;CHPT1;CEPT1;PCYT2 |
| Aldosterone-regulated sodium reabsorption | FXYD4;LOC100856339;FXYD2;INSR;LOC102152316;SCNN1A(down);PIK3CA;PIK3CB;PIK3CD;SCNN1G;MAPK3;MAPK1;PIK3R2;PIK3R1;HSD11B2;SCNN1B;IGF1;PRKCA;ATP1B4;PRKCB;ATP1B2;ATP1B3;PRKCG;ATP1B1;NR3C2(up);KRAS;KCNJ1;SGK1;IRS1;INS;SFN;NEDD4L;ATP1A4;ATP1A3;ATP1A1;SLC9A3R2;PDPK1 |
| Endocrine and other factor-regulated calcium reabsorption | PTH1R;VDR;FXYD2;DNM3;PTH;CLTA;CLTC;CLTB;SLC8A3(up);AP2B1;AP2M1;AP2S1;KL(up);RAB11A;SLC8A1;ATP1A3;SLC8A2;GNAS;PLCB2;PLCB3;PLCB4;PRKCA;ATP1B4;PRKCB;ATP1B2;ATP1B3;CALB1;ATP1B1;TRPV5;KLK2;AP2A1;PRKCG;AP2A2;ATP2B1;CLTCL1;ATP2B3;ATP2B2(up);ATP2B4;ADCY6;BDKRB2;GNAQ;PLCB1(up);ESR1;ADCY9;ATP1A4;PRKACA;ATP1A1;PRKACB;DNM1;KLK1 |
| Hippo signaling pathway - multiple species | RASSF1;RASSF2;RASSF4;SAV1;LATS2;LATS1;MOB1A;TEAD3;TEAD2;TEAD1;MOB1B;TEAD4;FAT4(up);WWC1;CSNK1E;NF2;LIMD1;AJUBA;FRMD1(down);DCHS1;DCHS2;STK3;FRMD6;RASSF6;YAP1;WWTR1;WTIP;PAK1 |
| mRNA surveillance pathway | LOC492024;NCBP1;NCBP2;PABPN1L;PPP2R3B;PPP2R3C;PPP2R3A;CPSF4L;NUDT21;LOC100687327;SMG5;SMG7;SMG6;SMG1;PCF11;SAP18;HBS1L;MAGOH;SYMPK;PPP2R2D;ACIN1;PPP2R2A;PPP2R2C;PABPC4L;PPP2CA;PPP2CB;CSTF2T;NCBP2L;PABPC1;PABPC4;PABPC5;SSU72;RNGTT;RNPS1;PABPC1L2A;GSPT1;NXF1;NXF3;ALYREF;PYM1;DDX39B;MAGOHB;WDR33;PPP2R5D;PPP2R5E;PPP2R5A;EIF4A3;PPP2R5C;UPF1;UPF2;RBM8A;PABPC1L;ETF1;NXT2;CLP1;NXT1;PPP2R1A;PPP2R1B;LOC100684102;WDR82;CPSF7;CPSF6;CPSF4;CPSF3;CPSF2;CPSF1;LOC485220;PELO;LOC608299;FIP1L1;RNMT;PABPN1;MSI2;MSI1;CASC3;PAPOLG;PAPOLA;PAPOLB;DAZAP1;LOC102156482;PPP1CB;PPP1CC;PPP1CA;SRRM1;CSTF1;CSTF3;UPF3B;UPF3A;PPP2R5B;PNN;PPP2R2B(up) |
| Non-homologous end-joining | DNTT;MRE11;PRKDC;LIG4;FEN1;XRCC4;RAD50;POLM;POLL;XRCC5;DCLRE1C;NHEJ1;XRCC6 |
| Biosynthesis of amino acids | OTC;GPT2;LOC611563;ACO1;GAPDH;GPT;LOC482320;CS;TPI1;PGAM2;NAGS;PGAM1;PYCR3;PYCR2;PYCR1;CPS1;LOC111096459;IDH3B;LOC102152592;IDH3A;PKLR;ACO2;PRPS2;IDH3G;PRPS1;TKTL1;TALDO1;TKT;GLUL;PFKP;MAT2B;PC;PFKM;PFKL;RPE;LOC111089999;LOC610338;ASNS;GOT2;RPIA;ALDH18A1;GOT1;PGK2;LOC119870407;PGK1;ASL;MAT2A;LOC100683724;ASS1;MTR;IDH2;IDH1;BCAT2;BCAT1;MAT1A;ENO1;ENO2;ENO3;ENO4;PHGDH;ALDOA;LOC608800;ALDOC;ALDOB;SHMT2;SHMT1;PKM;SDSL;CTH;SDS;LOC102154094;LOC477441;ARG2;PSPH;LOC612295;LOC119876526;PSAT1;CBS;LOC111094513;PAH;ARG1;LOC106558345;LOC476602;LOC102151775;LOC481849 |
| Primary bile acid biosynthesis | ACOX2;SCP2;CYP46A1;AKR1D1;CH25H(up);CYP27A1;AMACR;CYP7B1(up);HSD3B7;CYP39A1(up);ACOT8;CYP8B1;SLC27A5;CYP7A1;HSD17B4;BAAT |
| Pentose and glucuronate interconversions | SORD(up);KL(up);CRYL1;DCXR;LOC480777;LOC102154822;UGT2A3;UGDH;AKR1A1;RPE;UGT1A6(down);LOC102154742;DHDH;AKR1B1;XYLB;UGP2;GUSB |
| MAPK signaling pathway - plant | LOC102156116;LOC102153034(down);CAT;LOC102154109;ATP7A;ATP7B;CALM2;CALM3;CALM1;LOC100688420;NME4;LOC111091468;NME6;NME7;NME1;NME2;NME3;LOC102151128(down);CALML6;CALML5;CALML4;LOC102155863;LOC483943 |
| Notch signaling pathway | HDAC1;DTX4;HDAC2;MFNG;DTX1;DTX2;DTX3;KAT2B;KAT2A;NCSTN;CIR1;RFNG;ADAM17;PSENEN;NCOR2;APH1B;APH1A;MAML2;MAML1;PSEN2;PSEN1;LFNG(up);DVL2;DVL3;DVL1;JAG1;NUMBL;DTX3L;RBPJL;DLL1;DLL3;DLL4;NUMB;HES1;PTCRA;EP300;SNW1;NOTCH1;LOC119863905;CTBP1;CTBP2;RBPJ;CREBBP |
| Tropane, piperidine and pyridine alkaloid biosynthesis | TAT;GOT2;AOC3;AOC2;GOT1 |
| Oocyte meiosis | PPP3R2;PPP3R1;PLK1;ADCY1\_1;PKMYT1;CALM2;CALM3;CALM1;ANAPC11;ANAPC10;ANAPC13;ESPL1;LOC100855903;CUL1;IGF1R;SMC1B(up);ADCY5;CAMK2D;LOC102153034(down);CAMK2G;CAMK2A;CAMK2B;ADCY4(up);LOC479459;ADCY6;ADCY7;ADCY1;ADCY2;ADCY3;PPP2CA;SGO1;PPP2CB;ADCY8;ADCY9;LOC609669;RBX1;MAPK11;SMC1A;FBXW11;LOC100684434;CDC16;CCNE2;CCNE1;LOC119869567;IGF1;BUB1;CDC25C;STAG3;SPDYC;LOC100856295;LOC100685549;ITPR2;ITPR3;ITPR1;ANAPC5;ANAPC4;ANAPC7;RPS6KA1;RPS6KA2;RPS6KA6;MOS;CDC26;CDC23;CALML6;CALML5;CALML4;LOC476070;PPP2R5D;PPP2R5E;PPP2R5A;PPP2R5B;PPP2R5C;PLCZ1;LOC487020;MAPK14;ANAPC1;FBXO5;MAPK12;MAPK13;ANAPC2;SKP1;SPDYA;SMC3;MAPK3;MAPK1;CPEB4;PTTG1;FBXO43;INS;CDK1;CDK2;PRKACA;PRKACB;MAD2L2;PPP2R1A;PPP2R1B;LOC102156563;CPEB1;CPEB3;CPEB2;LOC608573;MAP2K1;CDC20;YWHAZ;MAD1L1;YWHAQ;CDC27;YWHAH;PGR;AURKA;YWHAB;YWHAG;YWHAE;LOC100682940;AR;SLK;LOC111090648;CCNB1;PPP1CB;PPP1CC;PPP1CA;BTRC;REC8;PPP3CA;PPP3CB;PPP3CC |
| Glucosinolate biosynthesis | BCAT2;BCAT1 |
| Cell cycle - Caulobacter | LONP2;CLPX;CLPP |
| Cell cycle - yeast | PPP2R1A;PPP2R1B;CKS1B;RAD17;NCAPG;SMC4;DYRK2;BUB1;DYRK4;RAD1;MOB1A;MOB1B;CDC6;CDC7;TTK;CDC16;LOC476070;SKP1;MAD1L1;SMC3;SMC2;ANAPC11;ANAPC10;NCAPD2;MAPK3;NIPBL;MAPK1;ESPL1;CKS2;CHEK2;CUL1;CHEK1;SMC1A;PPP2CB;SMC1B(up);STAG2;STAG1;RAD21;ORC6;PPP2R2D;ORC4;ORC5;ORC2;ORC3;PPP2R2C;ORC1;BUB3;MCM6;MCM5;MCM4;MCM3;MCM2;ANAPC1;ANAPC2;ANAPC5;ANAPC4;ANAPC7;CDC27;CDC26;PRMT5;FZR1;CDC23;CDC14B;CDC14A;CDC20;PPP2R2A;PPP2CA;NCAPH;CDC45;RBX1;LOC111090648;PPP2R2B(up) |
| Betalain biosynthesis | DDC;COMT;TYR;LOC106560171 |
| Retrograde endocannabinoid signaling | PTGS2;LOC100682956;NDUFB9;NDUFB8;NDUFB7;NDUFB6;NDUFB5;ADCY1\_1;NDUFB3;NDUFB2;NDUFB1;LOC100688835;NDUFAB1;NDUFA4L2(down);GNG14;CACNA1A;CACNA1B;CACNA1C;CACNA1D;GNG11;GNG12;GNG13;LOC119863873;LOC119872525;NDUFA10;NDUFA11;NDUFA12;GABRR1;CACNA1S;GABRR2;NDUFB4;NDUFB11;ADCY4(up);FAAH;ABHD6;ADCY5;ADCY6;ADCY7;ADCY1;ADCY2;ADCY3;NDUFB10;ADCY8;ADCY9;GNG7;GNG4;GNG5;GNG2;GNG3;LOC102151506;SLC17A6;GNG8;MAPK11;GABRG3;GABRG2;GABRG1;GRIA1;GRIA4;GABRA1;LOC100855681;LOC100855914;GRM5;GRM1;LOC119864666;LOC119869567;NDUFV3;PLCB2;PLCB3;PLCB4;GNAI1(up);ITPR2;ITPR3;LOC111096466;ITPR1;GNAQ;GABRQ;GABRP;GABRE;GABRD;LOC102154025;GNB4(up);SLC32A1;GABRR3;NAPEPLD;MAPK14;LOC475367;MAPK10;CACNA1F;MAPK12;MAPK13;GABRA5;GABRA4;GABRA6;GNAI2;GNAI3;GABRA3;GABRA2;CNR1;NDUFV2;NDUFV1;MAPK3;NDUFS5;GRIA2;MAPK1;GRIA3;GNAO1;LOC119870103;MAPK8;MAPK9;ND5;RIMS1;ND1;DAGLA;NDUFA7;NDUFA4;DAGLB;NDUFA2;NDUFA3;NDUFA1;GNB5;NDUFA13;GNB1;GNB3;GNB2;PLCB1(up);GABRB1;GABRB2;GABRB3;LOC100684768;ND3;KCNJ3;ND2;SLC17A8;KCNJ6;KCNJ5;KCNJ9;SLC17A7;ND4;ND4L;PRKACA;PRKACB;ND6;LOC102154590;NDUFA6;MGLL;LOC119874008;LOC106557778;NDUFA5;LOC100686889;LOC100688724;GNG10;NDUFC1;NDUFC2;NDUFS6;PRKCA;PRKCB;PRKCG;NDUFA8;NDUFA9;NDUFS1;NDUFS2;NDUFS3;NDUFS4;GNGT2;GNGT1;NDUFS7;NDUFS8 |
| Aldosterone synthesis and secretion | ADCY1\_1;MC2R;NR4A1(up);CACNA1H;CACNA1I;CALM2;CALM3;CALM1;CAMK1;CACNA1C;CACNA1D;CAMK4;CACNA1F;CACNA1G;AGT;CACNA1S;NPR1;LIPE;CAMK2D;ATP1B4;CAMK2G;CREB3;CAMK2A;CAMK2B;ATP1B1;ADCY4(up);ATP2B1;ATP2B3;ATP2B4;ADCY5;ADCY6;ADCY7;ADCY1;ADCY2;ADCY3;SCARB1;ADCY8;ADCY9;HSD3B2;STAR;ATP1B2;PLCB3;CYP11A1;PRKACA;NPPA;LOC119869567;PLCB2;ORAI1;PLCB4;ITPR2;ITPR3;ITPR1;GNAQ;GNAS;CALML6;CALML5;CALML4;AGTR1;LOC102153034(down);ATP2B2(up);DAGLA;DAGLB;PLCB1(up);LDLR;POMC;KCNJ5;ATP1A4;ATP1A3;ATP1A1;PRKACB;PRKD1;PRKD2;PRKD3;CAMK1D;CAMK1G;NR4A2(up);KCNK9;KCNK3;CREB5;PRKCA;CYP21A2;PRKCB;PRKCE;PRKCG;ATP1B3;CREB1;PDE2A;CREB3L2;CREB3L3;CREB3L1;CREB3L4;GNA11;ATF4;ATF6B;ATF1;ATF2 |
| TGF-beta signaling pathway | PPP2R1A;PPP2R1B;TGFB3;ACVR1;SMAD9;ACVR1C;ACVR1B;SMAD4;NODAL;SMAD6;SMAD7;TGFBR1;SMAD2;SMAD3;LOC607768;CDKN2B\_1(down);BMPR1B;FST;NOG;PITX2;RPS6KB2;ZFYVE16;AMH;LOC490387;MAPK3;ROCK1;SKP1;NBL1;AMHR2;RPS6KB1;GDF5;THBS1;INHBA;MAPK1;LOC102151783;SMAD5;MYC;CUL1;TGFBR2;TGFB1;ACVR2A;ACVR2B;RBL1;LTBP1;BMP6;IFNG;SP1;TGIF1;BMP5;LOC100855618;SMAD1;BAMBI(up);SMURF2;LOC102155562;RHOA;EP300;SMURF1;TGFB2;BMP7;BMP4;E2F5;E2F4;NEO1;ZFYVE9;PPP2CA;ID4;PPP2CB;ID2;ID3;ID1;BMPR1A;TNF;BMPR2;CREBBP;DCN;RBX1;LOC111090648;GDF7;TGIF2;TFDP1;CHRD |
| Hepatocellular carcinoma | LOC100856339;LOC479911;ACTG1;LOC479912;GSTA4;NQO1;AKT1;AKT2;AKT3;ARAF;GADD45A;LOC612475;LOC119867218;WNT8A;PIK3CA;PIK3CB;PIK3CD;LOC486404;GADD45G;MYC;SHC4;IGF1R;SHC1;SHC2;SHC3;LOC611366;PLCG2;MGST1(down);KRAS;CCND1;TCF7L1;TCF7L2;GSK3B;PTEN;GRB2;LOC481841;APC;ACTB;WNT7A;WNT7B;HRAS;GAB1;MGST2;MGST3;FZD10;BAK1;TGFA;BCL2L1(down);NFE2L2;KEAP1;POLK;LOC106557930;ELK1;PIK3R2;PIK3R1;TCF7;LOC119870217;LOC119870216;LOC119870215;GADD45B;GSTO2;GSTO1;TP53;CSNK1A1;HMOX1;FRAT1;LOC119863906;LOC100856518;WNT10A;WNT5B;WNT5A;WNT10B;WNT3A;TGFB1;SMAD4;CDKN2B;SMAD2;SMAD3;HGF;RAF1;FZD1;FZD2;FZD3;FZD4;FZD6;FZD7;FZD8;FZD9;RPS6KB1;RPS6KB2;MAPK3;MAPK1;DDB2;CDKN1A;TGFBR2;TGFBR1;MTOR;GSTT2B;LEF1;LRP6;SOS2;LRP5;WNT3;WNT2;WNT1;WNT6;BAD;WNT4;MET;CDK4;LOC477556;CDK6;LOC610304;LOC477558;PLCG1;TGFB2;TGFB3;EGFR;BAX;CTNNB1;MAP2K2;MAP2K1;AXIN2;AXIN1;LOC476006;NRAS;RB1;DVL2;DVL3;WNT8B;DVL1;BRAF;WNT2B;PRKCA;PRKCB;PRKCG;APC2;E2F3;E2F2;E2F1;LOC474938;GSTP1;WNT9B;WNT9A;WNT16;WNT11 |
| Tyrosine metabolism | AOC3;AOC2;AOX4;ALDH3A1;AOX2;MIF;LOC100856533;ALDH1A3;TAT;FAHD1;FAH;TH;GOT2;IL4I1;GOT1;LOC106560171;TYRP1;ALDH3B1;TPO;LOC482436;COMT;LOC119867230;DDC;HGD;DCT;HPD;DBH;ADH5;ADH4;GSTZ1;MAOB;MAOA;PNMT;TYR |
| Regulation of actin cytoskeleton | MYL7(up);LOC100856339;ACTG1;CRKL;MYH11;LOC102152706;BRK1;MYL5;MYL2;INSRR;LOC488190;MYL9;MYH15;LOC102154266;ACTB;ARHGEF6;ARHGEF7;ARHGEF4;FGF3;PIK3CD;GNG12;ARHGEF1;LOC102153128;LOC111094520;PIP4K2A;PIP4K2C;PIP4K2B;LOC607207;ARHGEF12;LPAR5;DIAPH1;DIAPH2;DIAPH3;SSH2;SSH3;SSH1;FGFR4;CXCL12;FGFR2;FGFR3;KRAS;LPAR4;FN1;ARPC1B;ARPC1A;MYLK;F2R;RRAS2;PAK4;APC;PAK6;PAK1;PAK2;ITGA8;ITGA9;ARPC5L;ITGA1;ENAH;DOCK1;ITGA4;ITGA5;ITGA6;ITGA7;RRAS;ITGA11;CRK;PDGFD;LOC100687971;LOC100683370;ITGB4;SLC9A1;FGF10;ITGB6;EGF;TIAM1;F2(down);PIK3R1;PIK3R2;ITGAL;GSN;NCKAP1;PAK3(up);SPATA13;ITGAM;PIKFYVE;ARPC3;ARPC2;MYL10;MRAS;ARPC5;ARPC4;HRAS;PAK5;FGF7;FGF6;RHOA;FGF4;BCAR1;FGF2;FGF1;ITGAX;RAC2(up);MYH4;VAV1;VAV3;VAV2;ITGAV;INS;PFN1;PFN2;PFN3;PFN4;GIT1;PIK3CA;BAIAP2;GNA13;ITGAD;PIK3CB;FGF20;MYH10;PDGFRB;PDGFRA;MYH13;MYH14;FGF9;MYH16;FGF8;LOC102156038;PTK2;SOS2;RAF1;FGF5;MYH2;MYH3;ITGB8(down);MYH1;ITGAX\_1;MAPK3;ITGA2B;MYH8;MYH9;ARAF;LIMK2;MAPK1;PPP1R12A;PPP1R12B;PPP1R12C;FGF11;BRAF;FGF17;ITGA2;SRC;ITGA3;FGD1;FGD3;GNG14;EGFR;FGF18;MYLK2;MYLK3;LOC106558389;MYH7B;LOC119866928;BDKRB1;BDKRB2;ITGB2(down);MYL12B;MAP2K2;PIP5K1C;PIP5K1B;PIP5K1A;ACTN4;MAP2K1;FGF16;PDGFB;MOS;LPAR2;FGFR1;CDC42;LOC102156243;NCKAP1L;CYFIP1;CYFIP2;TMSB4X;ROCK2;LIMK1;CHRM3;LOC102152609;MSN;LOC119866177;MYLPF;IQGAP1;IQGAP2;IQGAP3;ITGA10(up);LPAR1(up);WASF1;WASF2;NRAS;LOC102157022;PXN;LOC100686562;ARHGAP35;ITGB1;PDGFA;ITGB3;PDGFC;ITGB5;RAC3;ITGB7;RAC1;CHRM5;CHRM4;VCL;CHRM2;CHRM1;RDX;CFL2;CFL1;APC2;ACTN1;PPP1CB;PPP1CC;PPP1CA;ABI2;FGF22;KNG1;LOC119863891(up);ROCK1;EZR;ITGAE;GNA12;LOC111092429;SCIN |
| RNA degradation | PABPC5;LOC102154724;EDC4;DCP2;TENT4A;PARN;PNPT1;TENT4B;PABPC4;LOC102154080;PFKP;EDC3;EXOSC8;EXOSC9;LOC479071;DCP1B;DCP1A;EXOSC2;EXOSC3;EXOSC1;EXOSC6;EXOSC7;EXOSC4;EXOSC5;EXOSC10;PAN2;BTG4;BTG3;BTG2;LOC111093384;HSPD1;PAN3;PFKM;MPHOSPH6;LSM4;CNOT9;SKIV2L;DHX36;PABPC1;CNOT3;CNOT2;CNOT1;WDR61;ZCCHC7;CNOT6;CNOT4;DCPS;LSM7;LSM8;PABPC1L2A;LOC119865916;LSM5;LSM6;CNOT10;CNOT8;LSM2;LSM1;ENO1;ENO2;ENO3;ENO4;PABPC4L;LOC608800;LSM3;PFKL;CNOT6L;DIS3;PNLDC1;BTG1;CNOT7;DIS3L;TOB2;C1D;MTREX;DDX6;HSPA9;TTC37;XRN1;XRN2;NUDT16;PABPC1L;PATL1;LOC100687895 |
| Styrene degradation | FAH;GSTZ1;HGD |
| Carbohydrate digestion and absorption | LOC100856339;FXYD2;PIK3CD;G6PC1;G6PC3;G6PC2;SLC5A1;AKT2;AKT3;LOC607460;LOC119870215;GNAT3;SLC37A4;HKDC1;PIK3CA;MGAM;CACNA1D;PRKCB;LOC119870217;PIK3R2;PIK3R1;LOC119870216;MGAM2;PLCB2;PLCB3;PLCB4;HK2;HK3;ATP1B4;HK1;ATP1B2;ATP1B3;ATP1B1;PLCB1(up);LOC489640;LCT;AKT1;SLC2A2;SI;ATP1A4;ATP1A3;ATP1A1;TAS1R3;TAS1R2;PIK3CB |
| Propanoate metabolism | ACADS;PCCB;PCCA;LDHAL6B;ABAT;LOC100686034;HADHA;ACAT1;ACAT2;MCEE;LDHA;LDHB;LDHC;ACACA;DLD;ACSS3;ACSS2;ACSS1;LOC609911;LOC102153001;ACACB(down);MMUT;ALDH6A1;SUCLG1;LOC490690;LOC100684709;EHHADH;MLYCD;SUCLG2;ACOX3;SUCLA2;ACOX1;DBT;ECHS1;BCKDHB;BCKDHA;HIBCH;ECHDC1 |
| Valine, leucine and isoleucine biosynthesis | SDSL;BCAT2;BCAT1;SDS |
| C-type lectin receptor signaling pathway | PTGS2;LOC100856339;PPP3R2;PPP3R1;PLK3;IKBKB;AKT1;AKT2;AKT3;IKBKE;CALM2;CALM3;CALM1;PIK3CA;PIK3CB;PIK3CD;RELB;RELA;ARHGEF12;MDM2;KRAS;SYK;PTPN11;CYLD;PAK1;NFATC1;NFATC2;NFATC3;NFATC4;HRAS;RRAS2;IL12A(up);RRAS;CD209;MAPKAPK2;JUN;CARD9;PIK3R2;PIK3R1;LOC100683481;KSR1;LOC119870217;LOC119870216;LOC119870215;MRAS;CHUK;ITPR2;ITPR3;RHOA;ITPR1;IRF1;STAT1;IRF9;CALML6;CALML5;CALML4;LOC102153034(down);MAPK14;MAPK10;MAPK11;MAPK12;MAPK13;IL1B;RAF1;FCER1G;STAT2;NLRP3;MAPK3;MAPK1;NFKB1;NFKB2;MAPK9;IL17D;SRC;NFKBIA;MAPK8;IL10;IL6;IL2;IL23A;IL12B;PLCG2;CLEC7A(down);NRAS;PYCARD;MALT1;PRKCD;TNF;BCL10;CASP8;MAP3K14;PPP3CA;PPP3CB;PPP3CC;BCL3 |
| Mannose type O-glycan biosynthesis | ST3GAL3;CHST10;POMT2;LOC476397;POMT1;FUT4;MGAT5B;POMGNT1;B4GALT1;B3GAT2;B4GALT3;B4GALT2;B3GAT1;FUT9 |
| Other types of O-glycan biosynthesis | COLGALT2;GALNTL5;COLGALT1;ST6GAL2;EOGT;ST6GAL1;POGLUT1;MFNG;POFUT2;POFUT1;POC1B;PLOD3;LFNG(up);C1GALT1;GXYLT2(up);B4GALT1;B4GALT3;B4GALT2;B3GLCT;ST3GAL3;GALNTL6;GALNT5(up);GALNT8;GXYLT1;RFNG;GALNT6;GALNT7;GALNT1;GALNT2;GALNT3;GALNT16;GALNT17;GALNT14;GALNT15;GALNT12;GALNT13;GALNT10;GALNT11;OGT;C1GALT1C1;GALNT18;LOC476397;POMT1;POMT2;LOC102152620;GALNT9 |
| Various types of N-glycan biosynthesis | B4GALNT4;RPN1;RPN2;ALG9;TUSC3;ALG2;ALG3;ALG1;ALG11;DAD1;ALG12;B4GALNT3;MGAT4A(up);B4GALT1;B4GALT3;B4GALT2;ST3GAL3;LOC480667;MAN2A2;MAN2A1;CHST9;MGAT1;MGAT2;MAN1C1;STT3B;STT3A;ALG13;ALG14;MAN1A1;MAN1A2;CHST8;HEXA;LOC476397;HEXB;HEXD;MGAT4D;MGAT4C;MGAT4B;FUT8 |
| Mucin type O-glycan biosynthesis | GCNT4;B3GNT6;GALNT7;GCNT1;GCNT3;C1GALT1;GALNT2;B4GALT5;GALNTL5;ST3GAL2;ST3GAL1;GALNTL6;GALNT5(up);GALNT8;GALNT9;GALNT6;POC1B;GALNT1;ST6GALNAC1;GALNT3;GALNT16;GALNT17;GALNT14;GALNT15;GALNT12;GALNT13;GALNT10;GALNT11;C1GALT1C1;GALNT18;LOC102152620 |
| Other glycan degradation | MAN2B2;MANBA;MAN2B1;HEXA;AGA;HEXB;HEXD;GLB1;FUCA1;FUCA2;NEU4;MAN2C1;GBA2;ENGASE;GBA;NEU1;NEU2;NEU3 |
| N-Glycan biosynthesis | DOLK;RPN1;GANAB;ALG9;ST6GAL2;ST6GAL1;ALG2;ALG3;ALG1;ALG6;ALG8;ALG5;DOLPP1;RPN2;STT3B;DAD1;STT3A;TUSC3;MGAT5B;B4GALT1;MGAT4A(up);B4GALT3;B4GALT2;DPM3;DPM2;DPM1;LOC477647;DPAGT1;LOC480667;MAN2A2;MAN2A1;SRD5A3;MGAT1;MGAT2;MGAT3;MGAT5;ALG11;ALG12;ALG13;ALG14;MAN1A1;MAN1A2;LOC476397;MOGS;MGAT4D;MAN1C1;MGAT4C;MGAT4B;FUT8 |
| Dorso-ventral axis formation | PIWIL4;PIWIL2;PIWIL1;CPEB4;CPEB1;CPEB3;FMN2(up);MAP2K1;SPIRE2;MAPK3;ETS1;EGFR;LOC486307;CPEB2;KRAS;SOS2;ETV7;ETV6;SPIRE1;NOTCH1;LOC111094784;GRB2;BRAF;MAPK1 |
| GnRH secretion | LOC102151131;LOC100856339;KCNJ6;NRAS;HRAS;TRPC5;GPER1;AKT1;AKT2;AKT3;ARRB2;TRPC4;ARRB1;GABBR1;RAF1;CACNA1H;CACNA1I;MAPK3;CGA;PIK3CA;PIK3CB;CACNA1C;PIK3CD;KCNJ11(down);CACNA1F;CACNA1G;LOC119870217;MAPK1;PIK3R2;PIK3R1;CACNA1S;GNRH1;KISS1R;PLCB2;PLCB3;PLCB4;PRKCA;LOC119870216;LOC119870215;PRKCB;PRKCG;CACNA1D;PLCB1(up);KCNN4;KCNN3;KCNN2;KCNN1;ITPR2;ITPR3;ITPR1;KRAS;KCNJ3;ESR2;GNAQ;KCNJ5;MAP2K2;KCNJ9;HCN2;MAP2K1;TRPC1;SPP1;GNA11;GABBR2(up) |
| alpha-Linolenic acid metabolism | PLB1(up);PLA2G1B;PLA2G12B;PLA2G12A;PLA2G6;ACAA1;PLA2G5;PLA2G3;PLAAT3(down);PLA2G4E(up);PLA2G2E;PLA2G2D;PLA2G2F;PLA2G4B;PLA2G4A;FADS2;PLA2G4F;PLA2G4D;PLA2G2C(down);PLA2G10;ACOX3;ACOX1 |
| Linoleic acid metabolism | CYP2J2;PLB1(up);ALOX15;PLA2G1B;PLA2G12B;PLA2G12A;PLA2G6;PLA2G5;PLA2G3;LOC489851;PLAAT3(down);PLA2G4E(up);PLA2G2E;PLA2G2D;PLA2G2F;PLA2G4B;PLA2G4A;PLA2G4F;PLA2G4D;PLA2G2C(down);PLA2G10;CYP2E1;CYP1A2(up);CYP2C18;LOC100688697 |
| Arachidonic acid metabolism | CYP2J2;PTGS2;EPHX2;PLB1(up);GGT5;PTGS1;ALOX15;ALOX12;PLA2G1B;LTA4H;PTGDS;PTGES;CYP2U1;AKR1C3;PTGIS;PLA2G12B;PLA2G12A;CYP2B6;CBR3;LOC484867;HPGDS;PLA2G6;LTC4S;PLA2G5;PRXL2B;PLA2G3;ALOX12B;TBXAS1;ALOX15B(up);ALOX5;LOC100688697;PLAAT3(down);PLA2G4E(up);LOC100688446;GGT1;PLA2G2E;PLA2G2D;PLA2G2F;PLA2G4B;PLA2G4A;PLA2G4F;PLA2G4D;PLA2G2C(down);PLA2G10;LOC610164;PTGES3;PTGES2;LOC607806;CYP4A11(down);CYP2E1;LOC119863926;GPX1;GPX3;GPX2;GPX5;CYP2C18;GPX7;GPX6;GPX8;CYP4A38(down) |
| Apoptosis | LOC100856339;PMAIP1;ACTG1;CASP10;AKT1;AKT2;AKT3;PIK3CA;GADD45B;PIK3CD;NTRK1;MAP3K5;LOC477570;CAPN2;CAPN1;FADD;RELA;KRAS;CTSH;DDIT3;CTSO;CTSB;CTSC;CTSD;DFFA;DFFB;CTSZ;FASLG;PRF1;HRK;CTSS;CTSV;CTSW;IKBKB;PTPN13;BAK1;ACTB;CSF2RB;NGF;TNFSF10;SPTAN1;HRAS;NFKBIA;PIDD1;DAXX;BCL2L1(down);TUBA1C;JUN;LOC119870217;PIK3R2;PIK3R1;LOC100855928;IL3;FOS;PARP4;LOC119870216;LOC119870215;PARP1;PARP2;CHUK;ITPR2;ITPR3;PARP3;LMNB2;LMNB1;TP53;LOC610636;GZMB;TNFRSF1A;GADD45A;AIFM1;PIK3CB;TUBA8;ATM;SPTA1;XIAP;MAPK10;DIABLO;GADD45G;RAF1;HTRA2;ITPR1;MAPK3;TRADD;CFLAR;MAPK1;NFKB1;MAPK8;MAPK9;APAF1;CTSK(up);DAB2IP;PHF19;TUBAL3;IL3RA;BAX;EIF2AK3;BCL2A1;MAP2K1;SEPTIN4;ERN1;BIRC5;BIRC3;BIRC2;CYCS;RIPK1;MAP2K2;BAD;MCL1;BBC3;EIF2S1;LOC106557476;BCL2L11;FAS;BID;NRAS;LOC608051;TRAF2;ENDOG;LOC100689004;TNF;CTSF;PDPK1;TUBA4A;LMNA;CASP6;CASP7;CASP2;CASP3;CASP8;CASP9;MAP3K14;ATF4;LOC119866377;BCL2 |
| Thyroid hormone signaling pathway | LOC100856339;FXYD2;ACTG1;AKT1;AKT2;AKT3;MDM2;EP300;PIK3CA;PIK3CB;PIK3CD;PFKM;THRA;PFKL;MYC;DIO1;DIO2;ATP1B4;ATP1B2;ATP1B3;MED27;ATP1B1;LOC608800;GATA4;KRAS;BMP4;CCND1;SLC2A1;GSK3B;ACTB;ATP2A1;ATP2A2;HRAS;KAT2B;KAT2A;MED1;MED4;SLC9A1;TSC2;MED12L;PIK3R2;PIK3R1;DIO3;FOXO1;PRKACA;MED30;PLCB2;PLCB3;PLCB4;LOC119870217;LOC119870216;LOC119870215;HIF1A;TP53;ESR1;MED13L;ITGAV;CREBBP;HDAC1;HDAC3;HDAC2;ATP2A3(down);RCAN1;PLCZ1;PLCE1;RAF1;NCOR1;NCOA2;NCOA3;NCOA1;MYH7;STAT1;TBC1D4;MAPK3;MAPK1;SRC;RXRG;RXRA;RXRB;MED24;PLCB1(up);PLCD4;MTOR;PLCD3;PLCD1;RCAN2(up);LOC611719;MAP2K2;PLCG1;BAD;WNT4;ATP1A4;ATP1A3;ATP1A1;PRKACB;PLN;RHEB;PFKFB2;CTNNB1;PLCG2;MAP2K1;NRAS;PFKP;MED12;MED13;MED14;SLC16A10;MED16;MED17;SIN3A;ITGB3;PRKCA;PRKCB;PRKCG;THRB(up);SLCO1C1;SLC16A2;NOTCH1;CASP9;PDPK1 |
| Oxidative phosphorylation | LOC100683828;LOC100682956;NDUFB9;NDUFB8;NDUFB7;NDUFB6;NDUFB5;NDUFB4;NDUFB3;NDUFB2;NDUFB1;UQCRQ;LOC102151856;LOC119866697;LOC100685227;UQCRH;LOC100688835;LOC100683222;NDUFAB1;LOC100686787;NDUFA4L2(down);COX10;NDUFB11;NDUFB10;ATP5J2;LOC119872525;LOC609990;NDUFA11;COX8A;NDUFA13;ATP6V0D1;SDHC;LOC102155410;LOC612644;ATP6;ATP6V1H;LOC100685720;ATP6V1B2;COX6A2;LOC102154615;LHPP;LOC119870901;LOC102152785;ATP12A;LOC119864666;UQCRFS1;LOC102151506;UQCRC2;UQCRC1;LOC100685007;LOC476372;ATP6V1F;ATP6V1A;LOC102156968;LOC487689;LOC111092022;COX7B2;LOC477508;LOC481939;LOC100684983;LOC610725;LOC102154486;COX7A1;LOC111096466;ATP5PO;ATP5F1D;ATP5F1E;ATP5F1A;ATP5F1B;ATP5F1C;LOC119863903;ATP6V1G3;LOC100684996;ATP6V1G1;LOC119868043;COX11;COX15;COX17;LOC481394;LOC102156885;LOC119866701;LOC102154025;ATP6V0A1;PPA2;PPA1;LOC102153059;LOC100684842;NDUFV3;NDUFV2;NDUFV1;LOC100855914;NDUFA12;TCIRG1;LOC100686500;ND1;NDUFA10;ND3;ND2;ND5;ND4;ND6;NDUFA6;NDUFA7;NDUFA4;NDUFA5;NDUFA2;NDUFA3;NDUFA1;LOC100688796;NDUFA8;NDUFA9;COX4I2;COX4I1;SDHA;LOC100683076;LOC100684768;ATP6V1G2;LOC100682717;LOC608048;ATP6V1D;LOC100686510;ATP6V1E1;ATP6V1E2;ND4L;LOC119870103;LOC111090910;LOC119870343;LOC102154590;LOC100855425;ATP6V0B;LOC102151754;COX5A;COX5B;ATP6V0C;UQCR10;UQCR11;LOC119874008;LOC106557778;ATP6V0D2(up);ATP8;ATP5MC1;LOC100686889;ATP5MC2;LOC119865581;ATP5MC3;LOC100688724;ATP6V0A4;LOC119870484;COX2;COX3;CYTB;COX1;ATP5MG;ATP6V1B1;ATP5ME;LOC102156642;NDUFC1;NDUFC2;ATP6V0A2;LOC102152234;ATP6V0E1;LOC102154372;ATP6V0E2;COX7A2L;ATP4A;ATP4B;LOC100686830;CYC1;NDUFS1;NDUFS2;NDUFS3;NDUFS4;NDUFS5;NDUFS6;NDUFS7;NDUFS8;ATP6V1C2;ATP6V1C1;SDHB;SDHD |
| Nicotinate and nicotinamide metabolism | NADK;AOX2;AOX4;NT5E;NADSYN1;NMRK1;CD38(up);ASPDH;NT5M;NT5C;NT5C3A;NT5C3B;NAMPT;NT5C1B;NT5C1A;NMNAT2;NMNAT3;NADK2;NT5C2;SIRT1;QPRT;ENPP3(down);ENPP1;NUDT12;NNT;NMNAT1;PNP;LOC489397;NAPRT;NNMT;BST1 |
| Selenocompound metabolism | PAPSS1;SCLY;MTR;SEPHS2;SEPHS1;MARS1;MARS2;LOC100684412;PAPSS2;SEPSECS;PSTK;KYAT1;KYAT3;CTH |
| Synaptic vesicle cycle | STX1A;ATP6V1D;SLC6A1;SLC17A7;ATP6V1A;CPLX4;LOC111090910;SLC32A1;ATP6V1H;DNM3;STXBP1;ATP6V0D2(up);ATP6V1C2;CLTA;CLTC;CLTB;UNC13B;SLC1A7;SLC1A3(up);AP2B1;SLC1A6;TCIRG1;AP2S1;LOC100686787;STX1B(down);SLC6A3;CACNA1A;CACNA1B;SLC6A4;NSF;AP2M1;LOC481939;NAPA;ATP6V1B1;ATP6V1B2;RIMS1;ATP6V0A1;CPLX2(up);ATP6V0C;LOC119870343;ATP6V0E1;ATP6V1G3;ATP6V0E2;ATP6V0A2;CPLX3;CPLX1;AP2A1;UNC13C;RAB3A;AP2A2;CLTCL1;ATP6V1C1;ATP6V0B;SNAP25;VAMP2;SLC17A8;ATP6V1F;SLC1A1(up);LOC102152234;STX3;STX2;ATP6V1E1;ATP6V1E2;ATP6V0A4;ATP6V0D1;UNC13A(down);ATP6V1G2;ATP6V1G1;SYT1(up);SLC18A3;DNM1;SLC18A1;SLC18A2;SLC17A6;SLC1A2 |
| Protein digestion and absorption | COL10A1(up);KCNE3;KCNK5(down);CPA1;FXYD2;COL11A1;SLC7A8;CTRL;COL21A1;SLC7A7;LOC119870307;PRSS2;COL12A1;COL9A1;COL3A1;SLC8A3(up);COL18A1(down);COL2A1;SLC9A3;COL14A1;COL11A2;PGA;CPA2;CPA3;SLC7A9;COL5A1;SLC6A19;COL13A1;COL1A2(up);SLC16A10;SLC8A1;ATP1A3;SLC8A2;DPP4;LOC475521;KCNJ13;COL27A1(down);ELN;COL6A1;COL4A4;SLC38A2;COL9A2;ATP1B4;ATP1B2;ATP1B3;KCNQ1;ATP1B1;COL5A2;COL4A6;COL4A5;KCNN4;COL4A3;COL4A2;COL4A1;ACE2;COL24A1;SLC15A1;COL17A1;LOC483802;COL15A1(up);COL22A1;COL6A2;COL7A1;COL5A3;SLC3A2;LOC610614;SLC3A1;COL9A3;CPB2;CPB1;SLC1A1(up);ATP1A4;LOC478196;XPNPEP2;ATP1A1;COL1A1;MME;SLC1A5;COL6A3;PRCP;COL6A5;MEP1A;MEP1B;COL6A6 |
| Cholesterol metabolism | SOAT2;SOAT1;STAR;LCAT;VAPA;VDAC3;APOC3;APOA4;VDAC2;VDAC1;APOA1;APOA2;LRP2(up);NCEH1;APOB;NPC2;NPC1;CYP7A1;LIPA;LDLR;LPL;CD36;TSPO;LOC119876012;ABCG5;ABCG8;SCARB1;ABCB11;CYP27A1;ANGPTL4;PLTP;ABCA1;SORT1;LDLRAP1 |
| RNA polymerase | POLR3F;POLR3G;POLR3D;POLR1C;POLR1D;POLR3C;POLR1F;POLR3A;POLR3K;POLR3H;POLR3GL;POLR2E;POLR2D;POLR2G;POLR2F;POLR2A;POLR2C;POLR2B;POLR2L;POLR2I;POLR2H;POLR2K;POLR2J;LOC119870926;POLR1A;POLR1B;POLR3E;POLR3B;POLR1E |
| Degradation of aromatic compounds | AKR1A1;ADH5;RGN |
| Galactose metabolism | LALBA;G6PC1;G6PC3;G6PC2;GALM;UGP2;HKDC1;MGAM;PFKP;PFKM;PFKL;B4GALT1;B4GALT2;MGAM2;GAA;GALT;HK2;HK3;GCK;HK1;PGM1;PGM2;GALE;LOC608800;AKR1B1;LCT;GALK1;GANC;GLB1;SI |
| Ascorbate and aldarate metabolism | LOC102154822;UGDH;LOC480777;LOC486100(down);UGT2A3;ALDH2;KL(up);MIOX;AKR1A1;UGT1A6(down);LOC102154742;ALDH7A1;ALDH1B1;GUSB;RGN;ALDH9A1 |
| Ras signaling pathway | LOC100856339;NF1;IKBKB;AKT1;AKT2;AKT3;RASAL1;BDNF;RASAL2;EGF;LOC111090226;CALM2;CALM3;CALM1;GNG14;PIK3CA;PIK3CB;LOC119869607;PIK3CD;NTRK1;NTRK2;GNG13;PLA2G6;PLA2G5;PLA2G3;RELA;FGFR1;LOC102156003;SHC4;IGF1R;RAB5A;SHC1;SHC2;SHC3;PLA2G4E(up);RAP1B;HTR7;GNG5;FASLG;KITLG;FGFR2;FGFR3;KRAS;PLA2G2C(down);PLD1;PLD2;RAPGEF5;PTPN11;CSF1;PLCG1;KIT;GNG7;GNG4;GRB2;GNG2;GNG3;LAT;MAPK10;PAK6;LOC102156776;GNG8;RASGRP2;RASGRP3;NGF;RASGRP1;RASGRP4;RASSF1;HRAS;RRAS2;GAB2;GAB1;RRAS;RASA4B;PLA2G1B;RASAL3;TGFA;BCL2L1(down);PLA2G12B;PLA2G12A;LOC102156901;TIAM1;ELK1;PIK3R2;PIK3R1;LOC100683481;ANGPT2;KSR1;KSR2;ANGPT1;ANGPT4;PAK3(up);LOC119872979;IGF1;LOC119870217;LOC119870216;LOC119870215;MRAS;PLA1A;CHUK;VEGFD;GRIN1;VEGFA;VEGFC;VEGFB;FGF7;FGF6;FGF5;FGF4;FGF3;FGF2;FGF1;RAC2(up);RASA3;RAB5B;CALML6;CALML5;INS;PAK2;FGF22;RALB;FGF20;PDGFRB;PDGFRA;FGF9;RALBP1;GNB4(up);FGF8;LOC102153034(down);EPHA2;RAP1A;GNG11;LOC106559087;PLCE1;GNG12;REL;RASGRF1;ABL1;FLT4;ABL2;RAF1;RHOA;FLT1;STK4;LOC119863873;TEK;TBK1;GNGT2;PAK4;CSF1R;ARF6;RASA1;MAPK3;MAPK1;NFKB1;FGF11;FGF10;FGF17;FGF16;KDR;EXOC2;NTF3;RALGDS;PAK5;GNB5;RASSF5;EFNA5;EFNA4;EFNA3;EFNA2;EFNA1;GNB2;FLT3LG;PLA2G2D;PLA2G2F;PLA2G10;MAPK8;LOC119866928;MAPK9;SOS2;RASGRF2;PLCG2;AFDN;RIN1;MAP2K1;BAD;LOC111094784;MET;SHOC2;PRKACA;NGFR(up);PRKACB;CDC42;GNB1;LOC119869603;EGFR;FLT3(up);MAP2K2;INSR;FOXO4;CALML4;PGF;RAB5C;RGL1;RGL2;NRAS;ETS1;ZAP70;LOC609053;FGF18;GNG10;PDGFA;PDGFB;PDGFC;PDGFD;RAC3;RAC1;PRKCA;PLAAT3(down);PRKCB;PRKCG;SYNGAP1;GNB3;HGF;PLA2G4B;PLA2G4A;PLA2G4F;RASA2;PLA2G4D;PAK1;BRAP;LOC480788;GRIN2B;GRIN2A;FGFR4;RALA;GNGT1;PLA2G2E;LOC100855681 |
| Fructose and mannose metabolism | TIGAR;LOC482320;TPI1;GFUS;FBP2;PMM2;PMM1;ALDOC;ENOSF1;HKDC1;PFKP;PFKM;GMDS;HK2;HK3;HK1;GMPPA;GMPPB;FPGT;ALDOA;LOC608800;AKR1B1;ALDOB;PFKL;SORD(up);FCSK;PFKFB1;PFKFB3;PFKFB2;PFKFB4;TKFC;MPI;KHK;FBP1(up) |
| Oxytocin signaling pathway | PTGS2;ACTG1;PPP3R1;PRKAG1;PRKAG3;ADCY1\_1;MYL6;PPP3R2;LOC488190;MYL9;EEF2K;CALM2;CALM3;CALM1;CAMK1;RYR3;CACNA1C;CACNA1D;CAMK4;CACNA1F;CACNA1S;NPR1;NPR2;PRKAG2;CAMK2D;CAMK2G;CAMK2A;CAMK2B;ADCY4(up);CACNA2D2;CACNA2D3;CACNA2D1;CACNA2D4;KRAS;ADCY5;ADCY6;ADCY7;ADCY1;ADCY2;ADCY3;CCND1;ADCY8;MYLK;ELK1;GUCY1B1;ACTB;NFATC1;NFATC2;NFATC3;NFATC4;GNAO1;HRAS;ADCY9;CACNB2(up);RYR1;GNAI2;FOS;JUN;GUCY1A1;GUCY1A2;TRPM2;NPPA;LOC119869567;PLCB2;PLCB3;PLCB4;PRKAB1;OXT;PRKACB;GNAI1(up);ITPR2;ITPR3;RHOA;ITPR1;GNAQ;GNAS;CALML6;CALML5;CALML4;MEF2C;CAMKK2;RYR2;RCAN1;LOC102153034(down);RAF1;GNAI3;PRKAB2;CACNB3;CACNB1;CACNB4;MAPK3;MAPK1;PPP1R12A;PPP1R12B;PPP1R12C;CDKN1A;MAPK7;SRC;LOC485435;MYLK2;MYLK3;PLCB1(up);KCNJ3;KCNJ6;KCNJ5;KCNJ4;KCNJ9;PRKACA;OXTR;MYL6B;CAMK1D;CAMK1G;EGFR;PRKAA2;PRKAA1;CD38(up);MAP2K2;MAP2K1;MAP2K5;PPP3CB;NRAS;RGS2;KCNJ12;NOS3;KCNJ14;PRKCA;KCNJ2(down);PRKCB;PRKCG;PLA2G4E(up);PPP3CA;PLA2G4B;PLA2G4A;PLA2G4F;PLA2G4D;PPP3CC;PPP1CB;PPP1CC;PPP1CA;ROCK1;ROCK2;CACNG6;CACNG1;CACNG2;CACNG3;CACNG4;CACNG5;EEF2;CACNG7 |
| Folate biosynthesis | AKR1C3;TPH1;DHFR;QDPR;SPR;GCH1;AKR1B1;MOCS2;TPH2;LOC100688446;FPGS;TH;LOC610164;PAH;ALPI;GPHN;PTS;GGH;LOC609048;ALPL |
| Neurotrophin signaling pathway | LOC100856339;CRKL;IKBKB;AKT1;AKT2;AKT3;RAPGEF1;BDNF;CALM2;CALM3;IRS1;PIK3CA;PIK3CB;MAP3K1;PIK3CD;NTRK1;NTRK2;MAP3K5;IRAK2;IRAK3;RELA;IRAK1;NFKBIE;IRAK4;SHC4;SHC1;SHC2;SHC3;CAMK2D;TP73(down);CAMK2G;CAMK2A;CAMK2B;FASLG;KRAS;MAGED1;PTPN11;GSK3B;GRB2;ZNF274;NGF;NFKBIB;HRAS;NFKBIA;FRS2;CRK;MAPKAPK2;JUN;PIK3R2;PIK3R1;LOC119870216;LOC119870217;LOC100683481;LOC119870215;RHOA;RPS6KA1;RPS6KA2;TP53;RPS6KA6;CALM1;CALML6;CALML5;CALML4;PRDM4;MAP3K3;SORT1;MATK;LOC102153034(down);RAP1B;RAP1A;CAMK4;MAPK14;MAPK10;MAPK11;MAPK12;MAPK13;ABL1;NTRK3;RAF1;PSEN2;PSEN1;MAPK3;MAPK1;MAPK7;NFKB1;MAPK8;MAPK9;NTF3;KIDINS220;SH2B2;SH2B3;SH2B1;GAB1;LOC119866928;SOS2;MAP2K2;PLCG1;BAD;NGFR(up);CDC42;BAX;FOXO3;PLCG2;MAP2K1;RIPK2;MAP2K7;MAP2K5;NRAS;ARHGDIG;ARHGDIB;ARHGDIA;YWHAE;BEX3;RAC3;TRAF6;RAC1;PRKCD;ATF4;RPS6KA5(up);BRAF;PDPK1;LOC608573;BCL2 |
| Complement and coagulation cascades | SERPINB2(up);LOC477699;CD55;PLAUR;VSIG4;CD59;C1QB;C8A;C8B;SERPING1;CFB(down);C1QA;C8G;F13A1;PROS1(down);TFPI(down);SERPINE1;C1R(up);LOC102156626;LOC481722;SERPINA5;LOC609365;SERPINC1;LOC490269;C4BPA;C4BPB;CPB2;C9;F2(down);C3;C2;FGG;C7;FGA;FGB;LOC119869344;ITGAX;LOC100855476;ITGAM;CFI;KLKB1;SERPINA1;F13B;CFD(down);PLAU;PLAT;F2RL3;F3;SERPINF2;F12;CLU;F10;F11;SERPIND1;BDKRB1;F5(up);VTN;BDKRB2;ITGB2(down);ITGAX\_1;CFH;F7;F8;F9;KNG1;C1QC;MASP1;MASP2;C6;PLG;F2R;C5AR1;C5;C3AR1;C1S;F2RL2;VWF;PROCR;PROC;THBD(up);LOC479260 |
| Platelet activation | PTGS1;LOC100856339;ACTG1;FYN;ADCY1\_1;LOC119870307;AKT1;AKT2;AKT3;LOC488190;PIK3CA;PIK3CB;PIK3CD;ARHGEF1;COL1A2(up);GP9;GP6;FGG;FGA;FGB;F2(down);LOC607207;ARHGEF12;TBXAS1;PLA2G4E(up);ADCY4(up);ADCY5;ADCY6;ADCY7;ADCY1;ADCY2;ADCY3;SYK;SNAP23;ADCY8;MYLK;F2R;GUCY1B1;ACTB;RASGRP2;PRKCI;RASGRP1;APBB1IP;ITGA2;ADCY9;PLCB3;COL3A1;GUCY1A1;GUCY1A2;PRKG1;PIK3R2;PIK3R1;PRKG2;LOC119869344;LOC119869567;PLCB2;ORAI1;PLCB4;LOC119870217;LOC119870216;LOC119870215;COL1A1;GNAI1(up);ITPR2;ITPR3;RHOA;ITPR1;GNAQ;GNAS;VWF;FERMT3;RAP1B;RAP1A;MAPK14;MAPK11;MAPK12;MAPK13;GNAI2;GNAI3;FCER1G;ITGA2B;GP1BA;P2RY12;GP1BB;MAPK3;MAPK1;PPP1R12A;TBXA2R;LCP2;LOC485435;MYLK2;MYLK3;PLCB1(up);LYN;LOC102151885;MYL12B;VASP;PRKACA;PRKACB;F2RL3;STIM1;SRC;PLCG2;P2RX1;TLN2;TLN1;ARHGAP35;ITGB1;ITGB3;NOS3;PTGIR;PRKCZ;PLA2G4B;PLA2G4A;P2RY1;PLA2G4F;PLA2G4D;PPP1CB;PPP1CC;PPP1CA;VAMP8;ROCK1;ROCK2;GNA13 |
| Antigen processing and presentation | CD74;CALR;TAP2;KLRD1;DLA-64;HSPA2;LOC119869603;HSPA8;LOC119869607;LOC102156776;NFYC;NFYB;NFYA;TNF;LOC607182;HSP70;LOC100687242;CANX;LOC106559087;TAPBP;LOC111090226;CD8B;HSPA5;TAP1;CIITA;HSPA4;LOC102156901;HSP90AB1;IFNG;RFXAP;LOC609053;CTSB;LOC102156003;RFXANK;LOC100856137;LOC119873859;LOC486692;DLA-79;DLA88;LOC119881611;HLA-DRB1;PDIA3;LOC119872979;CREB1;PSME1;PSME3;LOC612100;IFI30;CD8A;CTSS;CTSV;CD4;LOC100683403;B2M;LGMN;LOC480788;DLA-DOA;DLA-DOB;PSME2;LOC119866425;DLA-DMB;DLA-DMA;RFX5;HSP90AA1;DLA-DRA(down);LOC609192;LOC474850 |
| Neutrophil extracellular trap formation | LOC100856339;ACTG1;LOC100682891;LOC607467;AKT2;AKT3;FCGR1A;PIK3CA;PIK3CB;NCF4;PIK3CD;MAP3K7;LOC106559121;RELA;FGG;FGA;FGB;FPR2;SLC25A6;SLC25A5;SLC25A4;LOC119879788;LOC106559212;AKT1;SYK;PLCG1;LOC106559111;TLR2;C5AR1;TLR7;TLR8;SLC25A31;LOC609798;PIK3R2;PIK3R1;LOC119869344;PLCB2;PLCB3;PLCB4;LOC119870217;LOC119870216;LOC119870215;ACTB;TLR4(down);RAC2(up);MPO;AQP9;ITGAL;ITGAM;NCF1;HDAC1;NCF2;HDAC3;HDAC2;LOC609805;MAPK14;MAPK11;MAPK12;MAPK13;VDAC3;VDAC2;VDAC1;LOC490269;RAF1;LOC102153980;ITGA2B;GP1BA;MAPK3;MAPK1;C3;NFKB1;C5;SRC;LOC111097181;PLCB1(up);MTOR;LOC119869025;ITGB2(down);MAP2K2;LOC102154760;PPIF;LOC478984;HMGB1;PLCG2;MAP2K1;CLEC7A(down);CAMP;SELPLG;ATG7;ITGB3;RAC3;RAC1;PRKCA;PRKCB;PRKCG;CYBA;LOC119869046;CTSG;VWF;SELP(up) |
| Ribosome biogenesis in eukaryotes | MDN1;UTP6;RPP38;UTP4;EFL1;IMP3;RPP30;RIOK1;LOC481575;TBL3;REXO1;NOP10;REXO2;LOC119867648;EIF6;PWP2;WDR3;EMG1;FBLL1;LOC100687327;NXF1;XPO1;GNL2;LOC492024;DROSHA;NMD3;NOP58;HEATR1;RAN;LOC609886;NOP56;GNL3L;REXO5;NOB1;GTPBP4;WDR36;UTP15;RBM28;LOC111096540;GAR1;GNL3;RPP40;TCOF1;XRN2;NXF3;SBDS;LOC486757;RPP25;LOC119870192;UTP14A;BMS1;SNU13;LOC100684788;MPHOSPH10;NOL6;CSNK2A2;CSNK2A1;NAT10;RIOK2;UTP18;WDR43;RPP25L;FCF1;LOC100686262;AK6;LSG1;NXT2;POP1;NXT1;POP7;XRN1;POP5;DKC1;LOC102152908;NHP2;RRP7;NVL;WDR75;CSNK2B;SPATA5;FBL |
| Hedgehog signaling pathway | FBXW11;IHH;EVC;GRK3;ARRB2;CCND2;ARRB1;BOC;SMURF1;SMURF2;SPOP;GRK2;GAS1;EVC2;GLI2;GLI3;CSNK1D;GLI1;GPR161;CUL1;CUL3;SPOPL;DHH;SMO(up);KIF7;CSNK1G2;CSNK1G3;CSNK1G1;SUFU;SHH;KIF3A;CSNK1A1;CCND1;BTRC;GSK3B;LRP2(up);CSNK1E;CDON;PRKACA;PRKACB;PTCH2;BCL2;PTCH1;MGRN1;HHIP |
| Isoquinoline alkaloid biosynthesis | TAT;AOC3;AOC2;MAOB;LOC482436;GOT2;MAOA;TH;DDC;IL4I1;GOT1;TYR |
| Butanoate metabolism | ACADS;ABAT;AACS;GAD1;ACAT1;BDH2;ACSM3;HADHA;ACSM5;HMGCL;ACAT2;HMGCLL1;HMGCS1;HMGCS2;LOC479821;LOC479822;LOC483960;EHHADH;BDH1;HADH;L2HGDH;ECHS1;OXCT1;ALDH5A1;LOC119872128 |
| Chemokine signaling pathway | LOC100856339;CRKL;NRAS;ADCY1\_1;IKBKB;AKT1;PTK2B;AKT3;TIAM1;GNG14;PIK3CA;PIK3CB;GRK5;PIK3CD;LOC119874472(up);FGR;GNG13;LOC119863873;LOC119866928;LYN;KRAS;JAK3;SHC4;SHC1;SHC2;SHC3;CCR10;ADCY4(up);CX3CL1;CXCL13;CXCL12;CXCL14;CXCL16;ADCY5;ADCY6;ADCY7;ADCY1;CX3CR1;ADCY3;ADCY8;ADCY9;GSK3B;GNG7;GNG4;GNG5;GNG2;GNG3;ARRB2;ELMO1;GNG8;CCL2;CCL3;CCL1;CCL7;CCL4;CCL5;DOCK2;CXCL8(up);CCL8;ARRB1;CRK;ADCY2;GRK4;PREX1;RAF1;LOC100855681;CCL28;GRB2;JAK2;PIK3R2;PIK3R1;CCL21;CCL20;CCL23;CCL25;CCL27;LOC119869567;PLCB2;PLCB3;PLCB4;LOC119870217;LOC119870216;LOC119870215;CHUK;GNAI1(up);AKT2;HCK;RHOA;BCAR1;GRK2;GSK3A;RAC2(up);GNAQ;VAV1;VAV3;VAV2;PAK1;GRK6;GRK7;GRK3;RASGRP2;NCF1;GNB4(up);RAP1B;RAP1A;GNG11;CCR1;CCR2;CCR3;CCR4;CCR5;CCR6;CCR7;CCR8;CCR9;GRK1;GNAI2;GNAI3;STAT3;STAT2;STAT1;MAPK3;PPBP;MAPK1;NFKB1;BRAF;HRAS;SRC;NFKBIA;GNB5;GNB1;GNB3;GNB2;PLCB1(up);WAS;RELA;CXCL10(down);CCL13;ITK;CCL19;LOC119876918(up);MAP2K1;BAD;STAT5B;PRKACA;PRKACB;SOS2;CDC42;PTK2;FOXO3;PLCG2;PLCG1;WASL;NFKBIB;XCR1;CXCR3;CXCR2;CXCR1;CXCR6;CXCR5;PXN;GNG10;RAC3;RAC1;PRKCB;PRKCD;PRKCZ;PARD3;LOC480600;LOC106557449;LOC119863891(up);ROCK1;ROCK2;GNGT2;GNGT1;GNG12 |
| Histidine metabolism | AOC1;ALDH3B1;HAL;UROC1;ALDH9A1;MAOB;ALDH3A1;HNMT(up);ALDH2;AMDHD1;MAOA;FTCD;HDC;CARNS1;ALDH7A1;ALDH1B1;ASPA(up);ALDH1A3;CNDP1;CNDP2;CARNMT1 |
| Cytokine-cytokine receptor interaction | LIF;CRLF2;TNFRSF25;GHR;LOC100855618;GDF7;IL1R2(up);CCL4;IL6ST;IFNAR2;EPO;IL22;IL25;LIFR;CCL23;IL26;LTBR;IFNLR1;CD70(up);IL12RB1;PRL;IL12RB2;CCL5;LOC119874472(up);CXCL14;RELT;INHBA;OSM;CXCR6;IFNK;IL18RAP(up);IL2RA;IFNG;CCR10;CD27;CX3CL1;CLCF1;CCL13;FASLG;CSF3R;CXCL13;CXCL12;IL22RA1;CXCL16;BMP7;IL21;BMP5;BMP4;GH1;CX3CR1;CSF1;CSF2;CSF3;IFNAR1;BMPR2;EDAR;ACVR1C;CSF2RB;TNFSF12;TNFSF11;IL20RB;IL10RA;IL10RB;ACVR1B;CCR3;CXCL8(up);CCL8;IL12A(up);TNFRSF17;TNFRSF14;IFNGR1;IFNGR2;LEP;IL1A(up);TNFRSF12A;IL1R1;AMH;TNFRSF19(down);PRLR;LOC611406;CCL28;TNFRSF8;TNFRSF9;TNFRSF4;CCL20;LOC119876828;CCL25;CCL27;NGFR(up);IL5RA;IL4R;TNFSF13;CD40;CNTFR;EPOR;IL13RA2;LOC100686162;TNFRSF1B;TNFRSF1A;LOC100683567;MPL;IL11RA;IL13;IL15RA;IL17RB;IL17RA;IL33;BMP6;IL20RA;TNFSF18(up);CD40LG;CCL3;NODAL;NGF;IL27RA;CD4;CCR1;CCR2;CSF2RA;CCR4;CCR5;CCR6;CCR7;CCR8;CCR9;IL1B;IL29L;TNFSF13B;TNFSF10;TNFSF15;IL11(up);TNFSF14;CSF1R;IFNB1;PPBP;TNFSF8;TNFSF4;IL17D;TGFBR2;ACVR2B;IL17A;IL17B;IL7R;IL13RA1;IL6R;LTB;LTA;IL3RA;CCL2;TNFRSF13B;TNFRSF13C;CXCL10(down);IL18;IL19;IL2RB;EDA;IL2RG;IL10;CCL19;LOC119876918(up);IL21R;IFNA7;IL15;IFNA5;IL6;IL7;IL4;IL5;IL2;IL3;IL9;TGFB1;TGFB2;TGFB3;ACVR1;IL23A;ACVR2A;IL12B;LOC607768;TGFBR1;BMPR1A;BMPR1B;IL23R;BMP15;TSLP;CTF1;FAS;AMHR2;XCR1;GDF5;THPO;CXCR3;CXCR2;CXCR1;OSMR;CCL1;TNFRSF18;CXCR5;CNTF;IFNL1;IL18R1;LEPR;TNF;TNFRSF11A;TNFRSF11B;IL1RAP;CCL21;LOC480600;LOC106557449;LOC119863891(up);TNFRSF21;ACKR3;CCL7 |
| Biosynthesis of vancomycin group antibiotics | TGDS |
| HIF-1 signaling pathway | LOC100856339;TIMP1;AKT1;AKT2;AKT3;LOC100686034;EGF;LTBR;HKDC1;PIK3CA;PIK3CB;PIK3CD;EDN1(down);RELA;LOC477441;CUL2;ERBB2;LOC477071;IGF1R;CAMK2D;HK3;CAMK2G;CAMK2A;CAMK2B;LOC490690;LOC608800;EPO;LOC102154094;LOC100685367;ARNT;SLC2A1;LDHA;LOC111094513;LOC106558345;LOC102151775;LOC481849;CDKN1B;CDKN1A;GAPDH;LOC119866339;LOC482320;NOS3;IFNGR1;IFNGR2;SERPINE1;LOC119870849;TFRC;PIK3R2;PIK3R1;ANGPT2;PDHB;ANGPT1;PGK2;ANGPT4;PGK1;IGF1;LOC119870217;LOC119870216;LOC119870215;IFNG;LOC100684709;VEGFA;RBX1;EIF4E;EGLN3;EGLN2;EGLN1;HMOX1;INS;MAP2K2;LOC100686736;EIF4EBP1;CREBBP;MKNK2;MKNK1;MAP2K1;RPS6;ELOB;ELOC;FLT1;TEK;STAT3;RPS6KB1;RPS6KB2;MAPK3;MAPK1;LOC610338;NFKB1;PDHA2;NOS2(up);LOC102152592;TLR4(down);LDHAL6B;IL6R;ENO1;ENO2;ENO3;MTOR;HIF1A;EP300;NPPA;PLCG2;INSR;IL6;EIF4E1B;LOC119876526;LOC106559872;EGFR;LOC477072;LOC491854;VHL;PLCG1;LOC111096459;PFKP;HK2;PFKM;PFKL;LOC111089999;LOC119870407;LDHB;LDHC;HK1;LOC100683724;PRKCA;PRKCB;LOC609911;PRKCG;LOC102153001;LOC100685582;ALDOA;ALDOC;ALDOB;EIF4E2;LOC608772;PFKFB3;PDK1;ENO4;BCL2 |
| Gastric cancer | LOC100856339;AKT1;AKT2;AKT3;ARAF;MLH1;FGF20;EGF;WNT8A;GADD45A;PIK3CB;PIK3CD;ABCB1;GADD45G;CDH1;MYC;SHC4;SHC1;SHC2;SHC3;FGFR2;KRAS;CDKN2B\_1(down);CCND1;TCF7L1;TCF7L2;GSK3B;GRB2;APC;WNT7A;WNT7B;CDKN1B;HRAS;GAB1;FZD10;BAK1;CCNE2;POLK;CCNE1;LOC106557930;PIK3R2;PIK3R1;TCF7;LOC119870217;LOC119870216;LOC119870215;FGF7;LOC489647;FGF9;FGF8;SHH;FGF6;FGF5;FGF4;FGF3;FGF2;FGF1;TP53;CSNK1A1;FRAT1;LOC119863906;PIK3CA;WNT5B;WNT5A;FGF22;GADD45B;WNT10A;WNT3A;SMAD4;SMAD2;SMAD3;HGF;TGFB3;RAF1;FZD1;FZD2;FZD3;FZD4;FZD6;FZD7;FZD8;FZD9;RPS6KB1;RPS6KB2;MAPK3;MAPK1;DDB2;FGF11;FGF10;FGF17;CDKN1A;TGFBR2;TGFBR1;RXRG;RXRA;RXRB;MTOR;LEF1;LRP6;SOS2;LRP5;WNT3;WNT2;WNT1;WNT6;WNT4;MET;CDK2;TGFB1;TGFB2;WNT10B;EGFR;BAX;CTNNB1;MAP2K2;MAP2K1;FGF16;AXIN2;AXIN1;NRAS;RB1;DVL2;DVL3;WNT8B;DVL1;FGF18;BRAF;WNT2B;APC2;CTNNA1;CTNNA2;CTNNA3;E2F3;E2F2;E2F1;ERBB2;WNT9B;WNT9A;WNT16;WNT11;BCL2 |
| NF-kappa B signaling pathway | PTGS2;IKBKB;LOC607467;LTBR;LOC111090226;GADD45A;GADD45B;MAP3K7;GADD45G;LOC106559121;RELB;LYN;IRAK1;IRAK4;BLNK;TRIM25;EDA;LOC119879788;LY96;LOC119874472(up);CXCL12;CSNK2A2;CSNK2A1;LOC106559212;SYK;LOC106559111;CYLD;EDAR;LAT;TNFSF11;CCL4;TNFSF14;CXCL8(up);NFKBIA;PIDD1;IL1R1;DDX58;BCL2L1(down);TIRAP;LOC102156901;CCL21;MYD88;LOC119872979;CHUK;UBE2I;TLR4(down);RELA;BCL2A1;TNFAIP3;CD14;LCK;CD40LG;ATM;LOC102156776;LOC106559087;XIAP;IL1B;TNFSF13B;TICAM1;TICAM2;LOC102153980;TRADD;CFLAR;ICAM1;ERC1;NFKB1;NFKB2;LOC485869(down);LOC119869607;LOC111097181;PHF19;LTB;LTA;VCAM1;TNFRSF13C;LOC119869025;CCL13;CCL19;LOC119876918(up);TNFRSF1A;PLCG1;TAB3;LOC102156003;PIAS4;CSNK2B;LOC119869603;BIRC3;BIRC2;RIPK1;PLCG2;LOC102154760;ZAP70;LOC609053;MALT1;TRAF2;TRAF3;TRAF5;TRAF6;CARD11;PRKCB;CD40;PLAU;TNF;TNFRSF11A;LOC119869046;PRKCQ;BCL10;LOC480788;TAB2;TAB1;MAP3K14;BCL2 |
| Starch and sucrose metabolism | PGM2L1;AGL;G6PC1;G6PC3;G6PC2;LOC607460;UGP2;HKDC1;MGAM;LOC479379;PYGB;GBE1;GBA3;PYGL;MGAM2;GAA;GPI;HK2;HK3;GCK;HK1;PGM1;PGM2;ENPP1;GYS1;GANC;GYS2;ENPP3(down);TREH;PYGM;SI;NUDT11 |
| ECM-receptor interaction | ITGA8;HMMR;ITGA10(up);TNC;CHAD;ITGA9;GP1BA;ITGA2;HSPG2;ITGA4;ITGB3;ITGA6;ITGA7;ITGA5;SV2C;LAMB1;LAMB3;LAMB2;COL2A1;LAMB4;ITGB4;ITGB8(down);TNN;ITGA3;THBS2;IBSP;THBS4;ITGB6;TNXB;THBS3;GP1BB;DAG1;COL1A2(up);GP9;ITGA1;SV2A;GP6;SV2B;RELN;ITGB1;LAMA1;LAMA2;LAMA3;ITGB5;LAMA5;ITGB7;TNR;LOC100855768;COMP;CD47;SDC1;COL9A2;COL9A3;CD36;COL9A1;COL4A6;COL4A5;COL4A4;COL4A3;COL4A2;COL4A1;LAMC2;LAMC3;LAMC1;VWF;AGRN;VTN;LAMA4(up);CD44;FN1;LOC610614;SDC4;ITGA2B;ITGAV;ITGA11;SPP1;COL1A1;COL6A1;COL6A3;COL6A2;COL6A5;THBS1;COL6A6 |
| Olfactory transduction | OR52B2;OR52B4;OR6C74;OR10T2;LOC488617;OR10T1;OR2L5;OR2L3;LOC119864931;OR2D37;OR52N4G;OR52N4F;OR5AK6;LOC119863872;LOC485179;OR9I2;OR7D2;OR10S1;OR9I1;OR52E22;OR7A54;OR5AK8;OR7D4;OR5J2;OR5J1;OR7A55;CAMK2D;CAMK2G;CAMK2A;CAMK2B;OR7R1;OR4G18;OR13D1B;OR5B2;OR4G11;OR2D3G;OR7A51;OR13C4;OR5M3C;OR13C1;OR13C2;OR13C3;OR1P1;OR13C8;OR9S26;LOC485275;OR9S25;OR5A2;OR5A3;OR5A1;OR2L17;OR7A70;OR7A71;OR7A73;OR7A74;ANO2;OR52K1B;OR5D18;OR2F1D;OR2W3;OR2T4B;OR2W1;OR5D15;OR5D14;OR5D17;OR2T4D;OR6C3I;OR11K2;OR9R6;OR9R5;OR1I1;OR5AR1;OR52P1;OR52P2;GUCY2D;LOC489342;OR5T1;OR51A18;OR51A10;CNGA2;OR4E1;OR8S13;LOC119864882;CNGA3;OR6C6;OR55B1;LOC482215;OR10AG68;OR4K6;OR4K1;OR4K2;OR4A39;LOC483612;OR10AG66;OR10AG64;OR10AG65;OR5BE4;OR8G3;OR5T8;OR8B9;LOC491652;LOC491653;OR3A10;OR08B12;LOC119864888;OR4L1;OR5B3B;OR2A5;OR2A7;OR2A9;OR5B31;OR5B33;OR6K8;OR5B35;OR6K2;LOC541584;OR6K5;OR6K4;OR10AA5;OR4A82;OR10AA2;OR3A1H;OR8S1;OR8S3;OR5E1;OR13C2B;OR8S7;OR6P1;OR8S9;LOC403898;OR4H12;LOC119863898;LOC102156012;OR2L5C;GNB1;OR9S5;LOC482569;OR52U1C;OR6B9;OR11G9;OR5W1;OR5W2;OR11G5;OR11G2;OR6B6;OR8A1;OR5W7;OR52L1;OR52L2;OR6C89;PDE1C;PDE1B;PDE1A;OR6B1;LOC485303;OR6B2;OR3A2;LOC485300;OR6C6K;OR13F1;LOC119865984;OR4P32;OR6C63;OR52AB2;OR52AB3;SLC24A4;OR52AB4;OR6C69;OR6C75D;OR2Z3;OR6B2C;OR8K54;OR52Z1;SLC8A3(up);OR8H4;OR5P5;OR5P4;OR5P6;OR2A58;OR8J2C;LOC119864989;OR6C17;OR52U1;OR4C11I;OR4C11H;OR7D7;OR51AI5;OR13P3;OR13P5;OR2D3;LOC100683141;OR10K2;OR6AA7;OR6AA6;OR9A7;OR9A4;OR8AP1;OR5BA1;OR2D2C;LOC486566;OR51A21;OR7G13;LOC119864128;OR51A25;OR51A24;OR4D9B;OR4D9C;OR4D9D;GNG13;OR52H1B;OR52H1C;OR3A1;OR1X2;RGS2;OR5T2B;OR51AB4;OR4A40;OR4A47C;OR4A47;OR4N2B;OR10R3C;GNG7;OR7C1B;OR51F23;OR6F1;OR5K4;OR5K5;LOC485299;OR5K1;OR52H4;OR52H7;OR52H1;OR52H2;OR4C15;OR4C11;OR52H9;LOC485266;PRKG1;OR1F15;PRKG2;OR5B3;OR9S22;OR56A4B;OR56A4C;OR6C2G;OR2AG1;OR51G2;OR51G1;OR2V1;OR51G4;OR6C22;OR11H12;OR6C27;OR8D4;OR8D6;OR5T2;CNGA4;OR6A2;OR5W2B;LOC482247;OR2Z1;LOC541568;OR51V21;OR51V22;OR9S3;OR11J4;OR9S6;OR11J6;OR8BJ4;OR13E1;OR5AQ1;OR4H12B;OR4S7;OR10G6;OR4D2C;OR10G9;OR1D6;OR6AE1;OR4S2;OR2AX2;OR4D9;OR6C69G;OR4D1;OR4D2;OR4D5;OR4D6;OR7A67;OR7A66;OR7A65;OR7A64;LOC119870756;OR7A62;OR7A61;OR7A60;OR8K1;OR7A63;OR8K5;OR8K7;OR7A68;OR2K2;OR10A5F;OR5AK7;OR6C53;OR10AI1;LOC489315;OR2AJ7;OR10G9C;OR6X1;OR10H2;OR10H1;OR13F1B;OR52E7;OR7A69;OR1AD1;OR2T24;OR2T27;OR5AN2;LOC483514;LOC483515;OR7E181;OR5AN6;OR2D4;OR2AT2B;OR10Z1;OR4C59;LOC487457;OR2T4C;OR52D3;OR4C11D;OR10A5D;LOC485224;LOC485225;OR6S1;LOC485221;OR52N2G;OR4Q3;OR4Q2;OR5F2;OR10Q1;OR10Q2;LOC119863959;OR56A3E;OR7G8;OR2R1;OR7G9;OR5H9;OR4C136;OR6E1;LOC482553;OR5L1C;OR5L1B;OR10AG1;NCALD;OR4B1E;OR4B1F;OR9S12;OR2AD2;LOC485259;OR2AH1B;OR13A1;OR4X2;OR11H6;OR6C75;LOC491695;OR10J14;OR8B60;OR7A58;OR7A56;OR7A57;OR10C1;LOC476827;OR7A52;OR7A53;LOC119864606;OR51B9;OR51B5;OR1M1B;OR8B8;OR13C7H;OR13C7I;OR52A18;CALM2;CALM3;CALM1;CNGB1;OR8G6;OR13C7F;OR13C7G;OR7A29;OR4D11;OR7A23;OR51AJ4;OR7A26;OR2AV1;OR2AV2;OR4N2;OR7E153;OR7E154;OR2G6;OR13P4;OR7G10;LOC477589;OR10D4;OR10D5;OR7G14;OR52AD1;OR6M2B;ADCY3;OR4G9;OR4G8;OR2L3B;OR56A3D;OR51AA2;OR10H4D;LOC481603;OR5G1B;OR4F63;OR9G10;LOC482237;OR8U10;OR10V4;OR10V5;LOC482230;LOC489328;LOC487250;OR51A8;LOC119880303;OR13J1;OR5M10;OR5M12;OR5M13;OR2AV12;OR9K1;OR9K2;OR2AV11;OR9K7;OR6D6;OR12J1;OR2A9B;OR52AE1;OR51P1;LOC485282;OR5L1;OR52E4B;OR5AC27;OR5AC26;OR4C10F;OR4C29;OR52I1;OR1R1;OR4F34;OR2T26;OR5C1;LOC484858;OR4C1;OR10AC1;OR2T20;OR8U9;OR8U8;OR4G10;GNAL;OR6N1;OR6N2;OR2T22;OR51F1;OR6C38;OR5J2B;OR2Q2;OR1L8;OR10D1K;OR6C33;OR1L6;OR52W1;ARRB2;OR11I1;OR8C7;ARRB1;OR5AC1;CALML4;OR52N4;OR52N2;OR5AP2;OR13D1;OR13D3;OR13D2;SLC8A1;OR1K1;SLC8A2;OR11G11;OR4P11;OR6M6;OR51M1;OR1E1;OR51V1B;LOC608046;OR4K15;OR7A17;OR8J3;OR10AH1;OR6C42;OR4A15;OR51S1;OR9G4;CALML5;OR9G1;OR9G3;OR2AK3;LOC119863894;LOC119863890;LOC491636;OR5AZ2;OR7A45;OR5H16;OR5H11;LOC483568;OR5H13;OR5H12;OR2T11;OR51T1;OR14I2;OR2C3;OR2T15;OR2C1;LOC608349;OR11G7;OR13C11B;OR13C11C;OR52E2;OR52E1;OR5B12;OR52E8;OR4P4;OR8U3;OR4P6;OR4P7;LOC119864887;OR14J1;LOC119864889;OR7E24;OR2M9;OR52E8C;OR52R1;OR5BH3;OR5G1;OR4N5;OR5G3;OR5G5;OR9K2C;OR4C139;OR9K2D;OR7G6;OR7G7;OR7G5;OR7G3;OR51A4;OR2Q1;LOC490585;LOC119869315;OR4K5B;OR10D5B;LOC482217;LOC102155554;CALML6;OR52A5C;OR6D7;OR6D5;OR52J8;OR52J3;OR52J1;GRK2;GRK3;OR6M1;OR6C4B;OR6M2;OR2AE1;OR10AG63;OR2T1;OR10H5B;OR8J2B;OR52X1;OR8K71;OR52X3;OR8B58;LOC102153034(down);OR52E5;OR2T6;OR51A1;OR8S2;OR1A1;OR2T2;OR51A6;LOC607515;OR9Q3;OR9Q4;OR2AG1D;OR52S1;OR52S2;OR56B2C;OR56B2D;OR5AS1;OR7C19;OR1AF1;OR51AG3;LOC100685324;OR56A3;OR56A1;OR56A9;OR51H5;OR8K86;OR51H2;OR51H1;OR52K1;OR5I1B;OR2B11;OR4A27;OR52A1;OR52A6;OR52A5;OR2AH1;OR10A3E;LOC489332;LOC489331;OR7C2;OR10N1;OR7C1;LOC491645;OR2H10;OR51B18;OR2F2;OR51B10;OR5M8;OR5M9;OR5B24;OR4A47D;OR5M3;OR11G2B;OR5M5;OR10X1;OR4F25;OR5D3;OR6Q1;OR10AB2;OR10AB3;OR13L2;OR7H3;OR10W1;OR9M1;OR10V1;LOC106559671;OR5AL2;OR2AI2;OR51E1;OR51E2;OR1M1;OR8B3;OR6C1;OR8B1;OR11H7;OR52A16;OR8B4;OR6C4;OR8S10;OR10V10;OR8S12;OR8S15;LOC488311;OR5B2B;OR13G1;OR56A4;PRKACA;PRKACB;LOC102154596;OR10A3;OR10A5;OR56A12;OR10A7;OR10A6;LOC486582;OR52AC1;OR52M2;OR51L2;OR51L1;OR5B32;OR51L5;OR51L4;OR5B21;PDE2A;LOC100684060;OR8B100;OR2W11;OR51Q1F;OR2AZ1;OR8B12;OR2T13;OR9S16;OR2AT2;OR6Z1;OR6Z2;OR6Z3;OR8B1L;OR8B1M;OR8B1N;OR8B1O;OR51R1;OR8B1J;OR8B1K;OR10J4;OR10J5;OR10J2;OR51K7;OR5AL1;OR9S19;OR51K2;OR51K1;OR56B6;OR56B2;OR56B1;OR2B8;OR1AB2;OR51AC4;OR56A1B;OR4A2;OR5T1B;OR2B7 |
| FoxO signaling pathway | PCK2;PCK1;PRKAG1;PRKAG3;PRKAG2;PLK4;IKBKB;CDKN2B\_1(down);AKT2;AKT3;ARAF;EGF;PIK3CA;PIK3CB;PIK3CD;GADD45G;PLK1;CSNK1E;PLK3;IGF1R;PLK2;SOD2;CDKN2D;PRMT1;FASLG;MDM2;SETD7;KRAS;AKT1;CCND1;CCND2;STK11;PTEN;GRB2;LOC100856339;TNFSF10;CDKN1B;HRAS;G6PC1;G6PC3;G6PC2;IL10;USP7;PIK3R2;PIK3R1;NLK;GRM1;SIRT1;IGF1;PRKAB2;PRKAB1;LOC119870217;LOC119870216;LOC119870215;CHUK;RAG2;ATG12;RAG1;STAT3;BNIP3;INS;FBXO32;GADD45A;GADD45B;CREBBP;IL7R;SMAD4;ATM;LOC487020;MAP2K1;MAPK14;CCNG2;MAPK10;MAPK11;MAPK12;MAPK13;GABARAP;RAF1;SKP2;S1PR4;MAPK3;MAPK1;FBXO25;MAPK8;MAPK9;CDKN1A;TGFBR2;TGFBR1;RBL2;AGAP2;EP300;SGK3;SOS2;SGK1;IRS4;IRS1;IRS2;IL6;CDK2;TGFB1;TGFB2;TGFB3;FOXG1;EGFR;PRKAA2;PRKAA1;CAT;FOXO3;MAP2K2;FOXO1;FOXO6;FOXO4;LOC111096471(down);SGK2;BCL2L11;GABARAPL2;NRAS;KLF2;HOMER1;HOMER3;PDPK1;GABARAPL1;CCNB1;SLC2A4;INSR;BRAF;STK4;BCL6 |
| Phototransduction | LOC485179;LOC102153034(down);RCVRN;GNAT1;GNAT2;CALM2;CALM3;CALM1;CNGB1;GRK7;SAG;RGS9(up);GRK1;GUCY2D;RHO;GUCY2F;GNB1;SLC24A1;CNGA1;CALML6;CALML5;CALML4;PDE6B;PDE6A;PDE6G;GNGT1;GUCA1A;GUCA1C;GUCA1B |
| Cell adhesion molecules | DLA-64;NRCAM;CD34;GLG1;TIGIT;VTCN1;L1CAM;CLDN16;LOC111090226;NTNG1;LOC102156776;SPN;CDH1;CDH3;CDH2;CDH5;CDH4;LOC102156003;LOC100856137;DLA-79;CD22;HLA-DRB1;NEGR1;NTNG2(up);NRXN3\_1;LRRC4C;LRRC4B;NRXN3;NRXN2;NRXN1;NECTIN3(down);MAG;ITGA8;ITGA9;ITGA4;NCAM1(up);ITGA6;CNTN2;CNTN1;ICOSLG;PECAM1(up);LOC102156901;CLDN8;CLDN9;MADCAM1;CLDN1;CLDN2;CLDN3;CLDN4;CLDN5;CLDN6;CLDN7;CTLA4;CLDN20;JAM3;DLA88;LOC119872979;NFASC;LOC489647;F11R;CD99;SDC1;SDC3;SDC4;ITGAV;SELL(up);ITGAL;ITGAM;ESAM;CD80;MPZ;CLDN10;CLDN11;CD40LG;CLDN17;CLDN14;CLDN15;CLDN18;CLDN19;CD4;CD6;CD2;NCAM2;LOC106559087;ITGB8(down);PVR;MPZL1;ALCAM;CD86;NECTIN2;NECTIN1;ICAM3;ICAM1;CADM3;CADM1;OCLN;ICOS;NLGN2;NLGN3;NLGN1;VCAM1;CD8A;CD8B;ITGB2(down);DLA-DOA;DLA-DOB;CD274;PDCD1;CD276;CDH15;LOC119869603;CD58;VCAN;LOC119869607;CNTNAP1;CNTNAP2;SELPLG;LRRC4;NEO1;LOC609053;PTPRC;ITGB1;ITGB7;CLDN23;LOC119881611;NLGN4X;CD40;CLDN25;CLDN24;PDCD1LG2;CD28;LOC480788;SELE;SELP(up);DLA-DRA(down);DLA-DMB;PTPRF;DLA-DMA;CD226(up);LOC482919;PTPRM;SIGLEC1 |
| Biosynthesis of secondary metabolites | AGL;ALDH7A1;GPAT3;GPAT2;PMM2;PMM1;ASS1;SQLE;TAT;OGDH;HKDC1;TKTL1;HMGCLL1;LOC482436;BCAT2;BCAT1;ACACB(down);LOC608800;LOC483960;LOC102151507;LOC119868057;SUCLA2;HSD17B12;LOC102156116;HSD17B10;AASS(down);AK7;ALDH3A1;LOC102156938;LOC482320;UROD;PAICS;LSS;KYAT1;KYAT3;GART;LOC490770;EPRS1;PISD;CBS;SC5D;MBOAT1;FH;LOC100685679;HMBS;PKLR;NT5C3A;LOC611724;NT5C3B;ALAS1;ALAS2;LOC610338;LOC102153601;NUS1;CPOX;MVD;ALDH9A1;MCAT;SDSL;MVK;AK9;AK8;AK3;AK2;AK1;PGLS;AK6;AK4;SI;LOC119876526;MDH1;PLA2G12A;FLAD1;CAT;BHMT2;PGD;LPIN2;LPIN3;LPIN1;IMPDH1;IMPDH2;LOC479379;ACAA2;ACAA1;HSD17B7;NOS1;ALDH3B1;NOS3;BPNT2;LOC609911;GANC;LOC119866771;PLA2G4B;PLA2G4A;PLA2G4F;PLA2G4D;PNP;CEPT1;PCK2;GRHPR;PCK1;TGDS;IDI1;MAT1A;NQO1;PYCR3;PYCR2;PYCR1;SELENOI;LOC477441;ZMPSTE24;PLD4;ADPGK;IMPA1;IMPA2;NME7;PHGDH;LOC102152410;LOC481043;NME2;LOC102151128(down);NME3;ECHS1;LOC111094513;DBT;TYR;GGCX;NSDHL;LOC102154822;PPOX;HDC;PLA2G1B;LOC485342;PLA2G12B;MGAM;TKT;FDFT1;PKM;DLD;GCK;DLAT;ACLY;RFK;BLVRB;BLVRA;CYP2R1;ICMT;HACD1;HACD3;HACD2;PLA2G10;HACD4;GLYCTK;MAOB;MAOA;COX10;LOC476602;COX15;AMPD2;AMPD1;UGT2A3;LOC102154725;PGAM2;PGAM1;ADSL;FECH;VKORC1;LOC100687518;LOC477562;ASL;PLPP1;PLPP3;PLPP2;PLPP5;LOC102152592;GMPPA;GMPPB;ENO1;ENO2;NUDT11;ENO4;ACADSB;UROS;IL4I1;TRIT1;ACOX3;ACOX1;ALDH2;PPAT;RCE1;PRDX6;LOC608853;ODC1;PLPP4;PFKP;PFKM;PFKL;RPE;LOC111089999;LOC119870407;MAT2A;MAT2B;LSM7;IDH3B;COQ7;DHCR24;OTC;ENO3;BCKDHB;FBP1(up);BCKDHA;GLDC(down);TM7SF2;PLB1(up);PCCB;LOC480777;PCCA;AOC3;TPI1;AOC2;GGPS1;GPAM;PLA2G6;PLA2G5;PLA2G3;CYP51A1;GUSB;DGKZ;GPI;HMGCL;IDH2;IDH1;HMGCR;ACOT7;AGXT2;EHHADH;SHMT2;SHMT1;NME4;PLA2G2C(down);NME6;ATIC;PLD1;PLD2;PLD3;DLST;ACADS;FBP2;LOC119867230;ACADM;RGN;THEM4;NT5M;NT5C;NT5E;XDH;PGK2;PGK1;LOC100684709;DDC;GPD1L;LOC483943;MSMO1;PTDSS2;EARS2;DGKG;LOC102154109;LOC102153215;BHMT;AK5(up);HPRT1;PDHA2;HMGCS1;HMGCS2;ACACA;ACSS2;ACSS1;PLAAT3(down);PLA2G2E;PLA2G2D;PLA2G2F;CS;GALM;CDS1;CDS2;AGXT;ASNS;AGPAT5;AGPAT4;AGPAT3;AGPAT2;AGPAT1;BPGM;LDHAL6B;DGKQ;GPD1;MDH2;LOC102157398;GPD2;LOC111096459;GCDH;ALDH1A3;PCYOX1;DAO(down);NT5C1B;NT5C1A;GAPDH;OGDHL;NT5C2;GAA;IDH3A;AADAT;MTR;ENPP3(down);LOC102153001;SUCLG2;SUCLG1;ALDOA;ALDOC;ALDOB;SDS;PSAT1;EBP;COQ5;HAO1;HAO2;COQ6;COQ3;COQ2;DHDDS;LOC100856533;LOC611563;DHCR7;OAT;LOC607460;LOC100686034;UGP2;PYGB;LOC100688420;LCLAT1;GOT2;GOT1;PYGL;PYGM;PFAS;NME1;FNTA;FNTB;FDPS;HK2;HK3;HK1;PLA2G4E(up);ENPP1;LOC490690;TREH;MBOAT2;LOC102154094;MGAM2;CYP1A2(up);DGKD;LOC106558345;LOC102151775;LOC481849;G6PC1;G6PC3;G6PC2;AKR1A1;UGT1A6(down);IDNK;LOC100683724;PDHX;CTH;PDHB;GBA3;AMT;ACOT4(up);PGM1;PGM2;GYS1;GYS2;ACO1;ACO2;HADH;ARG1;ARG2;HMOX1;MPI;ELOVL6;ELOVL7;ELOVL4;ELOVL5;ELOVL2;ELOVL3;ELOVL1;LOC102152275;GCSH;ALAD;LOC102154742;GADL1;GAD1;ACAT1;ACAT2;TECR;ALDH18A1;ISYNA1;PDSS2;PDSS1;H6PD;FADS2;ADH5;PSPH;LOC102155863;TALDO1;NOS2(up);LOC480491;PAPSS1;PAPSS2;PRPS2;IDH3G;PRPS1;HADHB;GBE1;HADHA;VKORC1L1;DGKK;DGKH;DGKI;RPIA;TH;DGKB;DGKA;LDHA;LDHB;LDHC;NAGS;GAPDHS;ALDH1B1;LOC111091468;DGKE;PEMT;PMVK;SDHA;SDHC;SDHB;SDHD |
| Mitophagy - yeast | YME1L1;LETM1;POLG;MAPK11;MAPK12;MAPK13;GABARAPL1;GABARAPL2;GABARAP;MAPK7;SIN3A;ULK2;PRKCA;USP10;MTOR;LOC102152884;CSNK2A2;CSNK2A1;MAPK14;SNRPD3;PIP5K1C;PIP5K1B;PIP5K1A;FIS1;DNM1L;CSNK2B |
| Prolactin signaling pathway | GALT;TNFSF11;LOC100856339;LOC102154108;PIK3CD;HRAS;TH;FOXO3;MAP2K2;MAP2K1;AKT3;MAPK10;MAPK11;MAPK12;MAPK13;CYP17A1;RAF1;SLC2A2;LHCGR;STAT3;STAT1;PRLR;PRL;PIK3CB;FOS;NRAS;ESR1;CGA;MAPK3;MAPK1;JAK2;PIK3R2;PIK3R1;NFKB1;MAPK8;MAPK9;GCK;CISH;SHC4;SRC;SHC1;SHC2;SHC3;LOC119870217;LOC119870216;LOC119870215;IRF1;CCND2;GSK3B;LOC100684658;ESR2;TNFRSF11A;RELA;KRAS;SOCS5;MAPK14;SOCS7;SOCS6;SOCS1;SOS2;SOCS3;AKT1;CCND1;SOCS4;SOCS2;AKT2;INS;STAT5B;STAT5A;GRB2;CSN2;PIK3CA;ELF5(down) |
| Fatty acid elongation | HSD17B12;THEM4;ELOVL7;HADHB;PPT1;PPT2;HADHA;TECR;ACAA2;MECR;ACOT4(up);ACOT7;LOC490770;LOC481043;HACD1;HADH;HACD3;HACD2;HACD4;ELOVL6;ECHS1;ELOVL4;ELOVL5;ELOVL2;ELOVL3;ELOVL1 |
| Fatty acid biosynthesis | HSD17B8;LOC485024;ACSF3;ACACA;ACSL3;ACSL1;ACSL6;OXSM;ACSL4;ACACB(down);FASN;ACSBG1;ACSBG2;MCAT;MECR;ACSL5(up) |
| Autophagy - yeast | PPP2CB;MTMR6;ATG7;IPMK;EIF2AK4;EIF2S1;RPTOR;VPS45;MLST8;GABARAPL1;ATG5;GABARAPL2;ATG16L1;NSF;GABARAP;ATG3;PIK3R4;SPTLC2;SPTLC3;NAPA;SPTLC1;MTMR7(up);VTI1A;LOC100856502;MAPK7;ULK2;RAB7A;BECN1;BECN2;ARPC3;ARPC2;VTI1B;ARPC5;ARPC4;ATG4C;ATG4B;ATG4A;ATG13;MTOR;YKT6;ATG4D;PIK3C3;IP6K3;IP6K2;IP6K1;KRAS;ATG10;PPP2CA;ARPC1B;ARPC1A;ARPC5L;ATG12;PRKACA;PRKACB |
| Carbon fixation in photosynthetic organisms | FBP2;GAPDH;LOC102152275;LOC482320;GPT;TPI1;ME1;MDH2;MDH1;LOC111096459;LOC102152592;TKTL1;TKT;RPIA;LOC111089999;LOC610338;GOT2;RPE;GOT1;PGK2;LOC119870407;PGK1;LOC100683724;SHPK;LOC111094513;ME3;LOC119866771;ALDOA;ALDOC;ALDOB;GPT2;LOC102154094;LOC477441;LOC119876526;FBP1(up);LOC106558345;LOC102151507;LOC102151775;LOC481849 |
| Cyanoamino acid metabolism | GGT1;GGT6;GGT7;GGT5;GBA3;SHMT2;SHMT1 |
| Pancreatic secretion | CEL;FXYD2;CTRL;ADCY1\_1;PNLIP;LOC607460;ADCY6;RYR2;PLA2G5;PLA2G3;SLC12A2;LOC475521;PLA2G2C(down);ATP1B4;ATP1B2;ATP1B3;ATP1B1;ADCY4(up);ATP2B1;ATP2B3;ATP2B4;ADCY5;RAB27B;ADCY7;ADCY1;ADCY2;ADCY3;ADCY8;CPB2;CPB1;BST1;CFTR;ATP2A1;ATP2A2;ADCY9;PLA2G1B;SLC9A1;CPA2;CPA3;PLA2G12A;CPA1;KCNMA1;RAB11A;SCT;LOC119869567;PLCB2;PLCB3;PLCB4;KCNQ1;SLC26A3;ITPR2;ITPR3;RHOA;ITPR1;PLA2G10;GNAQ;GNAS;SCTR;ATP2A3(down);RAP1B;RAP1A;SLC4A4;TRPC1;SLC4A2;CLCA2;CLCA1;CLCA4;RAB8A;PLCB1(up);PLA2G2E;PLA2G2D;PLA2G2F;CCK;TPCN2;CCKAR;ATP1A4;ATP1A3;ATP1A1;CD38(up);PRSS2;ATP2B2(up);PNLIPRP1;PNLIPRP2;RAC3;RAC1;PRKCA;PRKCB;CHRM3;PRKCG;RAB3D;LOC478196;PLA2G12B |
| Taste transduction | GNG13;KCNK5(down);HTR3A;LOC100856260;GABBR1;CAFA-T2R2;SCN9A;GABRA4;PKD2L1;TAS2R3;GABRA5;CALHM1;P2RX3;P2RX2;GABRA1;GNAT3;GABRA3;GABRA2;SCNN1A(down);PRKACB;GABRA6;PKD1L3;PDE1C;PDE1B;PDE1A;CACNA1C;LOC488101;CAFA-T2R12;SCNN1G;SCN2A;TAS2R40;SCNN1B;GRM4;TRPM5;LOC478649;GRM1;TAS2R38;TAS2R42;HTR1F;PLCB2;PLCB3;PLCB4;HTR1A;CHRM3;ENTPD2(down);TAS2R60;TAS2R10;GNB3;ASIC2;ADCY4(up);PLCB1(up);HTR1E;ITPR3;P2RY1;GABBR2(up);P2RY4;PRKACA;ADCY6;LOC100682759;TAS2R8;TAS2R9;TAS2R7;TAS2R4;TAS2R5;ADCY8;HCN4;TAS2R1;CAFA-T2R43;TAS2R41;CAFA-T2R67;HTR3B;HTR3C;HTR1D(down);CACNA1A;TAS1R1;TAS2R39;TAS1R3;TAS1R2;SCN3A;HTR1B |
| Apoptosis - fly | DNM1L;BIRC6;ATM;EGFR;PRDX2;CYCS;SAV1;LATS2;XIAP;BOK;MAPK10;RHOT1;ELF1;CASP8;ELF2;BTF3L4;HTRA2;TP63;JUN;MAP3K7;MAP3K5;MAPK3;MAPK1;ATR;LOC102157190;MAPK8;MAPK9;APAF1;TRAF4;TRAF6;LOC475353;LOC100689004;EIF5;STK3;ATF4;TSPO;DUSP10;KRAS;LMNB2;CASP7;LMNB1;BTF3;ELF4;TAB2;EIF2AK3;LATS1;LOC100683019;MFN2;BRAF;MAP2K7;AIFM1 |
| Fatty acid metabolism | HSD17B12;CPT2;HADHA;ACSL3;SCP2;CPT1C;ACADS;ACSBG1;ACSBG2;ECHS1;ACADL;ACADM;ACADVL;ACAA2;HADHB;SCD;PPT2;ACAT2;OXSM;TECR;HSD17B8;ACAT1;ACAA1;FASN;MECR;ACSL5(up);HSD17B4;CPT1A;LOC485024;ACSL1;ACACA;PPT1;ACSF3;CPT1B;SCD5;ACADSB;MCAT;FADS1;FADS2;EHHADH;LOC481043;HACD1;HADH;HACD3;HACD2;HACD4;ACOX3;ACOX1;ACSL6;ACSL4;ELOVL6;ELOVL7;ELOVL4;ELOVL5;ELOVL2;ELOVL3;ELOVL1 |
| Ferroptosis | LOC491817;SAT2;ACSL6;LPCAT3;ALOX15;VDAC3;VDAC2;GCLM;GCLC;TFRC;ATG7;PCBP1;LOC100684085;LOC100688200;LOC477071;LOC477072;ATG5;CP;FTL;LOC119870853;ACSL5(up);LOC611632;TP53;SLC40A1;SLC3A2;LOC484507;ACSL1;HMOX1;ACSL4;LOC102153158;LOC102154317;LOC487855;GPX4;ACSL3;STEAP3;LOC403631 |
| 2-Oxocarboxylic acid metabolism | IDH3A;ACO2;AADAT;NAGS;IDH3G;GPT2;IDH2;IDH1;ACO1;BCAT2;BCAT1;CS;IDH3B;GOT2;GPT;LOC476602;GOT1 |
| Monobactam biosynthesis | PAPSS1;PAPSS2 |
| Glycine, serine and threonine metabolism | GRHPR;GAMT;GNMT;BPGM;SDS;GLYCTK;AOC3;AGXT2;AOC2;PGAM2;DMGDH;PGAM1;CTH;CHDH(down);LOC485342;BHMT;MAOB;DAO(down);ALAS1;ALAS2;AMT;DLD;SRR;PHGDH;ALDH7A1;GCAT;SHMT2;SHMT1;SDSL;PIPOX;PSPH;AGXT;LOC477365;PSAT1;CBS;MAOA;SARDH;GLDC(down);GCSH |
| Longevity regulating pathway - worm | HSF1;UBL5;WASHC1;LOC479911;WASHC3;GSTA4;HSPA9;AKT1;AKT2;AKT3;LOC611366;PPP4R3A;PPP4R3B;LOC119867218;NFE2L2;PIK3CB;LOC479912;PIK3CD;LOC486404;GABARAP;LOC100855903;RNASEK;SOD2;SCD5;MGST1(down);LOC479459;PTEN;LOC609669;LOC481841;HIF1A;MGST2;MGST3;HSBP1;SCD;PARP4;FOXA2;MT1E;SIRT1;LOC119870217;LOC119870216;LOC119870215;PARP1;PARP2;PARP3;GSTO2;GSTO1;GSTT2B;LOC100856518;PIK3CA;TCERG1;MAPK14;MAPK10;MAPK11;MAPK12;MAPK13;LOC491791;LOC100686073;RPS6KB1;RPS6KB2;MAPK8;MAPK9;MTOR;IRS1;LOC102151236;LOC477556;LOC610304;LOC477558;LOC100686615;MT2A;CLPP;CAT;FOXO3;VHL;INSR;MAP2K6;YWHAZ;GABARAPL1;HCFC2;HCFC1;GABARAPL2;LOC476006;YWHAQ;GCLC;HSPD1;YWHAB;ULK2;LOC486776;KRIT1;LOC474938;GSTP1;FAR1;FAR2;ATF4 |
| Longevity regulating pathway - multiple species | HDAC1;HSPA2;LOC100856339;SOD1;HSP70;PRKAG1;PIK3CD;HRAS;ADCY1\_1;CRYAB;PRKAA2;INSR;ADCY9;FOXO3;AKT1;AKT2;AKT3;LOC607182;LOC111096471(down);CLPB;RPTOR;PIK3CA;PIK3CB;NRAS;RPS6KB1;RPS6KB2;AKT1S1;PIK3R2;PIK3R1;ATG5;HDAC2;PRKAG3;LOC119869567;IGF1R;IGF1;PRKAB2;LOC119873859;PRKAB1;LOC119870217;LOC119870216;LOC119870215;EIF4EBP2;FOXO1;ADCY4(up);MTOR;PRKAA1;HSPA8;CAT;KRAS;ADCY5;ADCY6;ADCY7;ADCY1;ADCY2;ADCY3;IRS4;ADCY8;IRS1;IRS2;INS;SOD2;FOXA2;PRKACA;PRKAG2;PRKACB;SIRT1;LOC474850 |
| Pathways of neurodegeneration - multiple diseases | LOC100682956;PPP3R2;PPP3R1;LOC102156968;NDUFAB1;DLG4;GABARAP;LOC119872525;GRIN1;ATP8;CAMK2D;CAMK2G;ATP6;CAMK2B;PIK3C3;COX6A2;TUBB4B;TUBB4A;LOC119870901;BAK1;LOC100688796;LOC102151506;EIF2AK3;LOC100685007;HSD17B10;ATP2A1;ATP2A2;RPS27A;FZD10;COX7B2;BCL2L1(down);TANK;LOC100684983;GRM5;GRM1;LOC100687152;LOC610636;PSMD14;PSMD11;PSMD13;PSMD12;GPX1;FRAT1;GPX3;GPX2;GPX5;GPX7;GPX6;GPX8;ATXN3;ATP2A3(down);LOC102153034(down);DCTN2;UBE2J2;DCTN1;DCTN6;HTRA2;DCTN5;NDUFV3;NDUFV2;NDUFV1;COX4I2;COX4I1;PSMD3;PSMD2;LOC106557778;LOC100687060;LOC100682717;LOC100687064;PSMD8;LOC100686510;BAD;LOC102151754;COX5A;COX5B;PSMD4;PSMD7;PSMD6;PSMD1;BAX;CAT;CTNNB1;PLCG1;CALML5;ATP5MC1;ATP5MC3;ATP5MC2;EIF2S1;LOC100686889;LOC100686569;PSMC1;PSMC2;PSMC3;PSMC4;PSMC5;PSMC6;NOS1;NDUFC2;CAMK2A;APC2;LOC102154372;SEM1;ATP5F1A;HAP1;DKK4;DKK2;DKK1;FAS;PPP3CA;PPP3CB;PPP3CC;LOC119866377;LOC100856399;CAPN1;ARAF;LOC100685227;LOC100688835;WNT10A;WNT10B;NDUFA10;NDUFA11;NDUFA12;NDUFA13;RELA;RAB5A;APP;CSF1;UBA52;APC;UQCRFS1;WNT7A;UQCRC1;NDUFC1;LOC609798;LOC106557930;DERL1;LOC102152785;FZD3;COX7A1;TUBB2A;TBK1;ATP5F1D;ATP5F1E;TUBB;ATP5F1B;ATP5F1C;LOC100684996;LOC102155658;WNT3A;TRPC3;PSEN2;PSEN1;LOC119874008;LOC102154615;SQSTM1;MTOR;LOC100683076;LOC491231;MFN2;ND4L;LOC119870103;CDK5;LOC477570;UQCR10;UQCR11;PRKN;IL1A(up);LOC100688724;GABARAPL1;AXIN2;AXIN1;GABARAPL2;CYTB;DVL2;DVL3;DVL1;NDUFS5;LOC487689;WNT9A;RAC1;PRKCA;CHRM5;PRKCB;CHRM3;PRKCG;VCP;TUBA4A;LOC100683681;LOC102156642;SLC6A3;LOC100683828;UBE2J1;NDUFB9;NDUFB8;NDUFB7;NDUFB6;NDUFB5;NDUFB4;NDUFB3;NDUFB2;NDUFB1;UQCRQ;UQCRH;CALM2;CALM3;CALM1;LOC100687556;CACNA1B;CACNA1C;CACNA1D;CACNA1F;MAP3K5;LOC609990;CACNA1S;SLC25A6;SLC25A5;SLC25A4;LOC100685720;KIF5A;KIF5B;KIF5C;LOC100855914;GRIA2;GRIA3;HRAS;GRIA1;GRIA4;BECN1;DAXX;LOC610725;PLCB2;PLCB3;PLCB4;BECN2;TUBB6;TUBB3;TUBB1;UBA7;ATG13;ATG14;UBA1;ATP5PO;TNFRSF1B;GNAQ;TNFRSF1A;PSMB2;STX1A;VDAC3;VDAC2;IL1B;RAF1;CALML4;PSMA2;PSMA3;PSMA1;PSMA6;PSMA7;PSMA4;PSMA5;ND1;ND3;ND2;ND5;ND4;ERN1;ND6;NDUFA6;NDUFA7;NOS2(up);NDUFA5;NDUFA2;NDUFA3;NDUFA1;FADD;NDUFA8;NDUFA9;LOC100687259;CSNK1A1;LRP6;LRP5;WNT3;WNT2;WNT1;WNT6;WNT4;IL6;PPIF;CSNK2B;LOC100686830;ATG101;CYCS;MAP2K3;MAP2K2;MAP2K1;MAP2K7;MAP2K6;RB1CC1;LOC106557476;LOC119870484;COX2;COX3;COX1;WNT2B;CASP7;CASP3;CYC1;CASP8;CASP9;NDUFS1;NDUFS2;NDUFS3;NDUFS4;WNT9B;MAPT;NDUFS7;NDUFS8;BID;BCL2;PTGS2;LOC102155410;CHMP2B;LOC610188;CALML6;BDNF;NDUFA4L2(down);RYR1;RYR3;RYR2;NDUFB11;NDUFB10;CSNK1E;CAPN2;COX8A;DDIT3;SOD1;LOC612644;LOC474547;FASLG;CSNK2A2;CSNK2A1;KRAS;CHRNA7(down);GSK3B;LOC119864666;UBC;LOC478702;UBE2L6;SLC25A31;UBE2L3;VDAC1;RAC3;TUBA1C;LOC477508;PIK3R4;DCTN4;LOC100855928;CHRM1;ITPR2;ITPR3;LOC111096466;ITPR1;LOC100686432;TUBAL3;UQCRC2;LOC119863903;LOC119863906;WNT5B;WNT5A;WNT7B;TUBA8;LOC102154025;MAPK14;MAPK10;MAPK11;MAPK12;MAPK13;LOC102153059;LOC100684842;FZD1;FZD2;RAB8A;FZD4;FZD6;FZD7;FZD8;FZD9;LOC100686500;MAPK3;MAPK1;NFKB1;MAPK8;MAPK9;APAF1;NDUFS6;PLCB1(up);LOC100684768;PSMA8;PSMB7;PSMB6;PSMB5;PSMB4;PSMB3;LOC102154590;PSMB1;NDUFA4;LOC119865581;XBP1;NRAS;LOC608051;WNT8A;WNT8B;UBE2G2;UBE2G1;HSPA5;ADRM1;ULK2;TRAF2;LOC100689004;TNF;ATF4;COX7A2L;SDHB;LOC611471;GRIN2B;GRIN2C;GRIN2A;BRAF;GRIN2D;SDHA;WNT16;SDHC;ATF6;SDHD;WNT11 |
| Steroid biosynthesis | MSMO1;SOAT2;LOC102154725;TM7SF2;NSDHL;DHCR7;SC5D;SOAT1;LOC102153215;LOC100685679;SQLE;HSD17B7;CEL;CYP27B1;CYP51A1;FDFT1;LSS;LIPA;CYP2R1;DHCR24;CYP24A1(up);EBP;LOC119868057 |
| Proximal tubule bicarbonate reclamation | PCK2;PCK1;FXYD2;SLC38A3(down);MDH1;CA4(up);ATP1B4;SLC9A3;ATP1B2;ATP1B3;GLUD1;ATP1B1;SLC4A4;ATP1A4;ATP1A3;ATP1A1;AQP1;GLS;GLS2;SLC25A10 |
| TNF signaling pathway | PTGS2;LIF;LOC100856339;IKBKB;AKT1;AKT2;AKT3;MAP3K8;PIK3CA;PIK3CB;PIK3CD;LOC119874472(up);MAP3K5;FADD;RELA;CREB5;CREB3;CREB1;CASP10;SOCS3;CSF1;CSF2;CCL2;CCL5;NFKBIA;JUN;PIK3R2;PIK3R1;CX3CL1;CCL20;FOS;LOC119870217;LOC119870216;LOC119870215;CHUK;VEGFD;VEGFC;IRF1;TNFRSF1B;NOD2(up);TNFAIP3;MMP14;BAG4;MAP3K7;MAPK14;PGAM5;MAPK10;MAPK11;MAPK12;MAPK13;IL1B;CFLAR;TRADD;IFNB1;MAPK3;MAPK1;ICAM1;NFKB1;MAPK8;MAPK9;DAB2IP;PHF19;LTA;VCAM1;RPS6KA4;CXCL10(down);RPS6KA5(up);RIPK1;LOC119876918(up);TNFRSF1A;IL15;IL6;TAB2;ITCH;IFGGC1(down);JUNB;BIRC3;BIRC2;MAP2K3;MAP2K1;MAP2K7;MAP2K6;MAP2K4;CEBPB;FAS;EDN1(down);JAG1;TRAF2;TRAF3;TRAF5;IL18R1;TNF;DNM1L;RIPK3;CASP7;CASP3;TAB3;SELE;TAB1;LOC106557449;CASP8;CREB3L2;CREB3L3;CREB3L1;MAP3K14;CREB3L4;MMP9;ATF4;ATF6B;MMP3;BCL3;ATF2 |
| Toll and Imd signaling pathway | FBXW11;MAPK12;DUOX1;LOC607817;NFKBIA;BIRC3;UBE2D3;UBE2D1;IKBKB;MAP2K7;MAPK11;REL;MAPK13;MAPK14;MAPK9;JUN;MAP3K7;MAP3K4;LOC119863875;FADD;NFKB1;MAPK8;MYD88;IRAK4;DUOX2;LOC119868036;LOC100684252;FAF1;UBE2D2;LOC610206;BIRC2;MAP2K3;UBE2V1;UBE2V2;LOC106558148;ANK1(up);IRAK1;UBE2N;TAB2;BTRC;CASP8;ANK2(up);ANK3;LOC111094434(up);MAPK10;ATF2 |
| Bacterial secretion system | OXA1L;SRP54 |
| Biotin metabolism | BTD;HLCS;OXSM |
| Melanogenesis | ADCY1\_1;CALM2;CALM3;CALM1;WNT10A;WNT10B;EDN1(down);DCT;MC1R;CAMK2D;CAMK2G;CREB3;CAMK2A;CAMK2B;ADCY4(up);KITLG;KRAS;ADCY5;ADCY6;ADCY7;ADCY1;ADCY2;ADCY3;ADCY8;TCF7L1;TCF7L2;KIT;WNT7A;TYR;WNT7B;GNAO1;HRAS;PLCB2;ADCY9;EDNRB;GSK3B;RAF1;LOC119869567;TCF7;PLCB3;PLCB4;GNAI1(up);GNAQ;GNAS;CALML6;CALML5;CALML4;LOC119863906;WNT5B;WNT5A;CREBBP;WNT3A;LOC102153034(down);GNAI2;GNAI3;FZD1;FZD2;FZD3;FZD4;FZD6;FZD7;FZD8;FZD9;MAPK3;MAPK1;WNT8A;PLCB1(up);POMC;LEF1;EP300;WNT3;WNT2;WNT1;WNT6;WNT4;PRKACA;PRKACB;TYRP1;CTNNB1;MAP2K2;MAP2K1;MITF;FZD10;NRAS;DVL2;DVL3;WNT8B;DVL1;ASIP;WNT2B;WNT9A;PRKCA;PRKCB;PRKCG;CREB1;CREB3L2;CREB3L3;CREB3L1;WNT9B;CREB3L4;WNT16;WNT11 |
| Fanconi anemia pathway | FAAP100;USP1;ERCC4;MUS81;ATR;FANCD2;POLN;BRIP1;POLK;RMI1;RMI2;POLH;SLX4;FANCF;WDR48;CENPS;MLH1;FANCG;ATRIP;FANCE;FANCC;FANCB;FANCA;ERCC1;FANCM;FANCL;FAAP24;FANCI;BRCA1;BRCA2;LOC483061;BLM;POLI;REV3L;CENPX;REV1;TELO2;PMS2;RAD51C;LOC100683112;FAN1;EME1;PALB2;RPA1;RPA3;RPA2;SLX1A;RAD51;HES1;EME2;TOP3A;UBE2T;TOP3B |
| Lipoic acid metabolism | LIPT2;LIAS |
| Phenylpropanoid biosynthesis | PRDX6;GBA3 |
| Vasopressin-regulated water reabsorption | LOC611471;DYNLL1;DYNLL2;STX4;DCTN2;RAB5A;DCTN1;DCTN6;DCTN4;DCTN5;DYNC1I1;DYNC1I2;NSF;RAB11A;ARHGDIG;RAB11B;ARHGDIB;ARHGDIA;DYNC2LI1;AQP4;AQP2;AQP3;RAB5C;RAB5B;CREB5;CREB3;CREB1;LOC474547;DYNC1LI1;DYNC1LI2;DYNC1H1;DYNC2H1;ADCY6;VAMP2;ADCY3;AVPR2;GNAS;AVP;ADCY9;CREB3L2;CREB3L3;CREB3L1;PRKACA;CREB3L4;PRKACB;LOC100856399 |
| Apelin signaling pathway | MYL4;CCN2;PRKAG1;PRKAG3;PRKAG2;AKT1;AKT2;AKT3;MYL2;MYL3;LOC488190;PLIN1;CALM2;CALM3;CALM1;GNG14;RYR3;RYR2;GNG10;GNG11;GNG12;GNG13;LOC119863873;CDH1;ADCY1\_1;LIPE;ADCY4(up);PIK3C3;KRAS;ADCY5;ADCY6;ADCY7;ADCY1;ADCY2;ADCY3;CCND1;ADCY8;MYLK;GNG7;GNG4;GNG5;GNG2;GNG3;GNG8;ACTA2;HRAS;RRAS2;ADCY9;RRAS;PRKAB2;SERPINE1;SLC9A1;RAF1;LOC100855681;BECN2;NOS2(up);PPARGC1A;PIK3R4;SPP1;LOC119869567;PLCB2;PLCB3;PLCB4;PRKAB1;LOC119870217;LOC119870216;LOC119870215;MRAS;GNAI1(up);LOC489647;ITPR2;ITPR3;ITPR1;APLNR;GNAQ;CALML6;CALML5;CALML4;MEF2C;RYR1;MEF2A;LOC100686736;MEF2D;AGTR1;APLN;SMAD4;GNB4(up);LOC102153034(down);SMAD2;SMAD3;RPS6;ADCY10;PDE3B;GNAI2;GNAI3;RPS6KB1;RPS6KB2;MAPK3;MAPK1;SLC8A1;SLC8A2;TGFBR1;GNB5;GABARAP;GNB1;MYLK3;GNB3;GNB2;PLCB1(up);MTOR;LOC491854;CAMK4;PRKACA;PRKACB;LOC106559872;SPHK2;SPHK1;PRKAA2;PRKAA1;MAP2K2;MAP2K1;SLC8A3(up);GABARAPL1;MEF2B;NRAS;KLF2;JAG1;GNA13;NOS1;NOS3;MYLK2;PRKCE;PLAT;UCP1;BECN1;LOC608772;GABARAPL2;GNGT2;GNGT1 |
| VEGF signaling pathway | SPHK2;SPHK1;LOC100856339;PPP3R2;PPP3R1;PTGS2;RAF1;HRAS;NFATC2;MAP2K1;MAPK14;PLCG2;PLCG1;AKT3;MAPK11;MAPK12;MAPK13;MAPKAPK3;MAPKAPK2;MAPK3;PIK3CA;PIK3CB;NRAS;PIK3CD;PRKCA;MAPK1;PIK3R2;PIK3R1;KRAS;PTK2;KDR;PXN;SRC;NOS3;RAC3;SHC2;HSPB1;LOC119870217;LOC119870216;LOC119870215;PRKCB;PLA2G4E(up);SH2D2A;VEGFA;PRKCG;PLA2G4B;PLA2G4A;PLA2G4F;LOC119866928;PLA2G4D;RAC2(up);AKT1;AKT2;BAD;CASP9;MAP2K2;RAC1;PPP3CA;PPP3CB;PPP3CC;CDC42 |
| Flavone and flavonol biosynthesis | GUSB |
| Biosynthesis of unsaturated fatty acids | HSD17B12;SCP2;SCD;TECR;ACAA1;HSD17B4;ACOT4(up);SCD5;FADS1;ACOT7;FADS2;LOC490770;HACD1;HACD3;HACD2;HACD4;ACOX3;ACOX1;BAAT;ELOVL6;ELOVL7;ELOVL4;ELOVL5;ELOVL2;ELOVL3;ELOVL1 |
| Cytosolic DNA-sensing pathway | POLR3F;CCL5;NFKBIB;NFKBIA;POLR3K;RIPK1;POLR3G;RIPK3;POLR1C;POLR3B;POLR3C;CGAS;IKBKE;IL1B;LOC611406;POLR3H;DDX58;CCL4;TBK1;ZBP1;ADAR;TREX1;IFNB1;POLR2K;NFKB1;RELA;IRF3;POLR2H;CHUK;POLR3GL;POLR2E;POLR2F;IL33;PYCARD;POLR3A;POLR2L;LOC119876828;CXCL10(down);IL18;IKBKB;LOC100686162;LOC119870926;IRF7;LOC100683567;IFNA7;POLR3D;IFNA5;IL6;POLR3E;STING1;POLR1D;MAVS |
| Growth hormone synthesis, secretion and action | LOC100856339;GHR;ADCY1\_1;AKT1;AKT2;AKT3;CRKL;PIK3CA;PIK3CB;CACNA1C;CACNA1D;CACNA1F;CACNA1S;SHC4;SHC1;SHC2;SHC3;CREB5;CREB3;CREB1;ADCY4(up);KRAS;ADCY5;ADCY6;ADCY7;SOCS1;ADCY1;SOCS3;ADCY3;SOCS2;ADCY8;ADCY9;PLCG1;GSK3B;MAP3K1;GRB2;GHRHR;ADCY10;HRAS;PLCB3;CRK;ADCY2;GNAI2;SSTR3;JAK2;PIK3R2;PIK3R1;SSTR5;LOC119869567;PLCB2;IGF1;PLCB4;FOS;LOC119870217;LOC119870216;LOC119870215;GNAI1(up);IGFBP3;ITPR2;ITPR3;ITPR1;BCAR1;GNAQ;GNAS;SST;CREBBP;GHRH;GHRL;PIK3CD;MAPK14;MAPK10;MAPK11;LOC102154108;MAPK13;RAF1;GNAI3;STAT3;STAT1;MAPK3;MAPK1;MAPK8;MAPK9;PLCB1(up);MTOR;GHSR;EP300;SOS2;MAP2K2;IRS4;IRS1;IRS2;GH1;SSTR1;STAT5B;STAT5A;SSTR2;PRKACA;PRKACB;PTK2;JUNB;MAP2K3;PLCG2;MAP2K1;MAP2K6;MAP2K4;LOC111096471(down);NRAS;PRKCA;PRKCB;PRKCG;MAPK12;CREB3L2;CREB3L3;CREB3L1;CREB3L4;GNA11;ATF4;ATF6B;ATF2 |
| D-Glutamine and D-glutamate metabolism | GLS;GLS2;GLUD1 |
| D-Arginine and D-ornithine metabolism | DAO(down) |
| Fc epsilon RI signaling pathway | PLCG1;LOC100856339;LOC119879788;VAV2;PIK3CD;LOC102155828;MAP2K4;GAB2;MAP2K1;MAPK14;LOC607467;LOC102154760;AKT3;MAP2K7;MAPK11;MAPK12;MAPK13;MAPK10;IL5;MS4A2;PLA2G4A;FCER1G;FCER1A;ALOX5;LOC102153980;PIK3CA;PIK3CB;NRAS;FYN;PRKCA;LOC106559121;MAPK1;PIK3R2;PIK3R1;LYN;LOC106559212;MAPK3;PLA2G4B;RAC3;RAC1;LOC119870217;LOC119870216;LOC119870215;MAPK8;ALOX5AP;LOC111097181;PLA2G4E(up);PLCG2;TNF;LOC119869046;LCP2;KRAS;PLA2G4F;RAF1;LOC119869025;MAP2K3;HRAS;MAPK9;INPP5D;SOS2;AKT1;SYK;VAV3;IL13;AKT2;CSF2;LOC106559111;MAP2K2;VAV1;IL4;GRB2;IL3;PLA2G4D;LAT;PDPK1;RAC2(up);LOC102155967;MAP2K6 |
| Fc gamma R-mediated phagocytosis | LOC100856339;CRKL;LOC607467;AKT2;AKT3;FCGR1A;GSN;LOC102154266;PIK3CA;PIK3CB;PIK3CD;PLA2G6;LOC106559121;MARCKS;LYN;PLA2G4E(up);LOC119879788;LOC106559212;INPP5D;PLD1;PLD2;AKT1;SYK;ARPC1B;ARPC1A;PLCG1;LOC106559111;LAT;PAK1;DOCK1;GAB2;CRK;LOC100687971;LOC119870217;PIK3R2;PIK3R1;ARPC2;ARPC3;LOC119870216;LOC119870215;ARPC5;ARPC4;PRKCG;HCK;AMPH;RAC2(up);VAV1;VAV3;VAV2;LOC478984;CDC42;NCF1;CFL1;SPHK2;FCGR2B;MYO10;RAF1;PLA2G4A;LOC102153980;RPS6KB1;ARF6;RPS6KB2;MAPK3;MAPK1;MARCKSL1;PLPP3;PLPP2;LOC111097181;WAS;LOC119866928;LOC119869025;PIP5K1C;PIP5K1B;PIP5K1A;ARPC5L;LOC102154760;ASAP1;ASAP2;ASAP3;PLPP1;SPHK1;LIMK2;LIMK1;LOC102152609;PLCG2;MAP2K1;WASL;BIN1;WASF1;INPPL1;WASF3;RAC3;RAC1;PRKCA;PRKCB;PRKCE;PRKCD;LOC119869994;CFL2;LOC119869046;PLA2G4B;VASP;PLA2G4F;PLA2G4D;WASF2;PTPRC;SCIN |
| Phosphatidylinositol signaling system | LOC100856339;IPMK;PI4K2B;PI4K2A;CALM2;CALM3;CALM1;PIK3CA;PIK3CB;PIK3CD;INPP4B;INPP4A;MTMR6;MTMR4;PIP4K2A;MTMR2;MTMR1;CDS1;MTM1;IMPA1;PIK3C2A;PIK3C2G;PIK3C3;IP6K3;IP6K2;IP6K1;INPP5A;INPP5B;INPP5D;INPP5E;INPP5J;INPP5K;PPIP5K2;PTEN;PPIP5K1;PIP5K1B;CDS2;ITPKA;ITPKC;ITPKB;PIK3R2;PIK3R1;ITPK1;PLCB2;PLCB3;PLCB4;PIKFYVE;ITPR2;ITPR3;ITPR1;CALML6;CALML5;CALML4;PI4KB;PI4KA;PLCZ1;LOC102153034(down);PLCE1;CDIPT;MTMR7(up);MTMR14;PLCB1(up);PLCD4;PLCD3;PLCD1;PIP5K1C;MTMR3;PIP5K1A;BPNT2;PIP4K2C;PIP4K2B;OCRL;PLCG2;PLCG1;DGKZ;INPPL1;DGKQ;DGKK;DGKH;DGKI;SYNJ1;SYNJ2;DGKB;DGKA;DGKG;DGKD;DGKE;PRKCA;PRKCB;PRKCG;IPPK;INPP1;PIK3C2B;PIP4P2;PIP4P1;IMPA2 |
| Sphingolipid signaling pathway | LOC100856339;PPP2R3B;PPP2R3C;LOC102155828;PPP2R3A;AKT1;AKT2;AKT3;MS4A2;CERS6;CERS4;CERS5;CERS2;CERS1;PIK3CA;PIK3CB;PIK3CD;MAP3K5;SPTLC2;SPTLC3;SPTLC1;RELA;PPP2R2D;CTSD;PPP2R2C;KRAS;PLD1;PLD2;PPP2CA;PPP2CB;PTEN;MAPK10;TRADD;ADORA1;HRAS;MAPK13;GAB2;SGPP2(down);RAF1;PIK3R2;PIK3R1;PLCB2;PLCB3;PLCB4;LOC119870217;LOC119870216;LOC119870215;SGPL1;GNAI1(up);RHOA;TP53;GNAQ;TNFRSF1A;PRKCZ;PPP2R5D;PPP2R5E;PPP2R5A;PPP2R5B;PPP2R5C;PPP2R1A;PPP2R1B;MAPK14;SMPD1;MAPK11;MAPK12;SMPD2;NSMAF;GNAI2;GNAI3;FCER1G;FCER1A;S1PR3;S1PR2;S1PR5;S1PR4;MAPK3;MAPK1;NFKB1;MAPK8;MAPK9;ADORA3;SGPP1;CERS3(up);FYN;PLCB1(up);RAC2(up);DEGS1;DEGS2;BDKRB2;KNG1;SPHK2;SPHK1;BAX;MAP2K2;MAP2K1;SGMS2;SGMS1;ACER2;ACER1;ASAH1;ASAH2;BID;NRAS;OPRD1;TRAF2;NOS3;RAC3;RAC1;PRKCA;PPP2R2A;PRKCB;PRKCE;PRKCG;TNF;ROCK1;ROCK2;LOC119864605;GNA12;GNA13;PDPK1;BCL2;LOC102155967;ABCC1;PPP2R2B(up) |
| Phospholipase D signaling pathway | AVPR1B;LOC100856339;FYN;LOC102155828;ADCY1\_1;PTGFR;RAPGEF4;AKT1;AKT2;AKT3;RAPGEF3;AGT;MS4A2;EGF;DGKK;PIK3CA;PIK3CB;PIK3CD;DGKH;AVPR1A;LOC106559121;LOC106559212;F2(down);SHC4;SHC1;SHC2;SHC3;PLA2G4E(up);ADCY4(up);LOC119879788;KITLG;KRAS;ADCY5;ADCY6;ADCY7;PLD1;PLD2;ADCY3;SYK;AVPR2;ADCY8;ADCY9;PLCG1;KIT;F2R;GRB2;PTPN11;ADCY1;CXCL8(up);HRAS;RRAS2;GAB2;GAB1;RRAS;DNM1;DNM3;LOC106559111;ADCY2;TSC2;PTK2B;GRM8;PIK3R2;PIK3R1;LOC119870216;GRM4;GRM5;GRM6;GRM7;GRM1;GRM2;GRM3;LOC119869567;PLCB2;PLCB3;PLCB4;LOC119870217;LOC100683481;LOC119870215;MRAS;RHOA;GNAS;AVP;INS;AGTR1;RALB;RALA;PDGFRB;PDGFRA;LOC607467;RAF1;PLA2G4A;TSC1;FCER1G;FCER1A;LOC102153980;ARF1;ARF6;PLA2G4F;MAPK3;MAPK1;PLPP1;PLPP3;PLPP2;RALGDS;LOC111097181;PLCB1(up);MTOR;LOC119869025;SOS2;PLCG2;PIP5K1C;PIP5K1B;PIP5K1A;MAP2K1;AGPAT5;AGPAT4;AGPAT3;AGPAT2;AGPAT1;LPAR2;LPAR3;RHEB;LPAR6;LPAR4;LPAR5;SPHK2;SPHK1;EGFR;MAP2K2;INSR;DGKZ;DGKQ;NRAS;CXCR2;CXCR1;DGKI;DGKB;DGKA;DGKG;DGKD;DGKE;PDGFA;PDGFB;PDGFC;PDGFD;PRKCA;LPAR1(up);LOC119869046;CYTH1;PLA2G4B;CYTH3;CYTH2;CYTH4;PLA2G4D;GNA12;GNA13;LOC102155967;LOC102154760 |
| B cell receptor signaling pathway | RASGRP3;NFATC2;LOC100856339;PPP3R2;PPP3R1;NFATC1;NFKBIB;JUN;HRAS;NFKBIA;MAP2K1;NFKBIE;IKBKB;AKT1;AKT2;AKT3;LOC106559212;LOC607467;LOC490269;CD79B;PIK3AP1;CD79A;CD72;INPPL1;LOC102153980;CD81;PIK3CA;PIK3CB;NRAS;PIK3CD;PTPN6;LOC484309;LOC106559121;MAPK1;NFATC3;PIK3R2;PIK3R1;CD22;NFKB1;RELA;LOC106559111;LOC119870215;MALT1;BLNK;LOC119869025;LYN;RAC3;FOS;LOC119870217;LOC119870216;CARD11;PRKCB;FCGR2B;CHUK;LOC111097181;LOC119879788;LOC119869046;RAC2(up);BCL10;RAF1;KRAS;MAPK3;DAPP1(up);INPP5D;SOS2;PLCG2;VAV1;VAV3;VAV2;LOC102154760;GSK3B;MAP2K2;SYK;GRB2;LOC484306;RAC1;LOC611446;PPP3CA;PPP3CB;PPP3CC;CD19 |
| Chloroalkane and chloroalkene degradation | ALDH1B1;EPHX2;ALDH2;ADH5 |
| Fatty acid degradation | ACADVL;CPT2;ACADS;LOC119867230;ACAA2;LOC100856533;ACSBG1;ACSBG2;ACADL;ACADM;GCDH;ECHS1;HADHB;HADHA;ACAT2;CPT1C;ACAT1;ACAA1;CYP2U1;ACOX1;ACSL5(up);CPT1A;LOC485024;ACSL1;ALDH2;CPT1B;ALDH9A1;ACADSB;ALDH7A1;ALDH1B1;EHHADH;LOC481043;HADH;ADH5;ADH4;ACSL3;CYP4A11(down);ACSL6;ACSL4;ECI2;ECI1;ACOX3;CYP4A38(down) |
| Synthesis and degradation of ketone bodies | HMGCS1;HMGCS2;HMGCL;ACAT1;ACAT2;OXCT1;HMGCLL1;BDH2;BDH1 |
| Cutin, suberine and wax biosynthesis | FAR1;FAR2 |
| Pyruvate metabolism | PCK2;GRHPR;PCK1;ME3;ALDH7A1;LDHAL6B;LOC102152275;ME1;MDH2;MDH1;ME2;FH;LOC100686034;PKLR;ACAT1;HAGH;PC;ACAT2;LDHD;GLO1;PDHB;LDHA;LDHB;PKM;PDHA2;ACACA;DLD;ACSS2;ACSS1;LOC609911;LOC102153001;ACACB(down);DLAT;ALDH9A1;LOC119866771;LOC490690;ALDH1B1;LOC100684709;ACOT12;ACYP1;ACYP2;ALDH2;LOC102151507;LDHC |
| Toluene degradation | CMBL |
| Regulation of lipolysis in adipocytes | ADRB2(down);PTGS1;LOC100856339;AQP7;ADORA1;TSHB;ADCY1\_1;MGLL;IRS1;AKT1;INSR;AKT3;TSHR;LOC111096471(down);PDE3B;GNAI2;GNAI3;PLIN1;CGA;PIK3CA;PIK3CB;PIK3CD;PTGER3;PNPLA2;NPY1R;PRKG1;PIK3R2;PIK3R1;PRKG2;NPY;NPPA;LOC119869567;NPR1;LIPE;LOC119870217;LOC119870216;LOC119870215;PLAAT3(down);GNAI1(up);ADCY4(up);AKT2;PTGS2;ABHD5;ADCY5;ADCY6;ADCY7;ADCY1;ADCY2;ADCY3;IRS4;GNAS;ADCY8;ADCY9;IRS2;INS;ADRB1;ADRB3;PRKACA;PRKACB;FABP4 |
| Human cytomegalovirus infection | TAP1;PTGS2;LOC100856339;PPP3R2;DLA-64;ADCY1\_1;IKBKB;AKT1;AKT2;AKT3;CGAS;B2M;CALM2;PPP3R1;CALM1;GNG14;PIK3CA;PIK3CB;PIK3CD;GNG11;GNG12;GNG13;LOC119863873;FADD;RELA;CDKN2B;ARHGEF11;ARHGEF12;DLA-79;SP1;CREB5;CREB3;CREB1;ADCY4(up);LOC480600;FASLG;LOC611406;MDM2;CXCL12;KRAS;ADCY5;ADCY6;ADCY7;ADCY1;ADCY2;ADCY3;CCND1;GNB4(up);ADCY9;BAK1;GNG7;GNG4;GNG5;GNG2;GNG3;GNG8;CCR5;CCL2;NFATC1;NFATC2;NFATC3;NFATC4;IL10RA;CCL4;CCL5;CXCL8(up);HRAS;NFKBIA;TRAF2;ELK1;CRK;GSK3B;IL1R1;TRAF5;TAP2;TAPBP;RAF1;LOC100855681;CRKL;PTK2B;GRB2;PIK3R2;PIK3R1;JAK1;CX3CL1;CDK4;LOC119876828;LOC119869567;PLCB2;PLCB3;PLCB4;LOC119870217;LOC119870216;LOC119870215;DLA88;CHUK;GNAI1(up);VEGFA;PRKCG;ITPR2;ITPR3;RHOA;ITPR1;BCAR1;CALM3;IRF3;LOC100686162;RAC2(up);GNAQ;TNFRSF1A;LOC100683567;CREB3L4;CALML6;ITGAV;CALML4;PTGER4;EIF4EBP1;STING1;PDGFRA;GNAI2;CCL3;GNG10;LOC102153034(down);PTGER1;MAPK14;CCR1;CCR3;PTGER2;MAPK12;MAPK13;ARHGEF1;IL1B;PTGER3;TSC2;GNAI3;TSC1;STAT3;IL10RB;TBK1;RPS6KB1;TRADD;RPS6KB2;MAPK3;MAPK1;GNAO1;NFKB1;CDKN1A;SRC;GNB5;GNB1;GNB3;GNB2;PLCB1(up);MTOR;CYCS;TP53;LOC612475;MYC;E2F2;RIPK1;SOS2;GNAS;IFNA7;IFNA5;IL6;IFNB1;PRKACA;CDK6;PRKACB;RHEB;MAP2K6;PTK2;ROCK2;EGFR;BAX;CTNNB1;MAP2K2;MAP2K1;CALML5;ADCY8;FAS;BID;NRAS;RB1;CXCR2;MAPK11;PXN;GNA13;ITGB3;RAC3;RAC1;PRKCA;PRKCB;PDIA3;LOC100689004;TNF;ATF4;IL6R;ATF6B;E2F3;CASP3;E2F1;CALR;LOC119863891(up);ROCK1;CASP8;CASP9;CREB3L2;CREB3L3;CREB3L1;GNA12;GNGT2;GNGT1;GNA11;PPP3CA;PPP3CB;PPP3CC;ATF2 |
| T cell receptor signaling pathway | LOC100856339;PPP3R2;PPP3R1;FYN;IKBKB;AKT1;AKT2;AKT3;DLG1;MAP3K8;PIK3CA;PIK3CB;PIK3CD;LOC102156776;RELA;LOC102156003;CD28;IFNG;KRAS;MAPK14;CSF2;GSK3B;GRB2;PAK4;PAK5;PAK6;CD3D;CD3E;PAK2;CD3G;NFATC1;NFATC2;NFATC3;NFKBIB;HRAS;NFKBIA;NFKBIE;JUN;LOC102156901;GRAP2(up);PIK3R2;PIK3R1;CDK4;PAK3(up);CTLA4;FOS;LOC119870217;LOC119870216;LOC119870215;CHUK;LOC119872979;LAT;LOC111090226;RHOA;PTPN6;VAV1;VAV3;VAV2;PAK1;LCK;CD40LG;MAP3K7;CD4;RASGRP1;MAPK10;MAPK11;MAPK12;SOS2;RAF1;LOC106559087;TEC;MAPK3;MAPK1;NFKB1;MAPK8;MAPK9;PDCD1;ICOS;CD8A;LOC612475;CD8B;LOC119866928;ITK;IL10;MAP2K1;IL4;IL5;IL2;CDC42;LOC119869603;LOC119869607;MAP2K2;PLCG1;CD247;MAP2K7;NRAS;ZAP70;LOC609053;PTPRC;MALT1;CARD11;TNF;PDPK1;PRKCQ;LCP2;BCL10;NCK1;NCK2;LOC480788;MAPK13;MAP3K14;PPP3CA;PPP3CB;PPP3CC |
| Adrenergic signaling in cardiomyocytes | LOC491979;KCNE1;FXYD2;PPP2R3B;PPP2R3C;ADCY1\_1;RAPGEF4;AKT1;MYL4;AKT3;MYL2;TNNC1;RAPGEF3;AGT;CALM2;CALM3;CALM1;PPP2R2A;CACNA1C;CACNA1D;CACNA1F;CAMK2B;CACNA1S;PPP2R3A;CAMK2D;PPP2R2D;ATP1B4;CAMK2G;CREB3;ATP1B3;CREB1;ATP1B1;ADCY4(up);ADRA1B;CACNA2D2;CACNA2D3;ATP2B3;CACNA2D1;ATP2B4;CACNA2D4;ADRA1D;ADRA1A;ADCY5;ADCY6;ADCY7;ADCY1;ADCY2;ADCY3;PPP2CA;PPP2CB;ADCY8;AKT2;MYL3;ATP2A1;ATP2A2;ADCY9;BCL2;CACNB2(up);PPP1R1A;TNNT2;SLC9A1;LOC489252;ATP1A3;LOC119869567;PLCB2;PLCB3;PLCB4;CACNG7;LOC119870217;LOC119870216;LOC119870215;GNAI1(up);ATP2B2(up);GNAQ;GNAS;CALML6;CALML5;CALML4;CREM;PPP2R5D;PPP2R5E;AGTR1;SCN5A;PPP2R5A;PPP2R5B;PPP2R5C;SCN7A;RYR2;ATP2A3(down);PPP2R1A;ACTC1;LOC102153034(down);MAPK14;MAPK11;MAPK12;MAPK13;AGTR2;GNAI2;GNAI3;MYH7;CACNB3;CACNB1;TPM4;TPM3;TPM2;TPM1;CACNB4;MAPK3;MAPK1;SLC8A1;SLC8A2;PLCB1(up);RPS6KA5(up);ATP2B1;SCN4B;ADRB1;ATP1A4;PRKACA;ATP1A1;PRKACB;PLN;ADRB2(down);PPP2R1B;SLC8A3(up);TNNI3;PPP2R2B(up);CREB5;PRKCA;ATP1B2;KCNQ1;CAMK2A;PPP2R2C;CACNG1;CACNG2;PPP1CB;PPP1CC;PPP1CA;CREB3L2;CREB3L3;CREB3L1;SCN1B;CREB3L4;ATF4;ATF6B;CACNG3;CACNG4;CACNG5;CACNG6;ATF2 |
| Cardiac muscle contraction | LOC491979;LOC100683828;FXYD2;LOC102155410;UQCRQ;MYL4;MYL2;TNNC1;LOC102156968;CACNA1C;CACNA1D;CACNA1F;LOC609990;LOC100686830;CACNA1S;ATP1B4;ATP1B2;ATP1B3;ATP1B1;CACNA2D2;CACNA2D3;CACNA2D1;MYL3;CACNA2D4;COX6A2;LOC119870901;LOC102152785;UQCRFS1;UQCRC2;UQCRC1;ATP2A1;ATP2A2;CACNB2(up);COX7B2;TNNT2;SLC9A1;LOC489252;LOC477508;LOC100684983;UQCRH;COX7A1;LOC119863903;RYR2;ATP2A3(down);ACTC1;LOC100686500;LOC102153059;LOC100684842;MYH7;CACNB3;CACNB1;TPM4;TPM3;TPM2;TPM1;CACNB4;SLC8A1;SLC8A2;COX8A;COX4I2;COX4I1;LOC100683076;LOC100686510;ATP1A4;ATP1A3;ATP1A1;LOC102151754;COX5A;COX5B;UQCR10;UQCR11;SLC8A3(up);LOC119865581;COX2;COX3;CYTB;COX1;TNNI3;LOC612644;COX7A2L;CYC1;LOC102156642;CACNG1;CACNG2;CACNG3;CACNG4;CACNG5;CACNG6;CACNG7 |
| IL-17 signaling pathway | CCL2;PTGS2;CCL7;ELAVL1;CXCL8(up);NFKBIA;JUND;HSP90B1;LOC119874472(up);IKBKB;MUC5B(down);IL25;MAPK11;MAPK12;IKBKE;MAPK10;TRAF4;IL1B;CSF3;LOC100687242;TRAF5;CEBPB;SRSF1;TRAF6;TBK1;ANAPC5;JUN;MAP3K7;TRADD;HSP90AB1;MAPK3;MAPK1;FOSL1;MAPK7;FADD;NFKB1;RELA;MAPK9;IL17D;TRAF2;TRAF3;CCL20;IL17A;IL17B;FOS;IFNG;MMP3;CHUK;LOC106557449;LOC102154292;TNF;LOC480466;FOSB(up);LOC612100;MAPK13;MAPK8;CXCL10(down);MAPK14;CASP3;CSF2;TAB3;TAB2;LOC119876918(up);IL13;CASP8;GSK3B;IL6;IL4;IL5;TNFAIP3;MMP9;HSP90AA1;IL17RB;IL17RA;MMP1 |
| Cysteine and methionine metabolism | APIP;PHGDH;LDHAL6B;MAT1A;LOC102152275;BHMT2;MDH2;MDH1;TST;CTH;LOC100686034;TAT;ADI1;GCLM;AHCYL1;BHMT;AHCYL2;DNMT1;GCLC;LOC100684709;MPST;CDO1;MRI1;GOT2;KYAT1;IL4I1;KYAT3;LDHA;LDHB;AMD1;DNMT3B;DNMT3A;MAT2A;LOC100685416;MAT2B;MTR;LOC482436;ENOPH1;LOC609911;BCAT2;SRM;BCAT1;LOC119866771;LOC490690;AGXT2;LOC102153001;MTAP;LOC100688166;GOT1;SDSL;LDHC;SDS;PSAT1;CBS;LOC100856397;LOC102151507;AHCY |
| Salivary secretion | ADRB2(down);AQP5;FXYD2;LOC486923;DMBT1(up);ADCY1\_1;LOC102153034(down);PRKG2;SLC9A1;CALML6;CD38(up);ATP2B1;MUC5B(down);PLCB3;LOC607460;SLC4A2;LOC485435;ATP2B2(up);CALM2;CALM3;CALM1;ATP1A3;RYR3;CAMP;KCNMA1;LYZ;GUCY1A1;KCNN4;GUCY1A2;PRKG1;SLC12A2;LPO;BST1;LOC607874;VAMP2;LOC119869567;NOS1;PLCB2;GNAQ;PLCB4;LYZF2;PRKCA;MUC7;ATP1B4;PRKCB;CHRM3;ATP1B3;PRKCG;ATP1B1;ADCY4(up);PLCB1(up);ADCY7;ITPR2;ITPR3;ATP2B3;ITPR1;ATP2B4;ATP1B2;ADCY1;ADRA1A;ADCY5;ADCY6;ADRA1B;ADRA1D;ADCY2;GNAS;ADCY3;ADCY8;ADCY9;CALML5;CALML4;ADRB1;ADRB3;PRKACA;GUCY1B1;ATP1A1;PRKACB;ATP1A4;BEST2 |
| Focal adhesion | MYL7(up);LOC100856339;ACTG1;CRKL;FYN;CAV3;AKT1;AKT2;AKT3;MYL2;RAPGEF1;CAV2;LOC488190;ZYX;MYL9;PIK3CA;PIK3CB;PIK3CD;COL1A2(up);CAPN2;PARVB(up);ERBB2;LOC607207;IGF1R;DIAPH1;SHC2;SHC3;RAP1B;EGFR;MYL12B;VEGFA;FN1;CCND1;LOC610614;CCND3;CCND2;MYLK;GSK3B;PTEN;MYL5;PAK4;PAK5;PAK6;PAK1;PAK2;ITGA8;ITGA9;ITGA1;ITGA2;ITGA3;ITGA4;ITGA5;ITGA6;ITGA7;ITGA11;BCL2;LAMB1;ARHGAP5;LAMB3;LAMB2;IBSP;LAMB4;RAC3;RAF1;THBS4;TNR;THBS2;THBS3;THBS1;ELK1;PIK3R2;PIK3R1;CAV1;PAK3(up);PDGFB;IGF1;LOC119870217;LOC119870216;LOC119870215;MYL10;COL4A6;COL4A5;COL4A4;COL4A3;COL4A2;COL4A1;VEGFB;LAMC2;LAMC3;RHOA;LAMC1;BCAR1;ACTB;HRAS;RAC2(up);VEGFD;VAV1;GRB2;VAV3;FLNC(up);ITGAV;FLNA;FLNB;PDGFD;PDGFRB;PDGFRA;VEGFC;CHAD;VAV2;ILK;RAP1A;XIAP;MAPK10;SOS2;FLT4;COL2A1;FLT1;ITGB8(down);TNN;ITGA2B;MAPK3;MAPK1;PPP1R12A;PPP1R12B;PPP1R12C;MAPK8;PARVA;EGF;PARVG;KDR;RELN;SRC;LAMA2;LAMA3;LAMA5;COMP;MYLK2;MYLK3;COL9A1;LAMA4(up);LOC119866928;MAPK9;RASGRF1;PIP5K1C;PIP5K1B;PIP5K1A;PPP1CA;MAP2K1;MET;SPP1;RAC1;COL1A1;CDC42;SHC4;PTK2;LAMA1;BIRC3;BIRC2;CTNNB1;BAD;MYLPF;CRK;SHC1;PGF;ITGA10(up);TLN2;TLN1;PXN;BRAF;ITGB1;PDGFA;ITGB3;PDGFC;ITGB5;ITGB4;ITGB7;ITGB6;PRKCA;COL9A2;PRKCB;VCL;PRKCG;COL9A3;JUN;TNC;HGF;VASP;VTN;VWF;ACTN1;TNXB;PPP1CB;PPP1CC;ACTN4;ROCK1;ROCK2;DOCK1;ARHGAP35;COL6A1;PDPK1;COL6A3;COL6A2;COL6A5;COL6A6 |
| Ovarian steroidogenesis | CYP2J2;PTGS2;CYP19A1;STAR;ADCY1\_1;CYP17A1;INSR;BMP15;BMP6;AKR1C3;LHCGR;CYP11A1;CYP1B1;FSHB;CGA;FSHR;HSD17B1;HSD17B2;ALOX5;HSD17B7;LOC119869567;IGF1R;IGF1;CYP1A1(up);ACOT4(up);PLA2G4E(up);ADCY4(up);LDLR;PLA2G4B;PLA2G4A;LOC490770;PLA2G4F;PLA2G4D;ADCY5;ADCY6;ADCY7;ADCY1;ADCY2;GNAS;SCARB1;ADCY3;ADCY8;ADCY9;INS;PRKACA;HSD3B2;PRKACB |
| Carbon metabolism | LOC611563;PCCB;PCCA;TPI1;FBP2;OGDH;HKDC1;TKTL1;GOT2;RPIA;GOT1;LOC477441;GPI;HK2;IDH2;IDH1;HK1;PHGDH;GPT;LOC608800;EHHADH;SHMT2;SHMT1;LOC102154094;ECHS1;LOC111094513;ACOX3;LOC106558345;LOC102151507;PKM;LOC481849;ACADS;ME3;PGLS;ME2;GAPDH;H6PD;LOC102152275;LOC482320;RGN;LOC485342;PC;TKT;PDHB;PGK2;AMT;PGK1;DLD;GCK;DLAT;ACO1;ACO2;LOC102151775;SDSL;ACOX1;GLYCTK;TKFC;CPS1;ESD;GCSH;PGAM2;ME1;PGAM1;FH;PKLR;GPT2;ACAT1;ACAT2;LOC610338;PDHA2;LOC102152592;MTHFR;ACSS2;ACSS1;ENO1;ENO2;NUDT11;ENO4;HIBCH;CS;ADH5;PSPH;DLST;SUCLA2;SDS;AGXT;GLUD1;LOC119876526;MDH1;TALDO1;CAT;MDH2;ADPGK;LOC111096459;IDH3A;PGD;IDH3B;PRPS2;IDH3G;PRPS1;HADHA;PFKP;LOC479379;PFKM;PFKL;RPE;LOC111089999;IDNK;HK3;MCEE;OGDHL;LOC119870407;LOC100683724;SUCLG2;ALDH6A1;SUCLG1;LOC119866771;ALDOA;ALDOC;ALDOB;HAO2;MMUT;GLDC(down);PSAT1;ENO3;FBP1(up);SDHA;HAO1;SDHC;SDHB;SDHD |
| Progesterone-mediated oocyte maturation | LOC100856339;PLK1;ADCY1\_1;PKMYT1;AKT1;AKT2;AKT3;ARAF;PIK3CA;PIK3CB;PIK3CD;ANAPC10;ANAPC13;HSP90AB1;IGF1R;ADCY4(up);KRAS;ADCY5;ADCY6;ADCY7;ADCY1;ADCY2;ADCY3;FZR1;ADCY8;ADCY9;LOC100684434;CDC16;CDK1;LOC100687242;LOC119870217;PIK3R2;PIK3R1;LOC119870216;LOC119870215;LOC119869567;IGF1;BUB1;CDC25C;CDC25B;CDC25A;LOC100856295;LOC100685549;GNAI1(up);ANAPC1;LOC612100;ANAPC5;ANAPC4;ANAPC7;RPS6KA1;RPS6KA2;RPS6KA6;CDC27;CDC26;CDC23;INS;LOC476070;ANAPC11;LOC487020;MAPK14;MAPK10;MAPK11;MAPK12;MAPK13;GNAI2;GNAI3;ANAPC2;SPDYA;SPDYC;PDE3B;MAPK3;MAPK1;RAF1;LOC102156563;MAPK8;MAPK9;MOS;CDK2;PRKACA;PRKACB;HSP90AA1;MAD2L2;CPEB4;CPEB1;CPEB3;CPEB2;MAP2K1;CCNA2;CCNA1;MAD1L1;PGR;AURKA;LOC100682940;CCNB1;BRAF |
| Protein export | SPCS1;LOC102152142;SPCS3;OXA1L;SRP9;SEC63;HSPA5;IMMP2L;LOC102151183;SRP54;SRPRB;SPCS2;SRPRA;LOC106559577;SRP14;SRP19;SRP72;SEC62;IMMP1L;SEC61G;SEC61B;SEC11A;SEC11C;SRP68;SEC61A1;SEC61A2 |
| Glutamatergic synapse | PPP3R2;PPP3R1;ADCY1\_1;DLG4;GNG14;CACNA1A;CACNA1C;GNG10;GNG11;GNG12;GNG13;LOC119863873;DLGAP1;GRIN1;SLC1A1(up);SLC38A1;SLC38A2;PLA2G4E(up);ADCY4(up);PLD1;ADCY5;ADCY6;ADCY7;ADCY1;ADCY2;ADCY3;SHANK2;ADCY8;ADCY9;GNG7;GNG4;GNG5;GNG2;GNG3;SLC17A6;GNG8;GRIK3;GRIA2;GRIA3;GRIA1;GRIA4;GLUL;LOC100855681;GRM8;GRM4;GRM5;GRM6;GRM7;GRM1;GRM2;GRM3;LOC119869567;PLCB2;PLCB3;PLCB4;GNAI1(up);ITPR2;ITPR3;ITPR1;GRK2;GNAQ;GNAS;SLC1A3(up);GNB4(up);GRK3;LOC612295;TRPC1;GRIN2D;GLS;GNAI2;GNAI3;PLA2G4F;MAPK3;MAPK1;GNAO1;GNB5;GNB1;GNB3;GNB2;PLCB1(up);KCNJ3;SLC17A8;GRIK1;GRIK2;SLC17A7;GRIK4;GRIK5;PRKACA;PRKACB;GLS2;PLD2;PPP3CB;GRIN3A;GRIN3B;HOMER1;HOMER3;SLC38A3(down);PRKCA;PRKCB;PRKCG;PLA2G4B;PLA2G4A;SHANK3;CACNA1D;SHANK1;PLA2G4D;SLC1A7;GRIN2B;GRIN2C;GRIN2A;GNGT2;GNGT1;PPP3CA;SLC1A6;PPP3CC;SLC1A2 |
| Retinol metabolism | AOX2;LOC100856533;AOX4;RETSAT;LOC102154822;RPE65;LOC480777;UGT2A3;LOC119867230;ALDH1A1(down);DHRS3;UGT1A6(down);LOC102154742;CYP2S1;AWAT2;RDH8;DHRS9;DGAT1;DHRS4;RDH5;CYP2B6;RDH16;CYP26B1;BCO1;ALDH1A2;PNPLA4;LRAT;LOC489851;HSD17B6;RDH11;SDR16C5;CYP2C18;CYP1A1(up);ADH5;ADH4;CYP4A11(down);CYP1A2(up);RDH10;CYP26A1;RDH12;LOC100688697;CYP4A38(down);CYP26C1 |
| Renin-angiotensin system | ACE;REN;AGT;CPA3;ATP6AP2;ENPEP(up);NLN;LNPEP;CTSA;MRGPRD;CTSG;KLK1;KLK2;MAS1;ACE2;THOP1;CMA1;ANPEP;MME;AGTR2;AGTR1;PRCP;PREP;LOC100856208 |
| Inflammatory mediator regulation of TRP channels | LOC100856339;ADCY1\_1;CALM2;CALM3;CALM1;PTGER4;PIK3CA;PIK3CB;PIK3CD;NTRK1;PTGER2;PLA2G6;TRPV4;TRPV1;TRPV3;TRPV2;PIK3R1;CAMK2D;CAMK2G;CAMK2A;CAMK2B;ADCY4(up);TRPM8(down);ADCY5;ADCY6;ADCY7;ADCY1;ADCY2;ADCY3;ADCY8;ADCY9;CYP4A38(down);NGF;TRPA1(down);PLCB3;IL1R1;HTR2A;PIK3R2;HTR2C;HRH1;LOC119869567;PLCB2;IGF1;PLCB4;ASIC5;ASIC4;PRKACB;ASIC1;ASIC3;ASIC2;ITPR2;ITPR3;ITPR1;GNAQ;GNAS;CALML6;CALML5;CALML4;CYP2J2;HTR2B;LOC102153034(down);MAPK14;PRKCQ;MAPK10;MAPK11;MAPK12;MAPK13;IL1B;MAPK8;MAPK9;SRC;PLCB1(up);BDKRB1;BDKRB2;KNG1;PRKACA;LOC100688697;F2RL1;MAP2K6;MAP2K3;PLCG2;PLCG1;ALOX12;PRKCH;PRKCA;PRKCB;PRKCE;PRKCD;PRKCG;PLA2G4E(up);IL1RAP;P2RY2;PLA2G4B;PLA2G4A;PLA2G4F;PLA2G4D;PPP1CB;PPP1CC;PPP1CA;CYP4A11(down);CYP2C18 |
| Aminobenzoate degradation | ECHS1;HADHA;EHHADH;ACYP1;ACYP2 |
| Novobiocin biosynthesis | TAT |
| Phenylalanine, tyrosine and tryptophan biosynthesis | TAT;LOC482436;GOT2;PAH;IL4I1;GOT1 |
| Adipocytokine signaling pathway | PCK2;PCK1;NFKBIB;PRKAG3;NFKBIA;G6PC1;LOC485024;G6PC3;NFKBIE;STK11;AKT1;AKT2;AKT3;MAPK10;ACSBG1;ACSBG2;LOC111096471(down);ADIPOQ;ADIPOR2;ADIPOR1;CD36;PPARGC1A;TRADD;PRKAG1;JAK2;IKBKB;LOC119870216;TRAF2;NFKB1;RELA;MAPK9;SLC2A1;CPT1A;CPT1C;CPT1B;PRKAB2;IRS2;AGRP;PRKAB1;LOC119870217;LOC100683481;LOC119870215;RXRG;RXRA;RXRB;PRKAA2;PRKAG2;LEPR;TNF;MTOR;PRKAA1;POMC;PRKCQ;STAT3;G6PC2;MAPK8;ACSL5(up);ACACB(down);SLC2A4;TNFRSF1B;SOCS3;TNFRSF1A;IRS4;ACSL3;PTPN11;ACSL1;ACSL6;IRS1;ACSL4;LEP;CAMKK2;NPY;CHUK;PPARA |
| Naphthalene degradation | ADH5 |
| Relaxin signaling pathway | LOC119870307;LOC100856339;ADCY1\_1;AKT1;AKT2;AKT3;RXFP1;PIK3CA;PIK3CB;RXFP2;PIK3CD;GNG11;GNG12;GNG13;LOC119863873;COL1A2(up);RELA;KRAS;SHC4;SHC1;SHC2;SHC3;CREB5;CREB3;CREB1;ADCY4(up);MMP2(down);ADCY5;ADCY6;ADCY7;ADCY1;ADCY2;ADCY3;ADCY8;ADCY9;GNG7;GNG4;GNG5;GNG2;GNG3;ARRB2;GNG8;ACTA2;GNAO1;HRAS;NFKBIA;COL3A1;EDNRB;RAF1;LOC100855681;JUN;GRB2;PIK3R2;PIK3R1;GNA15;LOC119869567;PLCB2;PLCB3;PLCB4;FOS;LOC119870217;LOC119870216;LOC119870215;PRKACB;GNAI1(up);COL4A6;COL4A5;COL4A4;VEGFA;COL4A2;COL4A1;VEGFB;ARRB1;VEGFD;GNG14;COL4A3;RXFP3;VEGFC;GNB4(up);SMAD2;SMAD3;MAPK14;MAPK10;MAPK11;MAPK12;MAPK13;GNAI2;GNAI3;MAPK3;MAPK1;NFKB1;MAPK8;MAPK9;SRC;NOS2(up);GNB5;GNB1;GNB3;GNB2;LOC102154292;PLCB1(up);SOS2;GNAS;PRKACA;COL1A1;TGFB1;TGFBR2;EGFR;TGFBR1;MAP2K2;MAP2K1;MAP2K7;MAP2K4;NRAS;EDN1(down);GNG10;NOS1;NOS3;GNGT1;PRKCA;PRKCZ;CREB3L2;CREB3L3;CREB3L1;GNGT2;CREB3L4;MMP9;ATF4;ATF6B;ATF2;MMP1 |
| Cortisol synthesis and secretion | STAR;ADCY1\_1;MC2R;CYP17A1;ORAI1;KCNK2;KCNK3;CACNA1H;CACNA1I;NCEH1;CYP11A1;CACNA1C;CACNA1D;CACNA1F;CACNA1G;AGT;SP1;AGTR1;CACNA1S;LOC119869567;PLCB2;PLCB3;PLCB4;HSD3B2;CYP21A2;CREB5;PRKACB;CREB3;CREB1;ADCY4(up);PLCB1(up);LDLR;NR4A1(up);CYP11B2;ITPR2;ITPR3;ITPR1;PDE8A;PDE8B;ADCY1;ADCY5;ADCY6;ADCY7;GNAQ;ADCY2;ADCY3;SCARB1;GNAS;ADCY8;ADCY9;CREB3L2;CREB3L3;POMC;CREB3L1;PRKACA;CREB3L4;GNA11;ATF4;ATF6B;ATF2 |
| Renin secretion | ADRB2(down);EDN3;PPP3R2;ACE;ADORA1;CACNA1D;LOC102153034(down);PLCB3;REN;CACNA1F;ADCYAP1;CLCA2;LOC485435;AGT;GNAI2;GNAI3;CLCA4;CLCA1;CALM2;CALM3;CALM1;PTGER4;PDE1B;PDE1A;CACNA1C;PDE3A;KCNMA1;PTGER2;PDE3B;GUCY1A1;GUCY1A2;PRKG2;PPP3R1;CACNA1S;PDE1C;KCNJ2(down);NPPA;NPR1;PLCB2;ORAI1;PLCB4;AQP1;CTSB;CREB1;GNAI1(up);PLCB1(up);PPP3CA;ITPR2;ITPR3;ITPR1;PPP3CB;ADCY5;ADCY6;EDN2;EDN1(down);GNAQ;GNAS;ADCYAP1R1;CALML6;CALML5;CALML4;ADRB1;ADRB3;PRKACA;GUCY1B1;PRKACB;EDNRA(up);AGTR1;PPP3CC;LOC100856208 |
| Thiamine metabolism | NTPCR;AK8;AK5(up);AK2;AK1;AK7;LOC102156938;LOC611724;AK4;NFS1;TPK1;ALPI;THTPA;ALPL |
| Phenylalanine metabolism | TAT;GOT1;ALDH3B1;AOC2;GOT2;LOC482436;ALDH3A1;AOC3;MIF;MAOB;MAOA;GLYAT;PAH;DDC;IL4I1;HPD;LOC483462;ALDH1A3 |
| Chlorocyclohexane and chlorobenzene degradation | CMBL |
| Benzoate degradation | ACAT1;ACAT2;GCDH |
| Hedgehog signaling pathway - fly | FBXW11;SMURF1;SMURF2;SPOP;SKP1;GRK6;GRK4;GRK5;SPOPL;EN1;EN2;CSNK1E;CUL1;CUL3;SMO(up);CSNK1G2;CSNK1G3;CSNK1G1;SUFU;BMP4;CSNK1A1;BTRC;GSK3B;PRKACA;STK36;PRKACB;RBX1;LOC111090648;PTCH1 |
| Fluorobenzoate degradation | CMBL |
| Caprolactam degradation | HADH;HADHA;ECHS1;AKR1A1;EHHADH;RGN |
| Leukocyte transendothelial migration | MYL7(up);LOC100856339;ACTG1;NCF1;RAPGEF4;PTK2B;MYL5;MYL2;RAPGEF3;MYL9;PIK3CA;PIK3CB;NCF4;PIK3CD;THY1;CDH5;LOC607207;CXCL12;MMP2(down);PTPN11;ACTB;MAPK12;ITGA4;ARHGAP5;PECAM1(up);EZR;CLDN8;CLDN9;PIK3R2;PIK3R1;ITK;CLDN1;CLDN2;CLDN3;CLDN4;CLDN5;CLDN6;CLDN7;CLDN20;JAM3;LOC100683481;MYL10;GNAI1(up);RHOH;PRKCA;PRKCG;RHOA;BCAR1;F11R;RAC2(up);CD99;VAV1;VAV3;VAV2;ITGAL;ITGAM;ESAM;NCF2;CLDN10;CLDN11;CLDN16;CLDN17;CLDN14;CLDN15;CLDN18;CLDN19;RAP1B;RAP1A;MAPK14;MAPK11;CTNND1;MAPK13;GNAI2;GNAI3;ICAM1;SIPA1;RASSF5;VCAM1;OCLN;LOC119866928;ITGB2(down);MYL12B;PLCG2;AFDN;CDC42;PTK2;CTNNB1;MSN;PLCG1;MYLPF;TXK;PXN;ITGB1;RAC3;RAC1;CLDN23;PRKCB;VCL;CLDN25;CLDN24;CYBA;VASP;CTNNA1;CTNNA2;CTNNA3;ACTN1;ACTN4;LOC119863891(up);ROCK1;ROCK2;ARHGAP35;LOC482919;MMP9 |
| Biosynthesis of ansamycins | TKTL1;TKT |
| PD-L1 expression and PD-1 checkpoint pathway in cancer | LOC100856339;PPP3R2;PPP3R1;IKBKB;AKT1;AKT2;AKT3;EGF;LOC111090226;PIK3CA;PIK3CB;PIK3CD;LOC102156776;RELA;LOC102156003;CD28;IFNG;JAK1;CSNK2A2;CSNK2A1;KRAS;CD4;PTPN11;TLR2;PTEN;LAT;CD3D;CD3E;CD3G;NFATC1;NFATC2;RASGRP1;NFKBIB;HRAS;NFKBIA;HIF1A;NFKBIE;TLR9\_1;IFNGR1;IFNGR2;TIRAP;JUN;LOC102156901;JAK2;PIK3R2;PIK3R1;LOC119870216;MYD88;FOS;LOC119870217;LOC100683481;LOC119870215;CHUK;LOC119872979;PTPN6;TLR4(down);CD274;MAP3K3;LCK;MAPK14;NFATC3;MAPK11;MAPK12;MAPK13;RAF1;LOC106559087;TICAM1;TICAM2;STAT3;STAT1;RPS6KB1;RPS6KB2;MAPK3;MAPK1;NFKB1;PDCD1;MTOR;PLCG1;CSNK2B;LOC119869603;EGFR;LOC119869607;MAP2K3;MAP2K2;MAP2K1;CD247;MAP2K6;NRAS;ZAP70;LOC609053;TRAF6;PRKCQ;LOC480788;PPP3CA;PPP3CB;PPP3CC |
| Glyoxylate and dicarboxylate metabolism | GRHPR;PCCB;HYI;PCCA;LOC102152275;ACO1;LOC612295;MDH2;MDH1;LOC485342;AFMID;ACAT1;ACAT2;MCEE;AMT;GLYCTK;DLD;ACSS2;ACSS1;LOC102151507;MMUT;CAT;LOC119866771;CS;ACO2;SHMT2;SHMT1;HOGA1;AGXT;GLUL;HAO1;HAO2;GLDC(down);GCSH |
| Cushing syndrome | ADCY1\_1;MC2R;CDKN2B\_1(down);RB1;NR4A1(up);LOC612475;CACNA1H;CACNA1I;NCEH1;WNT8A;WNT10A;WNT10B;CACNA1C;CACNA1D;CACNA1F;CACNA1G;AGT;WNT16;CACNA1S;CDKN2B;CDKN2C;CAMK2D;SP1;CREB5;CAMK2G;CREB3;CAMK2A;CREB1;ADCY4(up);PDE8A;PDE8B;ADCY5;ADCY6;ADCY7;ADCY1;ADCY2;ADCY3;CCND1;SCARB1;ADCY8;TCF7L1;TCF7L2;GSK3B;HSD3B2;APC;WNT7A;WNT7B;STAR;RASD1;CDKN1B;CDKN1A;TCF7;ADCY9;PLCB3;FZD10;CYP11A1;CCNE2;CCNE1;CDK4;LOC119869567;PLCB2;ORAI1;PLCB4;GNAI1(up);ITPR2;ITPR3;ITPR1;GNAQ;GNAS;MAP2K2;LOC119863906;WNT5B;AGTR1;WNT3A;RAP1B;RAP1A;FH;PDE11A;GNAI2;GNAI3;FZD1;FZD2;FZD3;FZD4;FZD6;FZD7;FZD8;FZD9;MAPK3;MAPK1;PLCB1(up);LDLR;POMC;LEF1;ARNT;WNT3;WNT2;WNT1;WNT6;WNT4;CDK2;PRKACA;CDK6;PRKACB;CRHR2;CRHR1;USP8;EGFR;CTNNB1;CYP17A1;MAP2K1;CRH;AXIN2;AXIN1;KCNK2;KCNK3;DVL2;DVL3;WNT8B;DVL1;WNT9B;WNT2B;KMT2A;WNT9A;KMT2D;CYP21A2;CAMK2B;ATF4;APC2;WNT5A;E2F3;E2F2;E2F1;CREB3L2;CREB3L3;CREB3L1;BRAF;CREB3L4;GNA11;CYP11B2;ATF6B;WNT11;ATF2 |
| Glycerophospholipid metabolism | PLB1(up);LYPLA1;LYPLA2;GPAT3;GPAT2;PHOSPHO1(up);ACHE(down);GPAM;SELENOI;PLA2G6;PLA2G5;PLA2G3;ETNK2;PCYT1A;PCYT2;PLA2G4E(up);GNPAT;PLD4;PLA2G2C(down);PLD1;PLD2;PLD3;ETNPPL;PLA2G1B;PGS1;CHKA;CHKB;PLA2G12B;PLA2G12A;PNPLA7;PNPLA6;ADPRM;PLA1A;PLA2G15;PLA2G10;PISD;CRLS1;GPD1L;PTDSS2;LCLAT1;LPCAT4;MBOAT2;MBOAT1;MBOAT7;CHAT;CDIPT;CHPT1;GPCPD1;PLPP1;PLPP3;PLPP2;PLPP5;PLPP4;PLAAT3(down);PLA2G2E;PLA2G2D;PLA2G2F;CDS1;CDS2;AGPAT5;AGPAT4;AGPAT3;AGPAT2;AGPAT1;LCAT;GPD1;GPD2;LPGAT1;DGKZ;LPIN2;LPIN3;LPIN1;DGKQ;DGKK;DGKH;DGKI;LPCAT2;LPCAT3;ETNK1;LPCAT1;DGKB;DGKA;DGKG;DGKD;DGKE;PLA2G4B;PLA2G4A;PLA2G4F;PLA2G4D;PEMT;CEPT1 |
| Ether lipid metabolism | AGPS;PLB1(up);UGT8;PLA2G1B;PLA2G4B;GAL3ST1;TMEM86B;PLA2G12B;PLA2G12A;LPCAT4;PLA2G6;PLA2G7;SELENOI;PLA2G5;LPCAT2;PLA2G3;LPCAT1;PLPP1;PLPP3;PLPP2;ENPP6;ENPP2;PLAAT3(down);PLA2G4E(up);PLA2G2E;PLA2G2D;PLA2G2F;CHPT1;PLA2G4A;PAFAH1B1;PLA2G4F;PAFAH1B3;PLA2G4D;PLD4;PLA2G2C(down);PAFAH2;PLA2G10;PLD1;PLD2;PLD3;CEPT1 |
| Inositol phosphate metabolism | OCRL;TPI1;PI4KB;PI4KA;PLCZ1;IPMK;PLCB3;PLCG2;PLCG1;MIOX;PI4K2B;PLCH1;PI4K2A;CDIPT;ALDH6A1;PIK3C2A;INPPL1;PIK3CA;PIK3CB;PIK3CD;INPP4B;INPP4A;ITPKA;ITPKC;ITPKB;SYNJ1;SYNJ2;MTMR7(up);MTMR6;MTMR4;PIP4K2A;PIP5KL1;PIP4K2C;PIP4K2B;ITPK1;PLCB2;MTMR14;PLCB4;ISYNA1;PIKFYVE;MTM1;PIK3C2B;IMPA1;IMPA2;PIK3C2G;PLCD3;PLCD1;PIK3C3;IPPK;MTMR3;INPP5A;INPP5B;INPP1;INPP5D;INPP5E;PLCB1(up);PIP5K1C;PIP5K1B;INPP5J;INPP5K;PLCD4;MTMR2;PTEN;MTMR1;PLCE1;MINPP1;PIP5K1A |
| Glycosylphosphatidylinositol (GPI)-anchor biosynthesis | PGAP1;LOC119872260;DPM2;PIGA;PIGB;PIGC;PIGF;PIGG;PIGH;PIGK;PIGL;PIGM;PIGN;PIGO;PIGQ;PIGS;GPLD1(down);PIGU;PIGV;PIGW;PIGX;PIGY;PIGZ;LOC478413;GPAA1;PIGT |
| Glycerolipid metabolism | DGKK;DGAT2;AGK;LCLAT1;ALDH7A1;MGLL;GPAT2;MBOAT2;MBOAT1;AKR1A1;PNLIP;ALDH2;PNLIPRP1;PNLIPRP3;PNLIPRP2;LPIN2;LPIN3;LPIN1;DGAT1;CEL;PNPLA3;PNPLA2;DGKH;DGKI;GK2;DGKB;DGKA;DGKG;DGKD;DGKE;PLPP1;DGKZ;PLPP3;PLPP2;PLPP5;PLPP4;LIPF;GPAT3;LPL;GPAM;MOGAT2;MOGAT3;MOGAT1;ALDH1B1;AKR1B1;GK;GLYCTK;TKFC;AGPAT5;AGPAT4;AGPAT3;AGPAT2;AGPAT1;DGKQ;ALDH9A1 |
| Fat digestion and absorption | DGAT2;CLPS;CD36;PNLIP;PLA2G1B;FABP1;APOA4;APOA1;PNLIPRP1;PNLIPRP2;APOB;DGAT1;PLA2G12B;PLA2G12A;CEL;ACAT1;ACAT2;PLA2G5;PLA2G3;GOT2;PLPP1;PLPP3;PLPP2;LIPF;PLA2G2E;PLA2G2D;PLA2G2F;SLC27A4;SLC27A1;PLA2G2C(down);PLA2G10;ABCG5;ABCG8;SCARB1;NPC1L1(down);MTTP;AGPAT2;AGPAT1;ABCA1;MOGAT2;MOGAT3 |
| Vascular smooth muscle contraction | AVPR1B;AVPR1A;ACTG2;CALCA;ADCY1\_1;MYL6;ARAF;AGT;LOC488190;MYL9;CALM2;CALM3;CALM1;CACNA1C;CACNA1D;CACNA1F;ARHGEF1;PLA2G6;KCNU1;PLA2G3;EDN3;CACNA1S;ARHGEF11;ARHGEF12;ADCY7;ADCY5;CALD1(up);ADCY6;PLA2G4E(up);RAMP2;ADCY4(up);ADRA1B;ADRA1D;ADRA1A;PLA2G2C(down);KCNMB3;KCNMB2;KCNMB4;ADCY2;ADCY3;ADCY8;MYLK;GUCY1B1;MAPK1;CYP4A38(down);ADCY1;ACTA2;ADCY9;PLA2G1B;ADORA2B;PLA2G12B;PLA2G12A;KCNMA1;GUCY1A1;GUCY1A2;PRKG1;NPPB;NPPC;NPPA;LOC119869567;PLCB2;PLCB3;PLCB4;ITPR2;ITPR3;RHOA;ITPR1;PLA2G10;GNAQ;GNAS;AVP;CALML6;CALML5;CALML4;KCNMB1;AGTR1;MYH10;MYH11;MYH13;MYH14;MYH15;MYH16;LOC102153034(down);RAF1;MYH2;MYH3;MYH1;MYH4;MYH8;MYH9;IRAG1;MAPK3;PLA2G5;PPP1R12A;PPP1R12B;PPP1R12C;LOC485435;MYLK2;MYLK3;PLCB1(up);PLA2G2E;PLA2G2D;PLA2G2F;MYH7B;ADM;LOC100686142;EDN2;PRKACA;PRKACB;MYL6B;NPR1;NPR2;MAP2K2;MAP2K1;ADORA2A(up);EDN1(down);GNA13;PRKCH;CALCRL;PRKCA;RAMP1;PRKCB;PRKCE;PRKCD;PRKCG;PTGIR;RAMP3;EDNRA(up);PRKCQ;PLA2G4B;PLA2G4A;PLA2G4F;PLA2G4D;PPP1CB;PPP1CC;PPP1CA;CYP4A11(down);ROCK1;ROCK2;GNA12;BRAF;GNA11;CRSP-3;CRSP-2;CALCB;CRSP-4 |
| Pyrimidine metabolism | LOC102156116;NT5E;UPRT;DCTPP1;RRM2;RRM1;LOC102154109;LOC102152523;CAD;NT5M;CTPS2;CTPS1;DPYD;CMPK1;CMPK2;DCTD;NT5C3A;NT5C3B;DPYS;UPP1;LOC100688420;RRM2B;NT5C1B;NT5C1A;NT5C2;ENTPD6;ENTPD5;ENTPD4;ENTPD3;UMPS;ENTPD1;UCK1;NUDT2;UCK2;AK9;ENPP3(down);ENTPD8;TK2;TK1;ENPP1;DUT;CDA;NME4;LOC111091468;NME6;NME7;NME1;NME2;NME3;LOC102151128(down);PNP;DHODH;UCKL1;TYMS;DTYMK;LOC102155863;CANT1;UPB1(up);DCK;LOC483943;NT5C |
| Human papillomavirus infection | PTGS2;LOC100856339;DLA-64;PPP2R3B;TNXB;PPP2R3A;PPP2R5E;IKBKB;AKT1;AKT2;AKT3;IKBKE;TYK2;EGF;PRKCZ;DLG1;DVL2;LOC100686787;PPP2R3C;WNT8A;PTGER4;PIK3CA;PIK3CB;LFNG(up);PIK3CD;PARD6B;PARD6A;PPP2R5C;DVL1;COL1A2(up);RELN;MAGI1;FADD;RELA;WNT10A;GNAS;RBPJL;DLA-79;PPP2R2D;CREB5;JAK1;CREB3;CRB3;ATP6V1H;CSNK1A1;FASLG;PSMC1;RFNG;MDM2;KRAS;IFNAR2;STAT1;FN1;CCND1;LOC610614;CCND3;CCND2;WNT2B;TCF7L1;TCF7L2;GSK3B;PTEN;GRB2;CREB3L2;PARD6G(up);APC;WNT7A;WNT7B;ITGA8;ITGA9;ATP6V1F;ATP6V1A;ITGA1;HRAS;ITGA3;ITGA4;ITGA5;ITGA6;ITGA7;ITGA11;LAMB1;TBPL1;LAMB3;LAMB2;IBSP;LAMB4;DLG2(up);MAML2;MAML1;CCNE2;COL2A1;THBS4;TNR;THBS2;THBS3;THBS1;LOC481939;ISG15;PIK3R2;PIK3R1;TLR3;CDK4;LOC119876828;PKM;TCF7;PPP2CB;LOC111090910;FZD1;LOC119870217;LOC119870216;LOC119870215;PRKACB;DLA88;CHUK;COL4A6;COL4A5;CDKN1A;COL4A3;COL4A2;COL4A1;LAMC2;LAMC3;LAMC1;WNT3A;IRF3;WNT9A;LOC100686162;TP53;CCNE1;TNFRSF1A;LOC100683567;TBP;IRF9;ITGAV;ATP6V0A4;COL4A4;EIF4EBP1;ATP6V1G2;LOC119863905;FZD9;PPP2R1A;VEGFA;VWF;RBPJ;CREBBP;PPP2CA;IFNAR1;PDGFRB;HDAC2;MFNG;CHAD;ATP6V1D;FZD6;ATM;BAK1;ATR;MPP5;IFNB1;TSC2;LOC611406;TSC1;TICAM1;ITGB8(down);TNN;FZD4;STAT2;TBK1;FZD7;FZD8;ITGA2B;PSEN1;WNT9B;RPS6KB1;TRADD;RPS6KB2;ATP6V0D2(up);MAPK3;MAPK1;RAF1;CDKN1B;NFKB1;FZD2;ITGA2;ATP6V0B;LAMA1;LAMA2;LAMA3;LAMA5;RBL2;TBPL2;COMP;COL9A2;COL9A3;FOXO1;COL9A1;MTOR;PPP2R2C;FZD3;LOC612475;EP300;ATP6V0D1;LOC119866928;PRKCI;BCAP31;ATP6V0E2;LLGL2;SOS2;LLGL1;WNT3;WNT2;WNT1;EIF2AK2;IFNA7;BAD;IFNA5;ATP6V1E2;WNT5A;CDK2;WNT4;SPP1;CDK6;COL1A1;ATP6V1E1;LOC119870343;RHEB;CDC42;ATP6V1G1;PPP2R1B;PTK2;UBE3A;ATP6V0C;EGFR;BAX;CTNNB1;MAP2K2;MAP2K1;WNT10B;PATJ;FZD10;RBL1;TCIRG1;ITGA10(up);CCNA2;AXIN2;AXIN1;CCNA1;FAS;NRAS;RB1;ATP6V0E1;DVL3;WNT8B;LOC119863906;PXN;PPP2R2B(up);ATP6V1B1;ATP6V1B2;JAG1;PPP2R5D;ATP6V0A1;ITGB1;ITGB3;TRAF3;ITGB5;ITGB4;ITGB7;ITGB6;IRF1;PPP2R2A;ATP6V0A2;ATP6V1G3;LOC102152234;TNF;CREB1;TNC;HES1;APC2;PRKACA;VTN;ATP6V1C1;HDAC1;PARD3;LAMA4(up);CASP3;E2F1;WNT5B;NOTCH1;CASP8;PPP2R5A;SCRIB;CREB3L3;CREB3L1;WNT6;PPP2R5B;CREB3L4;WNT16;COL6A1;ATP6V1C2;COL6A3;COL6A2;COL6A5;WNT11;COL6A6 |
| Kaposi sarcoma-associated herpesvirus infection | PTGS2;LOC100856339;PPP3R2;DLA-64;IL6ST;IKBKB;AKT1;AKT2;AKT3;IKBKE;TYK2;LEF1;CALM2;PPP3R1;CALM1;GNG14;PIK3CA;PIK3CB;PIK3CD;GNG11;GNG12;GNG13;LOC119863873;FADD;RELA;DLA-79;CREB1;CCND1;PIK3C3;KRAS;IFNAR2;SYK;TCF7L1;CSF2;GSK3B;GNG7;GNG4;GNG5;GNG2;GNG3;UBC;CCR4;GNG8;MAPK11;NFATC1;NFATC2;NFATC3;NFATC4;CXCL8(up);CDKN1A;NFKBIA;PDGFB;HIF1A;IFNGR1;BAK1;MAPKAPK2;TLR3;LOC100855681;FOS;JUN;UBA52;JAK2;PIK3R2;PIK3R1;JAK1;ANGPT2;LOC119876828;TCF7;BECN1;BECN2;LOC119870217;LOC119870216;LOC119870215;DLA88;CHUK;ATG14;ITPR2;ITPR3;ITPR1;FGF2;CALM3;IRF3;LOC100686162;TP53;STAT1;TNFRSF1A;LOC100683567;IRF9;CALML6;CALML5;CALML4;VEGFA;CREBBP;IFNAR1;RCAN1;GNB4(up);LOC102153034(down);LOC119874472(up);MAPK14;CCR1;CCR3;MAPK10;CCR5;MAPK12;HCK;CCR8;GABARAP;RAF1;LOC611406;TICAM1;STAT3;STAT2;TBK1;TRADD;IFNB1;MAPK3;MAPK1;ICAM1;C3;NFKB1;MAPK8;MAPK9;HRAS;SRC;RPS27A;GNB5;GNB1;GNB3;GNB2;MTOR;CYCS;IRF7;LOC612475;EP300;LYN;E2F2;PLCG2;LOC119876918(up);EIF2AK2;IFNA7;MAP2K1;IFNA5;IL6;CD86;CDK4;CDK6;MYC;TCF7L2;BAX;CTNNB1;MAP2K2;PLCG1;MAP2K7;MAP2K6;MAP2K4;GABARAPL1;GABARAPL2;FAS;BID;NRAS;RB1;ATG3;GNG10;TRAF2;TRAF3;RAC3;RAC1;LOC100689004;E2F3;CASP3;E2F1;PREX1;CASP8;CASP9;MAPK13;GNGT2;GNGT1;PPP3CA;PPP3CB;PPP3CC |
| Viral protein interaction with cytokine and cytokine receptor | CCL2;CCL3;CCL1;CCL7;CCL4;CCL5;CXCL8(up);CCL8;PPBP;IL20RA;IL6ST;CCR1;CCR2;CCR3;CCR4;CCR5;CCR6;CCR7;CCR8;CCR9;IL10RA;LTBR;TNFSF10;IL10RB;CXCR2;TNFSF14;XCR1;LOC119874472(up);CXCR3;CCL28;CXCR1;CXCR5;CCL21;CCL20;CCL23;CX3CL1;CCL25;CCL27;IL18RAP(up);IL19;CCR10;IL18R1;IL6R;LOC480600;LTA;TNF;CXCL13;CXCL12;CXCL14;CXCL10(down);IL18;IL2RA;IL2RB;CCL13;TNFRSF1B;CX3CR1;IL2RG;IL10;CCL19;LOC119876918(up);LOC106557449;LOC119863891(up);CSF1;ACKR3;IL6;IL2;TNFRSF14;CSF1R;IL22RA1;IL20RB;TNFRSF1A |
| Glutathione metabolism | GSR;LOC479911;LOC479912;GSTA4;PRDX6;LAP3;LOC486404;GGCT;MGST2;MGST3;LOC611366;RRM2;RRM1;OPLAH;LOC119867218;PGD;GCLM;LOC476006;GCLC;LOC481841;MGST1(down);RRM2B;LOC100685416;LOC102153601;ANPEP;GSTP1;LOC100856518;ODC1;IDH2;IDH1;LOC610304;SRM;GGT1;GGT6;GGT7;GGT5;LOC474938;HPGDS;GSTO2;GSTO1;GSTT2B;LOC477556;TXNDC12;GPX1;GPX3;GPX2;GPX5;GPX4;GPX7;GPX6;GPX8;LOC477558;GSTK1 |
| GnRH signaling pathway | ADCY1\_1;PTK2B;CALM2;CALM3;CALM1;MAP3K2;MAP3K3;CACNA1C;CACNA1D;CACNA1F;CACNA1S;CAMK2D;CAMK2G;CAMK2A;CAMK2B;ADCY4(up);KRAS;MMP2(down);ADCY5;ADCY6;ADCY7;PLD1;PLD2;ADCY3;ADCY8;ADCY9;ELK1;MAPK10;MAPK11;MMP14;HRAS;ADCY2;JUN;GRB2;LOC119869567;PLCB2;PLCB3;PLCB4;GNRH1;ITPR2;ITPR3;ITPR1;GNAQ;GNAS;CALML6;CALML5;CALML4;LOC102151131;MAP3K1;LOC102153034(down);MAPK14;GNRHR;MAP3K4;MAPK12;MAPK13;RAF1;MAPK3;CGA;HBEGF;MAPK1;MAPK7;MAPK8;MAPK9;SRC;PLCB1(up);LOC119866928;SOS2;PRKACA;PRKACB;CDC42;ADCY1;EGFR;MAP2K3;MAP2K2;MAP2K1;MAP2K7;MAP2K6;MAP2K4;FSHB;NRAS;PRKCA;PRKCB;PRKCD;PLA2G4E(up);PLA2G4B;PLA2G4A;PLA2G4F;PLA2G4D;GNA11;ATF4 |
