# Supplementary material for: Tumorigenicity decrease in Bcl-xL deficient MDCK cells ensuring the safety for influenza vaccine production
Source: PLoS One. 2024 Dec 16;19(12):e0311069. doi: 10.1371/journal.pone.0311069 (PMC11649150; doi:10.1371/journal.pone.0311069)
Supplement: S1 File — (ZIP) [file B_vs_A.down_CC_enrichment_map.pdf › S2_File/2_GO_enrichment/enrichmap/B_vs_A.down_CC_enrichment_map.pdf]

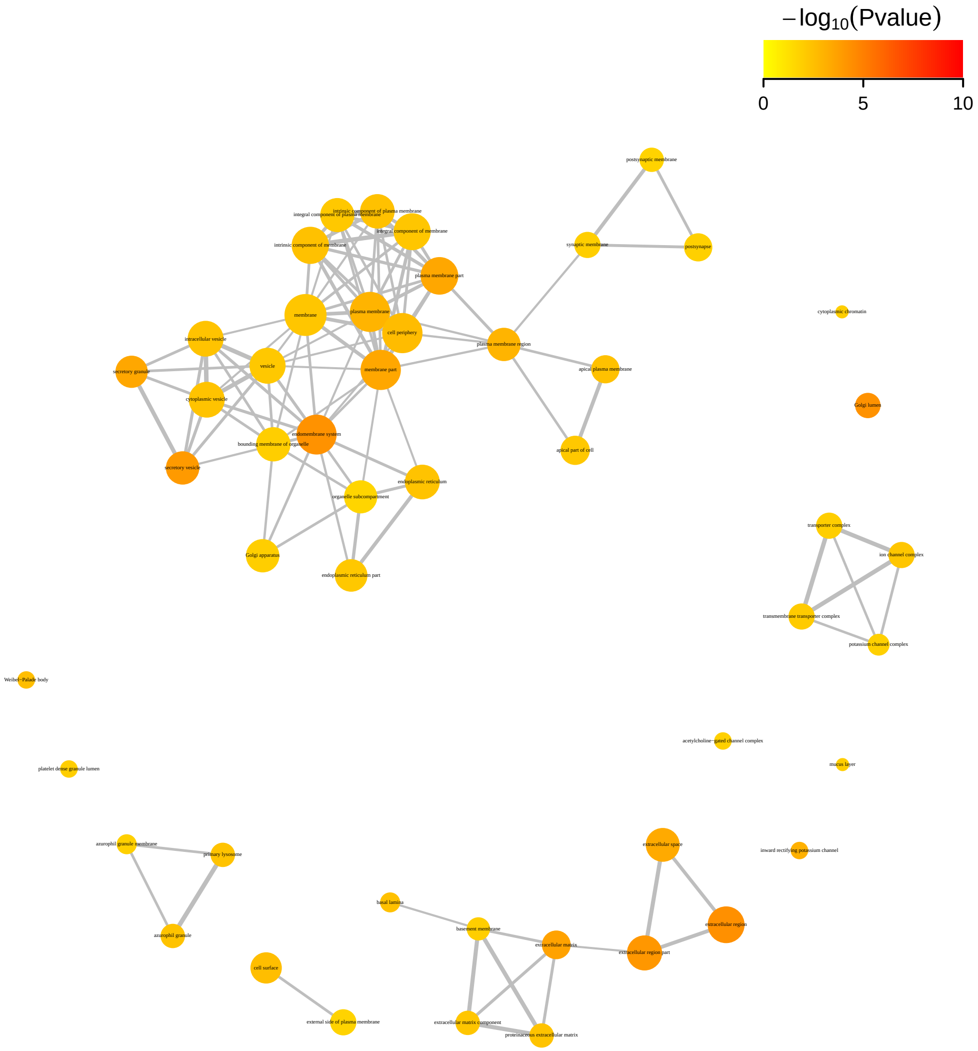

$-\log_{10}(\text{Pvalue})$
0
5
10
postsynaptic membrane
synaptic membrane
postsynapse
intrinsic component of plasma membrane
integral component of plasma membrane
integral component of membrane
intrinsic component of membrane
plasma membrane part
plasma membrane
membrane
cell periphery
plasma membrane region
apical plasma membrane
apical part of cell
cytoplasmic chromatin
Golgi lumen
intracellular vesicle
secretory granule
vesicle
membrane part
cytoplasmic vesicle
endomembrane system
bounding membrane of organelle
secretory vesicle
endoplasmic reticulum
organelle subcompartment
Golgi apparatus
endoplasmic reticulum part
transporter complex
ion channel complex
transmembrane transporter complex
potassium channel complex
Weibel−Palade body
acetylcholine−gated channel complex
mucus layer
platelet dense granule lumen
azurophil granule membrane
primary lysosome
azurophil granule
extracellular space
inward rectifying potassium channel
basal lamina
basement membrane
extracellular matrix
extracellular region
extracellular region part
cell surface
external side of plasma membrane
extracellular matrix component
proteinaceous extracellular matrix
