# Supplementary material for: Tumorigenicity decrease in Bcl-xL deficient MDCK cells ensuring the safety for influenza vaccine production
Source: PLoS One. 2024 Dec 16;19(12):e0311069. doi: 10.1371/journal.pone.0311069 (PMC11649150; doi:10.1371/journal.pone.0311069)
Supplement: S1 File — (ZIP) [file B_vs_A.sign_GO_enrichment_map.pdf › S2_File/2_GO_enrichment/enrichmap/B_vs_A.sign_GO_enrichment_map.pdf]

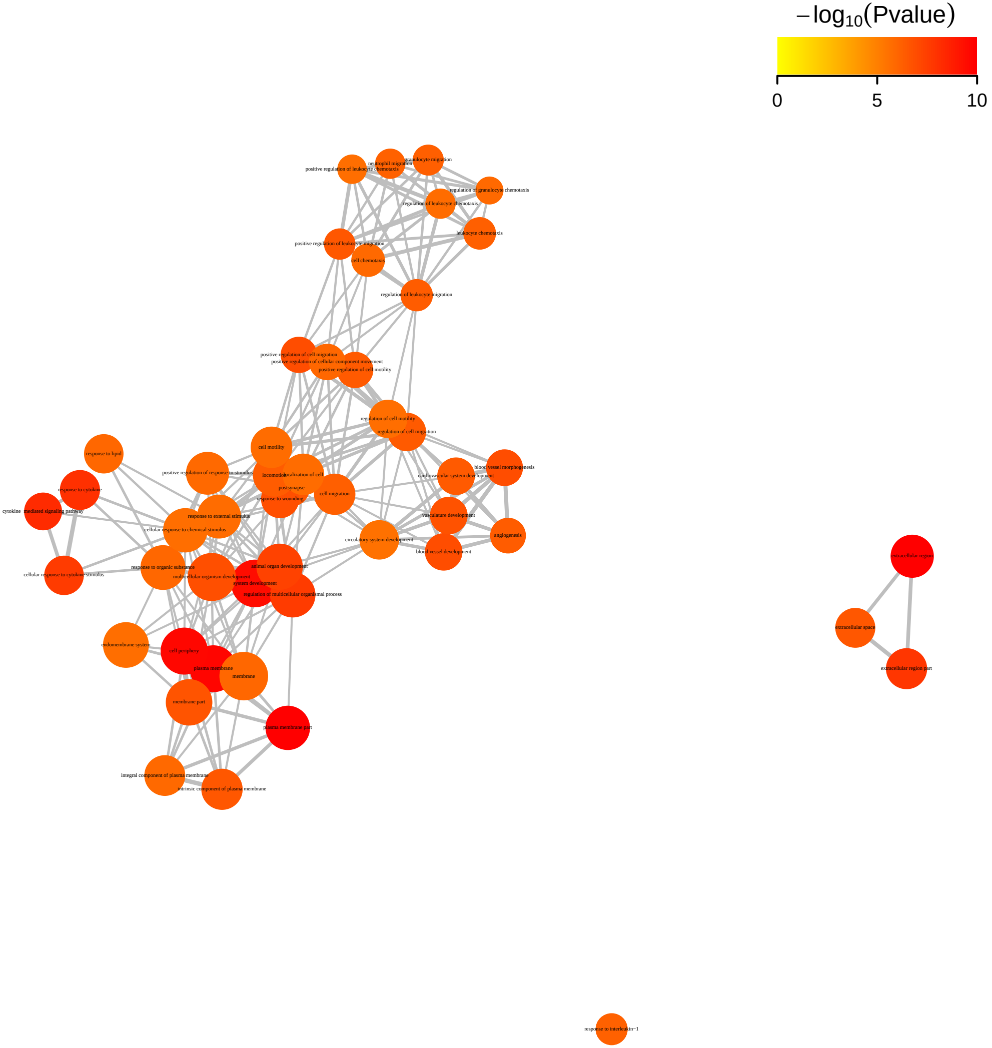

$-\log_{10}(\mathrm{Pvalue})$
0
5
10
positive regulation of leukocyte chemotaxis
neutrophil migration
granulocyte migration
regulation of granulocyte chemotaxis
regulation of leukocyte chemotaxis
leukocyte chemotaxis
positive regulation of leukocyte migration
cell chemotaxis
regulation of leukocyte migration
positive regulation of cell migration
positive regulation of cellular component movement
positive regulation of cell motility
regulation of cell motility
regulation of cell migration
blood vessel morphogenesis
cardiovascular system development
response to lipid
cell motility
positive regulation of response to stimulus
locomotion
localization of cell
postsynapse
response to cytokine
response to wounding
cell migration
cytokine−mediated signaling pathway
response to external stimulus
vasculature development
cellular response to chemical stimulus
angiogenesis
circulatory system development
blood vessel development
response to organic substance
animal organ development
multicellular organism development
system development
cellular response to cytokine stimulus
regulation of multicellular organismal process
extracellular region
extracellular space
endomembrane system
cell periphery
plasma membrane
membrane
extracellular region part
membrane part
plasma membrane part
integral component of plasma membrane
intrinsic component of plasma membrane
response to interleukin−1
