# Supplementary material for: Tumorigenicity decrease in Bcl-xL deficient MDCK cells ensuring the safety for influenza vaccine production
Source: PLoS One. 2024 Dec 16;19(12):e0311069. doi: 10.1371/journal.pone.0311069 (PMC11649150; doi:10.1371/journal.pone.0311069)
Supplement: S1 File — (ZIP) [file union.DE_gene.subcluster1_MF_enrichment_network.pdf › S2_File/2_GO_enrichment/network/union.DE_gene.subcluster1_MF_enrichment_network.pdf]

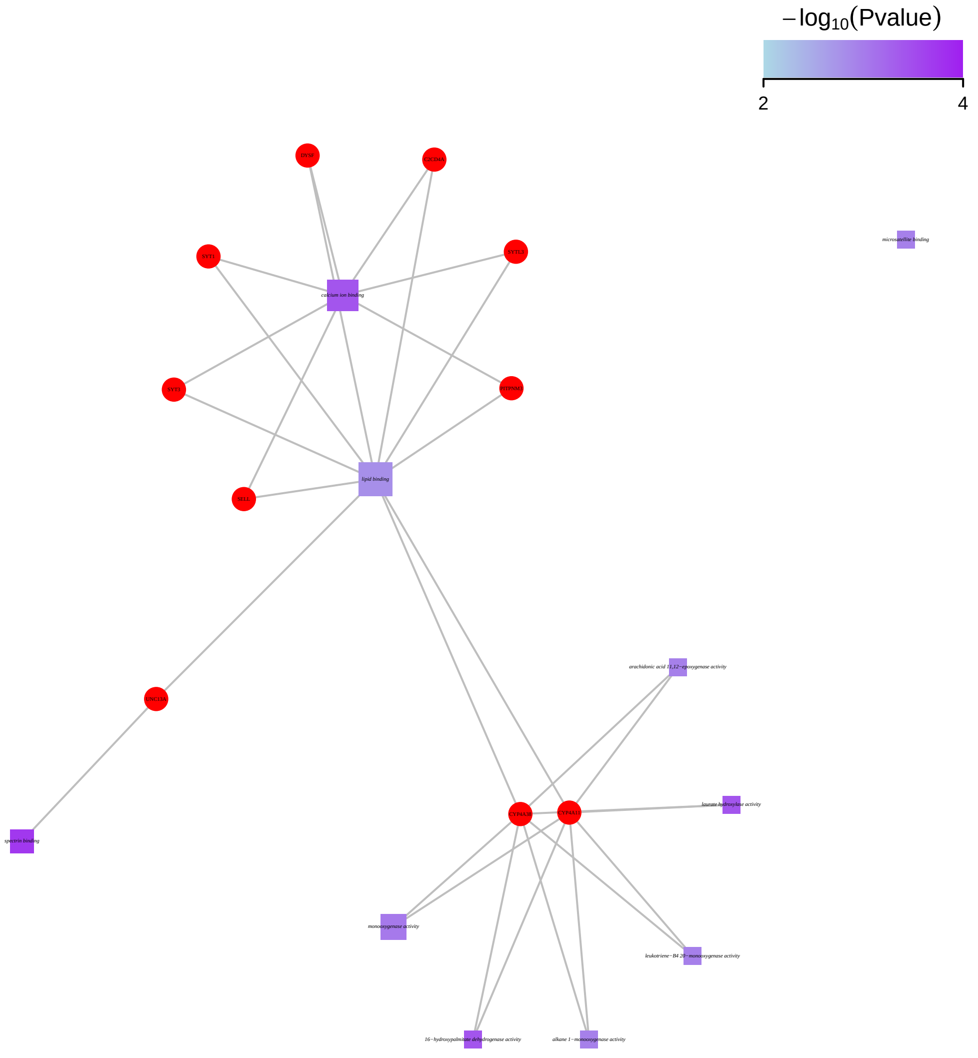
$-\log_{10}(\text{Pvalue})$
2
4
DYSF
C2CD4A
SYT1
SYTL3
calcium ion binding
SYT3
PITPNM3
SELL
lipid binding
microsatellite binding
UNC13A
spectrin binding
arachidonic acid 11,12−epoxygenase activity
CYP4A38
CYP4A11
laurate hydroxylase activity
monooxygenase activity
leukotriene−B4 20−monooxygenase activity
16−hydroxypalmitate dehydrogenase activity
alkane 1−monooxygenase activity
