# Supplementary material for: Tumorigenicity decrease in Bcl-xL deficient MDCK cells ensuring the safety for influenza vaccine production
Source: PLoS One. 2024 Dec 16;19(12):e0311069. doi: 10.1371/journal.pone.0311069 (PMC11649150; doi:10.1371/journal.pone.0311069)
Supplement: S1 File — (ZIP) [file union.DE_gene.subcluster1_KEGG_enrichment_map.pdf › S2_File/3_KEGG_enrichment/enrichmap/union.DE_gene.subcluster1_KEGG_enrichment_map.pdf]

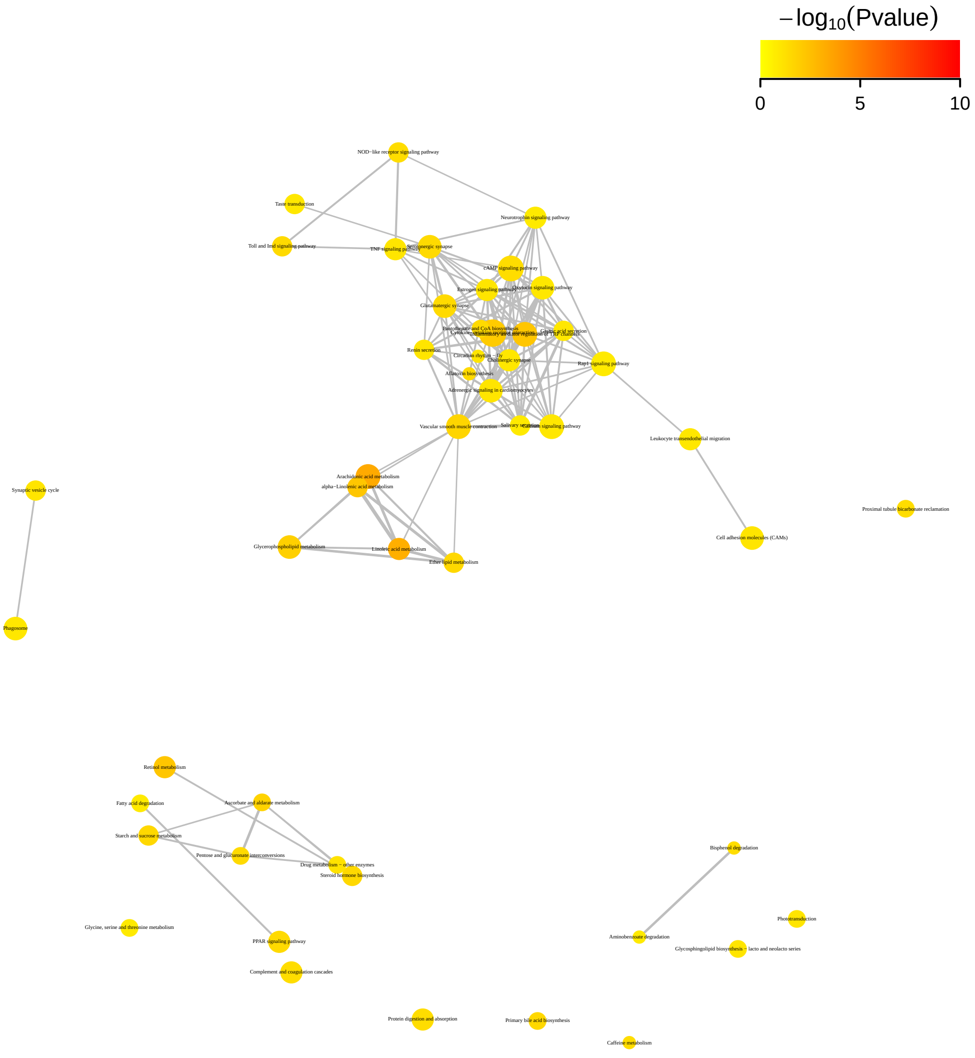

$-\log_{10}(\text{Pvalue})$
0
5
10
NOD−like receptor signaling pathway
Taste transduction
Neurotrophin signaling pathway
Toll and Imd signaling pathway
TNF signaling pathway
Serotonergic synapse
cAMP signaling pathway
Estrogen signaling pathway
Oxytocin signaling pathway
Glutamatergic synapse
Pantothenate and CoA biosynthesis
Cytokine−cytokine receptor interaction
Inflammatory mediator regulation of TRP channels
Gastric acid secretion
Renin secretion
Circadian rhythm − fly
Cholinergic synapse
Rap1 signaling pathway
Aflatoxin biosynthesis
Adrenergic signaling in cardiomyocytes
Vascular smooth muscle contraction
Salivary secretion
Calcium signaling pathway
Leukocyte transendothelial migration
Arachidonic acid metabolism
alpha−Linolenic acid metabolism
Synaptic vesicle cycle
Proximal tubule bicarbonate reclamation
Cell adhesion molecules (CAMs)
Glycerophospholipid metabolism
Linoleic acid metabolism
Ether lipid metabolism
Phagosome
Retinol metabolism
Fatty acid degradation
Ascorbate and aldarate metabolism
Starch and sucrose metabolism
Pentose and glucuronate interconversions
Drug metabolism − other enzymes
Steroid hormone biosynthesis
Bisphenol degradation
Glycine, serine and threonine metabolism
PPAR signaling pathway
Phototransduction
Aminobenzoate degradation
Glycosphingolipid biosynthesis − lacto and neolacto series
Complement and coagulation cascades
Protein digestion and absorption
Primary bile acid biosynthesis
Caffeine metabolism
