# Supplementary material for: Tumorigenicity decrease in Bcl-xL deficient MDCK cells ensuring the safety for influenza vaccine production
Source: PLoS One. 2024 Dec 16;19(12):e0311069. doi: 10.1371/journal.pone.0311069 (PMC11649150; doi:10.1371/journal.pone.0311069)
Supplement: S1 File — (ZIP) [file B_vs_A.down_KEGG_enrichment_network.pdf › S2_File/3_KEGG_enrichment/network/B_vs_A.down_KEGG_enrichment_network.pdf]

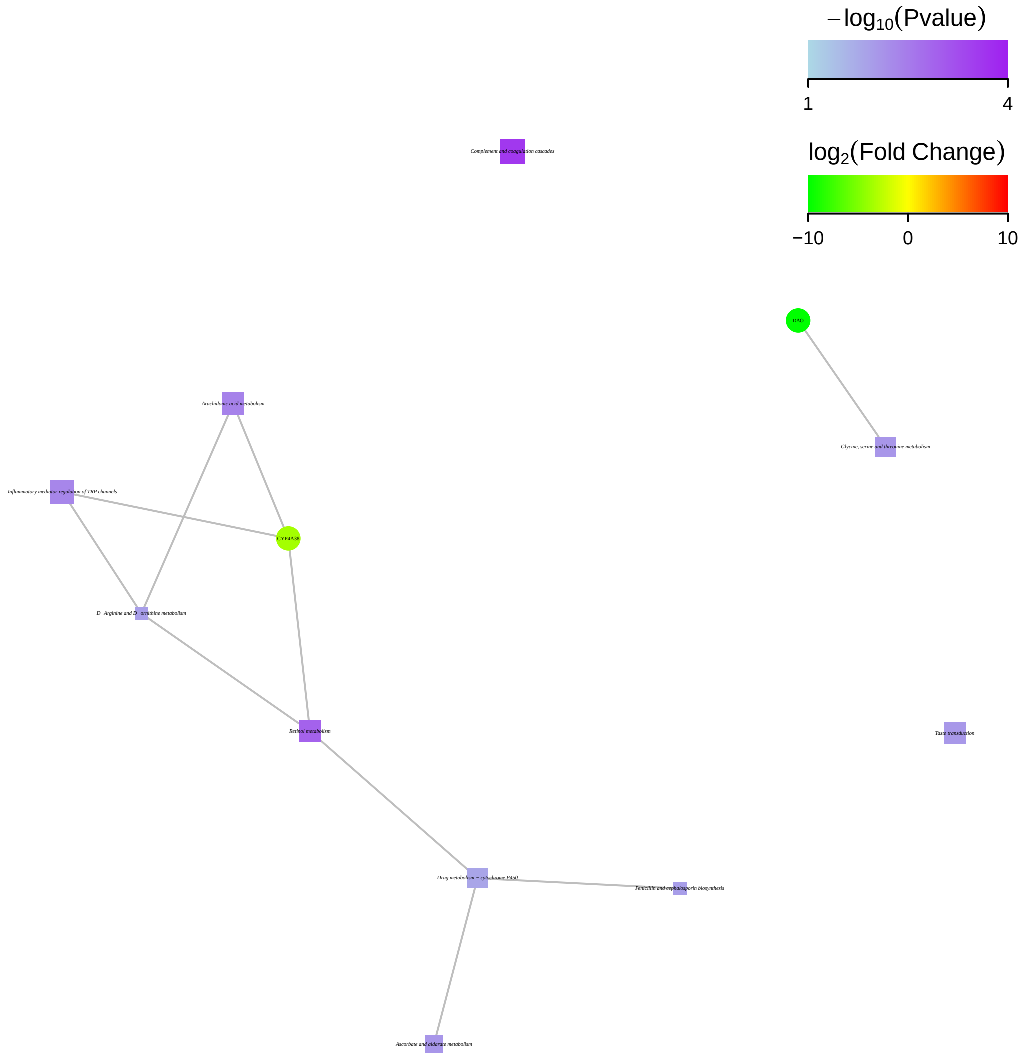

−log10(Pvalue)
1
4
log2(Fold Change)
−10
0
10
Complement and coagulation cascades
DAO
Arachidonic acid metabolism
Glycine, serine and threonine metabolism
Inflammatory mediator regulation of TRP channels
CYP4A38
D−Arginine and D−ornithine metabolism
Retinol metabolism
Taste transduction
Drug metabolism − cytochrome P450
Penicillin and cephalosporin biosynthesis
Ascorbate and aldarate metabolism
