# Supplementary material for: Tumorigenicity decrease in Bcl-xL deficient MDCK cells ensuring the safety for influenza vaccine production
Source: PLoS One. 2024 Dec 16;19(12):e0311069. doi: 10.1371/journal.pone.0311069 (PMC11649150; doi:10.1371/journal.pone.0311069)
Supplement: S1 File — (ZIP) [file union.DE_gene.subcluster1_KEGG_enrichment_network.pdf › S2_File/3_KEGG_enrichment/network/union.DE_gene.subcluster1_KEGG_enrichment_network.pdf]

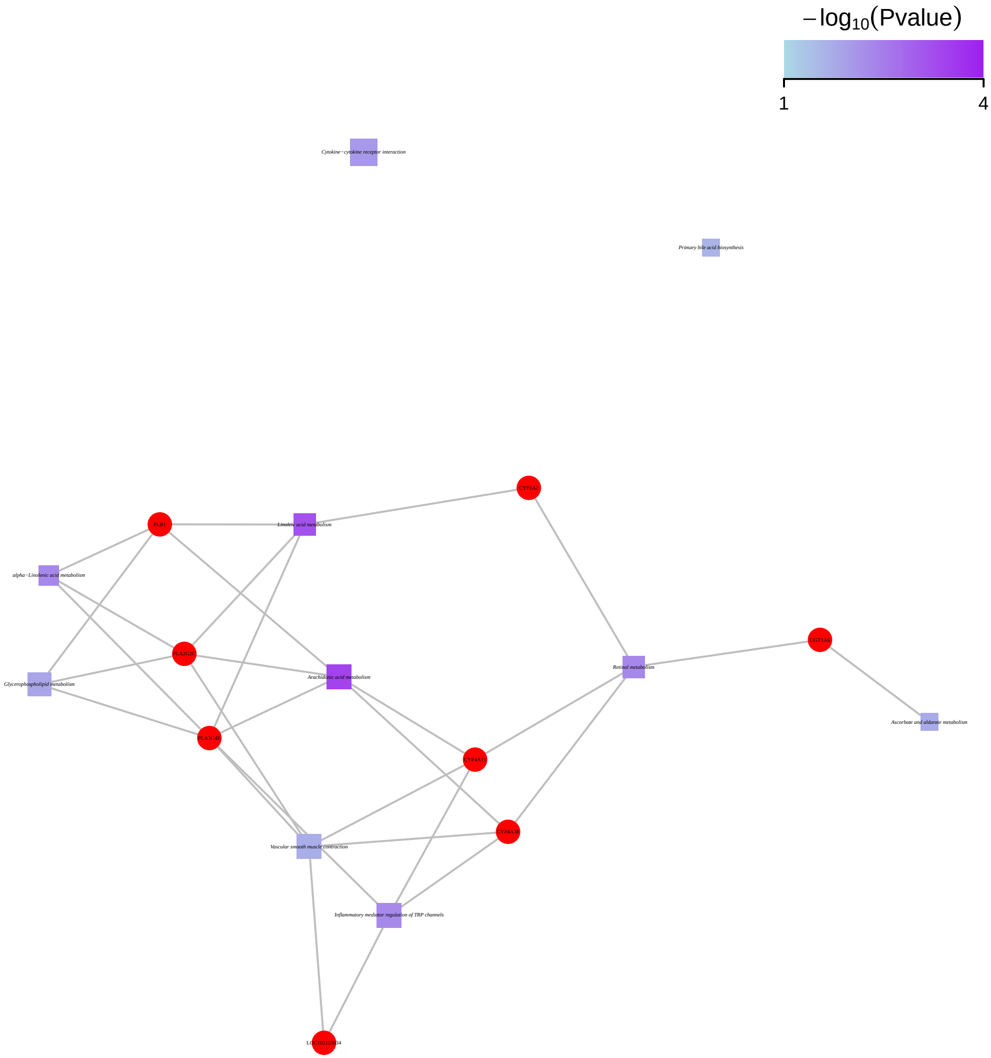

$-\log_{10}(\mathrm{Pvalue})$
1
4
Cytokine−cytokine receptor interaction
Primary bile acid biosynthesis
CYP1A2
PLB1
Linoleic acid metabolism
alpha−Linolenic acid metabolism
PLA2G2C
Arachidonic acid metabolism
Retinol metabolism
UGT1A6
Glycerophospholipid metabolism
PLA2G4E
Ascorbate and aldarate metabolism
CYP4A11
CYP4A38
Vascular smooth muscle contraction
Inflammatory mediator regulation of TRP channels
LOC102153034
